# Multiethnic meta-analysis identifies ancestry-specific and cross-ancestry loci for pulmonary function

**DOI:** 10.1038/s41467-018-05369-0

**Published:** 2018-07-30

**Authors:** Annah B. Wyss, Tamar Sofer, Mi Kyeong Lee, Natalie Terzikhan, Jennifer N. Nguyen, Lies Lahousse, Jeanne C. Latourelle, Albert Vernon Smith, Traci M. Bartz, Mary F. Feitosa, Wei Gao, Tarunveer S. Ahluwalia, Wenbo Tang, Christopher Oldmeadow, Qing Duan, Kim de Jong, Mary K. Wojczynski, Xin-Qun Wang, Raymond Noordam, Fernando Pires Hartwig, Victoria E. Jackson, Tianyuan Wang, Ma’en Obeidat, Brian D. Hobbs, Tianxiao Huan, Hongsheng Gui, Margaret M. Parker, Donglei Hu, Lauren S. Mogil, Gleb Kichaev, Jianping Jin, Mariaelisa Graff, Tamara B. Harris, Ravi Kalhan, Susan R. Heckbert, Lavinia Paternoster, Kristin M. Burkart, Yongmei Liu, Elizabeth G. Holliday, James G. Wilson, Judith M. Vonk, Jason L. Sanders, R. Graham Barr, Renée de Mutsert, Ana Maria Baptista Menezes, Hieab H. H. Adams, Maarten van den Berge, Roby Joehanes, Albert M. Levin, Jennifer Liberto, Lenore J. Launer, Alanna C. Morrison, Colleen M. Sitlani, Juan C. Celedón, Stephen B. Kritchevsky, Rodney J. Scott, Kaare Christensen, Jerome I. Rotter, Tobias N. Bonten, Fernando César Wehrmeister, Yohan Bossé, Shujie Xiao, Sam Oh, Nora Franceschini, Jennifer A. Brody, Robert C. Kaplan, Kurt Lohman, Mark McEvoy, Michael A. Province, Frits R. Rosendaal, Kent D. Taylor, David C. Nickle, L. Keoki Williams, Esteban G. Burchard, Heather E. Wheeler, Don D. Sin, Vilmundur Gudnason, Kari E. North, Myriam Fornage, Bruce M. Psaty, Richard H. Myers, George O’Connor, Torben Hansen, Cathy C. Laurie, Patricia A. Cassano, Joohon Sung, Woo Jin Kim, John R. Attia, Leslie Lange, H. Marike Boezen, Bharat Thyagarajan, Stephen S. Rich, Dennis O. Mook-Kanamori, Bernardo Lessa Horta, André G. Uitterlinden, Hae Kyung Im, Michael H. Cho, Guy G. Brusselle, Sina A. Gharib, Josée Dupuis, Ani Manichaikul, Stephanie J. London

**Affiliations:** 1 Epidemiology Branch National Institute of Environmental Health Sciences, National Institutes of Health, US Department of Health and Human Services, Research Triangle Park, NC, 27709 USA; 2 Division of Sleep and Circadian Disorders, Brigham and Women’s Hospital, Boston, MA 02115 USA; 3 Department of Medicine, Harvard Medical School, Boston, MA 02115 USA; 4 Department of Respiratory Medicine, Ghent University Hospital, Ghent, 9000 Belgium; 5 Department of Epidemiology, Erasmus University Medical Center, Rotterdam, 3000 CA The Netherlands; 6 Center for Public Health Genomics, University of Virginia, Charlottesville, VA 22908 USA; 7 Department of Bioanalysis, FFW, Ghent University, Ghent, 9000 Belgium; 8 Department of Neurology, Boston University School of Medicine, Boston, MA 02118 USA; 9 Icelandic Heart Association, Kopavogur, 201 Iceland; 10 Faculty of Medicine, University of Iceland, Reykjavik, 101 Iceland; 11 Department of Biostatistics, University of Michigan, Ann Arbor, MI 48109 USA; 12 Cardiovascular Health Research Unit, Department of Medicine, University of Washington, Seattle, WA 98101 USA; 13 Department of Biostatistics, University of Washington, Seattle, WA 98195 USA; 14 Division of Statistical Genomics, Department of Genetics, Washington University School of Medicine, St Louis, MO 63110 USA; 15 Department of Biostatistics, Boston University School of Public Health, Boston, MA 02118 USA; 16 The Novo Nordisk Foundation Center for Basic Metabolic Research, Metabolic Genetics Section, Faculty of Health and Medical Sciences, University of Copenhagen, Copenhagen, 2200 Denmark; 17 Steno Diabetes Center Copenhagen, Gentofte, 2820 Denmark; 18 Division of Nutritional Sciences, Cornell University, Ithaca, NY 14853 USA; 19 Hunter Medical Research Institute and Faculty of Health, University of Newcastle, Callaghan, NSW 2305 Australia; 20 Department of Genetics, University of North Carolina, Chapel Hill, NC 27599 USA; 21 Department of Epidemiologie, University of Groningen, University Medical Center Groningen, 9713 GZ Groningen, Netherlands; 22 Division of Biostatistics, Department of Public Health Sciences, University of Virginia, Charlottesville, VA 22908 USA; 23 Department of Internal Medicine, Section Gerontology and Geriatrics, Leiden University Medical Center, Leiden, 2300 RC The Netherlands; 24 Postgraduate Program in Epidemiology, Federal University of Pelotas, 96020-220 Pelotas, Brazil; 25 Medical Research Council Integrative Epidemiology Unit, School of Social and Community Medicine, University of Bristol, Bristol, BS8 2BN UK; 26 Department of Health Sciences, University of Leicester, Leicester, LE1 7RH UK; 27 Integrative Bioinformatics Support Group National Institute of Environmental Health Sciences, National Institutes of Health, US Department of Health and Human Services, Research Triangle Park, NC, 27709 USA; 28 The University of British Columbia Center for Heart Lung Innovation, St Paul’s Hospital, Vancouver, BC V6Z 1Y6 Canada; 29 Channing Division of Network Medicine, Brigham and Women’s Hospital, Boston, MA 02115 USA; 30 Division of Pulmonary and Critical Care Medicine, Brigham and Women’s Hospital, Boston, MA 02115 USA; 31 The Population Sciences Branch, Division of Intramural Research, National Heart, Lung, and Blood Institute, Bethesda, MD 20892 USA; 32 Center for Health Policy and Health Services Research, Henry Ford Health System, Detroit, MI 48202 USA; 33 School of Medicine, University of California San Francisco, San Francisco, CA 94143 USA; 34 Department of Biology, Loyola University Chicago, Chicago, IL 60660 USA; 35 University of California Los Angeles, Los Angeles, CA 90095 USA; 36 Westat, Durham, NC 27703 USA; 37 Department of Epidemiology, University of North Carolina, Chapel Hill, NC 27599 USA; 38 Department of Health and Human Services, Laboratory of Epidemiology and Population Sciences, National Institute on Aging, National Institutes of Health, Bethesda, MD 20892 USA; 39 Division of Pulmonary and Critical Care Medicine, Northwestern University Feinberg School of Medicine, Chicago, IL 60611 USA; 40 Department of Epidemiology, Cardiovascular Health Research Unit, University of Washington, Seattle, WA 98101 USA; 41 Division of Pulmonary, Allergy and Critical Care Medicine, Department of Medicine, College of Physicians and Surgeons, Columbia University, New York, NY 10032 USA; 42 Wake Forest School of Medicine, Winston-Salem, NC 27101 USA; 43 Department of Physiology and Biophysics, University of Mississippi Medical Center, Jackson, MS 39216 USA; 44 Department of Medicine, Massachusetts General Hospital, Boston, MA 02114 USA; 45 Department of Medicine, College of Physicians and Surgeons, Columbia University, New York, NY 10032 USA; 46 Department of Epidemiology, Mailman School of Public Health, Columbia University, New York, NY 10032 USA; 47 Department of Clinical Epidemiology, Leiden University Medical Center, Leiden, 2300 RC The Netherlands; 48 Department of Radiology, Erasmus University Medical Center, Rotterdam, 3015 GD The Netherlands; 49 Department of Pulmonary Diseases, University of Groningen, University Medical Center Groningen, Groningen, 9700 AB The Netherlands; 50 Hebrew SeniorLife, Harvard University, Boston, MA 02131 USA; 51 Department of Public Health Sciences, Henry Ford Health System, Detroit, MI 48202 USA; 52 Human Genetics Center, Department of Epidemiology, Human Genetics, and Environmental Sciences, School of Public Health, The University of Texas Health Science Center at Houston, Houston, TX 77030 USA; 53 Division of Pulmonary Medicine, Allergy, and Immunology, Department of Pediatrics, Children’s Hospital of Pittsburgh of UPMC, University of Pittsburgh, Pittsburgh, PA 15224 USA; 54 Sticht Center for Healthy Aging and Alzheimer’s Prevention, Wake Forest School of Medicine, Winston-Salem, NC 27157 USA; 55 Division of Molecular Medicine, Pathology North, NSW Health Pathology, Newcastle, NSW 2305 Australia; 56 Department of Epidemiology, Biostatistics and Biodemography, University of Southern Denmark, Odense, 5000 Denmark; 57 Department of Pediatrics, Institute for Translational Genomics and Population Sciences, Los Angeles Biomedical Research Institute, Harbor-UCLA Medical Center, Torrance, CA 90502 USA; 58 Department of Public Health and Primary Care, Leiden University Medical Center, Leiden, 2300 RC The Netherlands; 59 Department of Pulmonology, Leiden University Medical Center, Leiden, 2300 RC The Netherlands; 60 Department of Molecular Medicine, Institut Universitaire de Cardiologie et de Pneumologie de Québec, Laval University, Québec, G1V 4G5 Canada; 61 Department of Epidemiology and Population Health, Albert Einstein College of Medicine, Bronx, NY 10461 USA; 62 Merck Research Laboratories, GpGx, Merck & Co., Inc., Kenilworth, NJ 07033 USA; 63 Department of Internal Medicine, Henry Ford Health System, Detroit, MI 48202 USA; 64 School of Pharmacy, University of California San Francisco, San Francisco, CA 94143 USA; 65 Respiratory Division, Department of Medicine, University of British Columbia, Vancouver, BC V5Z 1M9 Canada; 66 Brown Foundation Institute of Molecular Medicine and Human Genetics Center, University of Texas Health Science Center at Houston, Houston, TX 77030 USA; 67 Cardiovascular Health Research Unit, Department of Health Services, University of Washington, Seattle, WA 98101 USA; 68 Kaiser Permanente Washington Health Research Institute, Seattle, WA 98101 USA; 69 National Heart, Lung, and Blood Institute’s Framingham Heart Study, Framingham, MA 01702 USA; 70 Pulmonary Center, Department of Medicine, Boston University School of Medicine, Boston, MA 02118 USA; 71 Department of Healthcare Policy and Research, Weill Cornell Medical College, New York, NY 10065 USA; 72 Department of Health Science, School of Public Health, Seoul National University, Seoul, 08826 South Korea; 73 Department of Internal Medicine and Environmental Health Center, Kangwon National University, Chuncheon, 24341 South Korea; 74 University of Colorado Denver, Denver, CO 80204 USA; 75 Department of Laboratory Medicine and Pathology, University of Minnesota, Minneapolis, MN 55455 USA; 76 Department of Internal Medicine, Erasmus University Medical Center, Rotterdam, 3015 CN The Netherlands; 77 Section of Genetic Medicine, The University of Chicago, Chicago, IL 60637 USA; 78 Department of Respiratory Medicine, Erasmus University Medical Center, Rotterdam, 3000 CA The Netherlands; 79 Department of Medicine, Computational Medicine Core, Center for Lung Biology, UW Medicine Sleep Center, University of Washington, Seattle, WA 98109 USA

## Abstract

Nearly 100 loci have been identified for pulmonary function, almost exclusively in studies of European ancestry populations. We extend previous research by meta-analyzing genome-wide association studies of 1000 Genomes imputed variants in relation to pulmonary function in a multiethnic population of 90,715 individuals of European (*N* = 60,552), African (*N* = 8429), Asian (*N* = 9959), and Hispanic/Latino (*N* = 11,775) ethnicities. We identify over 50 additional loci at genome-wide significance in ancestry-specific or multiethnic meta-analyses. Using recent fine-mapping methods incorporating functional annotation, gene expression, and differences in linkage disequilibrium between ethnicities, we further shed light on potential causal variants and genes at known and newly identified loci. Several of the novel genes encode proteins with predicted or established drug targets, including *KCNK2* and *CDK12*. Our study highlights the utility of multiethnic and integrative genomics approaches to extend existing knowledge of the genetics of lung function and clinical relevance of implicated loci.

## Abstract (web summary)

Pulmonary function is influenced by environmental factors, lifestyle, and genetics. Here, in a multiethnic GWAS meta-analysis for pulmonary function traits, the authors identify over 50 additional genetic loci, a subset of which are specific for European, African, Asian, or Hispanic/Latino ancestry.

## Introduction

Pulmonary function traits (PFTs), including forced expiratory volume in the first second (FEV_1_) and forced vital capacity (FVC), and their ratio FEV_1_/FVC, are important clinical measures for assessing respiratory health, diagnosing chronic obstructive pulmonary disease (COPD), and monitoring the progression and severity of various other lung conditions. Further, even when within the normal range, these parameters are related to mortality, independently of standard risk factors^1–3^.

In addition to lifestyle and environmental factors, such as smoking and air pollution, genetics influences pulmonary function^4–6^. Previous genome-wide association studies (GWAS) have identified nearly 100 loci associated with PFTs^7–15^. These analyses have been primarily conducted using HapMap imputed data among European ancestry populations^7–12^. Recently, the UK BiLEVE Study (*N* = 48,943) and SpiroMeta Consortium (*N* = 38,199) have also examined associations between 1000 Genomes imputed variants and PFTs, but only among Europeans^13–15^.

The present cohorts for heart and aging research in genomic epidemiology (CHARGE) meta-analysis builds upon previous studies by examining PFTs in relation to the more comprehensive 1000 Genomes panel in a larger study population (90,715 individuals from 22 studies, Table 1) comprised of multiple ancestral populations: European (60,552 individuals from 18 studies), African (8429 individuals from 7 studies), Asian (9959 individuals from 2 studies), and Hispanic/Latino (11,775 individuals from 6 ethnic background groups in 1 study). Along with look-up of our top findings in existing analyses of lung function traits and COPD, we additionally investigate correlation with gene expression in datasets from blood and lung tissue, identify known or potential drug targets for newly identified lung function associated loci, and assess the potential biological importance of our findings using recently developed methods integrating linkage disequilibrium (LD), functional annotation, gene expression, and the multiethnic nature of our data. By conducting a GWAS meta-analysis in a large multiethnic population and employing recently developed integrative genomic methods, we identify over 50 additional loci associated with pulmonary function, including some with functional or clinical relevance.

**Table 1:** Sample size and location of studies included in the CHARGE consortium 1000 Genomes and pulmonary function meta-analysis

Study^a^	Country	Sample size by ancestry			
		European	African	Hispanic/Latino	Asian
AGES^b^	Iceland	1620			
ALHS	United States	2844			
ARIC^b^	United States	8878	1837		
CARDIA^b^	United States	1580	883		
CHS^b^	United States	3135	566		
FamHS	United States	1679			
FHS^b^	United States	7689			
GOYA	Denmark	1456			
HCHS/SOL	United States			11775	
HCS^b^	Australia	1822			
Health ABC^b^	United States	1472	943		
Healthy Twin	South Korea				2098
JHS	United States		2015		
KARE3	South Korea				7861
LifeLines^b^	Netherlands	11851			
LLFS^b^	United States and Denmark	3787			
MESA^b^	United States	1339	863		
NEO	Netherlands	5460			
1982 Pelotas	Brazil	1357	1322		
RSI^b^	Netherlands	1232			
RSII^b^	Netherlands	1135			
RSIII^b^	Netherlands	2216			
	Total	60,552	8429	11,775	9959

^a^*AGES* Age Gene Environment Susceptibility Study; *ALHS* Agricultural Lung Health Study (1180 asthma cases and 1664 controls); *ARIC* Atherosclerosis Risk in Communities Study; CARDIA coronary artery risk development in young adults; *CHS* Cardiovascular Health Study; *FamHS* Family Heart Study; *FHS* Framingham Heart Study; *GOYA* Genetics of Overweight Young Adults Study (670 obese cases and 786 controls); *HCHS/SOL* Hispanic Community Health Study/Study of Latinos; *HCS* Hunter Community Study; *JHS* Jackson Heart Study; *KARE3* Korean Association Resource Phase 3 Study; *LLFS* Long Life Family Study; *MESA* Multi-Ethnic Study of Atherosclerosis; *NEO* Netherlands Epidemiology of Obesity Study; *RS* Rotterdam Study

^b^Studies included in one or more previous CHARGE papers: Hancock et al. (2010) included ARIC, CHS, FHS, RSI, and RSII; Soler Artigas et al. (2011) included AGES, ARIC, CHS, FHS, Health ABC, RSI, and RSII in stage 1 and HCS, CARDIA, LifeLines, MESA, and RSIII in stage 2; and Loth et al. (2014) included AGES, ARIC, CARDIA, CHS, FHS, Health ABC, HCS, MESA, RSI, RSII, and RSIII in stage 1 and LifeLines and LLFS in stage 2

## Results

### Ancestry-specific meta-analyses

Each study used linear regression to model the additive effect of variants on PFTs, adjusting for age, sex, height, cigarette smoking, weight (for FVC only), and center, ancestral principal components, and a random familial effect to account for family relatedness when appropriate. Ancestry-specific fixed-effects inverse-variance weighted meta-analyses of study-specific results, with genomic control correction, were conducted in METAL (http://www.sph.umich.edu/csg/abecasis/metal/). Meta-analyses included approximately 11.1 million variants for European ancestry, 18.1 million for African ancestry, 4.2 million variants for Asian ancestry, and 13.8 million for Hispanic/Latino ethnicity (see Methods).

**Fig. 1:**
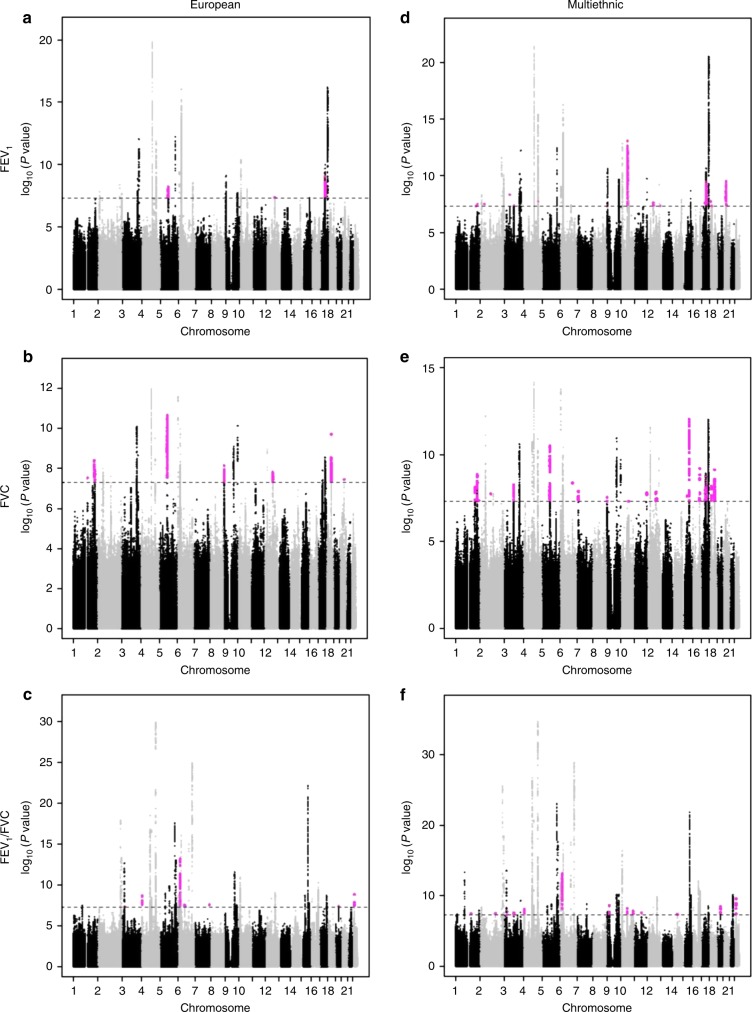
Manhattan plots of genome-wide association results for pulmonary function in the following CHARGE meta-analyses: **a** FEV_1_ European ancestry; **b** FVC European ancestry; **c** FEV_1_ /FVC European ancestry; **d** FEV multiethnic; **e** FVC multiethnic; **f** FEV_1_ /FVC multiethnic. Novel loci indicated by magenta. Significance level (5x10^‒8^) indicated by dashed line

European ancestry meta-analyses identified 17 novel loci (defined as more than 500 kb in either direction from the top variant of a known locus as has been used in previous multiethnic GWAS^16^), which were significantly (defined as *P* < 5.0 × 10^−8^ ^14,17^) associated with pulmonary function: two loci for FEV_1_ only, 6 loci for FVC only, 7 loci for FEV_1_/FVC only, and two loci for both FEV_1_ and FVC (Table 2, Fig. 1, Supplementary Figures 1 and 2). The African ancestry meta-analysis identified eight novel loci significantly associated with pulmonary function: two loci for FEV_1_, one locus for FVC, and five loci for FEV_1_/FVC (Table 3, Supplementary Figures 1–3). Five of these loci were also significant at a stricter *P* < 2.5 × 10^−8^ threshold as has been suggested for populations of African ancestry^17^. Six of the African ancestry loci were identified based on variants with low allele frequencies (0.01–0.02) in African ancestry and which were monomorphic or nearly monomorphic (allele frequency < 0.004) in other ancestries (European, Asian, and Hispanic; Supplementary Table 1). In the Hispanic/Latino ethnicity meta-analysis, we identified one novel locus for FVC (Table 3, Supplementary Figures 1–3). Another locus was significantly associated with FEV_1_, but this region was recently reported by the Hispanic Community Health Study/Study of Latinos (HCHS/SOL)^18^. For FEV_1_/FVC among Hispanics/Latinos, all significant variants were in loci identified in previous studies of European ancestry populations. In the Asian ancestry meta-analysis, all variants significantly associated with PFTs were also in loci previously identified among European ancestry populations (Supplementary Figure 3). Within each ancestry, variants discovered for one PFT were also looked-up for associations with the other two PFTs (Supplementary Table 2).

**Table 2:** Top variants from novel loci discovered in European ancestry meta-analysis of pulmonary function in the CHARGE consortium

Nearest gene(s)^a^	Trait^b^	Top variant	Chr:Pos	Coded allele	Allele freq	*N*	Beta^c^	SE	*P* value
*LOC728989*	FVC	rs12724426	1:146494027	a	0.21	31315	−36.75	6.63	2.95E−08
*CENPF*, *KCNK2*	FVC	rs512597	1:215095003	t	0.81	60507	−24.26	4.12	3.92E−09
*C1orf140*, *DUSP10*	FVC	rs6657854	1:221630555	a	0.72	60508	−19.89	3.49	1.18E−08
*RBMS3*	FEV_1_/FVC	rs17666332	3:29469675	t	0.72	60531	0.003	0.0005	4.76E−08
*AFAP1*	FEV_1_/FVC	rs28520091	4:7846240	t	0.48	60527	0.003	0.0004	2.17E−09
*AP3B1*	FEV_1_	rs252746	5:77392117	a	0.78	60551	20.05	3.45	6.19E−09
	FVC	rs12513481	5:77450828	c	0.23	60507	−25.01	3.74	2.15E−11
*LINC00340*	FEV_1_/FVC	rs1928168	6:22017738	t	0.51	60522	0.003	0.0004	6.74E−14
*SLC25A51P1*, *BAI3*	FEV_1_/FVC	rs9351637	6:67863782	t	0.61	60528	0.002	0.0004	2.89E−08
*CNTNAP2*	FEV_1_/FVC	rs1404154	7:146651409	t	0.99	23748	−0.03	0.006	2.80E−08
*DMRT2*, *SMARCA2*	FVC	rs771924	9:1555835	a	0.42	60507	−18.40	3.18	7.16E−09
*ALX1*, *RASSF9*	FEV_1_	rs10779158	12:85724096	a	0.34	60550	15.89	2.90	4.36E−08
	FVC	rs10779158	12:85724096	a	0.34	60506	18.72	3.31	1.52E−08
*LOC644172*, *CRHR1*	FEV_1_	rs143246821	17:43685698	a	0.79	39416	30.58	4.99	9.06E−10
*WNT3*	FEV_1_	rs916888	17:44863133	t	0.75	60551	20.53	3.48	3.76E−09
*DCC*	FVC	rs8089865	18:50957922	a	0.59	60509	20.57	3.23	1.95E−10
*TSHZ3*	FEV_1_/FVC	rs1353531	19:31846907	t	0.14	60530	−0.003	0.0006	4.53E−08
*EYA2*	FVC	rs2236519	20:45529571	a	0.38	60508	−18.06	3.28	3.51E−08
*KLHL22*, *MED15*	FEV_1_/FVC	rs4820216	22:20854161	t	0.15	60528	−0.004	0.0006	1.53E−09

^a^Nearest gene: indicates gene either harboring the variant or nearest to it. *C1orf140/DUSP10* locus also includes *HLX*. *CRHR1*/*LOC644172* locus also includes *ARHGAP27*, *MGC57346*, *CRHR1-IT1*, *LRRC37A4P*. *KLHL22/MED15* locus also includes *ZNF74*, *SCARF2*.

^b^Phenotypes: FEV_1_ forced expiratory volume in 1 s (in ml), FVC forced vital capacity (in ml), Ratio FEV_1_/FVC (as a proportion)

^c^Additive effect of variant on pulmonary function, adjusting for age, age^2^, sex, height, height^2^, smoking status, pack-years of smoking, weight (for FVC only), and center, ancestral principal components, and a random familial effect to account for family relatedness when appropriate

**Table 3:** Top variants from novel loci discovered in African ancestry and Hispanic/Latino ethnicity meta-analyses of pulmonary function in the CHARGE consortium

Nearest gene(s)^a^	Trait^b^	Top variant	Chr:Pos	Coded allele	Allele freq	*N*	Beta^c^	SE	*P* value
African ancestry									
*RYR2*	FEV_1_	rs3766889	1:237941781	t	0.82	8428	52.21	9.52	4.12E−08
*C2orf48*, *HPCAL1*	FEV_1_/FVC	rs139215025	2:10418806	a	0.01	5653	−0.07	0.01	9.03E−11
*EN1*, *MARCO*	FVC	rs114962105	2:119660943	a	0.98	7099	178.48	32.44	3.77E−08
*CADPS*	FEV_1_/FVC	rs111793843	3:62386350	t	0.01	7857	−0.05	0.008	1.97E−08
*ANKRD55*, *MAP3K1*	FEV_1_	rs11748173	5:55922145	t	0.21	8429	67.07	10.72	3.91E−10
*HDC*	FEV_1_/FVC	rs180930492	15:50555681	t	0.01	3852	−0.07	0.01	2.59E−09
*LOC283867*, *CDH5*	FEV_1_/FVC	rs144296676	16:66060569	t	0.99	6536	−0.03	0.006	5.35E−09
*CPT1C*	FEV_1_/FVC	rs147472287	19:50213396	t	0.01	5653	−0.05	0.009	3.25E−08
Hispanic/Latino ethnicity									
*DKFZp686O1327*, *PABPC1P2*	FVC	rs6746679	2:147046592	a	0.56	11,759	−37.36	6.67	2.17E−08

^a^Nearest gene: indicates gene either harboring the variant or nearest to it.

^b^Phenotypes: *FEV*_*1*_ forced expiratory volume in 1 s (in ml), *FVC* forced vital capacity (in ml), ratio FEV_1_/FVC (as a proportion).

^c^Additive effect of variant on pulmonary function, adjusting for age, age^2^, sex, height, height^2^, smoking status, pack-years of smoking, weight (for FVC only), and center, ancestral principal components, and a random familial effect to account for family relatedness when appropriate

### Multiethnic meta-analysis

In multiethnic fixed-effects meta-analyses of 10.9 million variants, we identified 47 novel loci significantly associated with pulmonary function. Thirteen of these loci were also identified in the ancestry-specific meta-analyses, while 34 were uniquely identified in the multiethnic meta-analysis: 11 loci for FEV_1_ only, 14 loci for FVC only, 7 loci for FEV_1_/FVC only, 1 locus for FEV_1_ and FEV_1_/FVC, and 1 locus for all three phenotypes (Tables 4–6, Fig. 1, Supplementary Figures 1–2). Although many of the 34 loci uniquely identified in the multiethnic meta-analysis were just shy of significance in the European ancestry meta-analysis, and therefore benefited from the additional sample size of the multiethnic meta-analysis, some multiethnic loci contained variants near genome-wide significance in at least one other ancestry-specific meta-analysis with some nominal significance (*P* < 0.05) in the remaining ancestry-specific meta-analyses (Supplementary Table 3). For example, rs7899503 in *JMJD1C* was significantly associated with FEV_1_ in the multiethnic meta-analysis (*β* = 21.16 ml, *P* = 8.70 × 10^−14^) and had the following ancestry-specific results: Asian *β* = 28.29 ml, *P* = 4.56 × 10^−7^; European *β* = 17.35 ml, *P* = 1.35 × 10^−5^; Hispanic *β* = 19.86 ml, *P* = 0.002; African *β* = 29.14 ml, *P* = 0.03; *I*^2^ = 0 and *P*_heterogeneity_ = 0.40 across the four ancestry-specific results.

**Table 4:** Top variants from novel loci discovered in multiethnic meta-analysis of FEV_1_^a^ in the CHARGE consortium

Nearest gene(s)^b,c^	Top variant	Chr:Pos	Coded allele^d^	Allele freq	*N*	Beta^e^	SE	*P* value
*PIK3C2B*	rs12092943	1:204434927	t	0.74	90703	−14.57	2.67	4.83E−08
*C1orf140*, *DUSP10*	1:221765779:C_CA	1:221765779	i	0.12	55548	−36.25	6.57	3.38E−08
*PKDCC*, *EML4*	rs963406	2:42355947	a	0.12	80755	−23.13	4.18	3.17E−08
*DNAH12*	rs79294353	3:57494433	a	0.92	79170	−29.56	5.05	4.82E−09
*DCBLD2*, *MIR548G*	rs6778584	3:98815640	t	0.70	90393	12.98	2.37	4.51E−08
*OTUD4*, *SMAD1*	rs111898810	4:146174040	a	0.20	80752	−20.24	3.61	2.14E−08
*DMRT2*, *SMARCA2*	rs9407640	9:1574877	c	0.41	80754	−14.48	2.65	4.77E−08
*JMJD1C*	rs7899503	10:65087468	c	0.25	90712	21.16	2.84	8.70E−14
*RAB5B*	rs772920	12:56390364	c	0.72	90572	13.86	2.49	2.48E−08
*NCOR2*, *SCARB1*	rs11057793	12:125230287	t	0.75	78930	17.66	3.24	4.78E−08
*SUZ12P1*	rs62070631	17:29087285	a	0.15	82835	20.26	3.64	2.57E−08
*LOC644172*, *CRHR1*	rs186806998	17:43682323	t	0.82	43927	29.50	4.70	3.47E−10
*WNT3*	rs199525	17:44847834	t	0.80	80753	18.85	3.08	9.59E−10
*SOGA2*	rs513953	18:8801351	a	0.29	82871	−14.5	2.58	1.96E−08
*CTAGE1*, *RBBP8*	rs7243351	18:20148531	t	0.45	90708	12.31	2.25	4.69E−08
*ZNF337*	rs6138639	20:25669052	c	0.79	90593	17.91	2.85	3.17E−10
*C20orf112*	rs1737889	20:31042176	t	0.22	80755	−16.82	3.07	4.17E−08

^a^Phenotype: *FEV*_*1*_ forced expiratory volume in 1 s (in ml).

^b^Nearest gene: indicates gene either harboring the variant or nearest to it. *C1orf140/DUSP10* locus also includes *HLX*. *JMJD1C* locus also includes *EGR2*, *NRBF2*, *JMJD1C-AS1*, *REEP3*. *RAB5B* locus also includes *SOUX*. *SMAD3* locus also includes *AAGAB*, *IQCH. MED1/CDK12* locus also includes *FBXL20*. *LOC644172/CRHR1* locus also includes *ARHGAP27*, *MGC57346*, *CRHR1-IT1*, *LRRC37A4P*. *ZNF337* locus also includes *ABHD12*, *PYGB*, *GINS1*, *NINL*, *NANP*, *FAM182B*, *LOC100134868*.

^c^Loci also discovered in European ancestry meta-analyses (Table 2): *C1orf140/DUSP10*, *DMRT2/SMARCA2*, *LOC644172/CRHR1*, *WNT3*.

^d^Alleles for INDELS: I insertion, D deletion.

^e^Additive effect of variant on pulmonary function, adjusting for age, age^2^, sex, height, height^2^, smoking status, pack-years of smoking, weight (for FVC only), and center, ancestral principal components, and a random familial effect to account for family relatedness when appropriate

**Table 5:** Top variants from novel loci discovered in multiethnic meta-analysis of FVC^a^ in the CHARGE consortium

Nearest gene(s)^b,c^	Top variant	Chr:Pos	Coded allele^d^	Allele freq	*N*	Beta^e^	SE	*P* value
*NR5A2*	rs2821332	1:200085714	a	0.47	90,642	14.50	2.51	7.65E−09
*C1orf140*, *DUSP10*	rs12046746	1:221635207	c	0.71	90,427	−16.99	2.81	1.41E−09
*RYR2*	1:237929787:T_TCA	1:237929787	i	0.11	48,215	−37.17	6.79	4.46E−08
*EDAR*	rs17034666	2:109571508	a	0.23	82,747	−27.93	4.96	1.81E−08
*DCBLD2*, *MIR548G*	rs1404098	3:98806782	a	0.71	90,334	15.93	2.73	5.45E−09
*AP3B1*	rs72776440	5:77440196	c	0.21	90,631	−21.30	3.21	3.20E−11
*CENPW*, *RSPO3*	rs11759026	6:126792095	a	0.72	80,687	−20.20	3.44	4.35E−09
*AGMO*	rs55905169	7:15506529	c	0.31	90,511	−17.57	3.09	1.28E−08
*DMRT2*, *SMARCA2*	rs9407640	9:1574877	c	0.42	80,686	−16.82	3.03	2.87E−08
*COMTD1*, *ZNF503-AS1*	10:77002679:TC_T	10:77002679	d	0.22	55,498	22.36	4.10	4.89E−08
*KIRREL3-AS3*, *ETS1*	rs73025192	11:127995904	t	0.12	90,529	−24.18	4.28	1.63E−08
*ALX1*, *RASSF9*	rs7971039	12:85724305	a	0.26	90,639	16.36	2.88	1.44E−08
*CRADD*	rs11107184	12:94184082	t	0.34	88,548	14.89	2.71	3.87E−08
*CCDC41*	rs10859698	12:94852628	a	0.21	88,159	21.19	3.84	3.49E−08
*SQRDL*, *SEMA6D*	rs4775429	15:46722435	t	0.17	79,231	40.23	7.21	2.45E−08
*SMAD3*	rs8025774	15:67483276	t	0.29	88,524	−20.87	2.92	9.34E−13
*PDXDC2P*	rs3973397	16:70040398	a	0.48	44,921	−22.38	4.05	3.31E−08
*PMFBP1*, *ZFHX3*	rs55771535	16:72252097	a	0.13	80,688	−29.88	4.83	6.38E−10
*MED1*, *CDK12*	rs8067511	17:37611352	t	0.80	90,632	18.30	3.20	1.08E−08
*LOC644172*, *CRHR1*	rs150741403	17:43682405	c	0.85	43,896	35.83	5.97	1.94E−09
*WNT3*	rs199525	17:44847834	t	0.80	80,686	20.32	3.52	7.52E−09
*CABLES1*	rs7238093	18:20728158	a	0.22	90,240	18.15	3.13	6.78E−09
*DCC*	rs8089865	18:50957922	a	0.53	90,578	15.81	2.57	7.38E−10

^a^Phenotype: *FVC* forced vital capacity (in ml).

^b^Nearest gene: indicates gene either harboring the variant or nearest to it. *C1orf140/DUSP10* locus also includes *HLX. SMAD3* locus also includes *AAGAB, IQCH. MED1/CDK12* locus also includes *FBXL20*. *LOC644172/CRHR1* locus also includes *ARHGAP27, MGC57346, CRHR1-IT1, LRRC37A4P*.

^c^Loci also discovered in European ancestry meta-analyses (Table 2): *C1orf140/DUSP10, AP3B1, DMRT2/SMARCA2, ALX1/RASSF9, LOC644172/CRHR1, WNT3, DCC*. Loci also discovered in African ancestry meta-analyses (Table 3): *RYR2*.

^d^Alleles for INDELS: I insertion, D deletion

^e^Additive effect of variant on pulmonary function, adjusting for age, age^2^, sex, height, height^2^, smoking status, pack-years of smoking, weight (for FVC only), and center, ancestral principal components, and a random familial effect to account for family relatedness when appropriate

**Table 6:** Top variants from novel loci discovered in multiethnic meta-analysis of FEV_1_/FVC^a^ in the CHARGE consortium

Nearest gene(s)^b,c^	Top variant	Chr:Pos	Coded allele^d^	Allele freq	*N*	Beta^e^	SE	*P* value
*DCAF8*	rs11591179	1:160206067	t	0.45	90,624	−0.002	0.0003	3.48E−08
*KCNJ3, NR4A2*	rs72904209	2:157046432	t	0.88	90,453	0.003	0.0005	3.09E−08
*RBMS3*	rs28723417	3:29431565	a	0.74	90,358	0.002	0.0004	1.77E−08
*DCBLD2*, *MIR548G*	rs80217917	3:99359368	t	0.88	90,617	−0.003	0.0005	2.58E−08
*AFAP1*	rs28520091	4:7846240	t	0.44	80,715	0.002	0.0004	8.40E−09
*LINC00340*	rs9350408	6:22021373	t	0.51	82,761	−0.003	0.0003	7.45E−14
*FLJ35282*, *ELAVL2*	rs10965947	9:23588583	t	0.39	90,475	0.002	0.0004	2.70E−09
*TMEM38B*, *ZNF462*	rs2451951	9:109496630	t	0.47	88,436	0.002	0.0003	2.36E−08
*JMJD1C*	rs75159994	10:64916064	t	0.77	86,988	−0.003	0.0004	6.09E−09
*HTRA1*	rs2293871	10:124273671	t	0.23	90,481	0.002	0.0004	1.51E−08
*FAM168A*	11:73280955: GA_G	11:73280955	d	0.20	55,521	0.004	0.0006	2.74E−08
*DDHD1*, *MIR5580*	rs4444235	14:54410919	t	0.54	80,712	0.002	0.0004	4.03E−08
*TSHZ3*	rs9636166	19:31829613	a	0.86	80,714	0.003	0.0005	3.25E−09
*KLHL22*, *MED15*	rs4820216	22:20854161	t	0.13	82,714	−0.003	0.0005	2.61E−10

^a^Phenotype: ratio FEV_1_/FVC (as a proportion).

^b^Nearest gene: indicates gene either harboring the variant or nearest to it. *HTRA1* locus also includes *DMBT1*. *JMJD1C* locus also includes *EGR2*, *NRBF2*, *JMJD1C-AS1*, *REEP3*. *KLHL22/MED15* locus also includes *ZNF74, SCARF2*.

^c^Loci also discovered in European ancestry meta-analyses (Table 2): *RBMS3*, *AFAP1*, *LINC00340*, *TSHZ3*, *KLHL22/MED15*.

^d^Alleles for INDELS: I insertion, D deletion.

^e^Additive effect of variant on pulmonary function, adjusting for age, age^2^, sex, height, height^2^, smoking status, pack-years of smoking, weight (for FVC only), and center, ancestral principal components, and a random familial effect to account for family relatedness when appropriate

In addition to the fixed-effects multiethnic meta-analysis, we conducted a random-effects meta-analysis using the Han and Eskin method^19^ in METASOFT (http://genetics.cs.ucla.edu/meta/) as a sensitivity analysis. In instances where significant heterogeneity is present, the Han-Eskin method mitigates power loss^19^. In the Han-Eskin random-effects model, 37 of the 47 loci identified in the fixed-effects model at *P* < 5 × 10^−8^ had a *P* value below the same threshold (Supplementary Table 4). Among the ten loci that did not, eight loci still gave a *P* < 5 × 10^−7^ in the Han-Eskin random-effects model (*PIK3C2B*, *SUZ12P1*, *NCOR2/SCARB1*, *CTAGE1/RBBP8*, *C20orf112*, *COMTD1/ZNF503-AS1*, *EDAR*, and *RBMS3*) while only two did not (*CRADD* and *CCDC41*) (Supplementary Table 4). In addition, there were six loci for FEV_1_/FVC that were genome-wide significant in the Han-Eskin random-effects model that had not quite achieved genome-wide significance in the fixed-effects model: G*STO1*/*GSTO2* (chr10, rs10883990), *FRMD4A* (chr10, rs1418884), *ETFA*/*SCAPER* (chr15, rs12440815), *APP* (chr21, rs2830155), *A4GNT* (chr3, rs9864090), *UBASH3B* (chr11, rs4935813) (Supplementary Table 4).

### X-chromosome meta-analysis

Imputed data for X-chromosome variants were available in 12 studies (ARIC, FHS, CHS, MESA, AGES, ALHS, NEO, RS1, RS2, RS3, JHS, Pelotas; *N* = 43,153). Among these studies, fixed-effects inverse-variance weighted meta-analyses were conducted separately in males and females using METAL and the resulting sex-specific results were combined using a weighted sums approach. No X-chromosome variants were associated with PFTs at genome-wide significance in ancestry-specific or multiethnic meta-analyses. Although the absence of associations between X-chromosome variants and PFTs could reflect the reduced sample size, previous GWAS of pulmonary function have only identified one variant^13^.

### Look-up replication of European and multiethnic novel loci

Our primary look-up replication was conducted in the UK BiLEVE study (*N* = 48,943)^14^. Since this study only included individuals of European ancestry, we sought replication only for the 52 novel loci (excluding the major histocompatibility complex, MHC) identified in either the European ancestry or multiethnic discovery meta-analyses. Data for the lead variant was available in the UK BiLEVE study for 51 loci, including 49 loci with a consistent direction of effect between our results and those from UK BiLEVE (Supplementary Table 5). Based on a two-sided *P* < 9.6 × 10^−4^ (0.05/52), 15 loci replicated for the same trait based on the lead variant from our analysis: *DCBLD2/MIR548G*, *SUZ12P1*, *CRHR1*, *WNT3*, *ZNF337*, *ALX1/RASSF9*, *MED1/CDK12*, *EYA2*, *RBMS3*, *LINC00340*, *FLJ35282/ELAVL2*, *DDHD1/MIR5580*, *TSHZ3*, *KLHL22/MED15*, *FAM168A* (Supplementary Table 5). It was recently demonstrated that using one-sided replication *P* values in GWAS, guided by the direction of association in the discovery study, increases replication power while being protective against type 1 error compared to the two-sided *P* values^20^; under this criterion, an additional four loci replicated for the same trait based on the lead variant: *RAB5B*, *JMJD1C*, *AGMO*, and *C20orf112* (Supplementary Table 5).

We also conducted a secondary look-up replication for European ancestry and multiethnic lead variants in the much larger UK Biobank study (*N* = 255,492 with PFTs) from which the UK BiLEVE study is sampled. Unlike the UK BiLEVE results which were adjusted for age, age^2^, sex, height, pack-years of smoking, and ancestral principal components^14^, the publicly available UK BioBank results (https://sites.google.com/broadinstitute.org/ukbbgwasresults/home) are only adjusted for sex and ancestral principal components. In addition, only results for FEV_1_ and FVC (not the ratio FEV_1_/FVC) were currently available. Nevertheless, this secondary look-up yielded evidence of replication for the same trait for an additional 9 loci with a two-sided *P* < 9.6 × 10^−4^: *NR5A2*, *PIK3C2B*, *OTUD4/SMAD1*, *AP3B1*, *CENPW/RSPO3*, *SMAD3*, *PDXDC2P*, *SOGA2*, *DCC* (Supplementary Table 5). Another locus also replicated for the same trait with a one-sided *P* < 9.6 × 10^−4^ (*DNAH12*) and another discovered for FEV_1_/FVC also replicated for FEV_1_ and FVC (*KCNJ3*/*NR4A2*) in the UK Biobank data. In summary, we found evidence of replication in UK BiLEVE or UK Biobank for 30 novel loci.

### Look-up replication of African and Hispanic novel loci

Look-up replication of lead variants for novel African ancestry loci was sought in three smaller studies of African Americans: COPDGene (*N* = 3219)^21,22^, SAPPHIRE (*N* = 1707)^23,24^, and SAGE (*N* = 1405; predominantly children)^25^. We did not find evidence of replication for most of the African ancestry loci identified in our study (Supplementary Table 6). This could possibly reflect low power given the smaller sample sizes of the external studies combined with the low minor allele frequencies (MAF) of most (six out of eight) of the African ancestry variants. We found the strongest evidence for replication for *RYR2* (rs3766889). This variant was common (MAF = 0.18) and well imputed (*r*^2^ > 0.90) in CHARGE. The effect size was similar across CHARGE (*β* = 52.21 ml, *P* = 4.12 × 10^−8^) and the two adult replication studies (COPDGene *β* = 46.85 ml, *P* = 0.03 and SAPPHIRE *β* = 22.00 ml, *P* = 0.32); meta-analysis of these adult studies resulted in a significant combined association (*β* = 47.35 ml, SE = 8.00 ml, *P* = 3.30 × 10^−9^). In SAGE, which includes mostly children and examined percent predicted values, the result was in the opposite direction and not significant. In our Hispanic ethnicity/Latino meta-analysis, the lead variant from the single novel locus (rs6746679, *DKFZp686O1327*/*PABPC1P2*) did not replicate in two smaller external studies of Hispanics: MESA (*N* = 806; MESA Hispanics not included in discovery) and GALA II (*N* = 2203; predominantly children)^26^ (Supplementary Table 6).

### Overlap of newly identified loci with COPD

Pulmonary function measures are the basis for the diagnosis of COPD, an important clinical outcome; therefore, we also looked-up the 52 novel loci identified in the European ancestry or multiethnic meta-analyses in the International COPD Genetics Consortium (ICGC). This consortium recently published a meta-analysis of 1000 Genomes imputed variants and COPD primarily among individuals of European ancestry (*N* = 15,256 cases and 47,936 controls), including some of the same individuals included in the present lung function analysis^27^. Ten lead variants representing eight novel loci were associated with COPD at *P* < 9.6 × 10^−4^: *RBMS3*, *OTUD4/SMAD1*, *TMEM38B/ZNF462*, *NCOR2/SCARB1*, *SUZ12P1*, *WNT3*, *SOGA2*, *C20orf112* (Supplementary Table 7). Directions of effects were consistent between our results and the COPD findings for these variants (i.e., variants associated with increased pulmonary function values were associated with decreased odds of COPD and vice-versa). Our top variant in *SOGA2* (also known as *MTCL1*) is in LD (*R*^2^ = 0.8) with the top variant for COPD as reported by the IGCG Consortium^27^.

### eQTL and mQTL signals

To query whether novel loci contained variants associated with gene expression (eQTLs), we looked-up variants from all 60 novel loci identified in any ancestry-specific or multiethnic meta-analyses in the following datasets: (1) lung samples from 278 individuals in genotype-tissue expression (GTEx) (https://www.gtexportal.org/home/)^28^; (2) lung samples from 1111 participants in studies from the Lung eQTL Consortium including Laval University, the University of Groningen and the University of British Columbia^29–31^; (3) whole blood samples from 5257 Framingham Heart Study participants^32^; (4) peripheral blood samples from 5311 participants in EGCUT, InCHIANTI, Rotterdam Study, Fehrmann, HVH, SHIP-TREND and DILGOM^33^; and (5) peripheral blood samples from 2116 participants in four Dutch studies collectively known as BIOS^34,35^. We examined both whole blood and lung datasets to take advantage of the much larger size, and higher statistical power, of available blood eQTL datasets since we have previously found substantial overlap between lung and blood eQTLs for lung function GWAS loci, as well as complementary information from these two different tissues^29^. The Lung eQTL Consortium study used a 10% FDR cut-off, while all other studies used a 5% FDR cutoff (see Supplementary Note 1 for further study descriptions and methods).

A significant cis-eQTL in at least one dataset was found for 34 lead variants representing 27 novel loci (Supplementary Table 8). Of these, 13 loci had significant cis-eQTLs only in datasets with blood samples and three loci only in datasets with lung samples (*COMTD1/ZNF503-AS1*, *FAM168A*, *SOGA2*). Eleven loci had significant cis-eQTLs in both blood and lung samples based on lead variants, with one locus having a significant cis-eQTL across all five datasets (*SMAD3*) and another four loci having a significant cis-eQTL in four datasets (*RAB5B*, *CRHR1*, *WNT3*, *ZNF337*). Significant trans-eQTLs in at least one dataset were found for seven lead variants representing four novel loci (*TMEM38B/ZNF462*, *RAB5B, CRHR1*, and *WNT3*, Supplementary Table 8).

In addition, mQTL data were available from FHS and BIOS. Significant cis-mQTLs and trans-mQTLs in at least one dataset were found for 52 lead variants (43 novel loci) and 1 lead variant (1 novel locus), respectively (Supplementary Table 8).

None of the novel variants discovered for African and Hispanic ancestry indicated significant cis-eQTLs in GTex which includes some slight multiethnic representation (12% African American and 3% other races/ethnicities). Although few ancestry-specific eQTL datasets exist, we identified two such studies with blood samples from African American participants: SAPPHIRE (*N* = 597) and MESA (*N* = 233)^36^. In SAPPHIRE, none of the eight African ancestry variants identified in the meta-analysis indicated significant cis-eQTLs (FDR < 0.05), but rs180930492 was associated with a trans-eQTL among individuals without asthma and rs111793843 and rs139215025 were associated with trans-eQTLs among individuals with asthma at FDR < 0.05 (Supplementary Table 9). In MESA, eQTL data were available for only three of the African ancestry variants (rs11748173, rs3766889, rs144296676), and none indicated significant (FDR < 0.05) cis-eQTLs (Supplementary Table 9).

### Heritability and genetic correlation

We used LD score regression^37^ to estimate the variance explained by genetic variants investigated in our European ancestry meta-analysis, also known as single nucleotide polymorphisms (SNP) heritability. Across the genome, the SNP heritability (narrow-sense) was estimated to be 20.7% (SE 1.5%) for FEV_1_, 19.9% (SE 1.4%) for FVC, and 17.5% (SE 1.4%) for FEV_1_/FVC.

We also partitioned heritability by functional categories to investigate whether particular subsets of common variants were enriched^38^. We found significant enrichment in six functional categories for all three PFTs: conserved regions in mammals, DNase I hypersensitive sites (DHS), superenhancers, the histone methylation mark H3K4me1 and histone acetylation marks H3K9Ac and H3K27Ac (Supplementary Figure 4). Another seven categories showed enrichment for at least one PFT (Supplementary Figure 5). We observed the largest enrichment of heritability (14.5–15.3 fold) for conserved regions in mammals, which has ranked highest in previous partitioned heritability analyses for other GWAS traits (Supplementary Figure 5)^38^.

Since both height and smoking are important determinants of pulmonary function, and have been associated with many common variants in previous GWAS, we also used LD score regression to investigate genetic overlap^39^ between our FEV_1_, FVC, and FEV_1_/FVC results and publicly available GWAS results of ever smoking^40^ and height^41^. No significant genetic correlation was found between PFTs and smoking or height (Supplementary Table 10), indicating our findings are independent of those traits.

In addition, we used LD Score regression to investigate genetic overlap between each PFT and the other two PFTs, as well as with asthma. Based on the overall PFT results presented in this paper, we found significant genetic correlation between FEV_1_ and FVC (*P* < 0.001) and between FEV_1_ and FEV_1_/FVC (*P* < 0.001), but not between FVC and FEV_1_/FVC (*P* = 0.23; Supplementary Table 10). Since measures of FEV_1_ and FVC (independent of genetics) are highly correlated, and to lesser degree FEV1/FVC^10^, these results are not surprising. Using publicly available GWAS results for asthma^42^, we also found significant correlation between PFTs and asthma (*P* < 0.003; Supplementary Table 10).

### Functional annotation

For functional annotation, we considered all novel variants with *P* < 5 × 10^−8^ from the 60 loci discovered in our ancestry-specific and multiethnic meta-analyses, plus significant variants from the MHC region, two loci previously discovered in the CHARGE exome chip study (*LY86*/*RREB1* and *SEC24C*)^43^ and *DDX1*. Using Ensembl variant effect predictor (VEP)^44^, we found six missense variants in four loci outside of the MHC region and 22 missense variants in the MHC region (Supplementary Table 11). Of the 28 total missense variants, two (chr15:67528374 in *AAGAB* and chr6:30899524 in the MHC region) appear to be possibly damaging based on sorting intolerant from tolerant (SIFT)^45^ and Polymorphism Phenotyping v2 (PolyPhen-2)^46^ scores (Supplementary Table 11). Using combined annotation dependent depletion (CADD)^47^, we found an additional 28 deleterious variants from 15 loci based on having a scaled *C*-score greater than 15 (Supplementary Data 1). In the MHC region, we found another 11 deleterious variants based on CADD. Based on RegulomeDB^48^, which annotates regulatory elements especially for noncoding regions, there were 52 variants from 18 loci with predicted regulatory functions (Supplementary Data 1). In the MHC region, there were an additional 72 variants with predicted regulatory functions.

### Pathway enrichment analysis

Gene set enrichment analyses conducted using data-driven expression prioritized integration for complex traits (DEPICT)^49^ on genes annotated to variants with *P* < 1 × 10^−5^ based on the European ancestry meta-analysis results revealed 218 significantly enriched pathways (FDR < 0.05) (Supplementary Data 2). The enriched pathways were dominated by fundamental developmental processes, including many involved in morphogenesis of the heart, vasculature, and lung. Tissue and cell type analysis noted significant enrichment (FDR < 0.05) of smooth muscle, an important component of the lung (Supplementary Table 12, Supplementary Figure 6). We found 8, 1, and 82 significantly prioritized genes (FDR < 0.05) for FEV_1_, FVC, and FEV_1_/FVC, respectively (Supplementary Data 3). Given the generally smaller p-values for variants associated with FEV_1_/FVC, enriched pathways and tissue/cell types as well as prioritized genes were predominantly discovered from DEPICT analyses of FEV_1_/FVC.

Due to extended LD across the MHC locus on chromosome 6 (positions 25000000 to 35000000), DEPICT excludes this region^49^. Standard Ingenuity Pathway Analysis (IPA) run without excluding the MHC highlighted 16 enriched networks based on the European ancestry meta-analysis results, including three involved in inflammatory diseases or immunity; 33 of the 84 genes in these 3 networks are in the MHC region (Supplementary Table 13). IPA based on the multiethnic results highlighted 21 enriched networks, including similar inflammatory and immunity related networks (Supplementary Table 14).

### Identification of potential causal variants using PAINTOR

Using a multiethnic fine-mapping analysis incorporating strength of association, variation in genetic background across major ethnic groups, and functional annotations in Probabilistic Annotation INtegraTOR (PAINTOR)^50^, we examined 38 loci that contained at least five genome-wide significant variants in the European ancestry and multiethnic meta-analyses or at least one significant variant in the African ancestry or Hispanic/Latino ethnicity meta-analyses. We identified 15 variants representing 13 loci as having high posterior probabilities of causality (>0.8): 3 for FEV_1_, 3 for FVC, and 9 for FEV_1_/FVC (Supplementary Table 15, Supplementary Figure 7). Of the 15 putative casual variants, 11 showed high posterior probabilities of causality (>0.8) before considering annotations, and 4 were identified by adding functional annotations. Nine were the top SNPs at that locus from the fixed-effects meta-analysis (loci: *WNT3*, *PMFBP1/ZFHX3*, *EN1/MARCO*, *C2orf48/HPCAL1*, *CPT1C*, *CADPS*, *LOC283867/CDH5*, *HDC*, and *CDC7/TGFBR3*), while 6 were not (loci: *CDK2/RAB5B*, *BMS1P4*, *PMFBP1/ZFHX3*, *FLJ35282/ELAVL2*, *HDC*, and *COL8A1*).

### Identification of independent signals using FINEMAP

We used FINEMAP^51^ to identify variants with a high posterior probability of causality (>0.6) independent of 118 lead variants in pulmonary function loci identified in the current or previous studies^14^. We identified 37 independent variants for 23 previously identified loci and one independent variant at each of two novel loci (*LINC00340* and *SLC25A51P1/BAI3*; Supplementary Table 16).

### Gene-based analysis of GWAS results using S-PrediXcan

Among the novel loci identified in the current GWAS of PFTs, we identified seven variants corresponding to nine genes demonstrating genome-wide significant evidence of association with lung or whole blood tissue-specific expression (Supplementary Table 17) based on the gene-based S-PrediXcan approach^52^. Bayesian colocalization analysis^53^ indicated the following associations demonstrated at least 50% probability of shared SNPs underlying both gene expression and PFTs: *ARHGEF17* and *FAM168A* in analysis of multiethnic GWAS for FEV_1_/FVC based on GTEx whole blood models, and *WNT3* in analysis of multiethnic GWAS for FVC based on GTEx lung models (Supplementary Table 18).

### Druggable targets

To investigate whether the genes identified in our study encode proteins with predicted drug targets, we queried the ChEMBL database (https://www.ebi.ac.uk/chembl/). In addition, we incorporated an IPA to identify potential upstream targets. Genes associated with pulmonary function, but not included in the drug target analysis performed by Wain et al.^14^, were evaluated, for a total of 139 genes outside of the MHC: 110 genes representing the 60 novel loci identified in our fixed-effects ancestry-specific and multiethnic meta-analysis, 13 genes representing the 6 novel loci identified in our random-effects meta-analysis^19^, 3 genes representing an additional 3 loci near significance in the African ancestry meta-analysis (*BAZ2B*, *NONE*/*PCDH10*, and *ADAMTS17*), 9 genes representing 2 loci identified in a recent CHARGE analysis of exome variants^43^, which were also significant in our 1000 Genomes analysis (*LY86*/*RREB1* and *SEC24C*), and 4 genes representing one locus identified at genome-wide significance in a separate publication from one of our participating studies (HCHS/SOL)^18^, but also significant in our analysis (*ADORA2B*/*ZSWIM7*/*TTC19*/*NCOR1*). In the ChEMBL database, 17 of these genes encode proteins with predicted or known drug targets: *NR5A2*, *KCNK2*, *EDAR*, *KCNJ3*, *NR4A2*, *BAZ2B*, *A4GNT*, *GSTO1*, *GSTO2*, *NCOR2*, *SMAD3*, *NCOR1*, *CDK12*, *WNT3*, *PYGB*, *NANP*, *EYA2* (Supplementary Table 19). Of these, two genes (*KCNK2* and *CDK12)* have approved drug targets. Using IPA, four additional genes were predicted as drug targets (*ADORA2B*, *APP*, *CRHR1*, and *MAP3K1*; Supplementary Table 20) and 37 genes had drugs or chemicals as upstream regulators (Supplementary Table 21).

## Discussion

By conducting a GWAS meta-analysis in a large multiethnic population we increased the number of known loci associated with pulmonary function by over 50%. In total, we identified 60 novel genetic regions (outside of the MHC region): 17 from European ancestry, 8 from African ancestry, 1 from Hispanic/Latino ethnicity, and 34 from multiethnic meta-analyses.

For 32 of the 52 loci novel loci identified in our European ancestry and multiethnic meta-analyses, we found evidence for look-up replication in the UK BiLEVE study, UK Biobank study, or ICGC COPD consortium. For an additional three loci, we found support for validation using new genomic methods such as PAINTOR, FINEMAP, or S-PrediXcan. Specifically, 19 novel variants replicated in look-up in a smaller independent sample of Europeans from the UK BiLEVE study^14^: *DCBLD2*/*MIR548G*, *SUZ12P1*, *CRHR1*, *WNT3*, *ZNF337*, *ALX1/RASSF9*, *MED1/CDK12*, *EYA2*, *RBMS3*, *LINC00340*, *FLJ35282/ELAVL2*, *DDHD1/MIR5580*, *TSHZ3*, *KLHL22/MED15*, *FAM168A*, *RAB5B*, *JMJD1C*, *AGMO*, and *C20orf112*. Based on a minimally adjusted publicly available analysis in a larger sample of Europeans from the UK Biobank, an additional 11 loci replicated: *NR5A2*, *PIK3C2B*, *OTUD4/SMAD1*, *AP3B1*, *CENPW/RSPO3*, *SMAD3*, *PDXDC2P*, *SOGA2*, *DCC*, *DNAH12*, and *KCNJ3/NR4A2*. Because UK BiLEVE is sampled from UK Biobank we are not able to perform a combined replication meta-analysis. Additionally, the studies adjusted for different covariates (UK BiLEVE results were adjusted for age, sex, height, pack-years and ancestral principal components while UK Biobank results were adjusted for only sex and ancestral components). Among those loci which did not directly replicate for PFTs in the UK BiLEVE or UK Biobank datasets, the lead variants in an additional two European or multiethnic loci were significantly associated in the ICGC Consortium with COPD, which was defined using PFT measures^27^: *TMEM38B*/*ZNF462* and *NCOR2*/*SCARB1*. FINEMAP and S-PrediXcan also produced evidence for causality for three European ancestry and multiethnic loci which had not replicated in UK BiLEVE, UK Biobank or ICGC: *DCAF8*, *AFAP1*, and *SLC25A51P1/BAI3*.

The few additional studies with 1000 Genomes imputed variants and pulmonary function in African ancestry individuals have smaller samples sizes making replication challenging for the eight novel loci identified in our African ancestry meta-analyses. Further, lead variants for six of the eight loci were low frequency in African Ancestry (*C2orf48/HPCAL1*, *EN1/MARCO*, *CADPS*, *HDC*, *LOC283867/CDH5*, and *CPT1C*) (MAF < 0.02), including three not well imputed (*r*^2^ < 0.75), and monomorphic or nearly monomorphic in other ancestries (European, Asian, and Hispanic). For the two novel African ancestry variants with MAF > 0.02 and well imputed (*r*^2^ > 0.90), we found the strongest evidence for replication for *RYR2* (rs3766889). This variant had a similar effect estimate for FEV_1_ in CHARGE, COPDGene, and SAPPHIRE with a significant combined association across these adult studies. Although this particular variant did not quite meet genome-wide significance in the multiethnic meta-analysis for FEV_1_ (*P* = 6.56 × 10^−4^), another variant in this gene did for FVC (1:237929787:T_TCA, *P* = 4.46 × 10^−8^).

Our analysis also sheds light on additional potential causal genes at a complex locus (chromosome 17 near positions 43600000 to 44300000, hg19) previously discovered from GWAS of FEV_1_, which identified *KANSL1* in European populations as the top finding for this region^14,15^. With the exception of a single INDEL in *KANSL1* in our European ancestry meta-analysis (17:44173680:T_TC, *P* = 1.03 ×  10^−10^), we found *CRHR1* as the strongest gene associated with FEV_1_ in this region. Although some variants in *CRHR1* identified in our study are within 500kb of *KANSL1* (e.g., rs16940672, 17:43908152, *P* = 2.07 × 10^−10^), a number of significant variants in this gene are more than 500 kb away from previously identified hits [our definition of novel] (e.g., rs143246821, 17:43685698, *P* = 9.06 × 10^−10^). In our multiethnic meta-analysis, several variants in *CRHR1* were associated with FEV_1_ at smaller *P* values than variants in *KANSL1*. Definitive assessment of the causal variants at this locus, as well as other multigenic GWAS loci, will likely require additional data from ongoing large-scale sequencing studies to enable detailed fine mapping.

In both our European and multiethnic meta-analyses we also noted a significant association with *WNT3* on chromosome 17 near position 44800000 (hg19) which is more than 500kb from *KANSL1* or *CRHR1* [our definition of novel]. We found that the top variant in *WNT3* for FEV_1_ among individuals of European ancestry (rs916888, 17:44863133, *P* = 3.76 × 10^−9^) had a high probability for causality based on PAINTOR, an analysis which integrates functional annotations along with association statistics and LD for each ethnicity^50^. We also found evidence that *WNT3* may be the causal gene at this locus using S-PrediXcan, a gene level association test that prioritizes potentially causal genes while filtering out LD-induced false-positives^52,53^. Notably, S-PrediXcan implicated *WNT3* as a likely mediating gene for FVC based on the top variant in our multiethnic meta-analyses (rs199525, 17:44847834, *P* = 7.52 × 10^−9^), which is an eQTL SNP for *WNT3* in lung and other tissues. Further, the lead *WNT3* variants for both FEV_1_ and FVC (rs916888 and rs199525) were significantly associated with COPD in a look-up of a large published meta-analysis dataset^27^. In addition, other genes in the *WNT* signaling pathway, a fundamental development pathway, have been implicated as influencing pulmonary function^54^. This pathway was also one of the significant pathways identified in our analysis. In a previous pathway analysis of asthma, *SMAD3* has been shown to interact with the *WNT* signaling pathway^55^. Finally, *WNT3* also emerged as having a potential druggable target, and incorporation of pathway analysis to identify upstream regulators found an additional four drugs in clinical use for which *WNT3* is a target molecule (chemotherapeutic agents doxorubicin and paclitaxel, the hormone beta-estradiol and LGK-974, a novel agent that targets a WNT-specific acyltransferase)^56^. Again, further evaluation of this interesting and complex locus which contains many significant variants in LD will benefit from data being generated in ongoing large-scale sequencing studies.

Some genes identified in our study play key roles in inflammation, immunity, and pulmonary biology. For example, *MARCO* (macrophage receptor with collagenous structure) has been shown in murine models to be required for lung defense against pneumonia and inhaled particles^57,58^. *SMAD3* is part of the SMAD family of proteins which are signal transducers and transcriptional modulators that mediate multiple signaling pathways. *SMAD3* is activated by transforming growth factor beta (TGF-B) which plays a key role in airway remodeling. S*MAD3* has a predicted drug target and SNPs in *SMAD3* are significantly associated with asthma in GWAS^42,59^.

Other genes identified in our study that are targeted by approved drugs include *CDK12* and *KCNK2*. *CDK12* drug targets include AT-7519, Roniciclib, AZD-5438, and PH.A-793887. Roniciclib has been used in clinical trials including lung cancer patients^60^. *KCNK2* (potassium channel subfamily K member 2) is targeted by five inhalational anesthetic agents. These agents have antiinflammatory effects both systemically^61^ and in the lungs^62^ and meta-analysis of clinical studies shows protection against pulmonary complications after cardiac surgery^63^. A recent trial suggested that one of these inhalation agents, sevoflurane, offers promise for reducing epithelial injury and improving outcomes in patients with acute respiratory distress syndrome^64^.

In addition to querying commonly used genome databases for functional annotation of variants, we sought to narrow down causal variants in implicated loci using recently developed methods that incorporate LD, functional data and/or the multiethnic analysis done in this paper. In particular, PAINTOR is a useful tool to identify potential causal variants in our novel loci as it leverages LD across ancestral groups along with association statistics and functional annotations^50^. PAINTOR identified 15 putative causal variants from 13 loci, including seven loci uniquely identified in the multiethnic meta-analyses such as *PMFBP1*/*ZFHX3* and *COL8A1* (part of the *DCBLD2* loci). Several of the putative causal variants from PAINTOR were the top SNPs from the fixed-effects meta-analysis (e.g., rs916888 *WNT3*). Similarly, FINEMAP has been shown to be an accurate and efficient tool for investigating whether lead SNPs for a given loci are driven by independent variants in the same region, especially when annotation information is not available^51^. Among previous and novel loci identified in individuals of European ancestry, we identified 37 independent variants for 23 previously identified loci and two lead variants for two novel loci (rs1928168 *LINC00340* and rs9351637 *SLC25A51P1*/*BAI3)* with a high probability of causality. Finally, we ran S-PrediXcan a gene level association test that prioritizes potentially causal genes^52^. Seven of our novel loci contained putative causal genes based on S-PrediXcan for lung or whole blood tissues, including *NRBF2* (part of the *JMJD1C* locus) and *WNT3*. S-PrediXcan also highlighted the region around chromosome 11 position 73280000 (hg19), noting strong evidence for both *FAM168A* and *ARHGEF17* which was further supported by the colocalization analysis. Interestingly, DEPICT also prioritized *ARHGEF17*, a member of the guanine nucleotide exchange factor (GEF) family of genes which can mediate actin polymerization and contractile sensitization in airway smooth muscle^65,66^.

Rather than conducting a standard gene-based pathway analysis, we performed a newer integrative method, DEPICT, that incorporates cell and tissue-specific functional data into a pathway analysis to prioritize genes within implicated loci^49^. In addition to identifying potential causal variants, this approach revealed a number of fundamental development processes, including pathways related to lung development, growth regulation, and organ morphogenesis. The *WNT* signaling pathway was also highlighted along with processes relevant to the pathogenesis of COPD including extracellular matrix structure and collagen networks. Tissue/cell type enrichment results highlighted smooth muscle which is highly relevant for lung function. DEPICT excludes the MHC due to extended LD in this region, which likely explains the relative paucity of inflammation-related pathways identified compared to previous pathway analyses in GWAS of PFTs^29,54^. Indeed, standard IPA analysis of our data including the MHC region, found that 33 of 84 genes (39%) in the 3 (out of 16) enriched networks involved in immune or inflammatory processes are in the MHC. The predominance of fundamental pathways related to lung growth, differentiation and structure is consistent with recent work^67^ that has rekindled interest in the observation made 40 years ago^68^ that individuals can cross the threshold for diagnosis of COPD either by rapid decline in adulthood or by starting from a lower baseline of maximal pulmonary function attained during growth. Within this context, understanding the genetic (and environmental) factors that influence the variability in maximal lung function attained during the first three decades of life is essential to reducing the public health burden of COPD^69^.

In summary, our study extends existing knowledge of the genetic landscape of PFTs by utilizing the more comprehensive 1000 Genomes imputed variants, increasing the sample size, including multiple ancestries and ethnicities, and employing newly developed computational applications to interrogate implicated loci. We discovered 60 novel loci associated with pulmonary function and found evidence of replication in UK BiLEVE, UK Biobank, or ICGC for 32 novel loci and validation for another 3 loci. We found evidence that several variants in these loci were missense mutations and had possible deleterious or regulatory effects, and many had significant eQTLs. Further, using new genomic methods that incorporate LD, functional data and the multiethnic structure of our data, we shed light on potential causal genes and variants in implicated loci. Finally, several of the newly identified genes linked to lung function are druggable targets, highlighting the clinical relevance of our integrative genomics approach.

## Methods

### Studies

Member and affiliate studies from The CHARGE consortium with pulmonary function and 1000 Genomes imputed genetic data were invited to participate in the present meta-analysis. Participating studies included: AGES, ALHS, ARIC, CARDIA, CHS, FamHS, FHS, GOYA, HCHS/SOL, HCS, Health ABC, Healthy Twin, JHS, KARE3, LifeLines, LLFS, MESA, NEO, 1982 PELOTAS, RSI, RSII, RIII. Characteristics of these studies are provided in Supplementary Table 22 and descriptions of study designs are provided in the Supplementary Note 1; informed consent was obtained from participants in each study. Although our meta-analysis included studies of asthma (ALHS) and obesity (GOYA and NEO), exclusion of these studies did not materially change results (Supplementary Note 2). Further, previous meta-analyses of GWAS of pulmonary function have demonstrated high correlation between results when including or excluding asthma and COPD cases^8^.

### Pulmonary function

Spirometry measures of pulmonary function (FEV_1_, FVC, and the ratio FEV_1_/FVC) were collected by trained staff in each study according to American Thoracic Society or European Respiratory Society guidelines. See cohort descriptions in Supplementary Note 1 for more details.

### Variants

Studies used various genotyping platforms, including Affymetrix Human Array 6.0, Illumina Human Omni Chip 2.5, and others, as described in cohort descriptions in the Supplementary Note 1. Using MACH, MINIMAC, or IMPUTE2, studies then used genotyped data to impute variants based on the 1000 Genomes Integrated phase 1 reference panel. One study (Hunter Community) imputed to the 1000 Genomes European phase 1 reference panel; sensitivity analyses excluding this study from the European ancestry meta-analysis showed no material differences (see Supplementary Note 2). The two Asian studies (Healthy Twin and KARE3) imputed to the 1000 Genomes Asian phase 1 reference panel.

### Statistical analysis

Within each study, linear regression was used to model the additive effect of variants on PFTs. FEV_1_ and FVC were modeled as milliliters and FEV_1_/FVC as a proportion. Studies were asked to adjust analyses for age, age^2^, sex, height, height^2^, smoking status (never, former, and current), pack-years of smoking, center (if multicenter study), and ancestral principal components, including a random familial effect to account for family relatedness when appropriate^70^. Models of FVC were additionally adjusted for weight. Analyses were conducted using ProbAbel, PLINK, FAST, or the R kinship package as described in the cohort descriptions of the Supplementary Note 1.

Ancestry-specific and multiethnic fixed-effects meta-analyses using inverse variance weighting of study-specific results with genomic control correction were conducted in Meta-Analysis Helper (METAL, http://www.sph.umich.edu/csg/abecasis/metal/). Multiethnic random-effects meta-analyses using the four ancestry-specific fixed-effects meta-analysis results were conducted using the Han-Eskin model^19^ in METASOFT (http://genetics.cs.ucla.edu/meta/). Only variants with p-values for association <0.05 or *P* values for heterogeneity <0.1 from fixed-effects models were included in the random-effects models.

Variants with imputation quality scores (*r*^2^) less than 0.3 and/or a minor allele count (MAC) less than 20 were excluded from each study prior to meta-analysis. Following meta-analysis, we also excluded variants with less than one-third the total sample size or less than the sample size of the largest study for a given meta-analysis to achieve the following minimal sample sizes: 20,184 for European ancestry; 2810 for African ancestry; 7862 for Asian ancestry; 4435 for Hispanic/Latino ethnicity; and 30,238 for Multiethnic.

Significance was defined as *P* < 5 × 10^−8^^14,17^. Novel variants were defined as being more than ±500 kb from the top variant of a loci identified in a previous GWAS of pulmonary function; previous multiethnic GWAS have used this definition^16^. We used the list of 97 known variants as published in the recent UK BiLEVE paper^14^ with the following modifications: added variants in *DDX1*, *DNER*, *CHRNA5* since listed in GWAS catalog; added variants in *LCT*, *FGF10*, *LY86/RREB1*, *SEC24C*, *RPAP1*, *CASC17*, and *UQCC1* since identified in exome chip paper^43^; added variant in *TMEM163* identified in Loth et al. paper^10^; used 17:44339473 instead of 17:44192590 to represent *KANSL1* since 17:44339473 was the original variant listed for *KANSL1* in Wain et al.^15^; and used 12:28283187 instead of 12:28689514 to represent *PTHLH* since 12:28283187 was the original variants listed for *PTHLH* in Soler Artigas et al.^13^.

Genomic inflation factors (lambda values) from quantile–quantile plots of observed and expected *P* values for ancestry- and phenotype-specific meta-analyses are presented in Supplementary Table 23. Lambda values were slightly higher in European and multiethnic meta-analyses (range of lambda 1.12–1.16) than in other ancestry-specific meta-analyses (range of lambda 1.01–1.06) likely due to the much larger sample sizes of the European and multiethnic meta-analyses^71^.

### LD score regression

The SNP heritability, i.e., the variance explained by genetic variants, was calculated from the European ancestry GWAS summary statistics (with genomic control off) using LD score regression (https://github.com/bulik/ldsc)^37^. Partitioned heritability was also calculated using the method described by Finucane et al.^38^. In total, 28 functional annotation classes were used for this analysis, including coding regions, regions conserved in mammals, CCCTC-binding factor, DNase genomic foot printing, DHS, fetal DHS, enhancer regions; including superenhancers and active enhancers from the FANTOM5 panel of samples, histone marks including two versions of acetylation of histone H3 at lysine 27 (H3K27ac and H3K27ac2), histone marks monomethylation (H3K4me1), trimethylation of histone H3 at lysine 4 (H3K4me), and acetylation of histone H3 at lysine 9 (H3K9ac5). In addition to promotor and intronic regions, transcription factor binding site, transcription start site, and untranslated regions (UTR3 and UTR5). A *P* value of 0.05/28 classes <1.79 × 10^−3^ was considered statistically significant. Genetic correlation between our pulmonary function (FEV_1_, FVC and FEV_1_/FVC) results and publicly available GWAS of ever smoking^40^ and height^41^ was also investigated using LD score regression^39^.

### Functional annotation

To find functional elements in novel genome-wide significant signals, we annotated SNPs using various databases. We used Ensembl VEP^44^ (Accessed 17 Jan 2017) and obtained mapped genes, transcripts, consequence of variants on protein sequence, SIFT^45^ scores, and PolyPhen-2^46^ scores. We checked if there were deleterious variants using CADD v1.3^47^, which integrates multiple annotations, compares each variant with possible substitutions across the human genome, ranks variants, and generates raw and scaled *C*-scores. A variant having a scaled *C*-score of 10 or 20 indicates that it is predicted to be in the top 10% or 1% deleterious changes in human genome, respectively. We used a cutoff of 15 to provide deleterious variants since it is the median for all possible splice site changes and nonsynonymous variants (http://cadd.gs.washington.edu/info, Accessed 18 Jan 2017). To find potential regulatory variants, we used RegulomeDB^48^ (Accessed 17 Jan 2017), which integrates DNA features and regulatory information including DNAase hypersensitivity, transcription factor binding sites, promoter regions, chromatin states, eQTLs, and methylation signals based on multiple high-throughput datasets and assign a category to each variant. Variants having RegulomeDB categories 1 or 2, meaning “likely to affect binding and linked to expression of a gene target” or “likely to affect binding,” were considered as regulatory variants.

### Pathway analysis using DEPICT and IPA

For gene prioritization and identification of enriched pathways and tissues/cell types, we used DEPICT^49^ with association results for FEV_1_, FVC, and FEV_1_/FVC. We used association results from our European ancestry meta-analysis and the LD structure from 1000 Genomes European (CEU, GBR, and TSI) reference panel. The software excludes the MHC region on chromosome 6 due to extended LD structure in the region. We ran a version of DEPICT for 1000 Genomes imputed meta-analysis results using its default parameters with an input file containing chromosomal location and *P* values for variants having unadjusted *P* < 1 × 10^−5^. For gene set enrichment analyses, DEPICT utilizes 14,461 reconstituted gene sets generated by genes’ coregulation patterns in 77,840 gene expression microarray data. For tissue/cell type enrichment analysis, mapped genes were tested if they are highly expressed in 209 medical subject headings annotations using 37,427 microarray data. Gene prioritization analysis using cofunctionality of genes can provide candidate causal genes in associated loci even if the loci are poorly studied or a gene is not the closest gene to a genome-wide significant variant. We chose FDR < 0.05 as a cutoff for statistical significance in these enrichment analyses and gene prioritization results. Because DEPICT excludes the MHC, we also ran a pathway analysis with IPA (Ingenuity Systems, Redwood City, CA, USA, http://www.ingenuity.com/) on genes to which variants with *P* < 1 × 10^−5^ annotated.

### PAINTOR

To identify causal variants in novel genome-wide significant loci, we used a transethnic functional fine mapping method^50^ implemented in PAINTOR (https://github.com/gkichaev/PAINTOR_FineMapping, Accessed 2 May 2016). This method utilizes functional annotations along with association statistics (*Z*-scores) and LD information for each locus for each ancestry. We included our ancestry-specific meta-analysis results and used the African, American, European, and East Asian individuals from 1000 Genomes to calculate LD^72^. For PAINTOR we focused on 22 novel loci identified in our European ancestry and multiethnic fixed-effects meta-analyses which had at least five genome-wide significant variants as well as all nine African or Hispanic loci which had at least one genome-wide significant variant. In addition, we included six loci which overlapped with the UK BiLEVE 1000 Genomes paper^14^ and one locus with the CHARGE exome paper^43^, since we ran PAINTOR prior to those publications. To reduce computational burden, we limited flanking regions to ±100 kilobase (kb) from the top SNPs and included variants with absolute value of *Z*-score greater than 1.96.

We used 269 publicly available annotations relevant to “lung”, “bronch”, or “pulmo” from the following: hypersensitivity sites^73^, superenhancers^74^, Fantom5 enhancer and transcription start site regions^75^, immune cell enhancers^76^, and methylation and acetylation marks ENCODE^77^. We ran PAINTOR for each phenotype separately to prioritize annotations based on likelihood-ratio statistics^78,79^. We included minimally correlated top annotations (less than five for each phenotype) to identify causal variants.

For the 38 loci from the fixed-effects meta-analysis, we used PAINTOR to construct credible sets of causal variants using a Bayesian meta-analysis framework. To obtain 95% credible sets for each locus, we ranked SNPs based on posterior probabilities of causality (high to low) and then took the SNPs filling in 95% of the summed posterior probability. We computed the median number of SNPs in the credible sets for ancestry-specific and multiethnic analyses of each trait.

### FINEMAP

We used FINEMAP^51^ to identify signals independent of lead variants for pulmonary function loci identified in the current or previous studies^14^. The Rotterdam Study (*N* = 6291), one of the larger cohort studies included in our meta-analysis, was used as a reference population. SNPs with MAF of <1% were excluded, leaving 118 SNPs for analysis. Ten SNPs for FEV_1_ and FVC and 20 SNPs for FEV_1_/FVC were further excluded because the LD matrix of the reference file from the Rotterdam Study did not represent the correlation matrix of the total study population. We allowed up to 10 causal SNPs per loci in FINEMAP analyses. To reduce the chance of false positive findings, we also conducted sensitivity analyses allowing up to 15 causal SNPs for loci with more than four SNPs with posterior probabilities of >0.8.

### S-PrediXcan

S-PrediXcan is a summary statistics based approach for gene-based analysis^52^ that was derived as an extension of the PrediXcan method for integration of GWAS and reference transcriptome data^80^. We used the S-PrediXcan approach to prioritize potentially causal genes, coupled with a Bayesian colocalization procedure^53^ used to filter out LD-induced false-positives. S-PrediXcan was used to analyze both European ancestry and multiethnic GWAS summary data for pulmonary function tests from the current study.

S-PrediXcan analysis was performed using the following publicly available tissue-specific expression models (http://predictdb.org) from the GTEx project v6p^28^: (1) GTEx Lung (278 samples) and (2) GTEx whole blood (338 samples). Approximately, 85% of participants in GTEx are white, 12% African American, and 3% of other races/ethnicities. Gene-based S-PrediXcan results were filtered on the following: (1) proportion of SNPs used = (*n* SNPs available in GWAS summary data)/(*n* SNPs in prediction model) > 0.6, and (2) prediction performance *R*-squared > 0.01. Following application of S-PrediXcan to each of the GWAS summary data sets, we computed Bonferroni-corrected *P* values derived as the nominal *P* value for each gene-based test divided by the number of genes passing specified filters in each analysis to test whether genetically regulated gene expression was associated with the trait of interest. The genome-wide S-PrediXcan results were then merged with novel loci from the current GWAS study by identifying all matches in which the novel locus SNP was within 500kb of the start of the gene.

We further incorporated a Bayesian colocalization approach^53^ to interpret the extent to which S-PrediXcan results may have been influenced by LD within the region of interest. The Bayesian colocalization procedure was run using the following priors: p1 = 1e−4; prior probability SNP associated to trait 1, p2 = 1e−4; prior probability SNP associated to trait 2, p12 = 1e−5; prior probability SNP associated to both traits. The procedure generated posterior probabilities that correspond to one of the following hypotheses: a region is (H0) has no association with neither trait, (H1) associated with PFT phenotype but not gene expression, (H2) associated with gene expression but not PFT phenotype, (H3) associated with both traits, due to two independent SNPs, and (H4) associated with both traits, due to one shared SNP.

### Druggable targets

We searched annotated gene lists against the ChEMBL database (v22.1, updated on November 15, 2016) to identify genes as targets of approved drugs or drugs in development. In addition, we used the Ingenuity Pathway Analysis (IPA, www.ingenuity.com, content of 2017-06-22) to identify drug targets and upstream regulators of the gene lists. We reported the upstream regulators in the following categories, biologic drug, chemical—endogenous mammalian, chemical—kinase inhibitor, chemical—other, chemical drug, chemical reagent, and chemical toxicant.

### Data availability

The complete meta-analysis results have been deposited in the database of Genotypes and Phenotypes (dbGaP) under the CHARGE acquisition number [phs000930]. GWAS data for most US studies are already available in dbGAP. For all other studies, please send requests to the study PI or Stephanie London (london2@niehs.nih.gov) who will forward them to the relevant party. Requests for METAL code can be directed to Stephanie London.

## Electronic supplementary material

Supplementary Information

Description of Additional Supplementary Files

Supplementary Data 1

Supplementary Data 2

Supplementary Data 3
