# Supplementary material for: Multiethnic meta-analysis identifies ancestry-specific and cross-ancestry loci for pulmonary function
Source: Nat Commun. 2018 Jul 30;9:2976. doi: 10.1038/s41467-018-05369-0 (PMC6065313; doi:10.1038/s41467-018-05369-0)
Supplement: Supplementary file 1 — Supplementary Information [file 41467_2018_5369_MOESM1_ESM.pdf]

Supplementary Information

Multiethnic meta-analysis identifies ancestry-specific and cross-ancestry loci for pulmonary function

Wyss et al.

Supplementary Note 1

Meta-Analysis Study Descriptions
Provided by cohort investigators and/or detailed in the supplements of Hobbs et al [1] or Loth et al [2]

Age Gene Environment Susceptibility (AGES) Study

The Reykjavik Study cohort originally comprised a random sample of 30,795 men and women born in 1907-1935 and living in Reykjavik in 1967. A total of 19,381 attended, resulting in 71% recruitment rate. The study sample was divided into six groups by birth year and birth date within month. One group was designated for longitudinal follow-up and was examined in all stages. One group was designated a control group and was not included in examinations until 1991. Other groups were invited to participate in specific stages of the study. Between 2002 and 2006, the AGES Reykjavik study re-examined 5,764 survivors of the original cohort who had participated before in the Reykjavik Study. Pulmonary function was obtained by spirometry (Vitalograph). Testing was conducted with the participant in a standing position and a disposable mouthpiece. Participants were shown how to perform the maneuver by the technician before testing. A successful test session was defined as at least three acceptable maneuvers.

Agricultural Lung Health Study (ALHS)

The Agricultural Lung Health Study (ALHS) is a case-control study of current asthma among farmers and their spouses nested within the prospective Agricultural Health Study (AHS data releases P3REL201209.00, PIREL201209.00 and AHSREL201304.00) [3-5]. Informed consent was obtained from all participants and the study was approved by the IRB at NIEHS. Pulmonary function was measured by trained staff during in-home visits with an EasyOne® Spirometer (NDD Medical Technologies, Chelmsford, MA) based on American Thoracic Society guidelines. Genotyping based on the UK Biobank Axiom Array (Axiom_UKB_WCSG) by Affymetrix Axiom Genotyping Services (Affymetrix, Inc., Santa Clara, CA) was completed using DNA extracted from blood (96%) or saliva (4%) collected from ALHS participants during the home visit. Variants with missing rate>5%, Hardy-Weinberg $p<1x10^{-6}$, or MAF>5% were excluded. Participants with missing call rate>5%, IBS distance>0.9, or sex discrepancies were excluded. The current analysis includes 1,180 asthma cases and 1,664 non-asthma cases of European ancestry with genotyping data, pulmonary function measures and complete covariate information. Imputation was performed using the 1000 Genomes Integrated phase 1 version 3 reference panel (released March 2012) in IMPUTE2. Linear regression was performed separately in cases and non-cases using FAST (https://bitbucket.org/baderlab/fast/wiki/Home).

The Atherosclerosis Risk in Communities (ARIC) Study

The Atherosclerosis Risk in Communities Study (ARIC) [6], is a population based study of risk factors for atherosclerosis and its sequelae in adults from four U.S. field centers aged 45-64 at recruitment in 1987-1989. Institutional Review Board (IRB) approval was obtained at all associated study centers and informed consent was obtained for all participants. ARIC spirometry measurements were made with a Collins Survey II water-seal spirometer (Collins Medical, Inc.) and Pulmo-Screen II software (PDS Healthcare Products, Inc.). Genotyping was done using the AffymetrixGeneChip SNP Array 6.0 and imputation was performed using IMPUTE2. Quality control steps for genotyping data included exclusions for call rate <95%, minor allele frequency <1%, HWE P<10-5, no chromosomal location, suspected first-degree relative of an included individual based on genotype data, or more than 8 standard deviations for any of the first ten principal components. The current analysis includes 8,878 Caucasian individuals

and 1,837 African American individuals with genotyping data, pulmonary function measures and complete covariate information. Imputation was performed using the 1000 Genomes Integrated phase 1 version 3 reference panel (released March 2012) in IMPUTE2. Linear regression was performed using FAST (https://bitbucket.org/baderlab/fast/wiki/Home).

Coronary Artery Risk Development in Young Adults (CARDIA)

Cross-sectional analyses of data from year 0 examination of the Coronary Artery Risk Development in Young Adults (CARDIA) cohort was performed. During 1985 -1986, CARDIA randomly recruited 5,115 black and white men and women, aged 18 to 30 years, from the general population at Birmingham, Alabama; Chicago, Illinois; and Minneapolis, Minnesota; and from the membership of the Oakland Kaiser -Permanente Health Plan in Oakland, California. Detailed methods, instruments, and quality control procedures are described at the CARDIA website (http://www.cardia.dopm.uab.edu/ex_mt.htm) and in other published reports [7,8]. Spirometric pulmonary function testing were performed using the Collins survey 8-liter water-sealed spirometer and the Eagle II microprocessor (Warren E. Collins, Inc., Braintree, MA) in a sitting position with noseclips, as per the 1979 American Thoracic Society criteria [9]. Specifically, each subject performed a minimum of three trials with expirations recorded to the FVC plateau, which occurs after six seconds of expiration in adult males and was maintained for at least one second before terminating the forced expiratory maneuver. If, at the end of the three trials, there were at least three acceptable tracings, and with the maximum FVC and FEV_1 reproduced to within 5% or 100 mL, whichever is greater, no more trials were performed. The current analysis includes 1661 whites and 895 African-American participants with available genotyping and pulmonary function data. Imputation analyses were performed using BEAGLE version 3.3.2 and the 1000 Genomes Integrated phase 1 version 1 reference panel (released June 2011) for whites and version 3 for African-Americans. Association analyses were performed using the ProbABEL package in R.

Cardiovascular Health Study (CHS)

The Cardiovascular Health Study (CHS) is a population-based cohort study of risk factors for coronary heart disease and stroke in adults ≥65 years conducted across four field centers [10] (NCT00005133 and NCT00149435). Local IRB approval was obtained at participating centers and written informed consent was obtained for all participants. The original predominantly European ancestry cohort of 5,201 persons was recruited in 1989-1990 from random samples of the Medicare eligibility lists; subsequently, an additional predominantly African-American cohort of 687 persons was enrolled in 1992-1993 for a total sample of 5,888. Pulmonary function testing was conducted by trained spirometry technicians. FEV_1/FVC and FEV_1 measures met American Thoracic Society criteria for acceptability. The pulmonary function measures analyzed were from the baseline visit for the original cohort and from one year after baseline for the second cohort. Measurements were made with a Collins Survey I water-seal spirometer (Collins Medical, Inc.) and software from S&M Instruments. Blood samples were drawn from all participants at their baseline examination and DNA was subsequently extracted from available samples. European ancestry participants were excluded from the GWAS study sample due to the presence at study baseline of coronary heart disease, congestive heart failure, peripheral vascular disease, valvular heart disease, stroke or transient ischemic attack. Genotyping was performed at the General Clinical Research Center's Phenotyping/Genotyping Laboratory at Cedars-Sinai among CHS participants who consented to genetic testing and had DNA available using the Illumina 370CNV BeadChip system (for European ancestry participants, in 2007) or the Illumina HumanOmni1-Quad_v1 BeadChip system (for African-American participants, in 2010). Additional genotypes for European ancestry participants were provided from the ITMAT-Broad-CARe (IBC) Illumina iSELECT chip. Imputation was performed using 1000

Genomes Phase 1 v3 haplotypes and minimac [11] (2012-11-16) for European ancestry participants and IMPUTE version 2.2.2 for African-American participants. Linear regression was performed in R, adjusting for CHS clinic (4 sites) and PCs 1-2 (for European ancestry participants) and PCs 1-5 (for African-American participants).

Family Heart Study (FamHS)

Study participants were from the NHLBI Family Heart Study (FamHS). Details of the design of FamHS have been described previously [12]. Spirometry was performed during the participant's clinical examination using a computerized volume-based spirometer and was reported at body temperature and pressure, saturated. FEV1 and FVC were measured. All FamHS participants were genotyped using Illumina arrays (either HumMap550K, Human 6100-Quadv1, or Human 1M-Duov3). Quality control was performed before imputation and included assessment of Mendelian errors (LOKI [13]), verification of reported pedigree relationships using GRR [14], and exclusion of SNPs failing quality control (HWE, MAF filter, not in 1000 Human Genomes (HG), allelic mismatch with 1000 HG). SNPs passing quality control were used for the 1000 HG imputation. Imputation was performed by genotyping platform (550K, 610K, or 1Mil). MaCH (version1.0.16) [15,16] was used to identify phased haplotypes of FamHS subjects (pre-imputation) using the framework map of SNPs that passed the quality control criteria: call rate >0.98; MAF >0.01; no deviation from HWE (p>1E-06); allelic match with 1000 HG; and present in 1000 HG genotyping, which left a total of 501,404 (550K), 530,979 (610K), and 910,456 (1Mil) SNPs for the haplotype phasing and imputation.

Framingham Heart Study (FHS)

Details on pulmonary function in the FHS have been previously published [17,18]. The study was IRB approved by Boston University Medical Center IRB and all participants provided written informed consent. Data from the most recent exam for each of the three generations of families participating in the FHS were analyzed. Genotypes were from the Affymetrix 500K array supplemented by the Affymetrix MIPS 50K. From a total number of 549,781 genotyped SNPs, 412,053 were used with the MaCH [15,16] program for phasing. A total of 137,728 genotyped SNPs were removed based on the following filtering criteria: 22,018 SNPs for Hardy-Weinberg Equilibrium p-value of less than 1x10-6, 48,285 SNPs for a call rate of less than 96.9%, 66,063 SNPs for a minor allele frequency of less than 0.01, 82 SNPs due to not mapping correctly from Build 36 to Build 37 locations, 428 SNPs missing a physical location, 25 SNPs for number of Mendelian errors greater than 1000, 786 SNPs due to not being on chromosomes 1-22 or X and 41 SNPs because they were duplicates. MaCH/minimac [11,15,16] were used in this imputation to impute the FHS sample using the November 2010 release of the 1000 Genomes [19] multi-ethnic panel comprised of 1,092 samples. We used lmekin implemented in the R package
kinship to account for familial relationship using a mixed effect model, adjusted for sex, age, smoking status, pack years and principal component 1 (to adjust for population stratification).

Genetics of Overweight Young Adults (GOYA) Males

The GOYA (Male) cohort is a longitudinal case-cohort (obese, non-obese) study comprising a randomly (1%) selected control group and all extremely overweight men identified among 362,200 Caucasian men examined at the mean age of 20 years at the draft boards in Copenhagen and its surrounding areas during 1943–1977. Obesity was defined as 35% overweight relative to a local standard in use at the time (mid 1970’s), corresponding to a BMI ≥31.0 kg/m2, which proved to be above the 99th percentile. All of

the obese and 50% of the random sampled controls, who were still living in the region, were invited to a follow-up survey in 1992–94 at the mean age of 46 years, at which time the blood samples were taken and genotyping were performed for a total of 673 extremely overweight and 792 controls [20,21]. With a sampling fraction of 0.5% (50% of 1%), the controls represent about 158,000 men among whom the case group was the most obese.

Pulmonary function was measured with a Vitalograph Pulmonary Function Test Printer (Maidenhead, U.K.). The participant was placed in a sitting position and asked to inhale to total lung capacity before beginning the forced expiration abruptly, and without hesitation. Maximum effort was to be exerted throughout the expiration. The measured variables were forced vital capacity (FVC) and forced expiratory volume in the first second of expiration (FEV1). The percentage expired in the first second was calculated from the best results of at least two measurements FEV1/FVC %. At least two reproducible measurements that differed less than 5% from each other were made. The values from the highest measurement were used. The values were compared with normal predicted values in relation to height (nearest lower cm), age and sex for each person. The predicted value equations by The European Coal and Steel Union were used [Quanjer PH (compiler and editor). Standardizing lung function testing. Report Working Party 'Standardization of Lung Function tests', European Community for Coal and Steel, Luxembourg. Bull Eur Physiopathol Respir, 1983, 19 (suppl 5), 1–95.] The spirometer was calibrated with a 1-l syringe on a daily basis. The study was approved by the regional scientific ethics committee and by the Danish Data Protection Board.

Genome-wide genotyping on the Illumina 610 k quad chip was carried out at the Centre National de Ge´notypage (CNG), Evry, France [21]). We excluded SNPs with minor allele frequency, 1%, .5% missing genotypes or which failed an exact test of Hardy-Weinberg equilibrium (HWE) in the controls. We also excluded any individual who did not cluster with the CEU individuals (Utah residents with ancestry from northern and western Europe) in a multidimensional scaling analysis seeded with individuals from the International HapMap release 22. Genotypes were phased using mach-1.0.16 and data were then imputed using minimac-comp using the GIANT reference panel: chr1.phase1_release_v3.20101123.snps_indels_svs.genotypes.refpanel.ALL.vcf.gz. Linear regression based association testing was performed using Quicktest Version 0.95 (http://toby.freeshell.org/software/quicktest.shtml).

Hispanic Community Health Study/Study of Latinos (HCHS/SOL)

The HCHS/SOL is a community-based cohort study of 16,415 self-identified Hispanic/Latino persons aged 18-74 years designed to examine risk factors of chronic disease among Hispanic/Latino persons. Participants were selected from households in predefined census-block groups from four United States field centers (Chicago, Miami, the Bronx, and San Diego). The census-block groups were chosen to provide diversity with regard to socioeconomic status and national origin or background. Households were selected using a stratified, two-stage area probability sample design with oversampling to increase the likelihood that a selected address would yield a Hispanic/Latino household [22] HCHS/SOL participants were recruited between 2009 and 2011 and underwent a baseline clinical examination [23] including biological, behavioral, and sociodemographic assessments. Of 39,384 individuals who were screened and selected and who met the eligibility criteria, 16,415 Hispanics (41.7%) from 9,872 households were enrolled. The study oversampled those ages 45–74 years to facilitate examination of target outcomes [24].

Consenting HCHS/SOL subjects were genotyped at Illumina on the HCHS/SOL custom 15041502 B3 array. The custom array comprised of the Illumina Omni 2.5M array (HumanOmni2.5-8v.1-1), ancestry-informative markers, variants from prior GWAS studies, and additional custom content including ~ 150,000 SNPs selected from samples in the 1000 Genomes phase 1 data to capture Amerindian genetic variation [25]. We applied standardized quality-assurance and quality-control methods [26] as previously describe [27]. Genome-wide imputation was carried out with the full, cosmopolitan 1000 Genomes Project phase 1 reference panel [19]. HCHS/SOL participants were classified in six genetic analysis groups: Central American, Cuban, Dominican, Mexican, Puerto Rican, and South American [27]. Exclusion criteria were lack of valid spirometric or genetic data, missing covariates, Asian ancestry and participants who were not classified in the Hispanic/Latino ancestry groups. Of the 12,803 participants consented for extracting genotypes information, 12,059 had complete data for association study of lung function. Since we performed stratified analysis, in which each genetic analysis group was analyzed separately and the GWASs across the analyses groups were meta-analyzed, we excluded 284 individuals who were genetically related (kinship coefficient larger than 2^{-11} in the kinship matrix, degree three relative) to individual from a different genetic analysis groups. 11,775 remained for analysis. Spirometry was conducted in accordance with American Thoracic Society/European Respiratory Society guidelines [28] using a dry rolling sealed spirometer with automated quality checks (Occupational Marketing, Houston, TX). Participants with pre-bronchodilator FEV_1/FVC ratios less than 70% or less than the lower limit of normal were selected for post-bronchodilator spirometry. We analyzed three outcomes: FEV_1, FVC, and FEV_1/FVC ratio.

Association analysis in the HCHS/SOL were done using linear mixed models, adjusting for the study-specific variables study center, sampling weights, and 5 first principal components of ancestry, in addition to other adjusting variables that were common to all studies. Variance components were estimated due to genetic relatedness, household, and community block unit sharing.

Health Aging and Body Composition (Health ABC)

The Health Aging and Body Composition (Health ABC) study is a prospective observational cohort of well-functioning individuals aged 70–79 years, which recruited 3,075 community-dwelling African and European Americans, men and women, at two field centers at the University of Pittsburgh, Pennsylvania and the University of Tennessee, Memphis. Spirometry was performed with a horizontal dry rolling seal spirometer (SensorMedics Corporation, Yorba Linda, CA) connected to a computer. Pulmonary function testing followed ATS guidelines for the standardization of spirometry, and is described in detail elsewhere [29]. Health ABC genotyped 1,794 self-described white participants and 1,281 self-described black participants at baseline with available DNA and consent to genetics testing; of these 1,661 white participants and 1,139 black participants passed quality control benchmarks (call rate > 97%, no sex mismatch, and cryptic relatedness). For this study, 1,472 white participants and 943 black participants had pulmonary function measurements and complete data on covariates.

Healthy Twin

The Healthy Twin Study, Korea (HT) is a prospective multi-center cohort study based on a large nation-wide family database. The HT has recruited 3,880 individuals, with 815 pairs of adult like -sex twins over age 30 and their first-degree families as of 2015 February. Being a twin was the only criteria for participation, and the participants were not selected by their health status. The protocols and measurements are described in detail in previous report [30-32]. Extended questionnaire and health examination are provided at recruitment and follow-up study is ongoing in every 3 year, and the 3rd

wave survey was finished in 2014. Spirometry was performed by trained paramedical personnel using a Vmax22® (Sensormedics, Yorba Linda, California, USA), according to American Thoracic Society (ATS)/European Respiratory Society (ERS) guidelines. FEV1, FEV5, FVC, FEV1/FVC ratio, FEV1/FVC ratio, FEF 50%, FEF 25 -75%, FEF 200-1200 ml, PEF, and FET 100% were measured. Genotyping of the HT was performed using two platforms. First 1,861 individuals were analyzed by Affymetrix Genome Wide Human SNP Array 6.0 (Affymetrix, Inc., Santa Clara, CA, USA). Genotyped markers with a Hardy-Weinberg Equilibrium ($p<0.001$), low call rate (<95%), and low minor allele frequency (MAF<0.01) were excluded. In addition to the conventional quality control, Mendelian and non-Mendelian errors mimicking double-recombinations were detected by within-family comparisons using Merlin. This error-checking step further detected and deleted 63,777 erroneous markers, leaving 516,452 SNPs. Second, 1,047 individuals were analyzed using Illumina HumanBeadCore chip with around 300K SNPs, and the same quality control method and criteria is applied (in process). Imputation was done using the 1000 Genomes Phase 1 and Phase 3 East Asian and Korean Reference Genome (hg19).

Hunter Community Study (HCS)

The Hunter Community Study is a population-based prospective cohort study of community -dwelling men and women aged 55–85 years of age who reside in Newcastle, New South Wales (NSW), Australia. The cohort comprises 3253 participants that were randomly selected from the NSW State electoral roll between 2004 and 2007. Spirometry was performed using electronic spirometers (Micro Medical SpiroUSB, Cardinal Health, Kent, UK) with Spida 5 software (Carefusion Ltd, Kent, UK) according to American Thoracic Society (ATS) guidelines, i.e. 3 acceptable traces with no more than 8 attempts. Key lung function measures were FEV1 and FVC. The spirometer was calibrated daily using a 3-Litre syringe.

Jackson Heart Study (JHS)

Jackson Heart Study (JHS) is a large, population-based observational study evaluating the etiology of cardiovascular diseases and related disorders among African Americans residing in the three counties (Hinds, Madison, and Rankin) that make up the Jackson, Mississippi metropolitan area [33,34]. Data and biologic materials have been collected from 5,301 participants, including a nested family cohort of 1,498 members of 264 families. The age at enrollment for the unrelated cohort was 35-84 years; the family cohort included related individuals >21 years old. During a baseline examination (2000-2004) and two follow-up examinations (2005-2008 and 2009-2012), participants provided extensive medical and social history, had an array of physical and biochemical measurements and diagnostic procedures, and provided blood for genomic DNA [35]. The study population is characterized by a high prevalence of diabetes, hypertension, obesity, and related disorders. Annual follow-up interviews and cohort surveillance are ongoing.

Korean Association Resource (KARE3)

Details on the Korean Association Resource project (KARE) have been previously published [36,37]. KARE was initiated in 2007 to undertake genome-wide analyses analysis among 10,038 participants in the rural-based Ansung and city-based Ansan South Korean cohorts. The study was approved at appropriate IRBs from participating institutions and participants provided informed consent. KARE3 data were obtained from the third phenotype collection in 2008; lung function was collected using a portable spirometer (Vmax-2130, Sensor Medics, Yorba Linda, CA, USA) according to standardized protocols of the American Thoracic Society (ATS). Genotyping was performed using the Affymetrix Genome-Wide Human array 5.0 (Affymetrix, Inc., Santa Clara, CA, USA). We performed imputation using IMPUTE2 and

the 1000 Genomes [19] Phase 1 East Asian and Phase 3 cosmopolitan panels. Markers were converted to genotype from dosage with call rate ≥ 95%, minor allele frequency ≥ 1%, p for HWE ≥ $1.0x10^{-5}$, imputation quality score ≥ 0.9. Linear regression was performed using PLINK [38].

LifeLines

The LifeLines Cohort Study is a large population-based cohort study and biobank that was established as a resource for research on complex interactions between environmental, phenotypic and genomic factors in the development of chronic diseases and healthy aging [39-41]. Between 2006 and 2013, inhabitants of the northern part of The Netherlands and their families were invited to participate, thereby contributing to a three-generation design. Participants visited one of the LifeLines research sites for a physical examination, including pre-bronchodilator spirometry following ATS guidelines. All participants signed an informed consent form before they received an invitation for the physical examination. The LifeLines Cohort Study is conducted according to the principles of the Declaration of Helsinki and in accordance with research code University Medical Center Groningen (UMCG), The Netherlands. The LifeLines study is approved by the medical ethical committee of the UMCG.

Blood samples for a subset of individuals were genotyped using the Illumina CytoSNP-12v2 array. Independent Caucasian-ancestry samples (n = 13,436) have been imputed using the 1000 Genomes[19] phase1 v3 reference panels. Quality control of the data is based on SNP filtering on MAF above 0.001, HWE p-value > $1x10^{-4}$, call rate of 0.95 using PLINK[42], and PCA to check for population outliers resulting in 268,407 SNPs and 13,436 samples kept for genome-wide association analysis. Before imputation, the genotypes were pre-phased using SHAPEIT2[43] and aligned to the reference panels using Genotype Harmonizer (www.molgenis.org/systemsgenetics) in order to resolve strand issues. The samples were imputed using minimac[11] (version 2012-10-09), yielding 28,681,763 SNPs. All analysis were performed in software package PLINK version 1.07 [38,42].

Long Life Family Study (LLFS)

The Long Life Family Study (LLFS) is a family -based cohort study that enrolled 4559 long-lived probands and their siblings (n=1445), their offspring (n=2329) and spousal controls (n=785) recruited from 3 U.S. field centers (Boston University Medical Center in Boston MA, Columbia College of Physicians and Surgeons in New York City NY, and the University of Pittsburgh in Pittsburgh PA) and the University of Southern Denmark. Participants were chosen from the Center for Medicare and Medicaid Services lists of Medicare enrollees who were ≥ 89 years old during study recruitment and lived in zip codes near (within 3 hours driving distance) one of the three U.S field centers. The University of Southern Denmark used the Danish National Register of Persons to identify individuals who were ≥ 90 years during the study recruitment period [44]. Only families with a Family Longevity Selection Score (FLoSS) of 7 or higher [45] who had the proband, at least one living sibling, and one of their living offspring (minimum family size of 3) who could give informed consent and were willing to participate in the interview and examination including donating a blood sample were eligible to participate in this study. This strategy led to the enrollment of families with the greatest potential utility for phenotypic and genetic studies of exceptional survival in families. The interviews and examinations were conducted in the home setting with portable equipment by centrally trained and certified research assistants using a common standardized protocol. Lung function was measured with a portable spirometer (EasyOne™, N DD Medical Technologies, Andover, MA) using American Thoracic Society guidelines. After excluding participants with non-European Ancestry (n=6), participants with poor quality spirometry readings (n=295), pulmonary fibrosis (n=11) and those with lung volume reducing surgery (n=14) and those with

missing genotypes (n=344), 3889 participants were included in the present analysis. The Illumina Human Omni chip 2.5 v1, was used to genotype all the LLFS participants at the Center for Inherited Disease Research (CIDR). A threshold call rate of > 98% per marker, identified 2,134,578 SNPs. Principal Components (PCs), for controlling for population structure, were produced with EIGENSTRAT [46] on 1,522 LLFS unrelated individuals using 116,867 tag SNPs, where in advance any SNPs with MAF < 5%, HWE p< 1e-6, and with missing genotypes were excluded. Imputations were performed based on the cosmopolitan phased haplotypes of 1000 Human Genome (1000HG, version 2010-11 data freeze, 2012-03-04 haplotypes) using MACH and MINIMACH [15,16] and a total of 38.05 million SNPs were imputed.

Multi-Ethnic Study of Atherosclerosis (MESA)

Multi-Ethnic Study of Atherosclerosis (MESA) is a longitudinal study of subclinical cardiovascular disease and risk factors that predict progression to clinically overt cardiovascular disease or progression of the subclinical disease [47]. Between 2000 and 2002, MESA recruited 6,814 men and women 45 to 84 years of age from Forsyth County, North Carolina; New York City; Baltimore; St. Paul, Minnesota; Chicago; and Los Angeles. Exclusion criteria were clinical cardiovascular disease, weight exceeding 136 kg (300 lb.), pregnancy, and impediment to long-term participation. The MESA Family Study recruited 1,595 African American and Hispanic participants, generally siblings of MESA participants, using the same inclusion and exclusion criteria as MESA except that clinical cardiovascular disease was permitted. The MESA Air Pollution Study recruited an additional 257 participants from Los Angeles and Riverside County, CA, and Rockland County, NY, using the same criteria as MESA, except that participants were ages 50 to 89 who lived in the area more than 50% of the year and had no plans to move in the next five years[48]. The MESA Lung Study performed spirometry following the 2005 ATS/ERS guidelines in a subset of the MESA and MESA Family Studies and all of the new recruits in the MESA Air Pollution Study, as previously described[49]. All participants provided informed consent and the protocols of MESA were approved by the IRBs of collaborating institutions and the National Heart, Lung and Blood Institute.

Participants in the original MESA cohort, the MESA Family Study and the MESA Air Pollution Study who consented to genetic analyses were genotyped in 2009 using the Affymetrix Human SNP array 6.0. Genotype quality control for these data included filter on SNP level call rate < 95%, individual level call rate < 95%, heterozygosity > 53%, described previously[50]. The cleaned genotypic data was deposited with MESA phenotypic data into dbGaP as the MESA SHARe project (study accession phs000209); 8,224 consenting individuals (2,685 White, 2,588 non-Hispanic African-American, 2,174 Hispanic, 777 Chinese) were included, with 897,981 SNPs passing study specific quality control (QC). For GWAS, IMPUTE version 2.2.2 was used to perform imputation for the MESA SHARe participants using the cosmopolitan 1,000 Genomes [19] Phase 1 v3 March 2012 reference set. For the current effort, analyses were restricted to MESA White and African-American participants with measures of pulmonary function. Linear regression was performed using SNPTEST v2.4.0 [51].

The Netherlands Epidemiology of Obesity (NEO) Study

The NEO study was designed for extensive phenotyping to investigate pathways that lead to obesity-related diseases [52]. The NEO study is a population-based, prospective cohort study that includes 6,671 individuals aged 45–65 years, with an oversampling of individuals with overweight or obesity. At baseline, information on demography, lifestyle, and medical history have been collected by questionnaires. In addition, samples of 24-h urine, fasting and postprandial blood plasma and serum, and DNA were collected. Genotyping was performed using the Illumina HumanCoreExome chip, which was subsequently imputed to the 1000 genome reference panel. Participants underwent an extensive

physical examination, including anthropometry, electrocardiography, spirometry, and measurement of the carotid artery intima-media thickness by ultrasonography. In random subsamples of participants, magnetic resonance imaging of abdominal fat, pulse wave velocity of the aorta, heart, and brain, magnetic resonance spectroscopy of the liver, indirect calorimetry, dual energy X-ray absorptiometry, or accelerometry measurements were performed. The collection of data started in September 2008 and completed at the end of September 2012. Participants are currently being followed for the incidence of obesity-related diseases and mortality.

1982 Pelotas (Brazil) Birth Cohort

The maternity hospitals in Pelotas, a southern Brazilian city (current population ~330,000), were visited daily in the year of 1982. The 5,914 liveborns whose families lived in the urban area were examined and their mothers interviewed. Information was obtained for more than 99% of the livebirths. These subjects have been followed-up at the following mean ages: 11.3 months (all children born from January to Abril 1982; n=1457), 19.4 months (entire cohort; n=4934), 43.1 months (entire cohort; n=4742), 13.1 years (random subsample; n=715), 14.7 years (systematic subsample; n=1076); 18.2 (male cohorts attending to compulsory Army recruitment examination; n=2250), 18.9 (systematic subsample; n=1031), 22.8 years (entire cohort; n=4297) and 30.2 years (entire cohort; n=3701). Details about follow-up visits and available data can be found in the two Cohort Profile papers [53,54]. DNA samples (collected at the mean age of 22.8 years) were genotyped for ~2.5 million of SNPs using the Illumina HumanOmni2.5-8v1 array (which includes autosomal, X and Y chromosomes, and mitochondrial variants). After quality control, the data were prephased using SHAPEIT and imputed using IMPUTE2 based on 1000 Genomes haplotypes.

Rotterdam Study (RS) I, II, III

The Rotterdam Study is a prospective population-based cohort study founded in 1990 in a suburb of Rotterdam, the Netherlands [55,56]. The first cohort (RS-I) consists of 7,983 participants, aged 55 years and over. The second cohort (RS-II) was recruited in 2000 with the same inclusion criteria. The third cohort (RS-III) consists of 3,932 participants, aged 45 years and over and was recruited in 2006. The Rotterdam Study was approved by the institutional review board (Medical Ethics Committee) of the Erasmus Medical Center and by the review board of The Netherlands Ministry of Health, Welfare and Sports. All participants provided written informed consent. Spirometry was performed by trained paramedical personnel using the Master Screen® PFT Pro (CareFusion, San Diego, CA) according to the American Thoracic Society(ATS)/European Respiratory Society (ERS) guidelines.

A total of 6,318 subjects were genotyped in RS I, 2,516 in RS II and 3,540 subjects in RS III. Exclusions included a call rate < 98%, Hardy-Weinberg $P < 1x10^{-6}$ and MAF < 0.01%. A total of 6,291 for RS I, 2,157 for RS II and 3,048 for RS III passed genotyping quality control. Regression coefficients and their standard errors were determined using the ProbABEL [57] program according to an additive model.

Replication Study Descriptions

UK BiLEVE

UK BiLEVE is a subset of UK Biobank and is described in detail elsewhere [58]. In brief, UK Biobank comprised 502,682 individuals of which 472,858 were of self-reported European ancestry and 275,939 had at least two measures of FEV_1 and FVC and passed ATS/ERS quality criteria [59]. Spirometry data

was obtained using a Vitalograph Pneumotrac 6800 (Buckingham, UK); at least two measures were obtained. From these 275,939 individuals, 50,000 individuals were selected based on % predicted FEV_1 such that 10,000 individuals with low FEV_1, 10,000 individuals with near-average FEV_1, and 5,000 with high FEV_1 were selected from amongst never smokers (total n=105,272) and the same numbers from amongst the heavy smokers (total n=46,758). Equal numbers of males and females were selected and the number of individuals selected within each age-sex band was proportional to the number of individuals available for sampling for each band. Genotyping was undertaken using the Affymetrix Axiom UK BiLEVE array[58] and imputed to the 1000 Genomes Project[19] Phase 1 and UK10K[60] combined panel. Of the 50,008 samples selected, 48,943 unrelated individuals passed all genotype and sample quality control steps and were used as the sampling frame for selection of samples included in the analysis described here. The UK Biobank received ethics approval from the National Health Service National Research Ethics Service (Ref 11/NW/0382). All participants provided written informed consent. Analyses were carried out using the score test, implemented in SNPTEST v2.5b4 and assuming an additive genetic model of genotype dose. For never smokers, sex, age, height and the first 10 ancestry principal components were included as covariates. For heavy smokers, pack years was also included as a covariate.

UK Biobank

The UK Biobank is a population-based study of health outcomes in the United Kingdom (UK). Approximately 500,000 people aged 40-69 years were recruited between 2006-2010 (http://www.ukbiobank.ac.uk/about-biobank-uk/). Preliminary results from analyses of 1000 Genomes imputed variants and many health outcomes are publicly available (https://sites.google.com/broadinstitute.org/ukbbgwasresults/home; http://www.nealelab.is/blog/2017/9/11/details-and-considerations-of-the-uk-biobank-gwas). Analyses of FEV_1 and FVC (best measures) included 255,492 individuals and were adjusted for sex and 10 PCs.

COPDGene

Details of the COPDGene Study (NCT00608764, www.copdgene.org) have been described previously [61,62]. Local IRB approval was obtained at all study centers and all study participants provided written informed consent. Eligible subjects were of non-Hispanic white or African-American ancestry, aged 45-80 years old, with a minimum of 10 pack-years of smoking and no lung disease (other than COPD or asthma). Genotyping was performed by Illumina (San Diego, CA) on the HumanOmniExpress array. Subjects were excluded for missingness, heterozygosity, chromosomal aberrations, gender check, population outliers, and cryptic relatedness. Genotyping at the Z and S alleles was performed in all subjects. Subjects known or found to have alpha-1 antitrypsin deficiency were excluded. Markers were excluded based on missingness, Hardy-Weinberg P-values, and low minor allele frequency. Imputation on the COPDGene cohorts was performed using the Michigan Imputation Server (Eagle version 2.3 phasing) and the HRC 1.1 reference panel. Variants with an r^2 value of ≤ 0.5 were removed from further analysis, and genotype dosages were extracted and analyzed in R. Linear regression on post-bronchodilator lung function was performed in 3,219 African American subjects adjusting for age, age^2, sex, height, $height^2$, smoking status, pack-years, and principal components of ancestry. Analysis of FVC was additionally adjusted for weight.

SAPPHIRE

The Study of Asthma Phenotypes and Pharmacogenomic Interactions by Race-ethnicity (SAPPHIRE) is an ongoing cohort study of asthma, related phenotypes, and medication response [63,64]. Members of large healthcare system from southeast Michigan were eligible to participate if they were age 12-56 years at the time of enrollment, had a physician diagnosis of asthma, and no prior history of congestive heart failure or chronic obstructive pulmonary disease. Control patients in SAPPHIRE met the same criteria for study entry with the exception of not having an asthma diagnosis. SAPPHIRE participants have retrospective and prospective clinical records of medical care and treatments received, and there is a biorepository of their biological samples. The race-ethnic distribution of the cohort reflects that of the southeast Michigan and the Detroit metropolitan area.

Genome-wide genotyping was performed using the Axiom AFR array (Affymetrix Inc., Santa Clara, CA); 574,370 SNPs passed quality control and data were available for 1891 subjects. The software program, PLINK2 [65], was used to generate principal components (PCs) to adjust for underlying population structure. We imputed to the 1000 Genomes Project phase 1 integrated dataset using IMPUTE2 [11].

SAGE and GALA

The Study of African Americans, Asthma, Genes and Environments (SAGE) includes 1,989 African American asthma cases and controls recruited from clinics in the San Francisco Bay Area (1,176 cases, 813 controls)[66]. The median age was 14 years (25th-75th percentile 11-17) for cases and 17 years (25th-75th percentile 13-19) for controls. The analysis included 1,405 individuals with baseline lung function measurements. Asthma was defined as having a history of physician-diagnosed asthma and two or more asthma symptoms (wheezing, coughing, and/or shortness of breath) in the preceding two years. Atopy was not an inclusion criterion for the study. Controls had no reported history of asthma, allergies, lung disease, chronic illness or medication use, coughing, wheezing or shortness of breath in the preceding 2 years, < 10 pack-years of smoking history, and had not smoked in the year preceding enrollment. Genotypes were called on Affymetrix Axiom LAT 1 (World Array 4) and LAT plus HLA genome-wide arrays usingAffymetrix Powertools software. Quality control was performed by removing SNPs with call rates < 95% and/or deviated from Hardy-Weinberg equilibrium (P < 10-6). Samples with call rates <95%, discrepancy between genetic sex and reported sex, or with cryptic relatedness (proportion of identity by descent > 0.3) were removed.

The Genes-environments and Admixture in Latino Americans study (GALA II) includes 4,436 Latino asthma cases and controls recruited from community clinics and hospitals from New York, Chicago, San Francisco, Houston, and Puerto Rico (2,275 cases, 2,161 controls)[67]. The median age of participants was 12 years (25th-75th percentile 10-15) for cases and 13 years (25th-75th percentile 11-17) for controls. The analysis included 2,203 individuals with baseline lung function measurements. Asthma was defined as having a history of physician-diagnosed asthma and two or more asthma symptoms (wheezing, coughing, and/or shortness of breath) in the preceding two years. Atopy was not an inclusion criterion for the study. Controls had no reported history of asthma, allergies, lung disease, chronic illness or medication use, coughing, wheezing or shortness of breath in the preceding 2 years, < 10 pack-years of smoking history, and had not smoked in the year preceding enrollment. Genotyping and QC process were performed as described in SAGE.

Genotype data of all samples were submitted to the Michigan Imputation Server (https://imputationserver.sph.umich.edu/) for imputation to the HRC r1.1 2016 reference panel

(http://www.haplotype-reference-consortium.org/) using the Minimac3 algorithm (http://genome.sph.umich.edu/wiki/Minimac3) and ShapeIT (http://www.shapeit.fr/) as the phasing method. Imputed markers were excluded from the analysis if they were monomorphic or their R-squared statistics were below 0.3. Global ancestry was estimated using ADMIXTURE (https://www.ncbi.nlm.nih.gov/pubmed/19648217) for each individual. For African Americans we assumed a 2-population model of admixture (European and African ancestry), and for Latinos a 3-population model (European, Native American, and African ancestry). Reference haplotypes were from the HapMap phase II CEU (European) and YRI (African), and 71 Native American individuals genotyped on the Axiom LAT1 array as described previously (https://www.ncbi.nlm.nih.gov/pubmed/?term=Case-control+admixture+mapping+in+Latino+populations+enriches+for+known+asthma-associated+genes). In SAGE, linear regression was performed in plink 1.9 (Shaun Purcell, Christopher Chang, www.cog-genomics.org/plink/1.9/) to assess the association of each SNP with percent predicted lung functions. The regression model was adjusted for asthma status, age, sex, height, BMI category, and global African ancestry. In GALA II, baseline FVC in liters was used as the outcome. The linear regression model was adjusted for age, sex, height, ethnicity, global African ancestry, and global Native American ancestry.

COPD Study Description (assessed overlap of PFT loci with COPD loci)

International COPD Genetics Consortium (ICGC)

Details of the ICGC meta-analysis of COPD have been previously published [1]. Briefly, 26 cohorts comprising 15,256 COPD cases and 47,936 controls contributed to the meta-analysis. Modified definitions of GOLD criteria based on pre-bronchodilator spirometry data were used since most cohorts did not have post-bronchodilator spirometry data: forced expiratory volume in 1 s (FEV_1) < 80% and FEV_1 to forced vital capacity (FVC) ratio of < 0.7 for cases and FEV_1 > 80% and FEV_1/FVC > 0.7 for controls. Each cohort used logistic regression to model the effect of variants on COPD adjusting for age, sex, pack-years of smoking, ever smoking status, current smoking status, and ancestry-based principal components, as appropriate for each study. Fixed-effects meta-analyses were performed in METAL.

eQTL and mQTL Study Descriptions (assessed overlap of PFT loci with eQTL/mQTL signals)

Lung eQTL Consortium (Laval, UBC, Groningen)

The details and subjects' characteristics of the lung eQTL study population have been previously described [68-71]. All lung tissue samples were obtained in accordance with Institutional Review Board guidelines at the three sites: Laval University (Quebec, Canada), University of British Columbia (Vancouver, Canada) and Groningen University (Groningen, The Netherlands). All patients provided written informed consent and the study was approved by the ethics committees of the Institut universitaire de cardiologie et de pneumologie de Québec and the UBC-Providence Health Care Research Institute Ethics Board for Laval and UBC, respectively. The study protocol was consistent with the Research Code of the University Medical Center Groningen and Dutch national ethical and professional guidelines ("Code of conduct; Dutch federation of biomedical scientific societies"; http://www.federa.org). Briefly, following standard microarray and genotyping quality controls, 1,111 patients were available including 339 from The University of British Columbia Centre for Heart and Lung Innovation (Vancouver, Canada), 409 from Laval University (Quebec City, Canada) and 363 from the University of Groningen (Groningen, The Netherlands). Gene expression profiling was performed using an Affymetrix custom array (GPL10379) testing 51,627 non-control probesets and normalization was

performed using multi-array average (RMA) [72]. The expression data are available at NCBI Gene Expression Omnibus repository through accession numbers GSE23546. Genotyping was performed on DNA extracted from blood or lung tissue using the Illumina Human1M-Duo BeadChip array, and imputation was performed with MaCH/Minimac software [11] using the 1000G reference panel, March 2012 release. The eQTL analysis was adjusted for age, sex and smoking status in each study separately, and the results were meta-analysed using inverse variance weighting meta-analysis. The resulting eQTLs were categorized into cis-acting (less than 1Mb away from transcription start site) or trans eQTLs (further than 1Mb away or on a different chromosome). Genome-wide significant threshold was set using Benjamini-Hochberg 10% FDR.

Westra (EGCUT, InCHIANTI, Rotterdam Study, Fehrmann, HVH, SHIP-TREND and DILGOM)

Details of the study population and eQTL methods have been previously described [73] (see also https://genenetwork.nl/bloodeqtlbrowser/). Briefly, whole-genome eQTL analysis was performed for 5,311 peripheral blood samples from 7 cohorts: EGCUT (n = 891), InCHIANTI (n = 611), Rotterdam Study (n = 762), Fehrmann (n = 1,469), HVH (n = 106), SHIP-TREND (n = 963) and DILGOM (n = 509). Cohorts used various genotyping platforms, with imputation performed using the HapMap 2 CEU reference population. Illumina whole-genome Expression BeadChips (HT12v3, HT12v4 or H8v2 arrays) were used to obtain gene expression. Gene expression data were then quantile normalized, $\log_2$ transformed, and mapped for cis-eQTLs and trans-eQTLs. Genome-wide significant threshold was set using 5% FDR.

BIOS (CODAM, LLD, LLS, RS)

Details of the study population and methods have been previously described [74,75] (see also http://www.genenetwork.nl/biosqtlbrowser/). Briefly, eQTL analyses included 2,116 peripheral blood samples from 4 cohorts: CODAM (n=184), LLD (n=626), LLS (n=654), RS (n=652). mQTL analyses included 2,111 samples. Cohorts used various genotyping platforms, with Imputation performed using the GoNL reference panel in IMPUTE2. Gene, exon, exon ratio and poly(A) ratio expression levels were performed using Ensembl v.71 annotation. Genome-wide DNA methylation data were generated using Illumina 450k array. Gene and exon level expression data were quantile normalized and $\log_2$ transformed. Expression data were mapped for cis-eQTLs, cis-mQTLS and trans-mQTLS. Genome-wide significant threshold was set using Benjamini-Hochberg 5% FDR.

FHS

Details of the study population and methods have been previously described [76]. Briefly, eQTL analyses were conducted in whole blood from 5257 individuals participating in the Framingham Heart Study (2240 from the Offspring Cohort and 3017 from the Generation 3 cohort). Genotyping was performed using the Affymetrix 500K and MIPS 50K platforms, and imputation was performed with MACH/Minimac software based on the 1000G “cosmopolitan” SNP set. Gene expression was performed using the Affymetrix Human Exon 1.0 ST microarray. Gene expression data were $\log_2$ transformed, normalized, and mapped for cis-eQTLs and trans-eQTLS. The cis-eQTLs were identified using 2MB window, 1MB before and after transcription start sites of the corresponding genes. Genome-wide significant threshold was set using 5% FDR.

mQTLs were performed in 4100 FHS offspring and the third generation samples whose DNA methylation data and genotype data were both available (unpublished). DNA methylation were measured in whole blood using Ilummina Infinium Human DNA methylation 450K platform. DNA methylation was

normalized as described before [77]. Genotype data were used 1000g with MAF>0.01 and imputation ratio >0.3. Linear mixed models were used to calculate the pairwise associations between CpGs and SNPs adjusting for age, sex, cell types, top 50 PCs in the DNA methylation, and familiar relatedness. Cis-mQTLs were defined as SNPs residing within +/- 1MB on either side of the corresponding CpGs. The significant threshold used was Bonferroni corrected P<0.05.

GTEx

Details of the GTEx data are available online at https://www.gtexportal.org/home/documentationPage. Briefly, we used eQTL data from 278 lung samples. Genotyping data used for eQTL analysis was based on the Illumina OMNI 5M SNP Array. Imputation was based on the 1000 Genomes Project Phase I, version 3. Expression data were generated using the Affymetrix Expression Array and Illumina TrueSeq RNA sequencing. Genome-wide significant threshold was set using 5% FDR.

The Genotype-Tissue Expression (GTEx) Project was supported by the Common Fund of the Office of the Director of the National Institutes of Health, and by NCI, NHGRI, NHLBI, NIDA, NIMH, and NINDS. The data (version 6) used for the analyses described in this manuscript were obtained from the GTEx Portal on 07/15/2016.

SAPPHIRE

Details of the study population and methods have been previously described above. RNA was isolated from whole blood originally collected in PAXgene tubes (PreAnalytiX GmbH, Switzerland). Sequencing libraries were constructed using TruSeq Stranded Total RNA Library Prep Kit with Ribo-Zero Globin (Illumina, San Diego, CA). Next generation sequencing was performed on an Illumina HiSeq. The HiSeq reads were mapped to human genome using HISAT2 and quantified at the gene levels using Stingtie; read counts were normalized using DESeq2 [78]. Genes with zero read counts in over 50% of individuals were excluded from the analysis, leaving 14139 protein-coding genes available for analysis. RNA-sequence data was available for 597 individuals (408 asthma cases and 189 healthy controls). To control for hidden batch effects, we performed factor analysis on normalized gene expression using the software program PEER [79].

We used linear regression and the MatrixeQTL software to model the relationship between the genotype of each SNP (coded 0, 1, and 2) and gene expression [80]. Cis-eQTLs were defined as associations with genes within 1MB of the SNP, whereas trans-eQTLs were SNP associations with genes located >1Mb away on the same chromosome or with genes located on different chromosomes. The regression models accounted for participant age, sex, BMI, smoking status, batch ID from RNAseq, the counts of five white blood cell types (i.e., lymphocytes, monocytes, neutrophils, eosinophils, and basophils), the top 3 PCs, and 60 PEER factors. Separate analyses were performed for the controls, asthma cases, and both groups combined (combined also adjusted for case/control status).

MESA

Details of the study population and methods have been previously described above. Details of eQTL analyses are available in [81]. Briefly, we used Matrix eQTL [80] to perform a genome-wide cis-eQTL analysis in each population separately (AFA, HIS, CAU), in the AFA and HIS combined (AFHI), and in all three populations combined (ALL). We used SNPs with MAF > 0.05 and defined cis-acting as SNPs within 1 Mb of the transcription start site (TSS). The linear regression models included 10 genotype principal

component covariates and a range of PEER factors (0, 10, 20, 30, 50, or 100) [79]. The false discovery rate (FDR) for each SNP was calculated using the Benjamini-Hochberg procedure.

Supplementary Note 2

Ancestry-Specific Meta-Analyses Sensitivity Analyses

European ancestry

Since HCS imputed to the 1000 Genomes European phase 1 reference panel (rather than the Integrated reference panel), we conducted a sensitivity analysis by excluding this study from the European ancestry meta-analysis. Results were not materially changed; correlation between the betas from the main and sensitivity analyses were 0.99 or more for each phenotype.

Since ALHS is a case-control study of asthma nested within the AHS cohort, we conducted a sensitivity analysis by excluding this study from the European ancestry meta-analysis. Results were not materially changed; correlations between the betas from the main and sensitivity analyses were 0.99 or higher for each phenotype. Further, Forest plots (Supplementary Figure 2) do not suggest that results were driven by this study.

Since GOYA is a case-control study of obesity and NEO is a cohort study which oversampled individuals with obesity, we conducted a sensitivity analysis by excluding these studies from the European ancestry meta-analysis. Results were not substantially changed; correlation between the betas from the main and sensitivity analyses were 0.96 or higher for each phenotype. Further, Forest plots (Supplementary Figure 2) do not suggest results were driven by these studies.

African ancestry

Given the admixture in the non-European population from PELTOAS, the only non-US study included in the African ancestry meta-analysis, we also conducted a sensitivity analysis by excluding this study. The correlation between the main African ancestry meta-analysis betas and sensitivity analysis betas was 0.91 or more for each phenotype and top results were reassuringly similar between the main and sensitivity meta-analysis. For example, the top finding was rs11748173 (*ANKRD55/MAP3K1*) for FEV_1, rs114962105 (*EN1/MARCO*) for FVC, rs139215025 (*C2orf48/HPCAL1*) for FEV_1/FVC whether including PELOTAS or not in the African ancestry meta-analysis.

Supplementary Note 3

Meta-Analysis Study Funding and Acknowledgements

AGES

This study has been funded by NIH contract N01-AG-1-2100, the NIA Intramural Research Program, Hjartavernd (the Icelandic Heart Association), and the Althingi (the Icelandic Parliament). The study is approved by the Icelandic National Bioethics Committee, VSN: 00-063. The researchers are indebted to the participants for their willingness to participate in the study.

ALHS

This work was supported by the intramural research program of the NIH, National Institute of Environmental Health Sciences (NIEHS) (Z01-ES049030 and ZO1-ES102385) and National Cancer Institute (Z01-CP010119). This work was also supported in part by American Recovery and Reinvestment Act (ARRA) funds through NIEHS contract number NO1-ES-55546. We thank the numerous study staff at Social & Scientific Systems, Inc. who played a role in the data collection. We acknowledge Dr. Nathan Gaddis, RTI, International for performing the 1000 Genomes imputation.

ARIC

The Atherosclerosis Risk in Communities Study is carried out as a collaborative study supported by National Heart, Lung, and Blood Institute contracts (HHSN268201100005C, HHSN268201100006C, HHSN268201100007C, HHSN268201100008C, HHSN268201100009C, HHSN268201100010C, HHSN268201100011C, and HHSN268201100012C), R01HL087641, R01HL59367 and R01HL086694; National Human Genome Research Institute contract U01HG004402; and National Institutes of Health contract HHSN268200625226C. The authors thank the staff and participants of the ARIC study for their important contributions. Infrastructure was partly supported by Grant Number UL1RR025005, a component of the National Institutes of Health and NIH Roadmap for Medical Research. This work was also supported in part by the Intramural Research Program of the NIH, National Institute of Environmental Health Sciences.

CARDIA

The CARDIA Study is conducted and supported by the National Heart, Lung, and Blood Institute in collaboration with the University of Alabama at Birmingham (HHSN268201300025C & HHSN268201300026C), Northwestern University (HHSN268201300027C), University of Minnesota (HHSN268201300028C), Kaiser Foundation Research Institute (HHSN268201300029C), and Johns Hopkins University School of Medicine (HHSN268200900041C). CARDIA is also partially supported by the Intramural Research Program of the National Institute on Aging. Genotyping was funded as part of the NHLBI Candidate-gene Association Resource (N01-HC-65226) and the NHGRI Gene Environment Association Studies (GENEVA) (U01-HG004729, U01-HG04424, and U01-HG004446). This manuscript has been reviewed and approved by CARDIA for scientific content.

CHS

This CHS research was supported by NHLBI contracts HHSN268201200036C, HHSN268200800007C, HHSN268200960009C, N01HC55222, N01HC85079, N01HC85080, N01HC85081, N01HC85082, N01HC85083, N01HC85086; and NHLBI grants U01HL080295, R01HL087652, R01HL105756, R01HL103612, R01HL120393, R01HL130114, and R01HL085251 with additional contribution from the National Institute of Neurological Disorders and Stroke (NINDS). Additional support was provided through R01AG023629 from the National Institute on Aging (NIA). A full list of principal CHS investigators and institutions can be found at CHS-NHLBI.org. The provision of genotyping data was supported in part by the National Center for Advancing Translational Sciences, CTSI grant UL1TR000124, and the National Institute of Diabetes and Digestive and Kidney Disease Diabetes Research Center (DRC) grant DK063491 to the Southern California Diabetes Endocrinology Research Center. The content is solely the responsibility of the authors and does not necessarily represent the official views of the National Institutes of Health.

FamHS

Support was provided by the National Heart, Lung and Blood Institute grants 5R01HL08770003 and R01HL117078.

FHS

This research was conducted in part using data and resources from the Framingham Heart Study of the Boston University School of Medicine and National Heart Lung and Blood Institute of the National Institutes of Health (contract number N01-HC-25195), and its contract with Affymetrix, Inc for genotyping services (contract number N02-HL-6-4278). The analyses reflect intellectual input and resource development from the Framingham Heart Study investigators participating in the SNP Health Association Resource (SHARe) project. Also supported by NIH P01 AI050516.

GOYA Males

This study was conducted as part of the activities of the Gene-diet Interactions in Obesity project (GENDINOB, www.gendinob.dk) and the MRC centre for Causal Analyses in Translational Epidemiology (MRC CAiTE). We thank all the participants of the study. TSA received his Post-Doctoral Research Grant from the GENDINOB project and acknowledges the same.

HCHS/SOL

The Hispanic Community Health Study/Study of Latinos was carried out as a collaborative study supported by contracts from the National Heart, Lung, and Blood Institute (NHLBI) to the University of North Carolina (N01-HC65233), University of Miami (N01-HC65234), Albert Einstein College of Medicine (N01-HC65235), Northwestern University (N01-HC65236), and San Diego State University (N01-HC65237). The following Institutes/Centers/Offices contribute to the HCHS/SOL through a transfer of funds to the NHLBI: National Institute on Minority Health and Health Disparities, National Institute on Deafness and Other Communication Disorders, National Institute of Dental and Craniofacial Research, National Institute of Diabetes and Digestive and Kidney Diseases, National Institute of Neurological Disorders and Stroke, NIH Institution-Office of Dietary Supplements. The Genetic Analysis Center at the University of Washington was supported by NHLBI and NIDCR contracts (HHSN268201300005C AM03

and MOD03).

Health ABC

The Health Aging and Body Composition cohort study was supported by NIA contracts N01AG62101, N01AG2103, and N01AG62106, NIA grant R01-AG028050, NINR grant R01-NR012459, and in part by the Intramural Research Program of the NIA, NIH. The genome-wide association study was **funded** by NIA grant 1R01AG032098–01A1 to Wake Forest Health Sciences, and genotyping services were provided by the Center for Inherited Disease Research, which is fully funded through a federal contract from the National Institutes of Health to The Johns Hopkins University, contract number HHSN268200782096C. This research was further supported by RC1AG035835.

HCS

The authors would like to thank the men and women participating in the HCS as well as The University of Newcastle, Vincent Fairfax Family Foundation and The Hunter Medical Research Institute.

KARE3 and Healthy Twin Study

This work was funded by the Consortium for Large Scale Genome Wide Association Study III (2011E7300400), which was supported by the genotyping data (the Korean Genome Analysis Project, 4845-301) and the phenotype data (the Korean Genome Epidemiology Study, 4851-302) and a grant from the Centers for Disease Control and Prevention of Korea (budget 2012-E71011-00, 2011-E71011-00, and 2010-E71010-00) and also supported by the Ministry of Science, ICT & Future Planning (2013R1A1A1057961, NRF-2017R1A2B4003790).

JHS

The Jackson Heart Study (JHS) is supported and conducted in collaboration with Jackson State University (HHSN268201300049C and HHSN268201300050C), Tougaloo College (HHSN268201300048C), and the University of Mississippi Medical Center (HHSN268201300046C and HHSN268201300047C) contracts from the National Heart, Lung, and Blood Institute (NHLBI) and the National Institute for Minority Health and Health Disparities (NIMHD). Dr. Wilson is supported by U54GM115428 from the National Institute of General Medical Sciences. The authors also wish to thank the staffs and participants of the JHS. The views expressed in this manuscript are those of the authors and do not necessarily represent the views of the National Heart, Lung, and Blood Institute; the National Institutes of Health; or the U.S. Department of Health and Human Services.

LifeLines

The LifeLines cohort study was supported by the Dutch Ministry of Health, Welfare and Sport, the Ministry of Economic Affairs, Agriculture and Innovation, the province of Groningen, the European Union (regional development fund), the Northern Netherlands Provinces (SNN), the Netherlands Organisation for Scientific Research (NWO), University Medical Center Groningen (UMCG), University of Groningen, de Nierstichting (the Dutch Kidney Foundation), and the Diabetes Fonds (the Diabetic Foundation).

LLFS

The Long Life Family Study (LLFS) was supported by the National Institute on Aging (NIA) grants U01-AG023712, U01-AG023744, U01AG023746, U01-AG023749, and U01-AG023755. The investigators would like to thank the LLFS participants and staff for their valuable contributions.

MESA

MESA and the MESA SHARe project are conducted and supported by the National Heart, Lung, and Blood Institute (NHLBI) in collaboration with MESA investigators. Support for MESA is provided by contracts HHSN268201500003I, N01-HC-95159, N01-HC-95160, N01-HC-95161, N01-HC-95162, N01-HC-95163, N01-HC-95164, N01-HC-95165, N01-HC-95166, N01-HC-95167, N01-HC-95168, N01-HC-95169, UL1-TR-000040, UL1-TR-001079, UL1-TR-001420, UL1-TR-001881, and DK063491. MESA Family is conducted and supported by the National Heart, Lung, and Blood Institute (NHLBI) in collaboration with MESA investigators. Support is provided by grants and contracts R01HL071051, R01HL071205, R01HL071250, R01HL071251, R01HL071258, R01HL071259, by the National Center for Research Resources, Grant UL1RR033176, and the National Center for Advancing Translational Sciences, Grant UL1TR001881. The MESA Lung study was supported by grants R01 HL077612, R01 HL093081 and RC1 HL100543 from the NHLBI. This publication was developed under a STAR research assistance agreement, No. RD831697 (MESA Air), awarded by the U.S Environmental protection Agency. It has not been formally reviewed by the EPA. The views expressed in this document are solely those of the authors and the EPA does not endorse any products or commercial services mentioned in this publication. Funding for SHARe genotyping was provided by NHLBI Contract N02-HL-64278. Genotyping was performed at Affymetrix (Santa Clara, California, USA) and the Broad Institute of Harvard and MIT (Boston, Massachusetts, USA) using the Affymetrix Genome-Wide Human SNP Array 6.0. This research was supported by NIH R01HL131565. The MESA Epigenomics and Transcriptomics Studies were funded by R01HL101250, R01 DK103531-01, R01 DK103531, R01 AG054474, and R01 HL135009-01 to Wake Forest University Health Sciences. The MESA eQTL studies were funded by R15 HG009569 to Loyola University Chicago.

NEO

The authors of the NEO study thank all individuals who participated in the Netherlands Epidemiology in Obesity study, all participating general practitioners for inviting eligible participants and all research nurses for collection of the data. We thank the NEO study group, Pat van Beelen, Petra Noordijk and Ingeborg de Jonge for the coordination, lab and data management of the NEO study. The genotyping in the NEO study was supported by the Centre National de Génotypage (Paris, France), headed by Jean-Francois Deleuze. The NEO study is supported by the participating Departments, the Division and the Board of Directors of the Leiden University Medical Center, and by the Leiden University, Research Profile Area Vascular and Regenerative Medicine. Dennis Mook-Kanamori is supported by Dutch Science Organization (ZonMW-VENI Grant 916.14.023).

1982 Pelotas (Brazil) Birth Cohort

The 1982 Pelotas Birth Cohort Study is conducted by the Postgraduate Program in Epidemiology at Universidade Federal de Pelotas with the collaboration of the Brazilian Public Health Association (ABRASCO). From 2004 to 2013, the Wellcome Trust supported the study. The International Development Research Center, World Health Organization, Overseas Development Administration,

European Union, National Support Program for Centers of Excellence (PRONEX), the Brazilian National Research Council (CNPq), and the Brazilian Ministry of Health supported previous phases of the study. Genotyping was supported by the Department of Science and Technology (DECIT, Ministry of Health) and National Fund for Scientific and Technological Development (FNDCT, Ministry of Science and Technology), Funding of Studies and Projects (FINEP, Ministry of Science and Technology, Brazil), Coordination of Improvement of Higher Education Personnel (CAPES, Ministry of Education, Brazil).

RS I, II & III

The Rotterdam Study is funded by Erasmus Medical Center and Erasmus University, Rotterdam, Netherlands Organization for the Health Research and Development (ZonMw), the Research Institute for Diseases in the Elderly (RIDE), the Ministry of Education, Culture and Science, the Ministry for Health, Welfare and Sports, the European Commission (DG XII), and the Municipality of Rotterdam.

The generation and management of GWAS genotype data for the Rotterdam Study (RS I, RS II, RS III) was executed by the Human Genotyping Facility of the Genetic Laboratory of the Department of Internal Medicine, Erasmus MC, Rotterdam, The Netherlands. The GWAS datasets are supported by the Netherlands Organisation of Scientific Research NWO Investments (nr. 175.010.2005.011, 911-03-012), the Genetic Laboratory of the Department of Internal Medicine, Erasmus MC, the Research Institute for Diseases in the Elderly (014-93-015; RIDE2), the Netherlands Genomics Initiative (NGI)/Netherlands Organisation for Scientific Research (NWO) Netherlands Consortium for Healthy Aging (NCHA), project nr. 050-060-810. The generation and management of spirometric data was supported by FWO project G035014N. Lies Lahousse is a Postdoctoral Fellow of the Fund for Scientific Research Foundation - Flanders (FWO).

Replication Study Funding and Acknowledgements

UK BiLEVE

The UK BiLEVE study was funded by a Medical Research Council (MRC) strategic award (MC_PC_12010). The research undertaken by V.E.J. was partly funded by the National Institute for Health Research (NIHR). The views expressed are those of the author(s) and not necessarily those of the NHS, the NIHR or the Department of Health. This research used the ALICE High Performance Computing Facility at the University of Leicester.

COPDGene

The COPDGene project (NCT00608764) was supported by Award Number R01HL089897 and Award Number R01HL089856 from the National Heart, Lung, And Blood Institute. The COPDGene project is also supported by the COPD Foundation through contributions made to an Industry Advisory Board comprised of AstraZeneca, Boehringer Ingelheim, Novartis, Pfizer, Siemens, Sunovion, and GlaxoSmithKline.

COPDGene Investigators

Administrative Core: James Crapo, MD (PI), Edwin Silverman, MD, PhD (PI), Barry Make, MD, Elizabeth Regan, MD, PhD

Genetic Analysis Core: Terri Beaty, PhD, Nan Laird, PhD, Christoph Lange, PhD, Michael Cho, MD, Stephanie Santorico, PhD, John Hokanson, MPH, PhD, Dawn DeMeo, MD, MPH, Nadia Hansel, MD, MPH, Craig Hersh, MD, MPH, Peter Castaldi, MD, MSc, Merry-Lynn McDonald, PhD, Emily Wan, MD, Megan Hardin, MD, Jacqueline Hetmanski, MS, Margaret Parker, PhD, Marilyn Foreman, MD, Brian Hobbs, MD, MMSc, Robert Busch, MD, MMSc, Adel El-Boueiz, MD, MMSc, Dandi Qiao, PhD, Elizabeth Regan, MD, Eitan Halper-Stromberg, Ferdouse Begum, Sungho Won, Sharon Lutz, PhD
Imaging Core: David A Lynch, MB, Harvey O Coxson, PhD, MeiLan K Han, MD, MS, MD, Eric A Hoffman, PhD, Stephen Humphries MS, Francine L Jacobson, MD, Philip F Judy, PhD, Ella A Kazerooni, MD, John D Newell, Jr., MD, Elizabeth Regan, MD, James C Ross, PhD, Raul San Jose Estepar, PhD, Berend C Stoel, PhD, Juerg Tschirren, PhD, Eva van Rikxoort, PhD, Bram van Ginneken, PhD, George Washko, MD, Carla G Wilson, MS, Mustafa Al Qaisi, MD, Teresa Gray, Alex Kluiber, Tanya Mann, Jered Sieren, Douglas Stinson, Joyce Schroeder, MD, Edwin Van Beek, MD, PhD
PFT QA Core, Salt Lake City, UT: Robert Jensen, PhD
Data Coordinating Center and Biostatistics, *National Jewish Health, Denver, CO*: Douglas Everett, PhD, Anna Faino, MS, Matt Strand, PhD, Carla Wilson, MS
Epidemiology Core, *University of Colorado Anschutz Medical Campus, Aurora, CO*: John E. Hokanson, MPH, PhD, Gregory Kinney, MPH, PhD, Sharon Lutz, PhD, Kendra Young PhD, Katherine Pratte, MSPH, Lindsey Duca, MS
Ann Arbor VA: Jeffrey L. Curtis, MD, Carlos H. Martinez, MD, MPH, Perry G. Pernicano, MD
Baylor College of Medicine, Houston, TX: Nicola Hanania, MD, MS, Philip Alapat, MD, Venkata Bandi, MD, Mustafa Atik, MD, Aladin Boriek, PhD, Kalpatha Guntupalli, MD, Elizabeth Guy, MD, Amit Parulekar, MD, Arun Nachiappan, MD
Brigham and Women's Hospital, Boston, MA: Dawn DeMeo, MD, MPH, Craig Hersh, MD, MPH, George Washko, MD, Francine Jacobson, MD, MPH
Columbia University, New York, NY: R. Graham Barr, MD, DrPH, Byron Thomashow, MD, John Austin, MD, Belinda D'Souza, MD, Gregory D.N. Pearson, MD, Anna Rozenshtein, MD, MPH, FACR
Duke University Medical Center, Durham, NC: Neil MacIntyre, Jr., MD, Lacey Washington, MD, H. Page McAdams, MD
Health Partners Research Foundation, Minneapolis, MN: Charlene McEvoy, MD, MPH, Joseph Tashjian, MD
Johns Hopkins University, Baltimore, MD: Robert Wise, MD, Nadia Hansel, MD, MPH, Robert Brown, MD, Karen Horton, MD, Nirupama Putcha, MD, MHS,
Los Angeles Biomedical Research Institute at Harbor UCLA Medical Center, Torrance, CA: Richard Casaburi, PhD, MD, Alessandra Adami, PhD, Janos Porszasz, MD, PhD, Hans Fischer, MD, PhD, Matthew Budoff, MD, Harry Rossiter, PhD
Michael E. DeBakey VAMC, Houston, TX: Amir Sharafkhaneh, MD, PhD, Charlie Lan, DO
Minneapolis VA: Christine Wendt, MD, Brian Bell, MD
Morehouse School of Medicine, Atlanta, GA: Marilyn Foreman, MD, MS, Gloria Westney, MD, MS, Eugene Berkowitz, MD, PhD
National Jewish Health, Denver, CO: Russell Bowler, MD, PhD, David Lynch, MD
Reliant Medical Group, Worcester, MA: Richard Rosiello, MD, David Pace, MD
Temple University, Philadelphia, PA: Gerard Criner, MD, David Ciccolella, MD, Francis Cordova, MD, Chandra Dass, MD, Gilbert D'Alonzo, DO, Parag Desai, MD, Michael Jacobs, PharmD, Steven Kelsen, MD, PhD, Victor Kim, MD, A. James Mamary, MD, Nathaniel Marchetti, DO, Aditi Satti, MD, Kartik Shenoy, MD, Robert M. Steiner, MD, Alex Swift, MD, Irene Swift, MD, Maria Elena Vega-Sanchez, MD
University of Alabama, Birmingham, AL: Mark Dransfield, MD, William Bailey, MD, J. Michael Wells, MD, Surya Bhatt, MD, Hrudaya Nath, MD

University of California, San Diego, CA: Joe Ramsdell, MD, Paul Friedman, MD, Xavier Soler, MD, PhD, Andrew Yen, MD
University of Iowa, Iowa City, IA: Alejandro Comellas, MD, John Newell, Jr., MD, Brad Thompson, MD
University of Michigan, Ann Arbor, MI: MeiLan Han, MD, Ella Kazerooni, MD, Carlos Martinez, MD
University of Minnesota, Minneapolis, MN: Joanne Billings, MD, Tadashi Allen, MD
University of Pittsburgh, Pittsburgh, PA: Frank Sciurba, MD, Divay Chandra, MD, MSc, Joel Weissfeld, MD, MPH, Carl Fuhrman, MD, Jessica Bon, MD
University of Texas Health Science Center at San Antonio, San Antonio, TX: Antonio Anzueto, MD, Sandra Adams, MD, Diego Maselli-Caceres, MD, Mario E. Ruiz, MD

SAPPHIRE

The SAPPHIRE cohort was funded through grants from the Fund for Henry Ford Hospital, the American Asthma Foundation, and the following institutes of the U.S. National Institutes of Health: the National Allergy and Infectious Diseases (R01AI079139, R01AI061774), the National Heart Lung and Blood Institute (R01HL118267, R01HL079055), and the National Institute of Diabetes Digestive and Kidney Diseases (R01DK113003, R01DK064695).

SAGE and GALA II

This work was supported in part by the Sandler Family Foundation, the American Asthma Foundation, the RWJF Amos Medical Faculty Development Program, Harry Wm. and Diana V. Hind Distinguished Professor in Pharmaceutical Sciences II, National Institutes of Health 1R01HL117004, R01Hl128439, R01HL135156, 1X01HL134589, National Institute of Health and Environmental Health Sciences R01ES015794, R21ES24844, the National Institute on Minority Health and Health Disparities 1P60MD006902, RL5GM118984, 1R01MD010443 and the Tobacco-Related Disease Research Program under Award Number 24RT-002.

Additional Study-Specific Funding and Acknowledgements

International COPD Genetics Consortium (ICGC) (assessed overlap of PFT loci with COPD loci)

BDH is supported by NIH K08 HL136928 and the Parker B. Francis Research Opportunity Award. M.H.C. was supported by R01HL113264, R01HL137927, and R01HL135142.

ICGC Investigators

Executive Committee: James Crapo, William MacNee, David Lynch, Dirkje Postma, Edwin Silverman, Jorgen Vestbo. Members: Alvar Agusti, Wayne Anderson, Nawar Bakerly, Per Bakke, Robert Bals, Kathleen Barnes, R. Graham Barr, Terri Beaty, Eugene Bleecker, Marike Boezen, Yohan Bosse, Russell Bowler, Christopher Brightling, Marleen de Bruijne, Guy Brusselle, Peter Castaldi, Bartolome Celli, Michael Cho, Harvey Coxson, Ron Crystal, Dawn DeMeo, Asger Dirksen, Jennifer Dy, Marilyn Foreman, Judith Garcia-Aymerich, Pierre Gevenois, Hester Gietema, Ian Hall, Nadia Hansel, Craig Hersh, Eric Hoffman, John Hokanson, Pim de Jong, Phil Judy, Noor Kalsheker, Hans-Ulrich Kauczor, Woo Jin Kim, Lies Lahousse, Tarja Laitinen, Diether Lambrechts, Sang Do Lee, Augusto Litonjua, David Lomas, Stephanie London, Daan Loth, Sharon Lutz, Masaharu Nishimura, John Newell, Merry-Lynn McDonald, Borge Nordestgaard, George O'Connor, Yeon-Mok Oh, Peter Pare, Massimo Pistolesi, Milo Puhan, Elizabeth Regan, Stephen Rennard, Stephen Rich, Andrew Sandford, Joon-Beom Seo, Andrea Short, Martijn Spruit,

Berend Stoel, David Strachan, Gerben ter Riet, Nicola Sverzellati, Yohannes Tesfaigzi, Martin Tobin, Edwin Van Beek, Bram van Ginneken, Claus Vogelmeier, Adam Wanner, George Washko, Els Wauters, Emiel Wouters, Robert Young, Loems Zeigler-Heitbrock

Lung eQTL Consortium (assessed overlap of PFT loci with eQTL signals)

The lung eQTL study at Laval University was supported by the Fondation de l'Institut universitaire de cardiologie et de pneumologie de Québec, the Respiratory Health Network of the FRQS, the Canadian Institutes of Health Research (MOP - 123369). YB holds a Canada Research Chair in Genomics of Heart and Lung Diseases. The authors would like to thank the staff at the Quebec Respiratory Health Network Biobank of the FRQS for their valuable assistance with the lung eQTL dataset.

Supplementary Figures

A.

FEV_1_multiethnic_ancestry_rs12092943

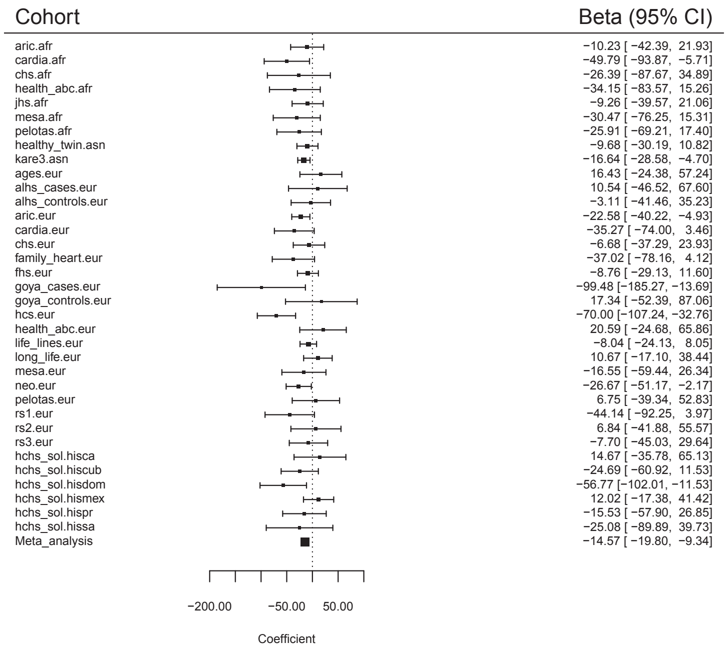

FEV_1_multiethnic_ancestry_1:221765779:C_CA

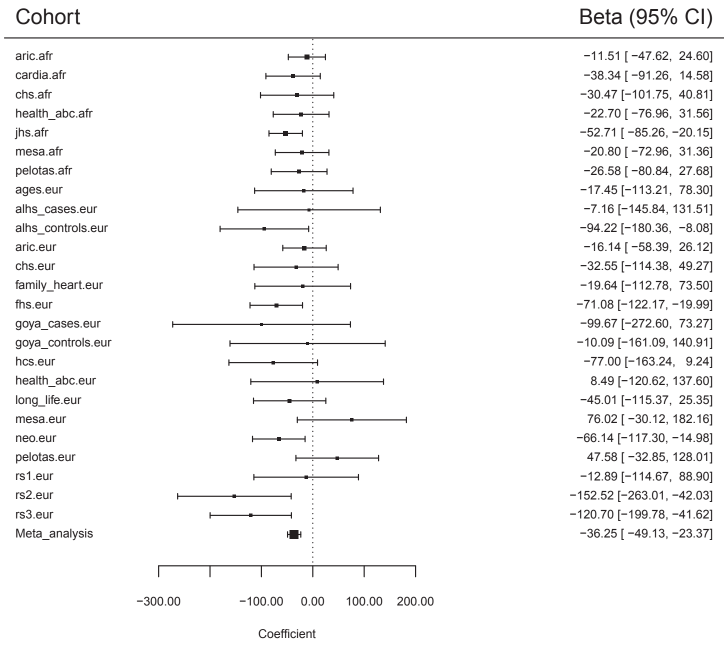

FEV_1_multiethnic_ancestry_rs963406

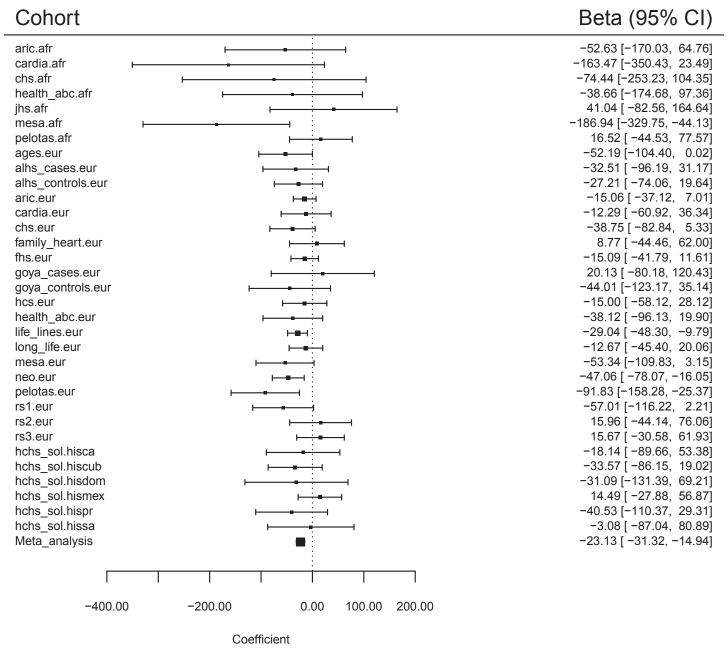

FEV_1_multiethnic_ancestry_rs79294353

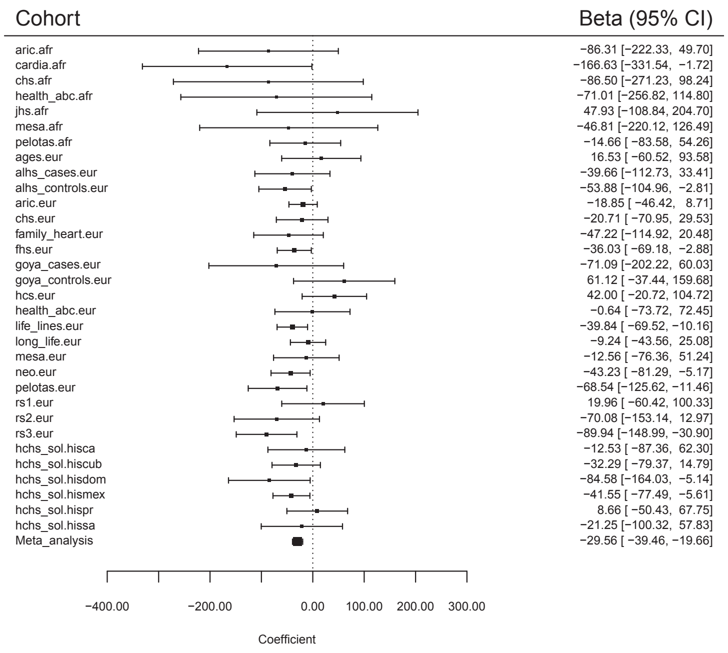

FEV$_1$_multiethnic_ancestry_rs6778584

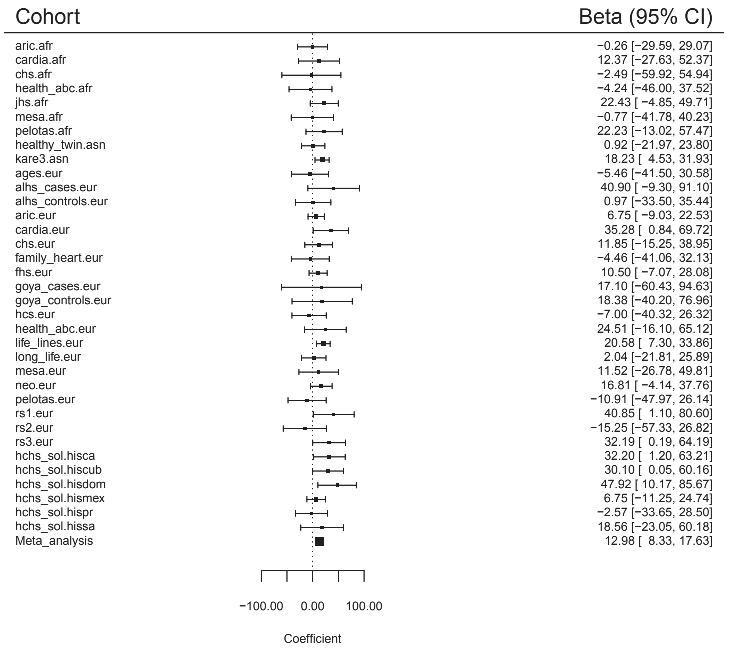

FEV$_1$_multiethnic_ancestry_rs111898810

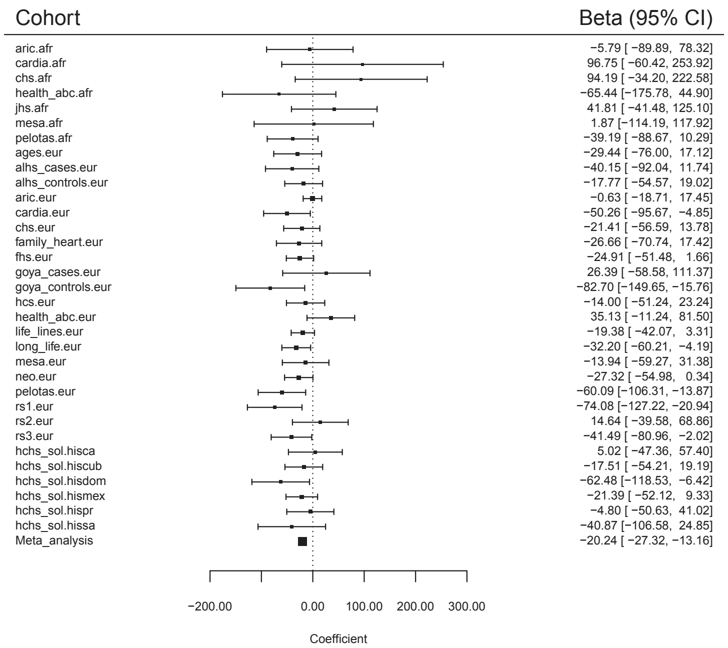

FEV$_1$_multiethnic_ancestry_rs9407640

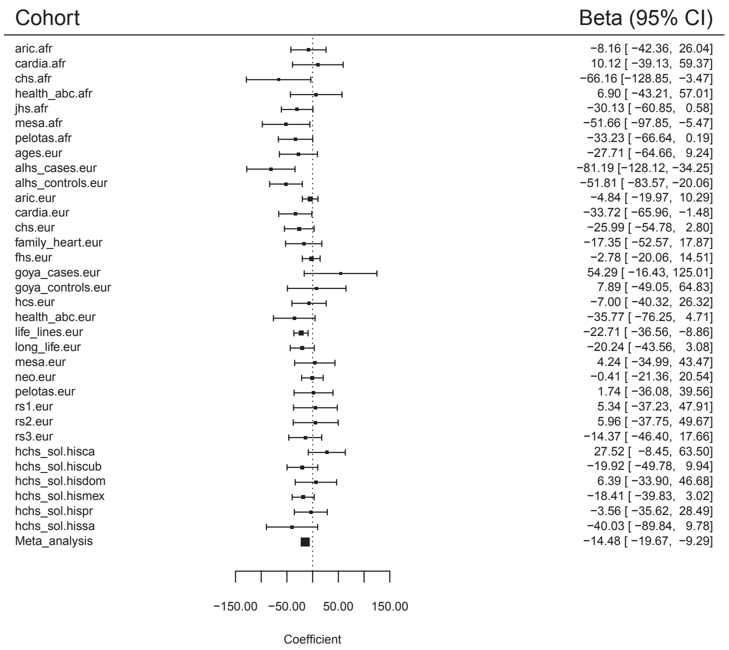

FEV$_1$_multiethnic_ancestry_rs7899503

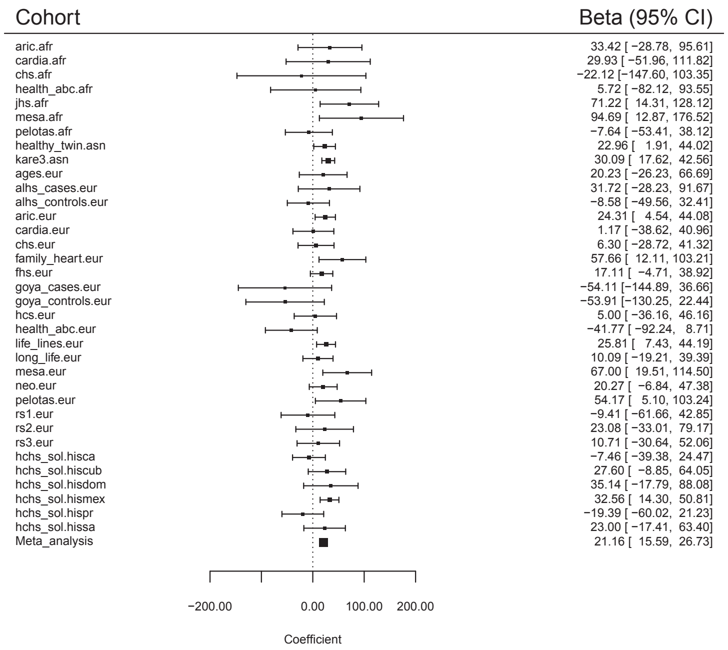

FEV$_1$_multiethnic_ancestry_rs772920

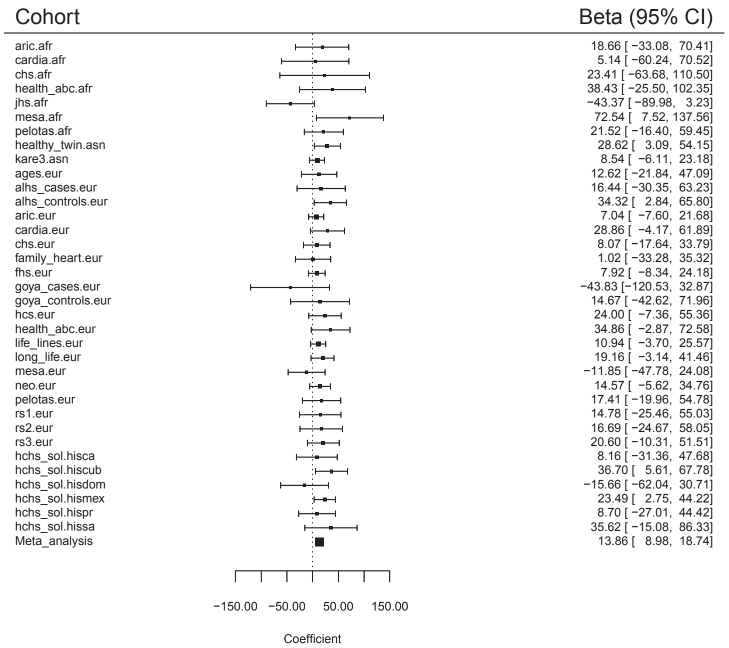

FEV$_1$_multiethnic_ancestry_rs11057793

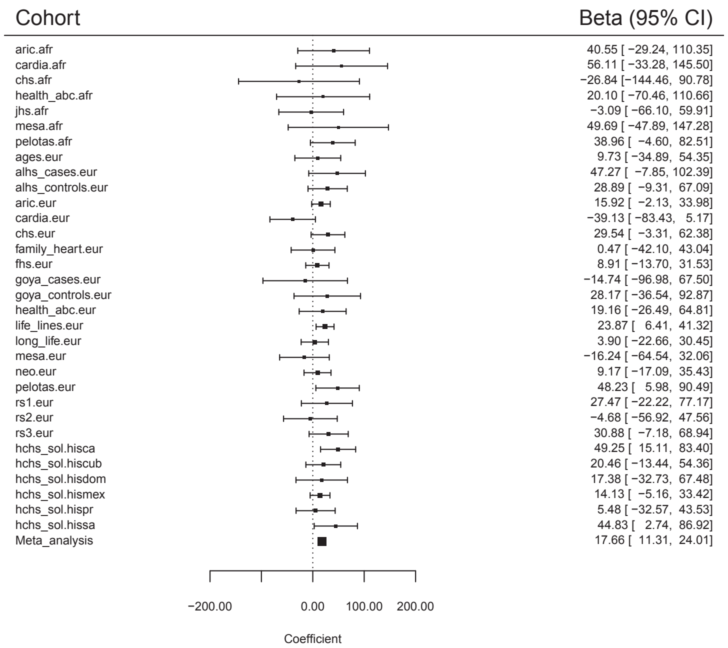

FEV$_1$_multiethnic_ancestry_rs62070631

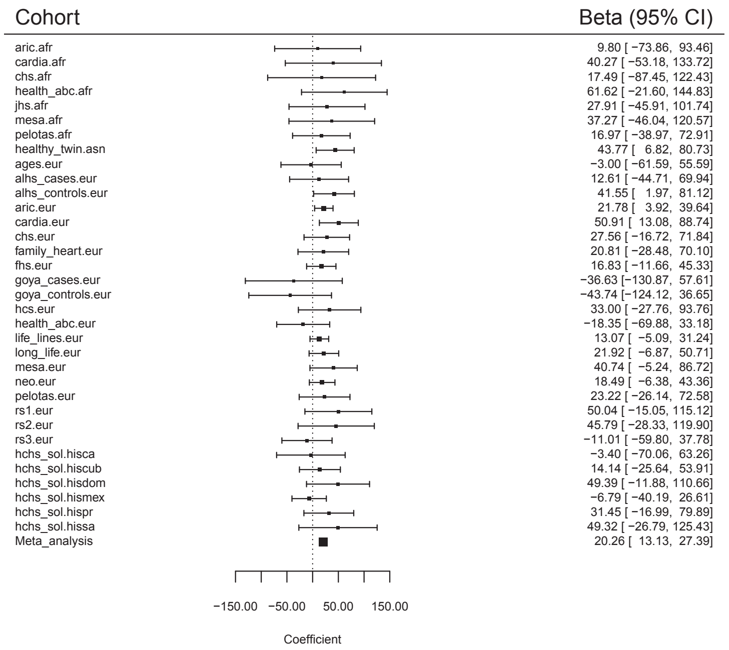

FEV$_1$_multiethnic_ancestry_rs186806998

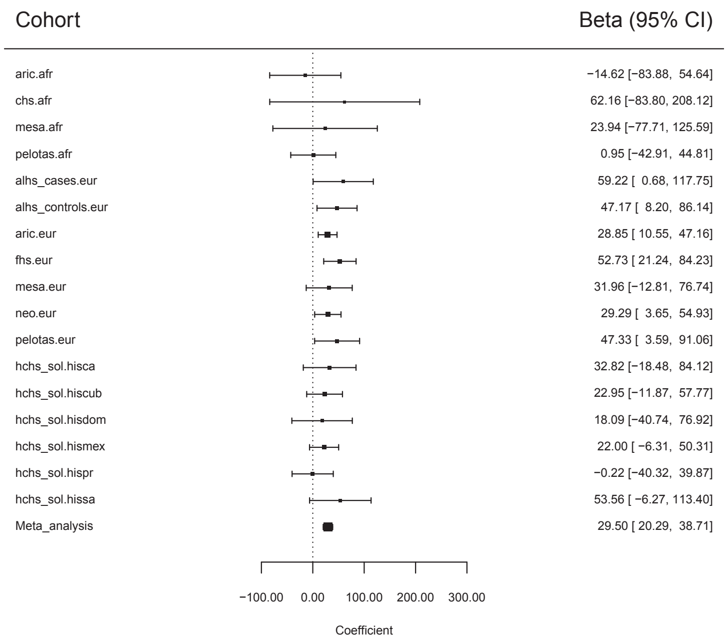

FEV_1_multiethnic_ancestry_rs199525

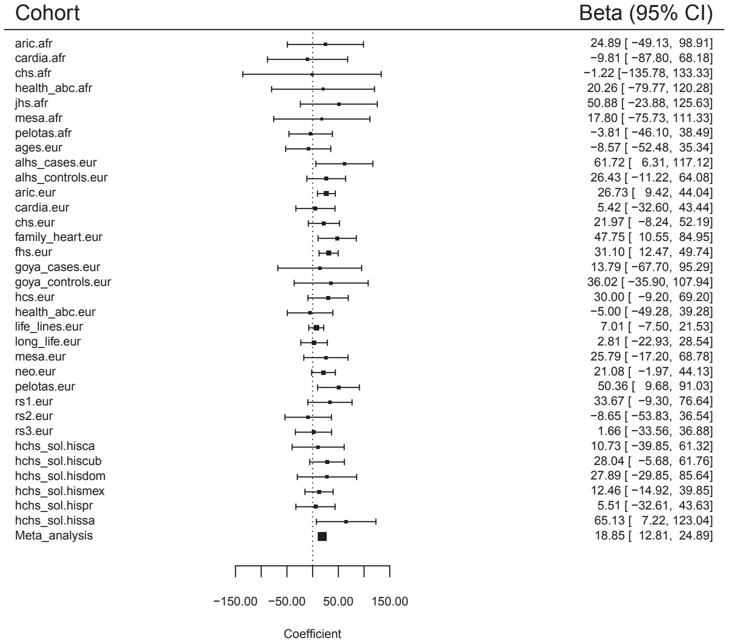

FEV_1_multiethnic_ancestry_rs513953

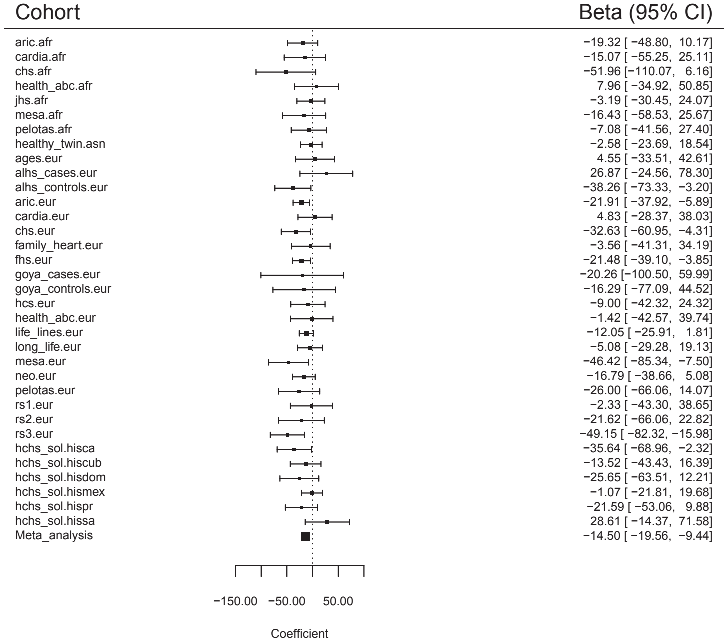

FEV_1_multiethnic_ancestry_rs7243351

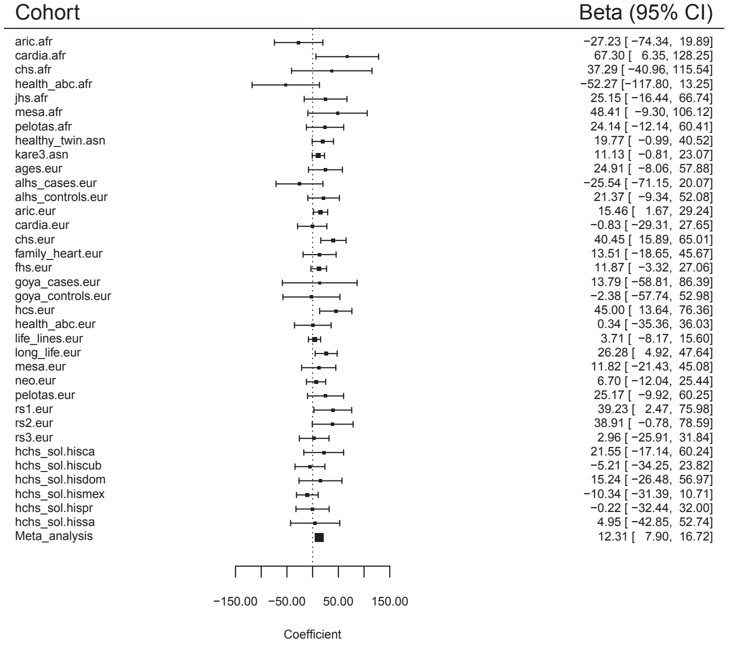

FEV_1_multiethnic_ancestry_rs6138639

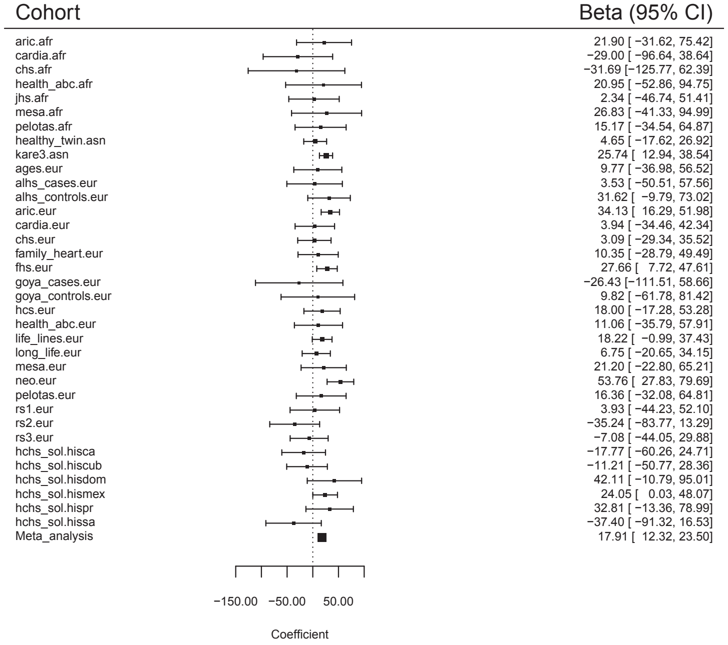

FEV_1_multiethnic_ancestry_rs1737889

Cohort	Beta (95% CI)
aric.afr	−9.79 [−43.49, 23.90]
cardia.afr	−25.98 [−75.18, 23.22]
chs.afr	14.15 [−53.61, 81.90]
health_abc.afr	27.62 [−20.96, 76.20]
jhs.afr	−14.67 [−45.67, 16.33]
mesa.afr	19.31 [−28.68, 67.29]
pelotas.afr	−47.82 [−86.64, −8.99]
ages.eur	−23.75 [−70.99, 23.50]
alhs_cases.eur	−83.30 [−138.34, −28.25]
alhs_controls.eur	−15.22 [−52.40, 21.96]
aric.eur	−4.01 [−22.41, 14.39]
cardia.eur	−30.15 [−70.60, 10.30]
chs.eur	−11.68 [−44.38, 21.01]
family_heart.eur	−18.59 [−59.96, 22.78]
fhs.eur	−25.24 [−50.06, −0.42]
goya_cases.eur	21.23 [−61.92, 104.38]
goya_controls.eur	−14.27 [−82.33, 53.78]
hcs.eur	9.00 [−32.16, 50.16]
health_abc.eur	−17.56 [−64.56, 29.44]
life_lines.eur	−23.21 [−38.23, −8.19]
long_life.eur	−23.95 [−50.59, 2.69]
mesa.eur	−11.74 [−59.34, 35.86]
neo.eur	−21.65 [−48.91, 5.61]
pelotas.eur	3.63 [−39.07, 46.32]
rs1.eur	−32.29 [−81.27, 16.68]
rs2.eur	−35.87 [−88.73, 16.99]
rs3.eur	−28.29 [−67.90, 11.31]
hchs_sol.hisca	−23.00 [−63.25, 17.25]
hchs_sol.hiscub	7.36 [−26.47, 41.19]
hchs_sol.hisdom	5.49 [−37.44, 48.42]
hchs_sol.hismex	−26.05 [−48.33, −3.78]
hchs_sol.hispr	−18.96 [−55.72, 17.81]
hchs_sol.hissa	−23.47 [−74.19, 27.26]
Meta_analysis	−16.82 [−22.84, −10.80]

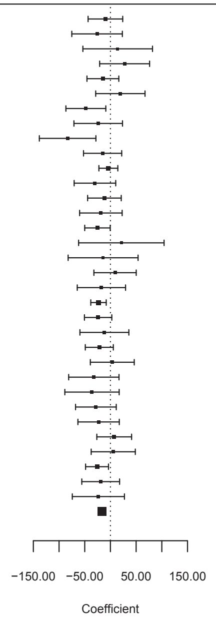

B.

FVC_multiethnic_ancestry_rs2821332

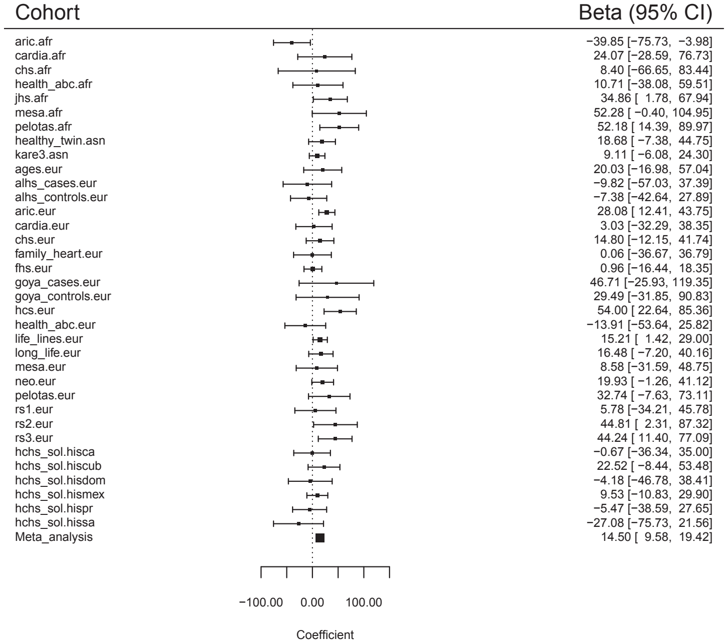

FVC_multiethnic_ancestry_rs12046746

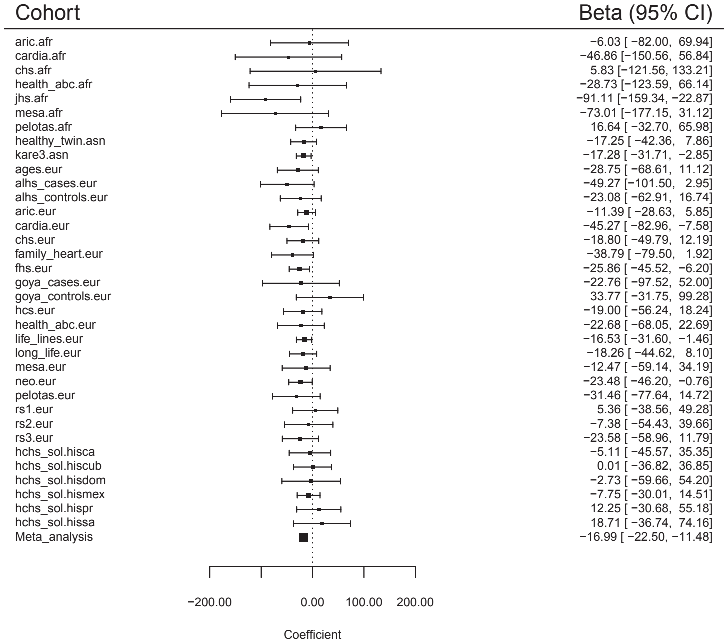

FVC_multiethnic_ancestry_1:237929787:T_TCA

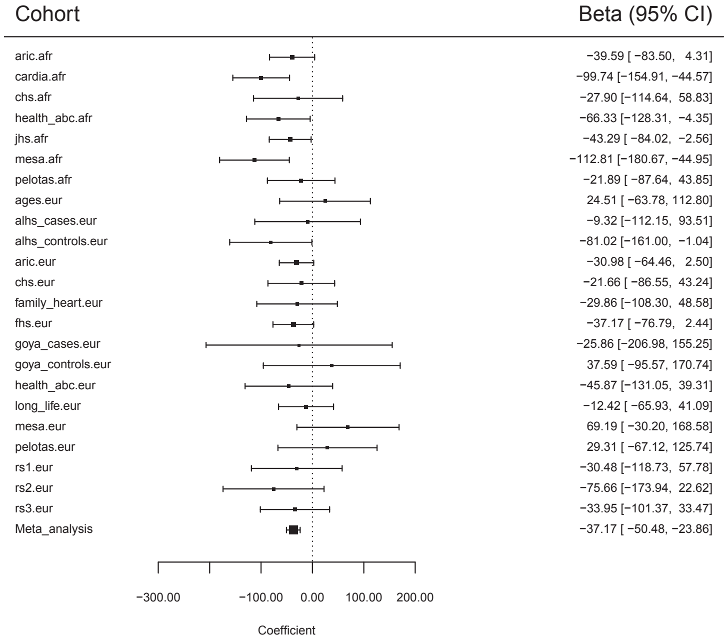

FVC_multiethnic_ancestry_rs17034666

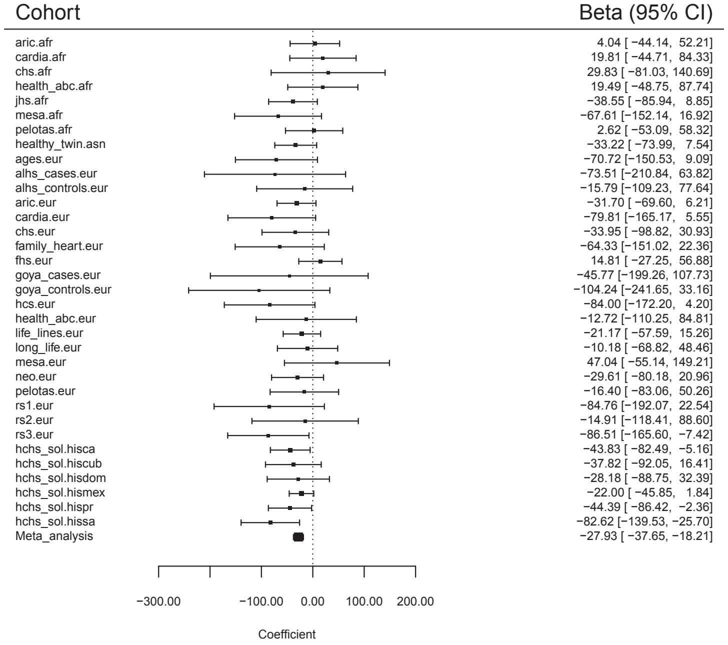

FVC_multiethnic_ancestry_rs1404098

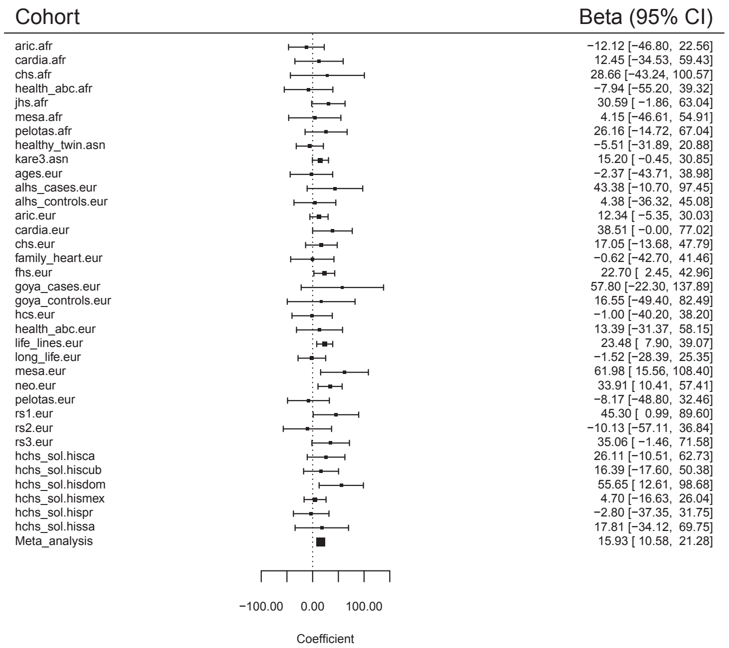

FVC_multiethnic_ancestry_rs72776440

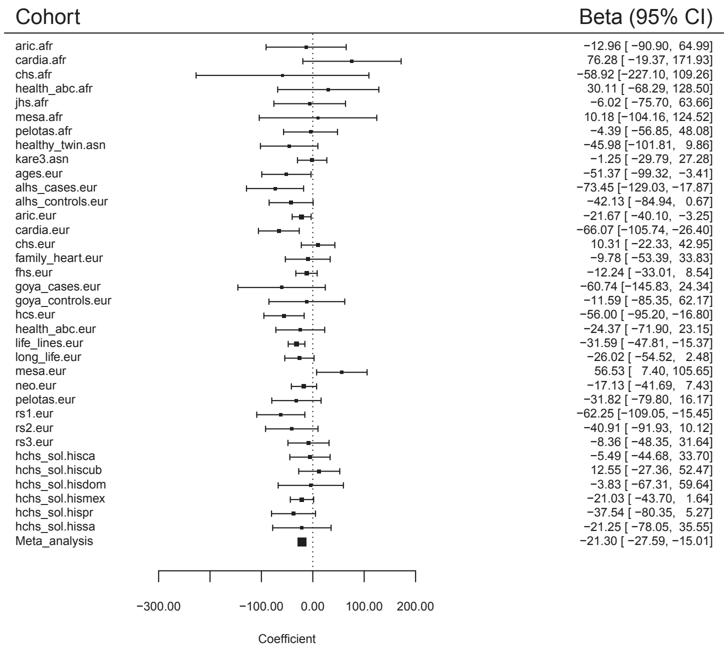

FVC_multiethnic_ancestry_rs11759026

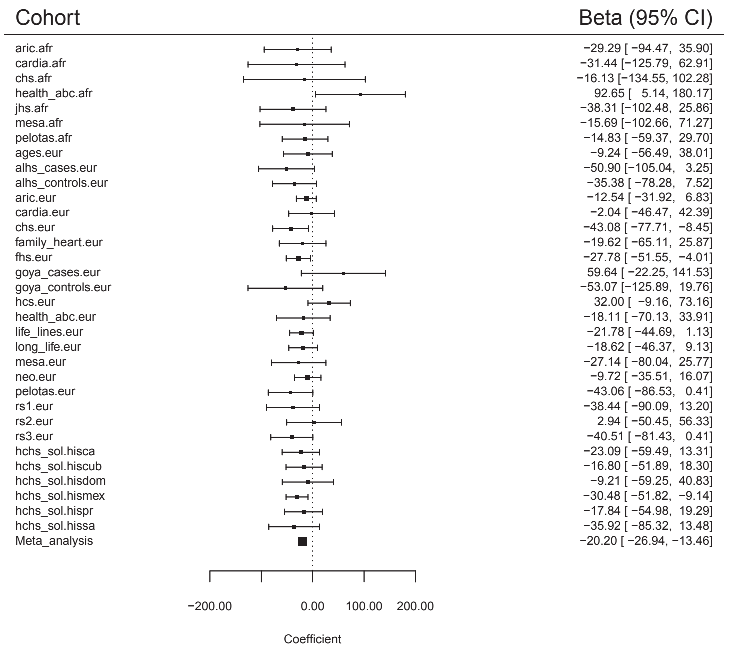

FVC_multiethnic_ancestry_rs55905169

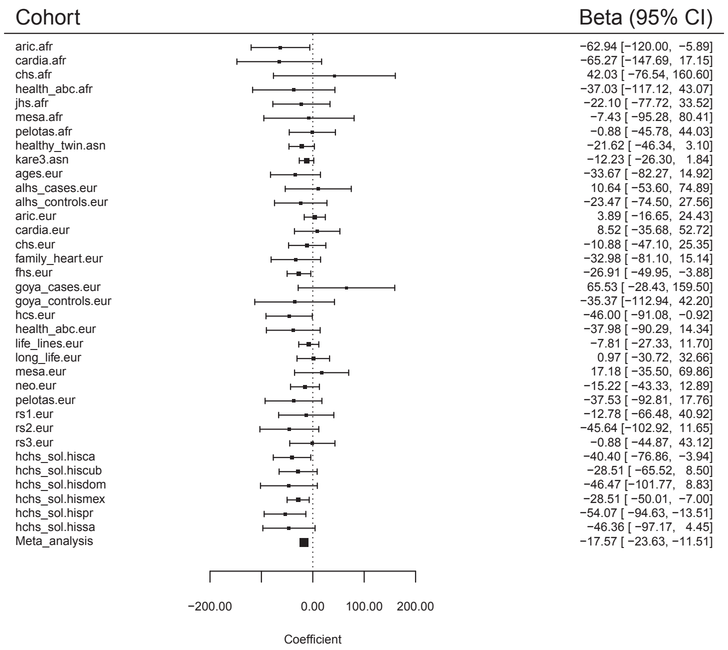

FVC_multiethnic_ancestry_rs9407640

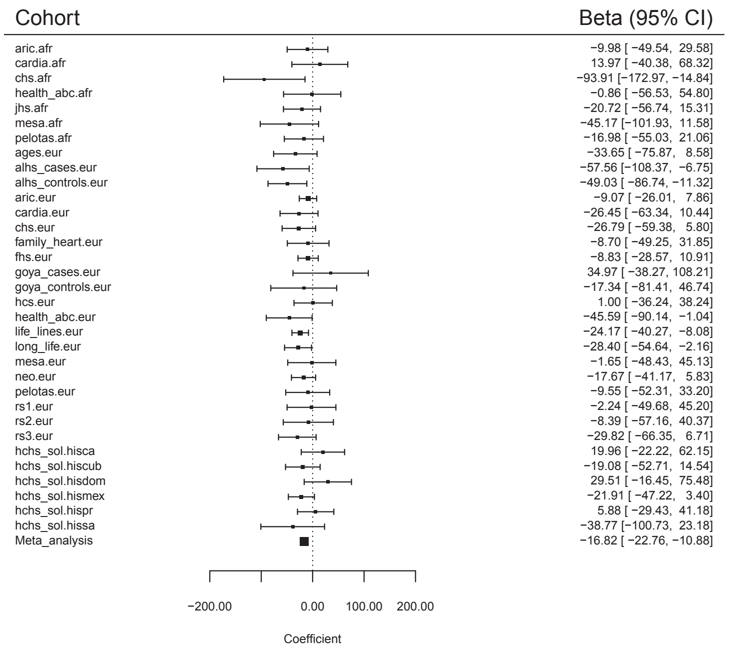

FVC_multiethnic_ancestry_10:77002679:TC_T

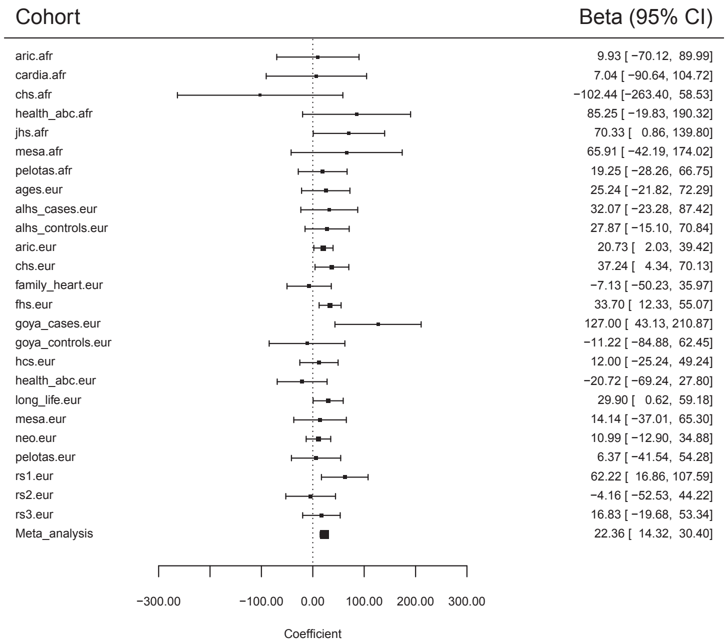

FVC_multiethnic_ancestry_rs73025192

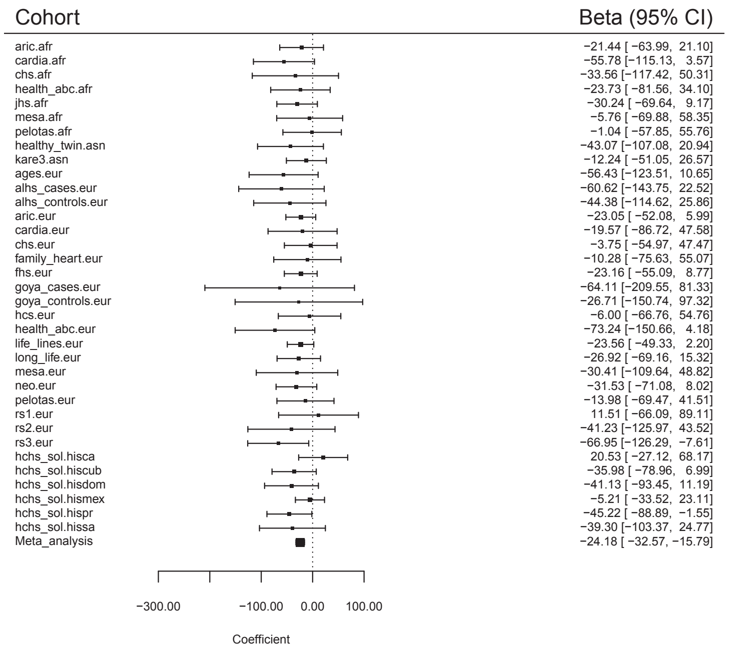

FVC_multiethnic_ancestry_rs7971039

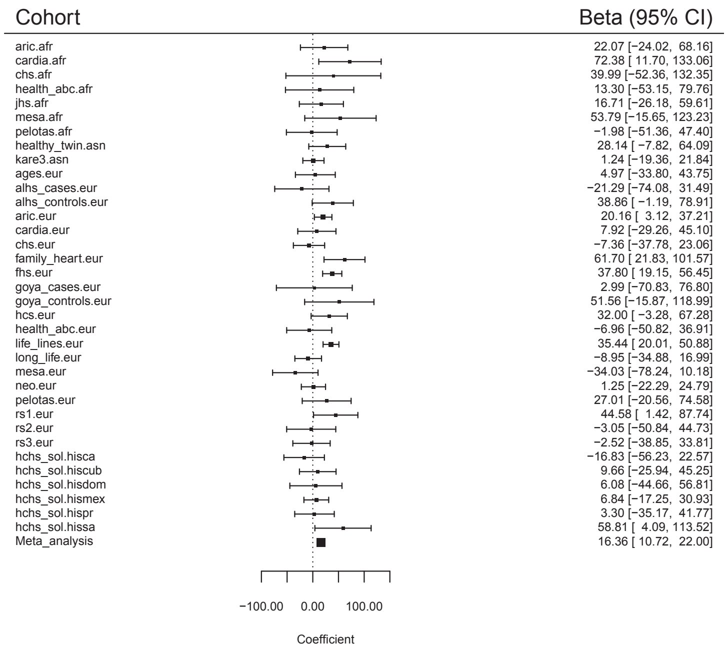

FVC_multiethnic_ancestry_rs11107184

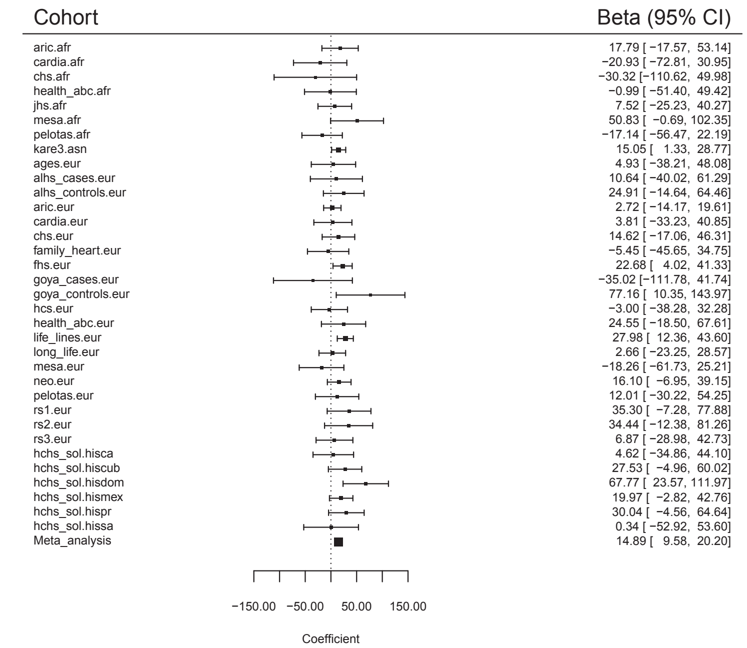

FVC_multiethnic_ancestry_rs10859698

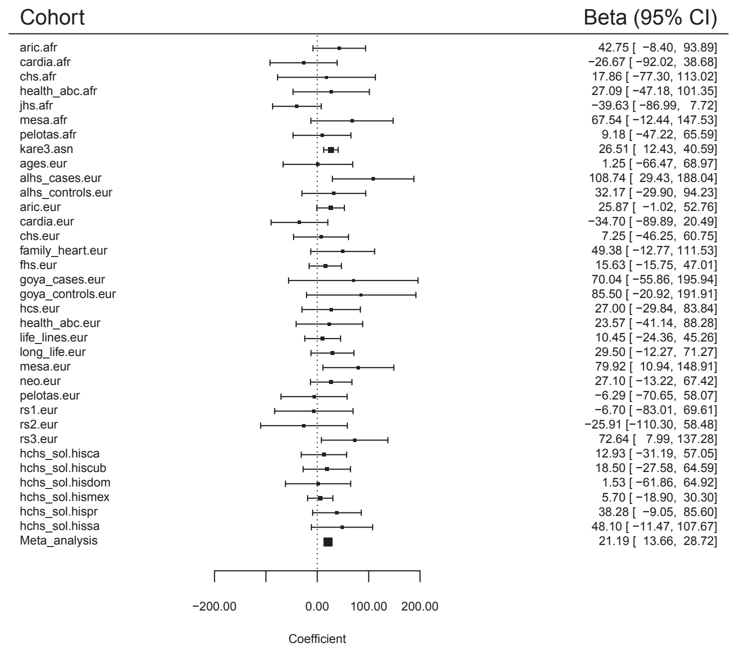

FVC_multiethnic_ancestry_rs4775429

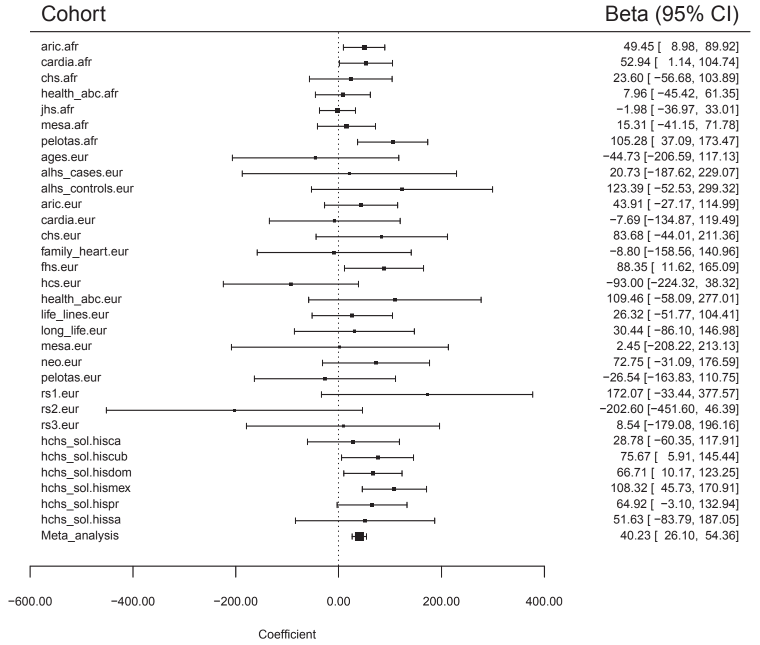

FVC_multiethnic_ancestry_rs8025774

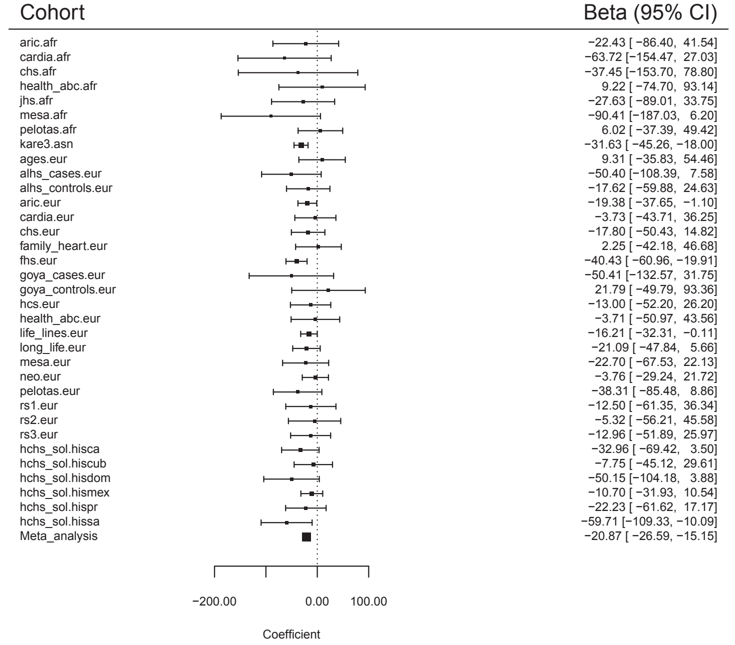

FVC_multiethnic_ancestry_rs3973397

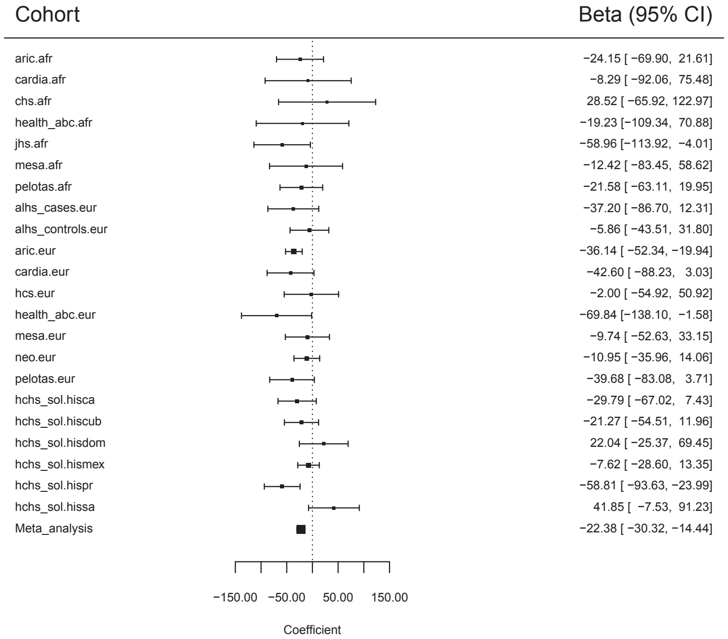

FVC_multiethnic_ancestry_rs55771535

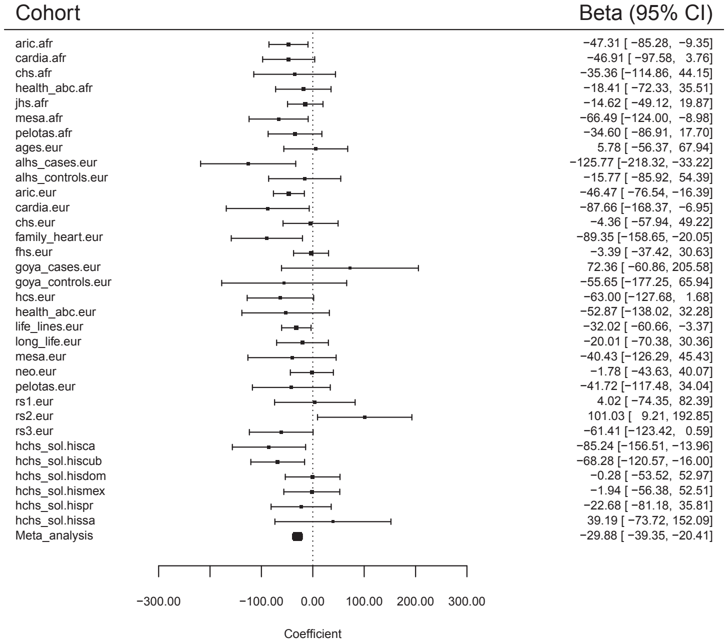

FVC_multiethnic_ancestry_rs8067511

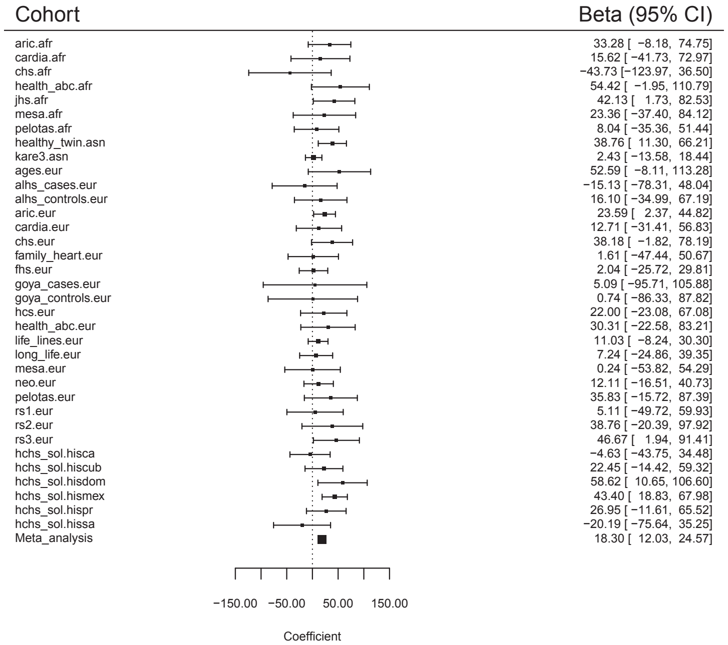

FVC_multiethnic_ancestry_rs150741403

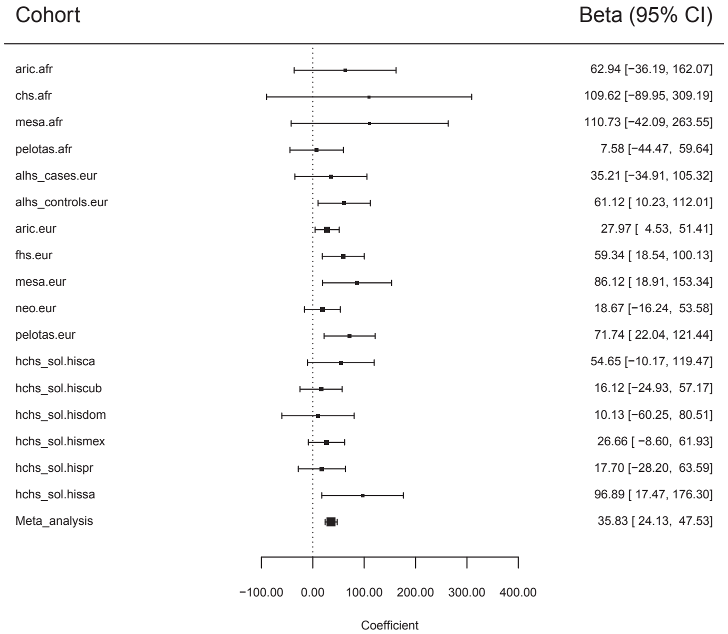

FVC_multiethnic_ancestry_rs199525

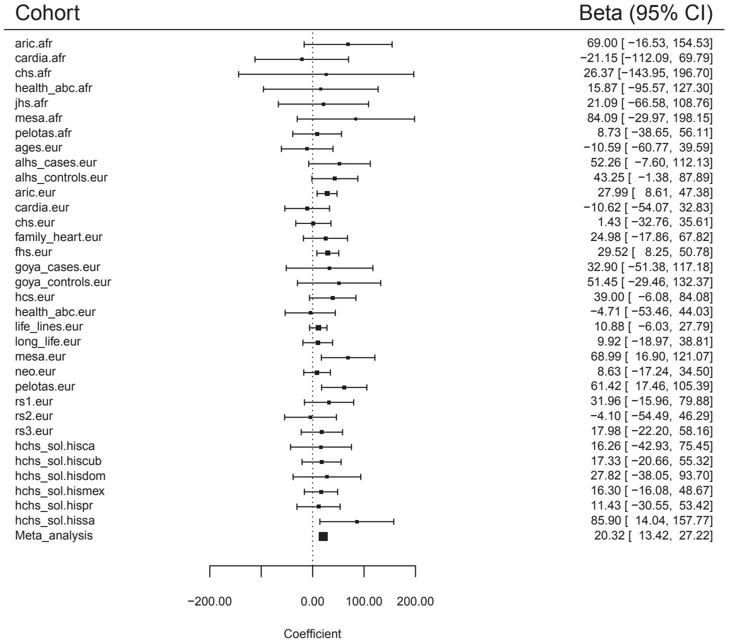

FVC_multiethnic_ancestry_rs7238093

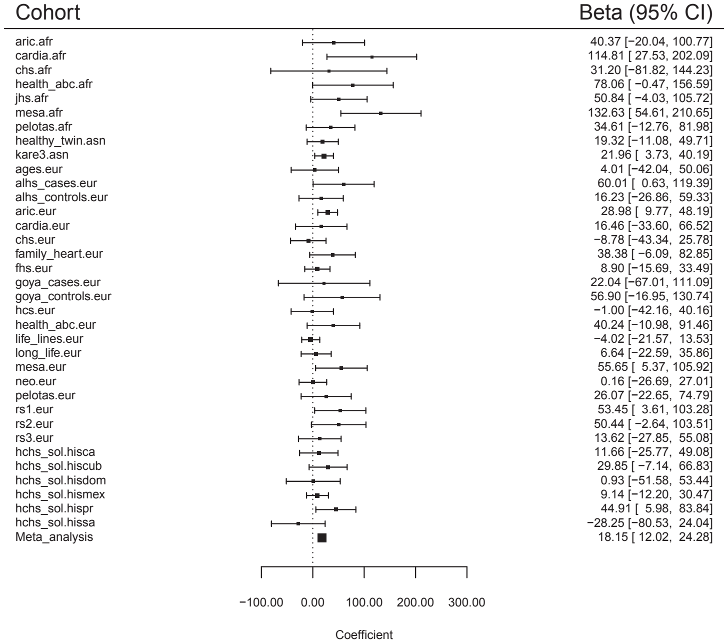

FVC_multiethnic_ancestry_rs8089865

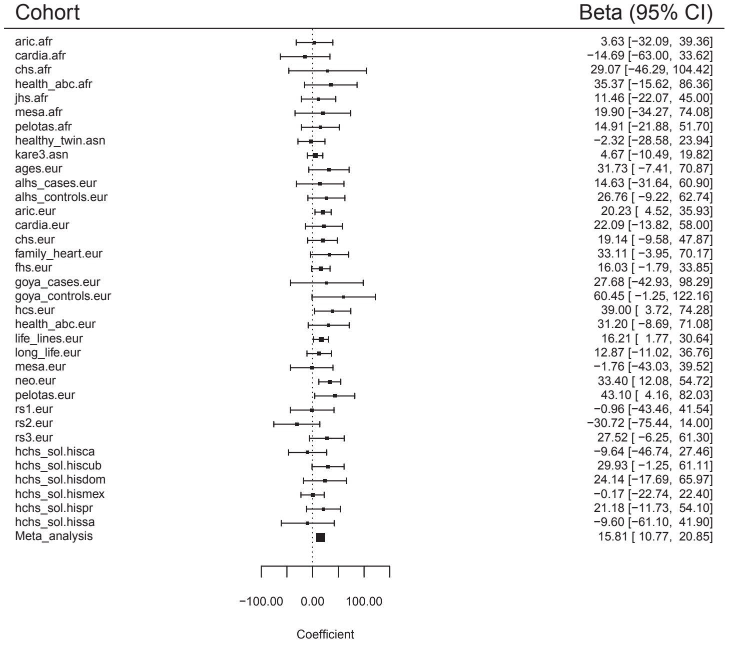

C.

FEV_1/FVC_multiethnic_ancestry_rs11591179

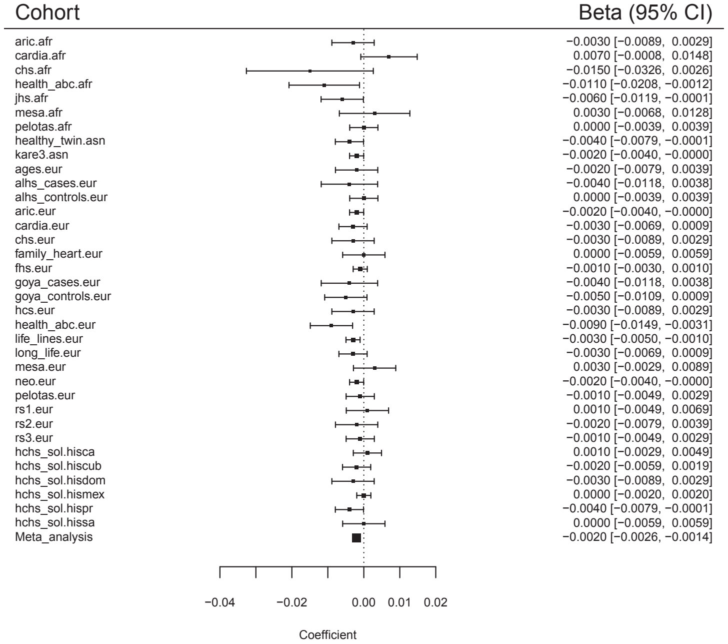

FEV_1/FVC_multiethnic_ancestry_rs72904209

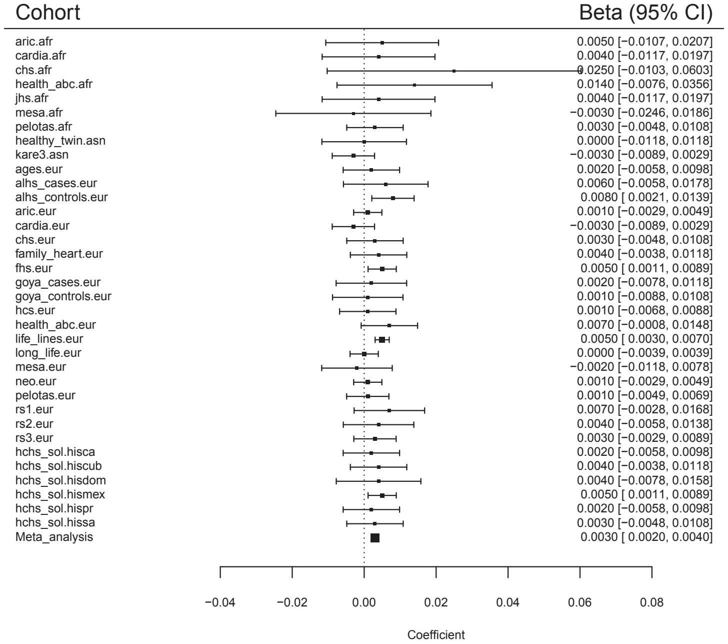

FEV_1/FVC_multiethnic_ancestry_rs28723417

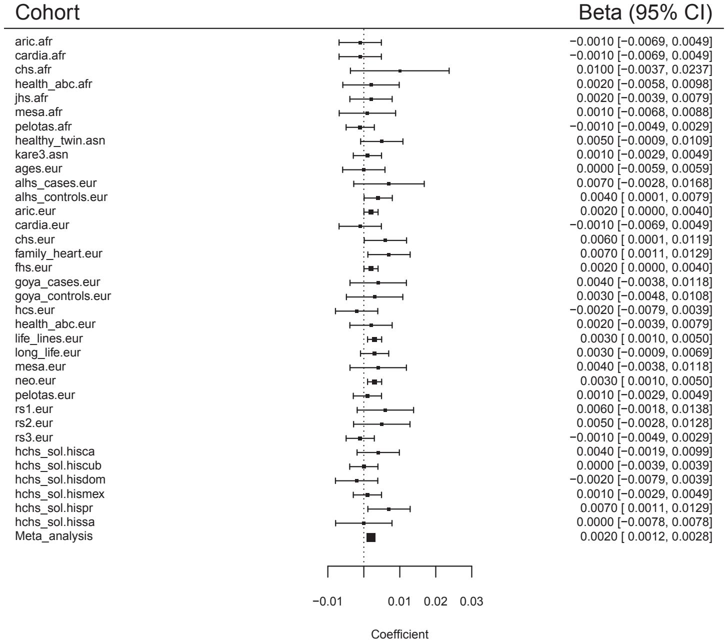

FEV_1/FVC_multiethnic_ancestry_rs80217917

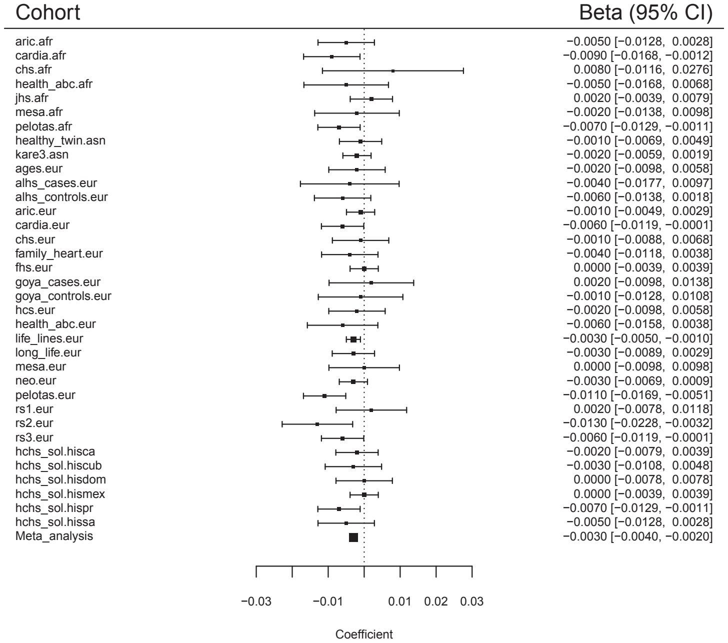

FEV$_1$/FVC_multiethnic_ancestry_rs28520091

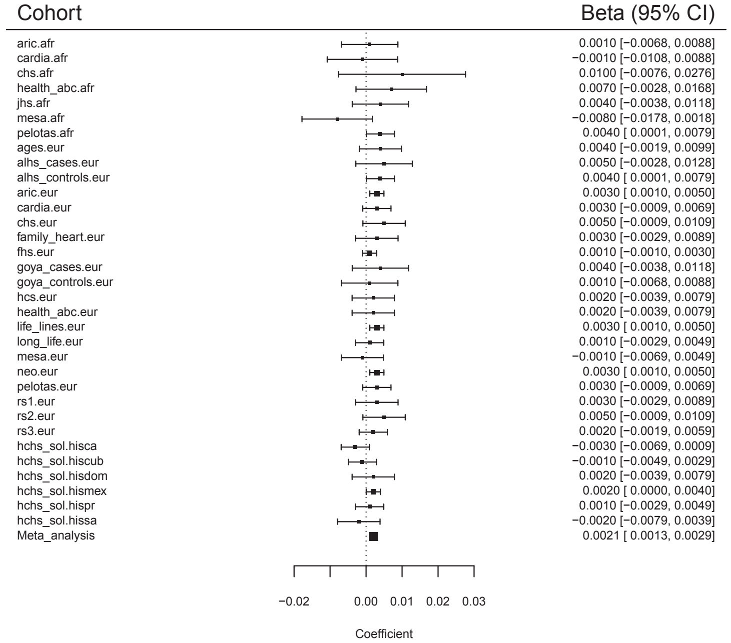

FEV$_1$/FVC_multiethnic_ancestry_rs9350408

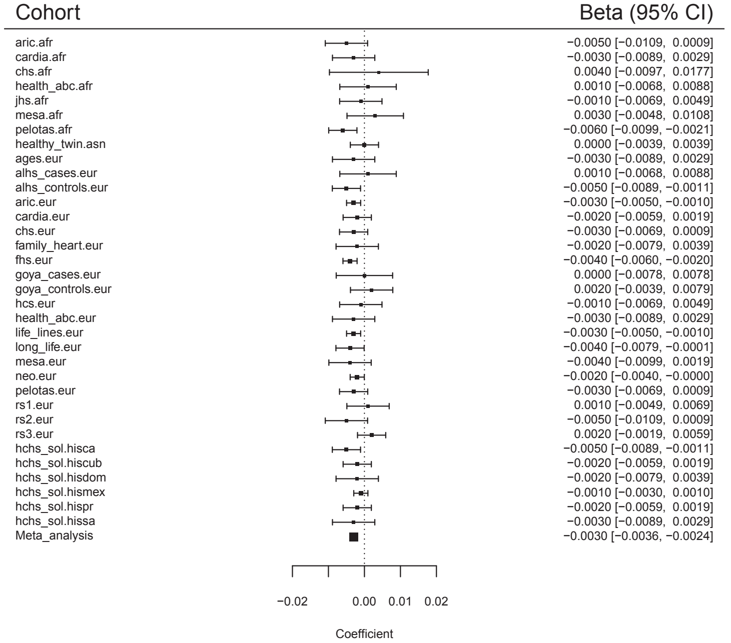

FEV$_1$/FVC_multiethnic_ancestry_rs10965947

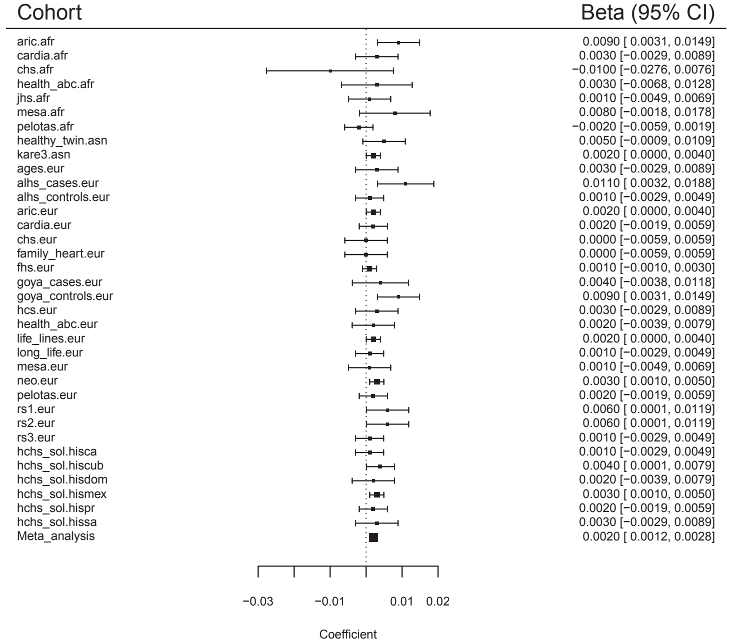

FEV$_1$/FVC_multiethnic_ancestry_rs2451951

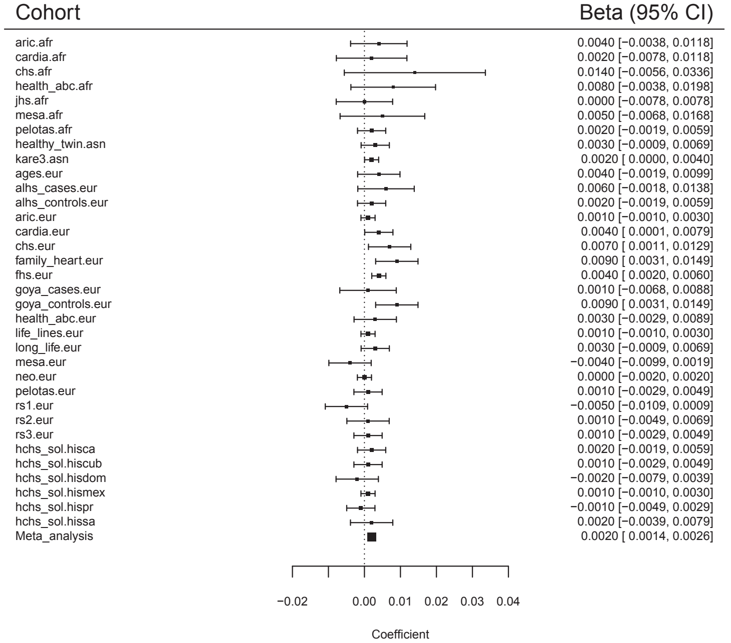

FEV$_1$/FVC_multiethnic_ancestry_rs75159994

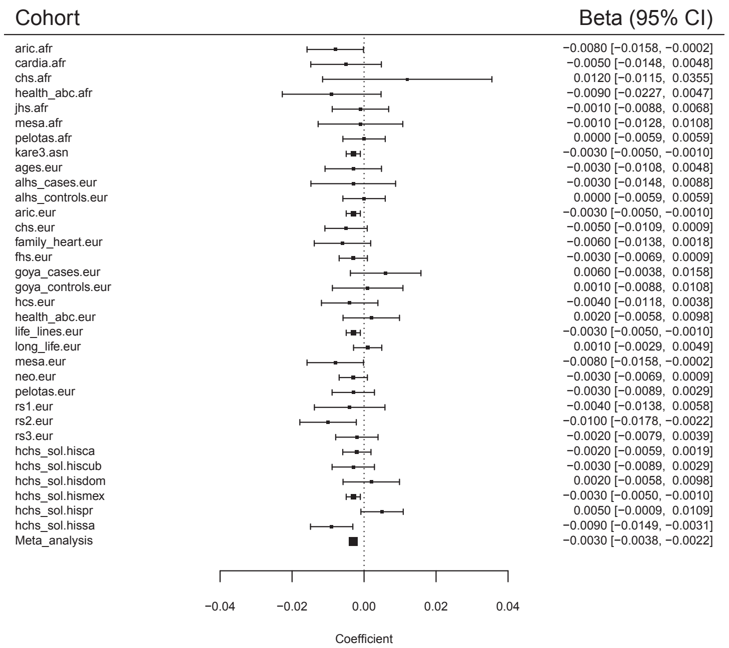

FEV$_1$/FVC_multiethnic_ancestry_rs2293871

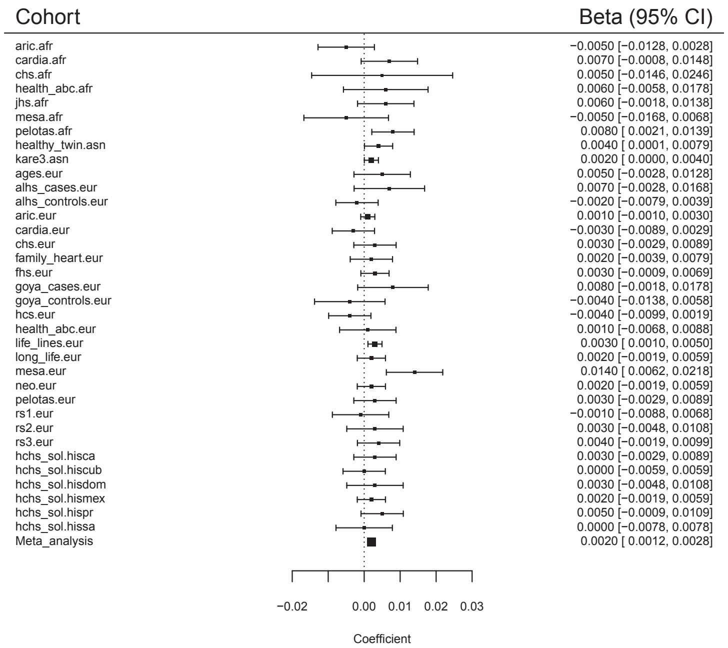

FEV$_1$/FVC_multiethnic_ancestry_11:73280955:GA_G

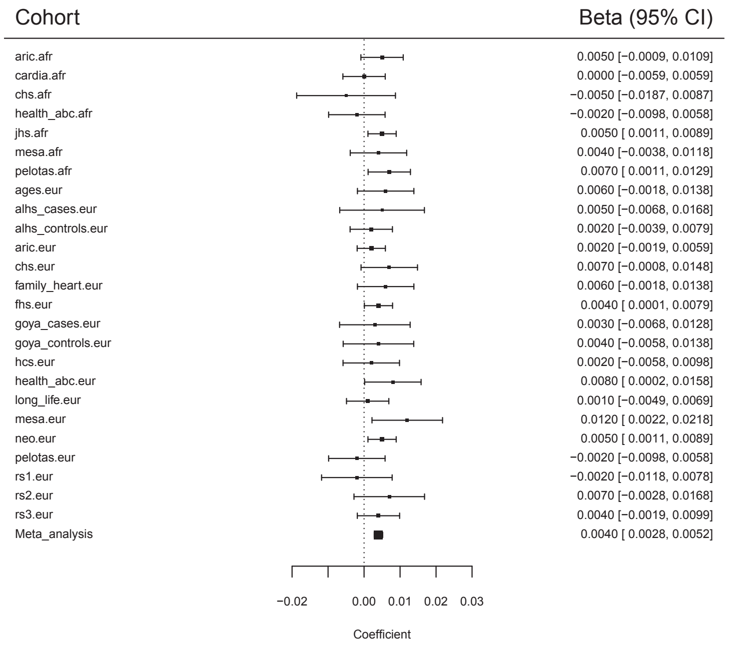

FEV$_1$/FVC_multiethnic_ancestry_rs4444235

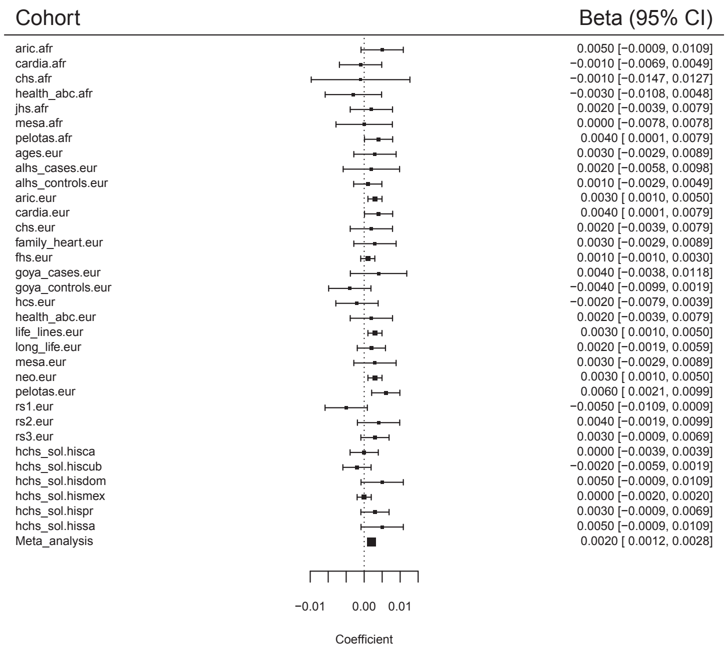

FEV_1/FVC_multiethnic_ancestry_rs9636166

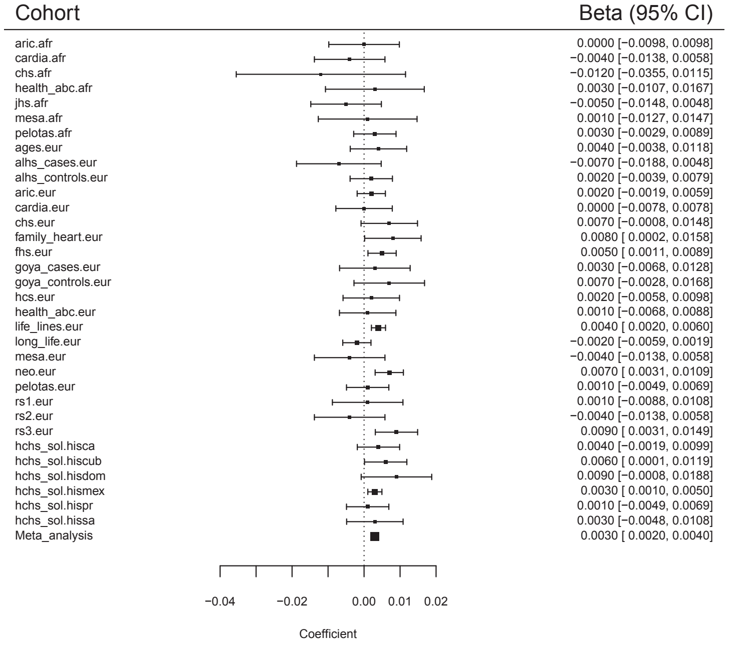

FEV_1/FVC_multiethnic_ancestry_rs4820216

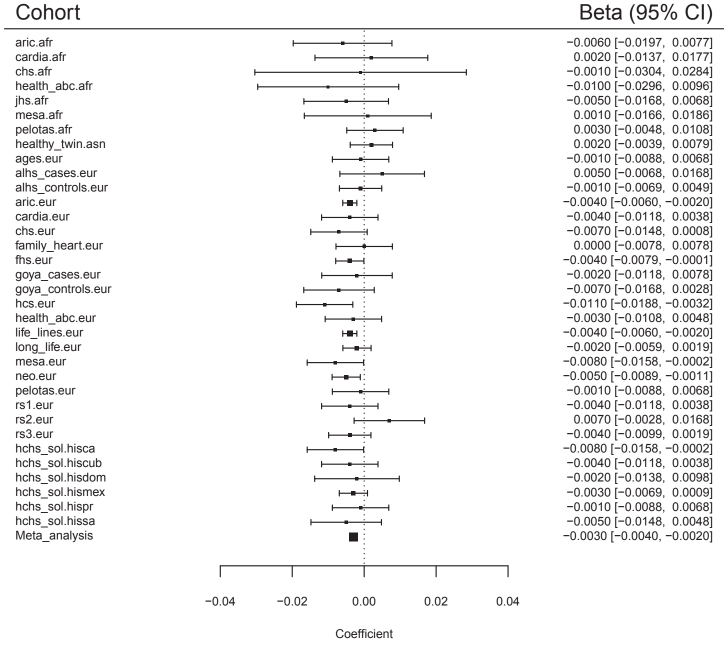

D.

FEV_1_European_ancestry_rs252746

Cohort	Beta (95% CI)
aric	11.20 [−6.26, 28.65]
cardia	46.18 [11.45, 80.91]
mesa	−18.22 [−61.65, 25.21]
fhs	18.14 [−1.60, 37.88]
ages	44.75 [1.08, 88.42]
alhs_cases	59.67 [5.43, 113.92]
alhs_controls	4.52 [−33.18, 42.22]
chs	4.14 [−26.05, 34.34]
family_heart	20.29 [−19.28, 59.86]
goya_cases	40.41 [−40.70, 121.53]
goya_controls	44.79 [−19.99, 109.57]
hcs	57.00 [19.76, 94.24]
health_abc	11.37 [−33.64, 56.38]
life_lines	21.27 [6.60, 35.95]
long_life	14.24 [−11.67, 40.15]
neo	19.50 [−3.55, 42.55]
pelotas	24.64 [−15.69, 64.97]
rs1	60.88 [17.36, 104.40]
rs2	28.12 [−19.17, 75.42]
rs3	−6.32 [−42.93, 30.29]
Meta_analysis	20.05 [13.29, 26.81]

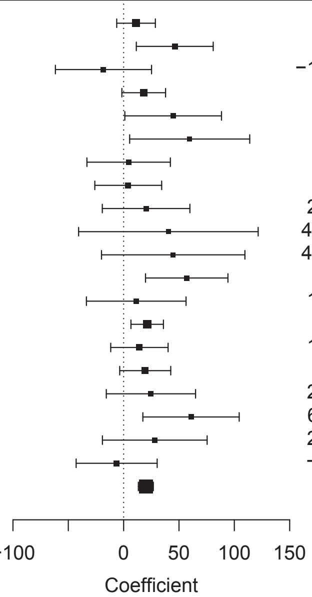

FEV_1_European_ancestry_rs10779158

Cohort	Beta (95% CI)
aric	16.10 [1.50, 30.70]
cardia	22.71 [−8.34, 53.76]
mesa	−8.30 [−42.70, 26.11]
fhs	17.83 [2.09, 33.58]
ages	6.77 [−25.78, 39.32]
alhs_cases	−25.46 [−72.40, 21.48]
alhs_controls	28.14 [−3.89, 60.16]
chs	12.08 [−13.38, 37.54]
family_heart	46.75 [13.74, 79.76]
goya_cases	−1.70 [−70.37, 66.98]
goya_controls	13.17 [−43.08, 69.43]
hcs	29.00 [−0.40, 58.40]
health_abc	6.77 [−30.78, 44.32]
life_lines	28.12 [15.45, 40.79]
long_life	6.45 [−15.44, 28.34]
neo	5.96 [−14.05, 25.97]
pelotas	39.09 [−0.32, 78.51]
rs1	7.53 [−29.25, 44.31]
rs2	−11.93 [−51.98, 28.12]
rs3	−6.12 [−36.33, 24.10]
Meta_analysis	15.89 [10.21, 21.57]

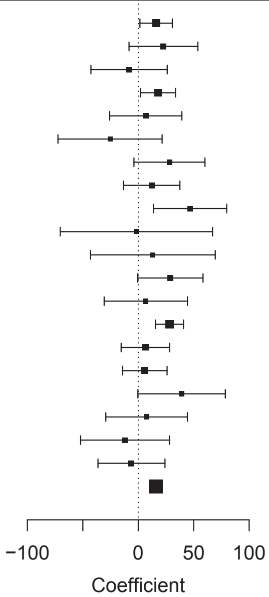

FEV_1_European_ancestry_rs143246821

Cohort	Beta (95% CI)
aric	24.66 [7.65, 41.67]
mesa	28.06 [−15.29, 71.41]
fhs	37.59 [15.46, 59.73]
alhs_cases	65.96 [10.09, 121.83]
alhs_controls	35.29 [−1.88, 72.46]
life_lines	20.38 [−8.44, 49.20]
neo	26.78 [3.61, 49.95]
pelotas	51.37 [9.98, 92.75]
Meta_analysis	30.58 [20.80, 40.36]

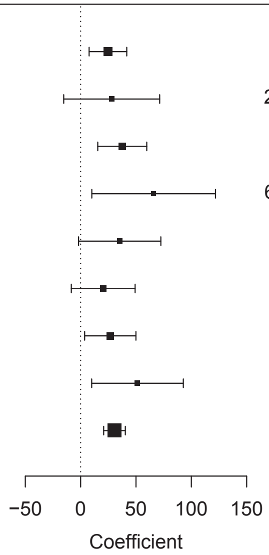

FEV_1_European_ancestry_rs916888

Cohort	Beta (95% CI)
aric	32.58 [15.00, 50.17]
cardia	5.14 [−36.96, 47.24]
mesa	9.06 [−33.77, 51.90]
fhs	33.46 [12.92, 54.00]
ages	−4.47 [−48.75, 39.81]
alhs_cases	54.61 [2.31, 106.91]
alhs_controls	18.10 [−17.45, 53.65]
chs	16.22 [−14.39, 46.83]
family_heart	50.35 [13.40, 87.30]
goya_cases	−27.47 [−107.32, 52.37]
goya_controls	44.07 [−23.74, 111.88]
hcs	40.00 [−3.12, 83.12]
health_abc	−0.11 [−43.02, 42.79]
life_lines	8.42 [−6.83, 23.67]
long_life	7.64 [−17.06, 32.33]
neo	24.18 [1.64, 46.72]
pelotas	38.54 [−1.03, 78.11]
rs1	34.68 [−7.77, 77.13]
rs2	7.05 [−37.77, 51.87]
rs3	3.52 [−32.06, 39.09]
Meta_analysis	20.53 [13.71, 27.35]

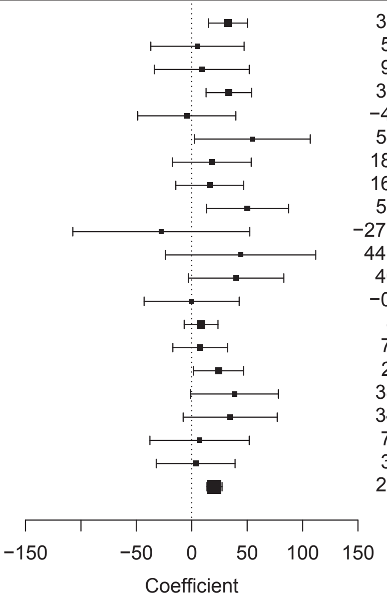

E.

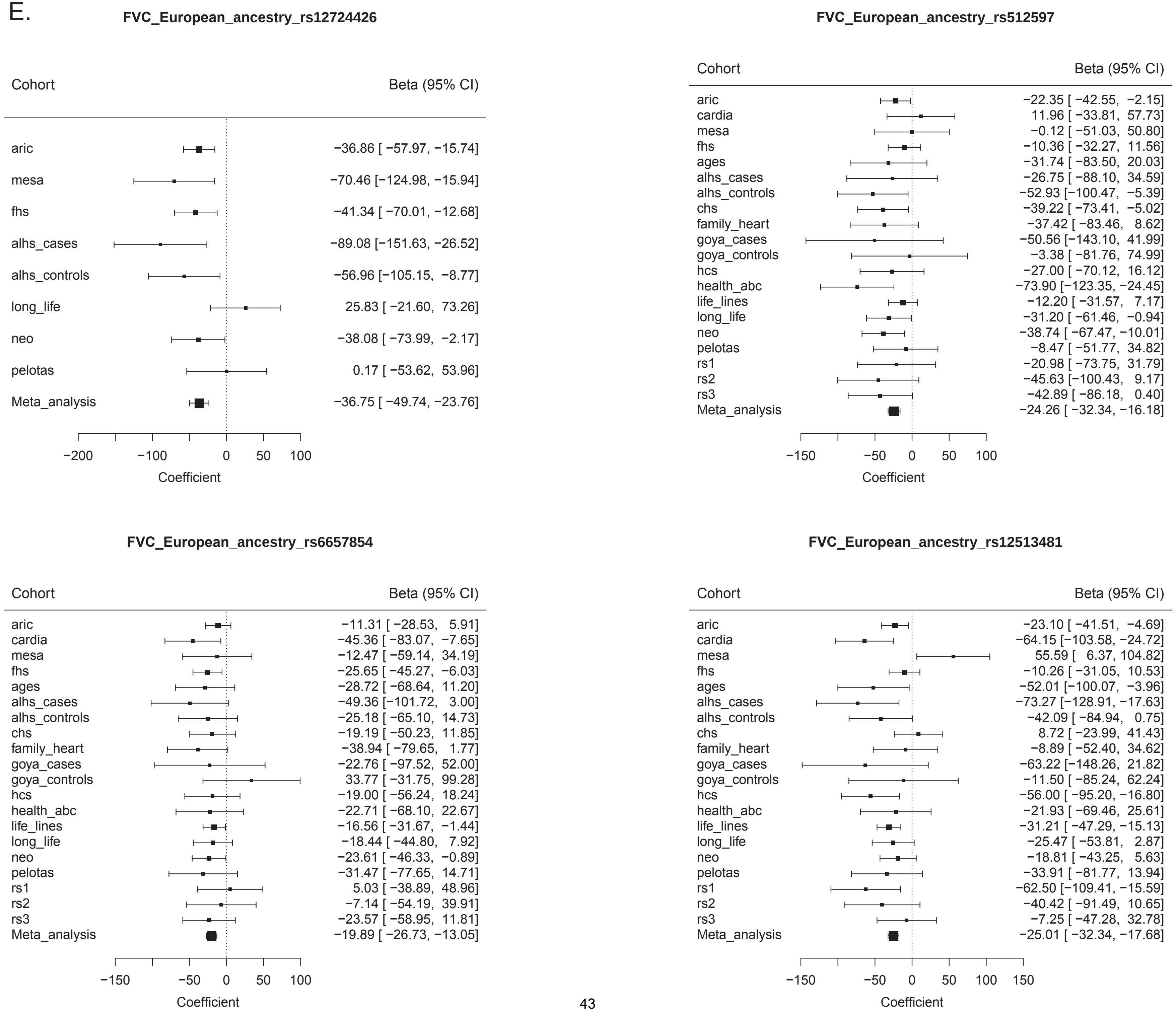
FVC_European_ancestry_rs12724426
Cohort
Beta (95% CI)
aric −36.86 [−57.97, −15.74]
mesa −70.46 [−124.98, −15.94]
fhs −41.34 [−70.01, −12.68]
alhs_cases −89.08 [−151.63, −26.52]
alhs_controls −56.96 [−105.15, −8.77]
long_life 25.83 [−21.60, 73.26]
neo −38.08 [−73.99, −2.17]
pelotas 0.17 [−53.62, 53.96]
Meta_analysis −36.75 [−49.74, −23.76]
−200 −100 0 50 100
Coefficient
FVC_European_ancestry_rs512597
Cohort
Beta (95% CI)
aric −22.35 [−42.55, −2.15]
cardia 11.96 [−33.81, 57.73]
mesa −0.12 [−51.03, 50.80]
fhs −10.36 [−32.27, 11.56]
ages −31.74 [−83.50, 20.03]
alhs_cases −26.75 [−88.10, 34.59]
alhs_controls −52.93 [−100.47, −5.39]
chs −39.22 [−73.41, −5.02]
family_heart −37.42 [−83.46, 8.62]
goya_cases −50.56 [−143.10, 41.99]
goya_controls −3.38 [−81.76, 74.99]
hcs −27.00 [−70.12, 16.12]
health_abc −73.90 [−123.35, −24.45]
life_lines −12.20 [−31.57, 7.17]
long_life −31.20 [−61.46, −0.94]
neo −38.74 [−67.47, −10.01]
pelotas −8.47 [−51.77, 34.82]
rs1 −20.98 [−73.75, 31.79]
rs2 −45.63 [−100.43, 9.17]
rs3 −42.89 [−86.18, 0.40]
Meta_analysis −24.26 [−32.34, −16.18]
−150 −50 0 50 100
Coefficient
FVC_European_ancestry_rs6657854
Cohort
Beta (95% CI)
aric −11.31 [−28.53, 5.91]
cardia −45.36 [−83.07, −7.65]
mesa −12.47 [−59.14, 34.19]
fhs −25.65 [−45.27, −6.03]
ages −28.72 [−68.64, 11.20]
alhs_cases −49.36 [−101.72, 3.00]
alhs_controls −25.18 [−65.10, 14.73]
chs −19.19 [−50.23, 11.85]
family_heart −38.94 [−79.65, 1.77]
goya_cases −22.76 [−97.52, 52.00]
goya_controls 33.77 [−31.75, 99.28]
hcs −19.00 [−56.24, 18.24]
health_abc −22.71 [−68.10, 22.67]
life_lines −16.56 [−31.67, −1.44]
long_life −18.44 [−44.80, 7.92]
neo −23.61 [−46.33, −0.89]
pelotas −31.47 [−77.65, 14.71]
rs1 5.03 [−38.89, 48.96]
rs2 −7.14 [−54.19, 39.91]
rs3 −23.57 [−58.95, 11.81]
Meta_analysis −19.89 [−26.73, −13.05]
−150 −50 0 50 100
Coefficient
FVC_European_ancestry_rs12513481
Cohort
Beta (95% CI)
aric −23.10 [−41.51, −4.69]
cardia −64.15 [−103.58, −24.72]
mesa 55.59 [6.37, 104.82]
fhs −10.26 [−31.05, 10.53]
ages −52.01 [−100.07, −3.96]
alhs_cases −73.27 [−128.91, −17.63]
alhs_controls −42.09 [−84.94, 0.75]
chs 8.72 [−23.99, 41.43]
family_heart −8.89 [−52.40, 34.62]
goya_cases −63.22 [−148.26, 21.82]
goya_controls −11.50 [−85.24, 62.24]
hcs −56.00 [−95.20, −16.80]
health_abc −21.93 [−69.46, 25.61]
life_lines −31.21 [−47.29, −15.13]
long_life −25.47 [−53.81, 2.87]
neo −18.81 [−43.25, 5.63]
pelotas −33.91 [−81.77, 13.94]
rs1 −62.50 [−109.41, −15.59]
rs2 −40.42 [−91.49, 10.65]
rs3 −7.25 [−47.28, 32.78]
Meta_analysis −25.01 [−32.34, −17.68]
−150 −50 0 50 100 150
Coefficient

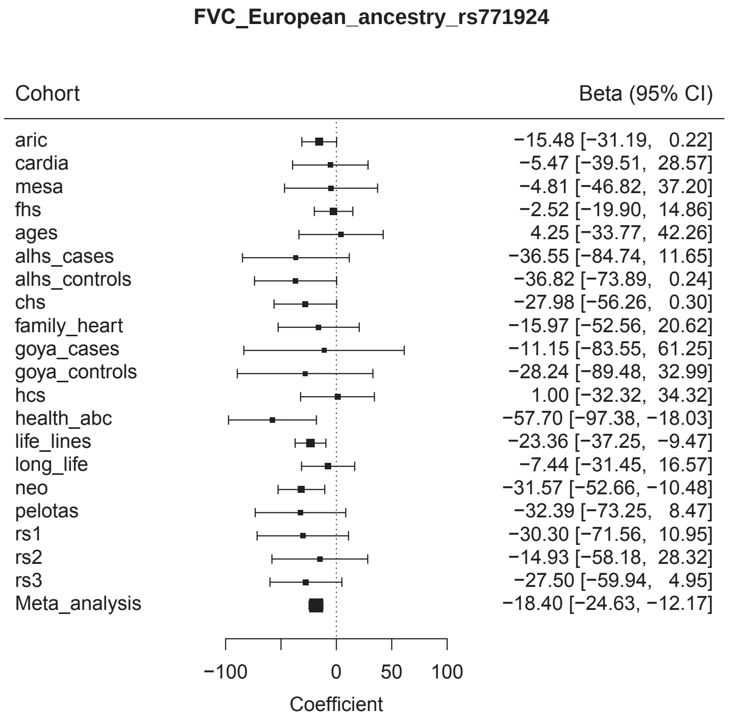
FVC_European_ancestry_rs771924
Cohort
Beta (95% CI)
aric −15.48 [−31.19, 0.22]
cardia −5.47 [−39.51, 28.57]
mesa −4.81 [−46.82, 37.20]
fhs −2.52 [−19.90, 14.86]
ages 4.25 [−33.77, 42.26]
alhs_cases −36.55 [−84.74, 11.65]
alhs_controls −36.82 [−73.89, 0.24]
chs −27.98 [−56.26, 0.30]
family_heart −15.97 [−52.56, 20.62]
goya_cases −11.15 [−83.55, 61.25]
goya_controls −28.24 [−89.48, 32.99]
hcs 1.00 [−32.32, 34.32]
health_abc −57.70 [−97.38, −18.03]
life_lines −23.36 [−37.25, −9.47]
long_life −7.44 [−31.45, 16.57]
neo −31.57 [−52.66, −10.48]
pelotas −32.39 [−73.25, 8.47]
rs1 −30.30 [−71.56, 10.95]
rs2 −14.93 [−58.18, 28.32]
rs3 −27.50 [−59.94, 4.95]
Meta_analysis −18.40 [−24.63, −12.17]
−100
0
50
100
Coefficient

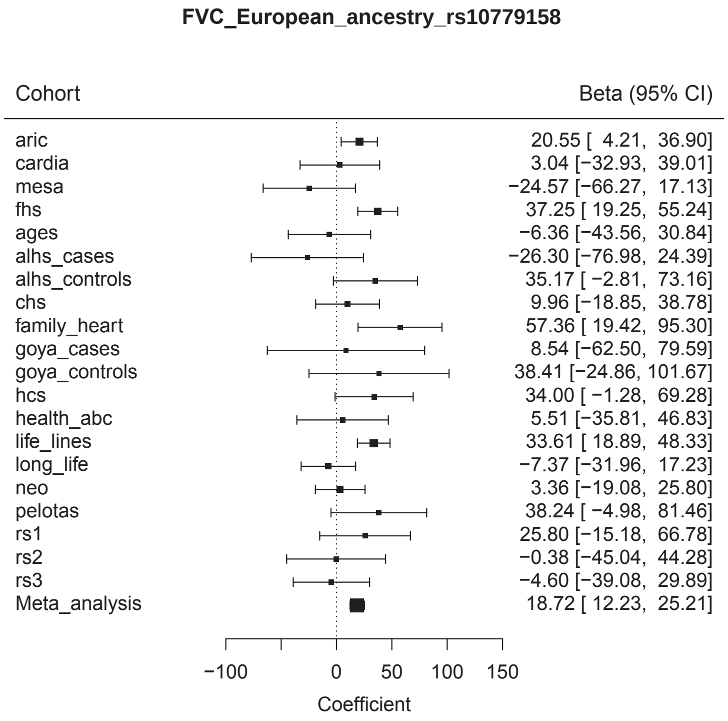
FVC_European_ancestry_rs10779158
Cohort
Beta (95% CI)
aric 20.55 [4.21, 36.90]
cardia 3.04 [−32.93, 39.01]
mesa −24.57 [−66.27, 17.13]
fhs 37.25 [19.25, 55.24]
ages −6.36 [−43.56, 30.84]
alhs_cases −26.30 [−76.98, 24.39]
alhs_controls 35.17 [−2.81, 73.16]
chs 9.96 [−18.85, 38.78]
family_heart 57.36 [19.42, 95.30]
goya_cases 8.54 [−62.50, 79.59]
goya_controls 38.41 [−24.86, 101.67]
hcs 34.00 [−1.28, 69.28]
health_abc 5.51 [−35.81, 46.83]
life_lines 33.61 [18.89, 48.33]
long_life −7.37 [−31.96, 17.23]
neo 3.36 [−19.08, 25.80]
pelotas 38.24 [−4.98, 81.46]
rs1 25.80 [−15.18, 66.78]
rs2 −0.38 [−45.04, 44.28]
rs3 −4.60 [−39.08, 29.89]
Meta_analysis 18.72 [12.23, 25.21]
−100
0
50
100
150
Coefficient

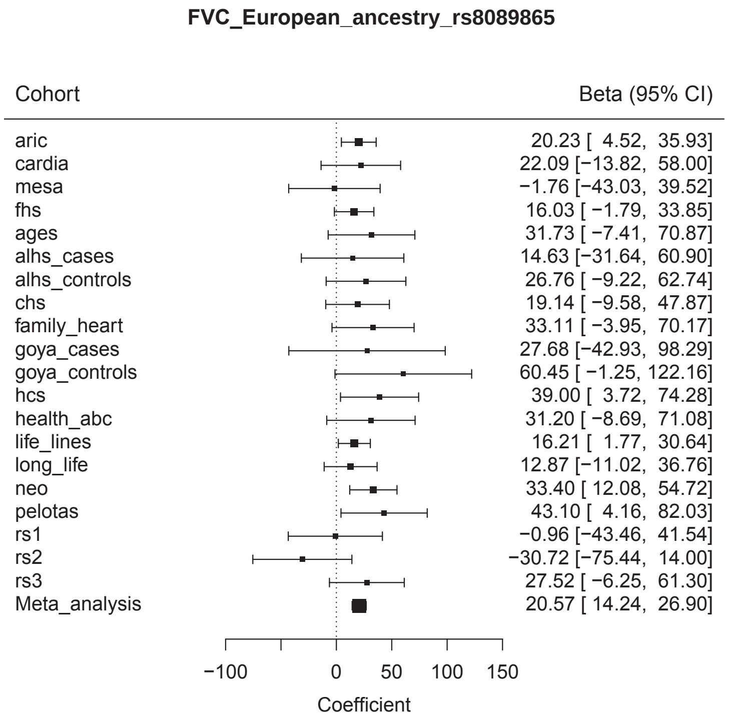
FVC_European_ancestry_rs8089865
Cohort
Beta (95% CI)
aric 20.23 [4.52, 35.93]
cardia 22.09 [−13.82, 58.00]
mesa −1.76 [−43.03, 39.52]
fhs 16.03 [−1.79, 33.85]
ages 31.73 [−7.41, 70.87]
alhs_cases 14.63 [−31.64, 60.90]
alhs_controls 26.76 [−9.22, 62.74]
chs 19.14 [−9.58, 47.87]
family_heart 33.11 [−3.95, 70.17]
goya_cases 27.68 [−42.93, 98.29]
goya_controls 60.45 [−1.25, 122.16]
hcs 39.00 [3.72, 74.28]
health_abc 31.20 [−8.69, 71.08]
life_lines 16.21 [1.77, 30.64]
long_life 12.87 [−11.02, 36.76]
neo 33.40 [12.08, 54.72]
pelotas 43.10 [4.16, 82.03]
rs1 −0.96 [−43.46, 41.54]
rs2 −30.72 [−75.44, 14.00]
rs3 27.52 [−6.25, 61.30]
Meta_analysis 20.57 [14.24, 26.90]
−100
0
50
100
150
Coefficient

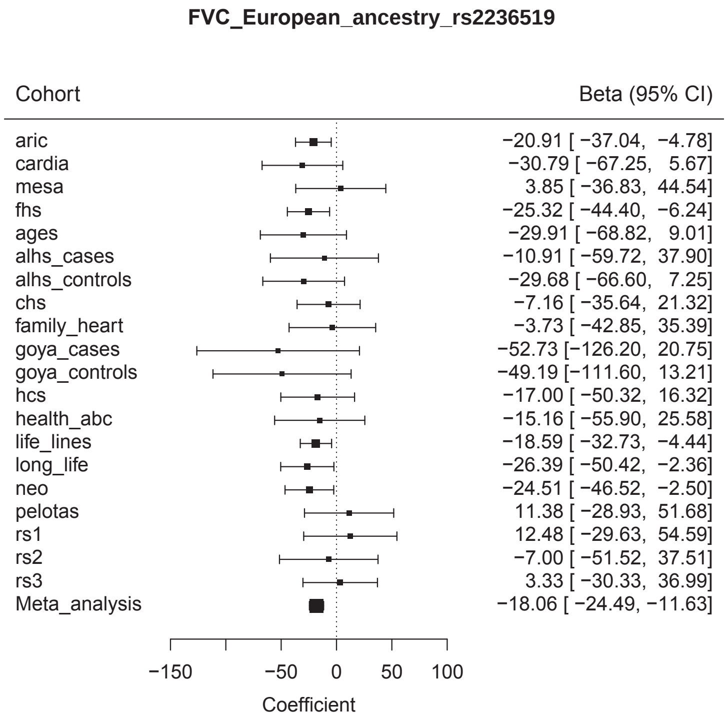
FVC_European_ancestry_rs2236519
Cohort
Beta (95% CI)
aric −20.91 [−37.04, −4.78]
cardia −30.79 [−67.25, 5.67]
mesa 3.85 [−36.83, 44.54]
fhs −25.32 [−44.40, −6.24]
ages −29.91 [−68.82, 9.01]
alhs_cases −10.91 [−59.72, 37.90]
alhs_controls −29.68 [−66.60, 7.25]
chs −7.16 [−35.64, 21.32]
family_heart −3.73 [−42.85, 35.39]
goya_cases −52.73 [−126.20, 20.75]
goya_controls −49.19 [−111.60, 13.21]
hcs −17.00 [−50.32, 16.32]
health_abc −15.16 [−55.90, 25.58]
life_lines −18.59 [−32.73, −4.44]
long_life −26.39 [−50.42, −2.36]
neo −24.51 [−46.52, −2.50]
pelotas 11.38 [−28.93, 51.68]
rs1 12.48 [−29.63, 54.59]
rs2 −7.00 [−51.52, 37.51]
rs3 3.33 [−30.33, 36.99]
Meta_analysis −18.06 [−24.49, −11.63]
−150
−50
0
50
100
Coefficient

F.

FEV$_1$/FVC_European_ancestry_rs17666332

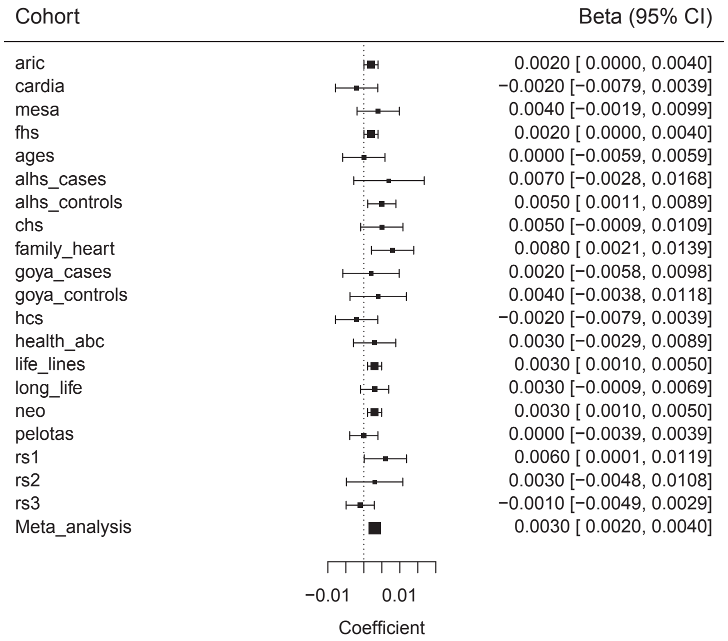

FEV$_1$/FVC_European_ancestry_rs28520091

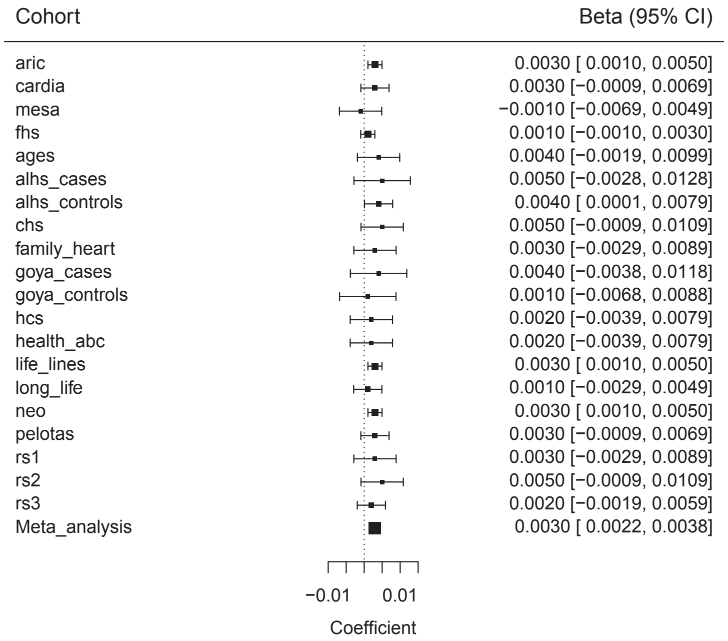

FEV$_1$/FVC_European_ancestry_rs1928168

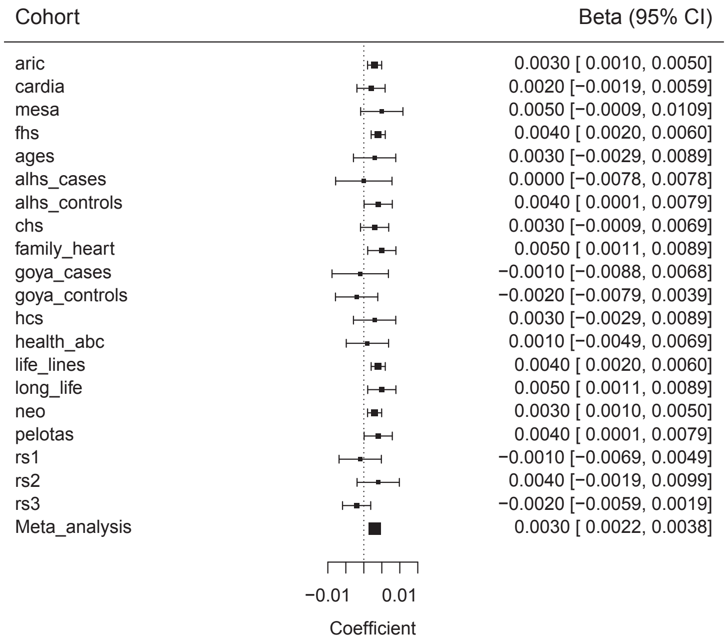

FEV$_1$/FVC_European_ancestry_rs9351637

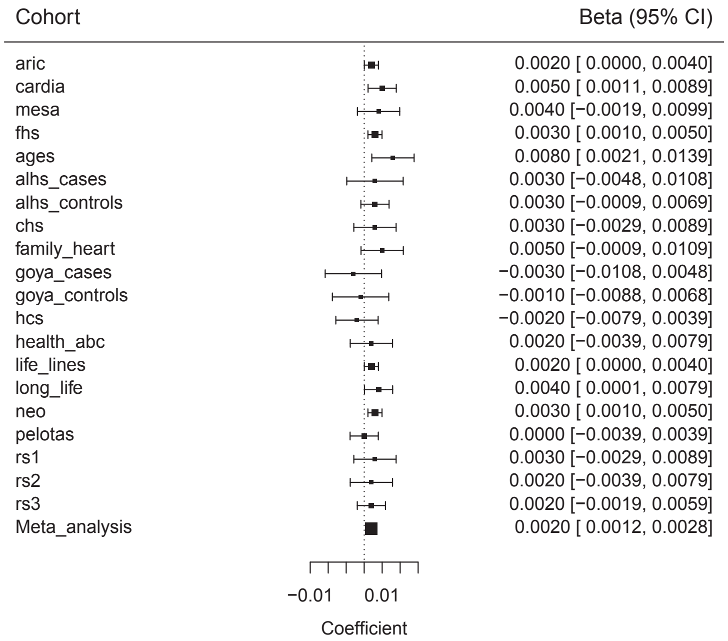

FEV_1/FVC_European_ancestry_rs1404154

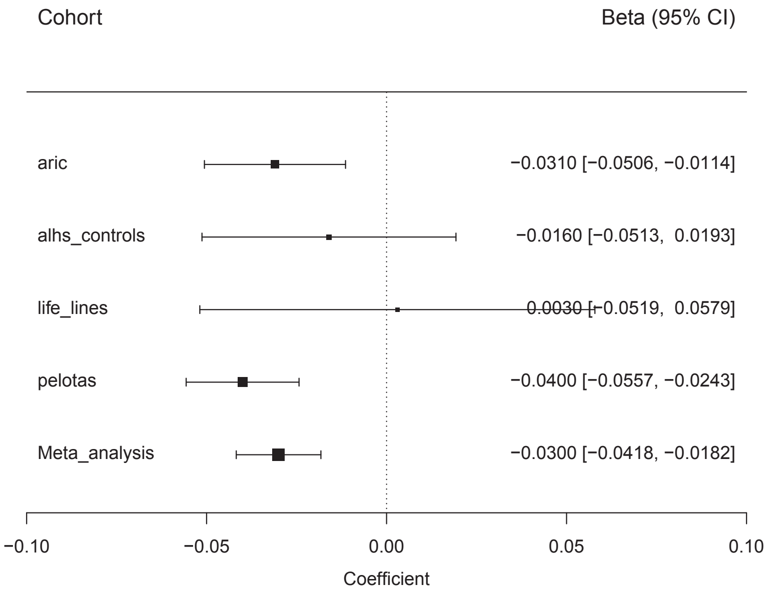

FEV_1/FVC_European_ancestry_rs1353531

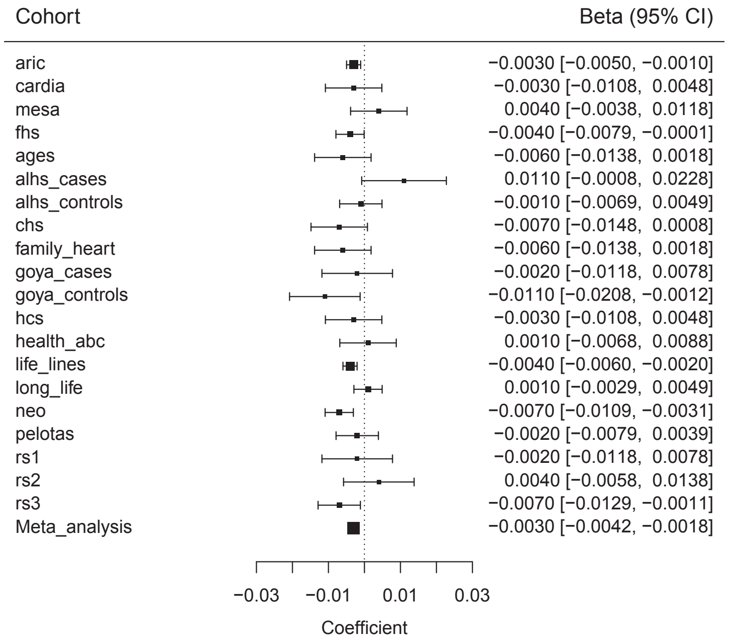

FEV_1/FVC_European_ancestry_rs4820216

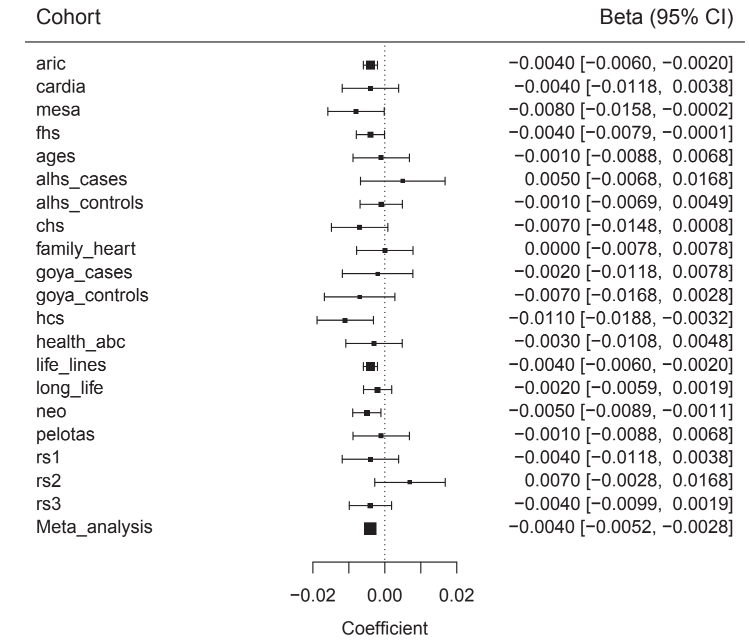

G.

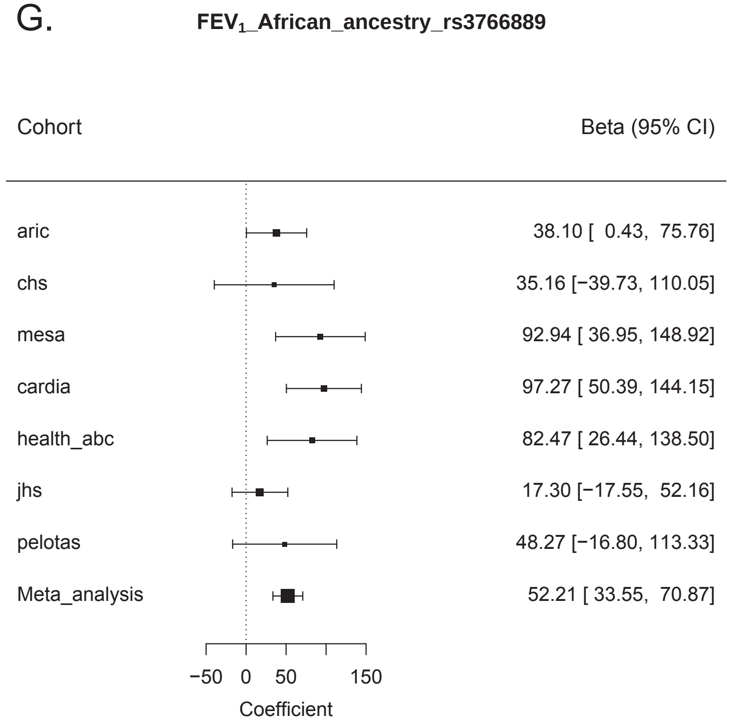
FEV1_African_ancestry_rs3766889
Cohort
Beta (95% CI)
aric
38.10 [0.43, 75.76]
chs
35.16 [−39.73, 110.05]
mesa
92.94 [36.95, 148.92]
cardia
97.27 [50.39, 144.15]
health_abc
82.47 [26.44, 138.50]
jhs
17.30 [−17.55, 52.16]
pelotas
48.27 [−16.80, 113.33]
Meta_analysis
52.21 [33.55, 70.87]
−50
0
50
150
Coefficient

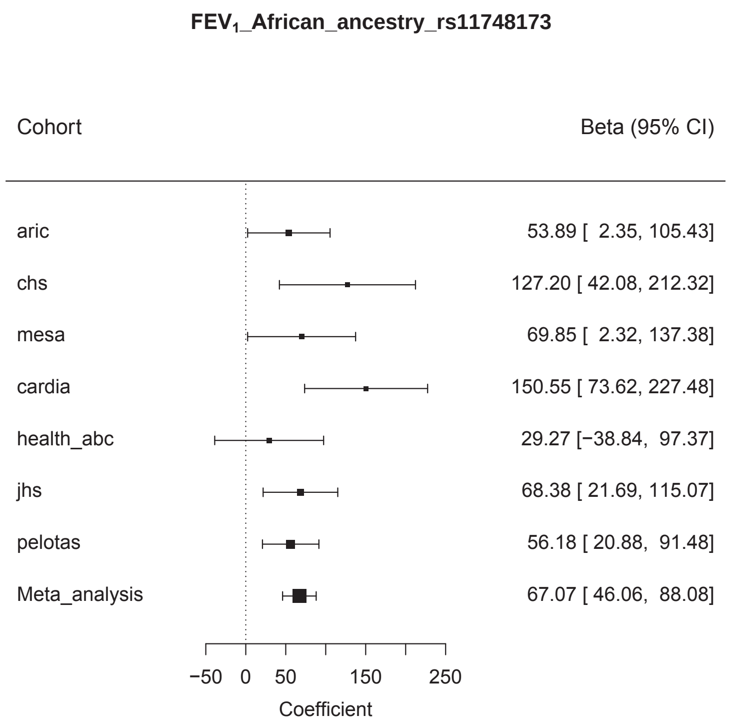
FEV1_African_ancestry_rs11748173
Cohort
Beta (95% CI)
aric
53.89 [2.35, 105.43]
chs
127.20 [42.08, 212.32]
mesa
69.85 [2.32, 137.38]
cardia
150.55 [73.62, 227.48]
health_abc
29.27 [−38.84, 97.37]
jhs
68.38 [21.69, 115.07]
pelotas
56.18 [20.88, 91.48]
Meta_analysis
67.07 [46.06, 88.08]
−50
0
50
150
250
Coefficient

H.

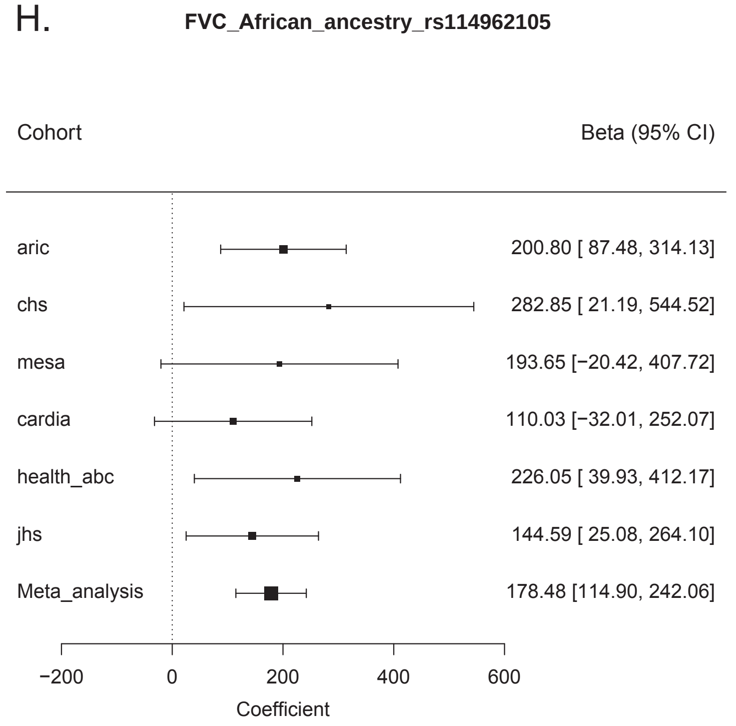
FVC_African_ancestry_rs114962105
Cohort
Beta (95% CI)
aric
200.80 [87.48, 314.13]
chs
282.85 [21.19, 544.52]
mesa
193.65 [−20.42, 407.72]
cardia
110.03 [−32.01, 252.07]
health_abc
226.05 [39.93, 412.17]
jhs
144.59 [25.08, 264.10]
Meta_analysis
178.48 [114.90, 242.06]
−200
0
200
400
600
Coefficient

I.

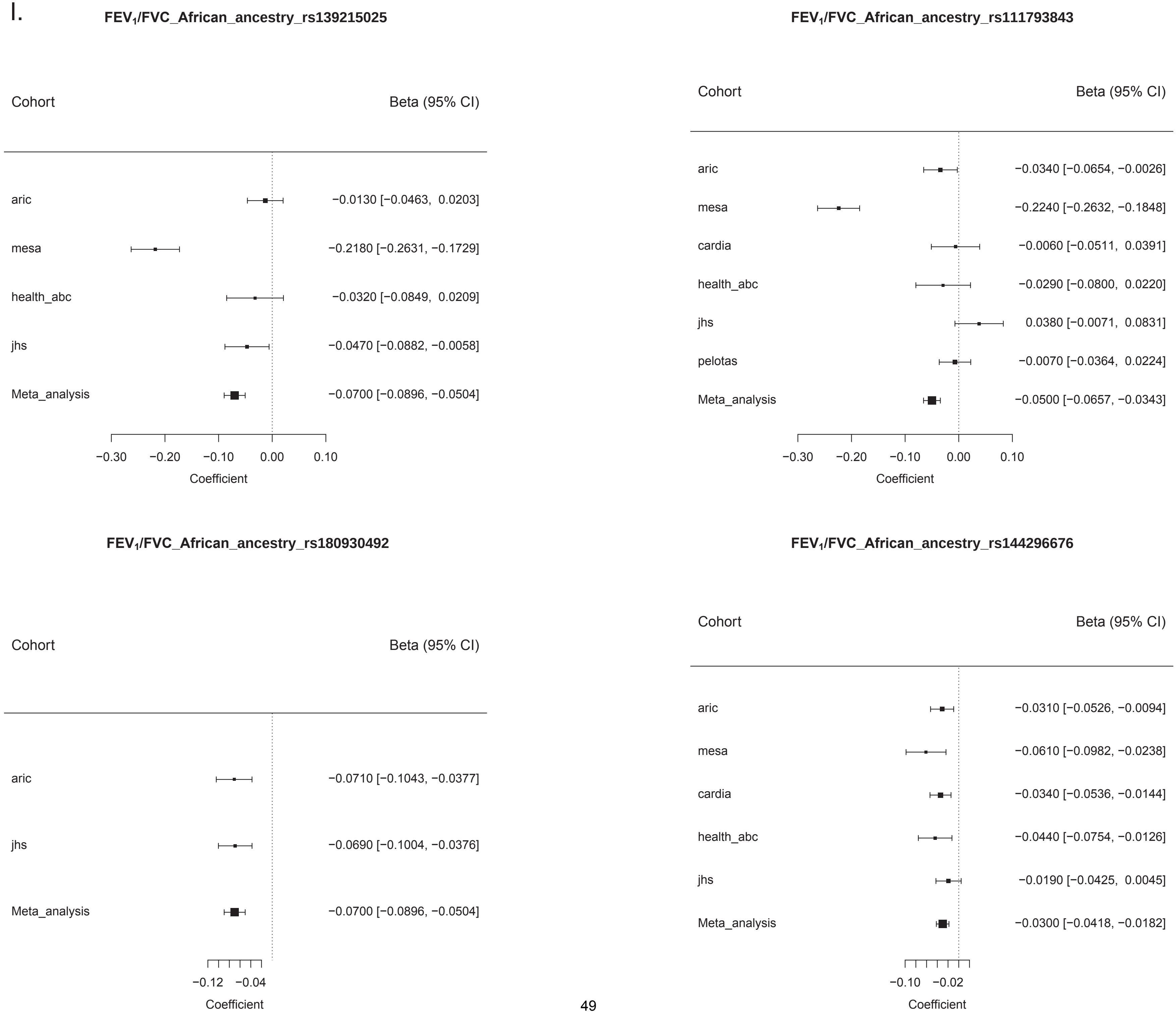
FEV1/FVC_African_ancestry_rs139215025
Cohort
Beta (95% CI)
aric
−0.0130 [−0.0463, 0.0203]
mesa
−0.2180 [−0.2631, −0.1729]
health_abc
−0.0320 [−0.0849, 0.0209]
jhs
−0.0470 [−0.0882, −0.0058]
Meta_analysis
−0.0700 [−0.0896, −0.0504]
−0.30 −0.20 −0.10 0.00 0.10
Coefficient
FEV1/FVC_African_ancestry_rs111793843
Cohort
Beta (95% CI)
aric
−0.0340 [−0.0654, −0.0026]
mesa
−0.2240 [−0.2632, −0.1848]
cardia
−0.0060 [−0.0511, 0.0391]
health_abc
−0.0290 [−0.0800, 0.0220]
jhs
0.0380 [−0.0071, 0.0831]
pelotas
−0.0070 [−0.0364, 0.0224]
Meta_analysis
−0.0500 [−0.0657, −0.0343]
−0.30 −0.20 −0.10 0.00 0.10
Coefficient
FEV1/FVC_African_ancestry_rs180930492
Cohort
Beta (95% CI)
aric
−0.0710 [−0.1043, −0.0377]
jhs
−0.0690 [−0.1004, −0.0376]
Meta_analysis
−0.0700 [−0.0896, −0.0504]
−0.12 −0.04
Coefficient
FEV1/FVC_African_ancestry_rs144296676
Cohort
Beta (95% CI)
aric
−0.0310 [−0.0526, −0.0094]
mesa
−0.0610 [−0.0982, −0.0238]
cardia
−0.0340 [−0.0536, −0.0144]
health_abc
−0.0440 [−0.0754, −0.0126]
jhs
−0.0190 [−0.0425, 0.0045]
Meta_analysis
−0.0300 [−0.0418, −0.0182]
−0.10 −0.02
Coefficient

FEV_1/FVC_African_ancestry_rs147472287

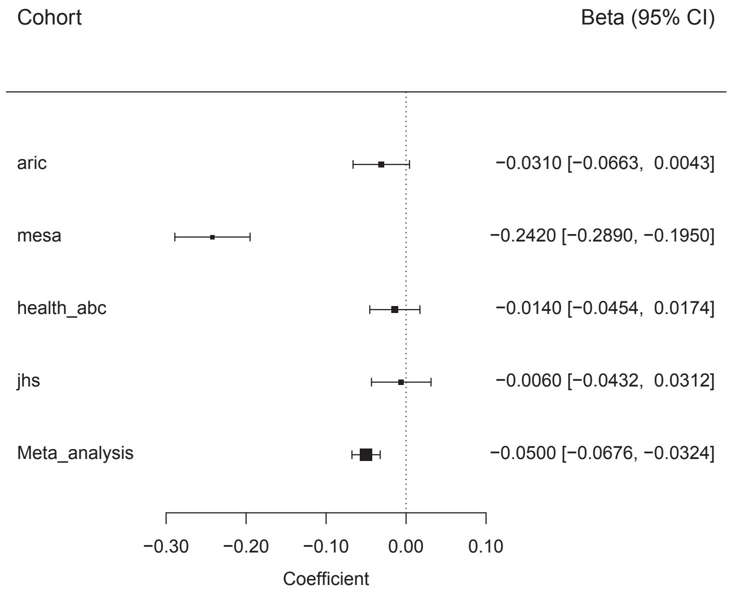

J.

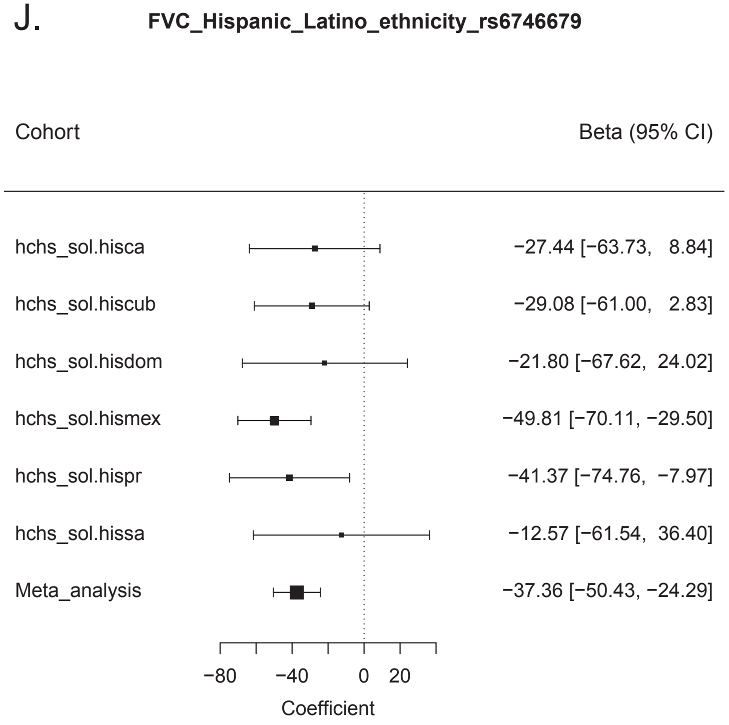

Supplementary Figure 1. Forest Plots for lead variants in/near novel loci identified for pulmonary function in the following CHARGE meta-analyses: Panel A Multiethnic FEV_1; Panel B Multiethnic FVC, Panel C Multiethnic FEV_1/FVC; Panel D European ancestry FEV_1; Panel E European ancestry FVC, Panel F European ancestry FEV_1/FVC; Panel G African ancestry FEV_1; Panel H African ancestry FVC, Panel I African ancestry FEV_1/FVC; Panel J Hispanic/Latino ethnicity FVC.

Cohort abbreviations: AGES Age Gene Environment Susceptibility Study; ALHS Agricultural Lung Health Study; ARIC Atherosclerosis Risk in Communities Study; CARDIA Coronary Artery Risk Development in Young Adults; CHS Cardiovascular Health Study; FamHS Family Heart Study; FHS Framingham Heart Study; GOYA Genetics of Overweight Young Adults Study; HCHS/SOL Hispanic Community Health Study/Study of Latinos; HCS Hunter Community Study; Health ABC; Healthy Twin; JHS Jackson Heart Study; KARE3 Korean Association Resource Phase 3 Study; LifeLines; LLFS Long Life Family Study; MESA Multi-Ethnic Study of Atherosclerosis; 1982 Pelotas; NEO Netherlands Epidemiology of Obesity Study; RS Rotterdam Study (I,II,III). Ancestry/ethnicity abbreviations in multiethnic plots: Afr African ancestry; Asn Asian ancestry; Eur European ancestry; HisCA Hispanic/Latino Central American; HisCub Hispanic/Latino Cuban; HisDom Hispanic/Latino Dominican; HisMex Hispanic/Latino Mexican; HisPR Hispanic/Latino Puerto Rican; HisSA Hispanic/Latino South American

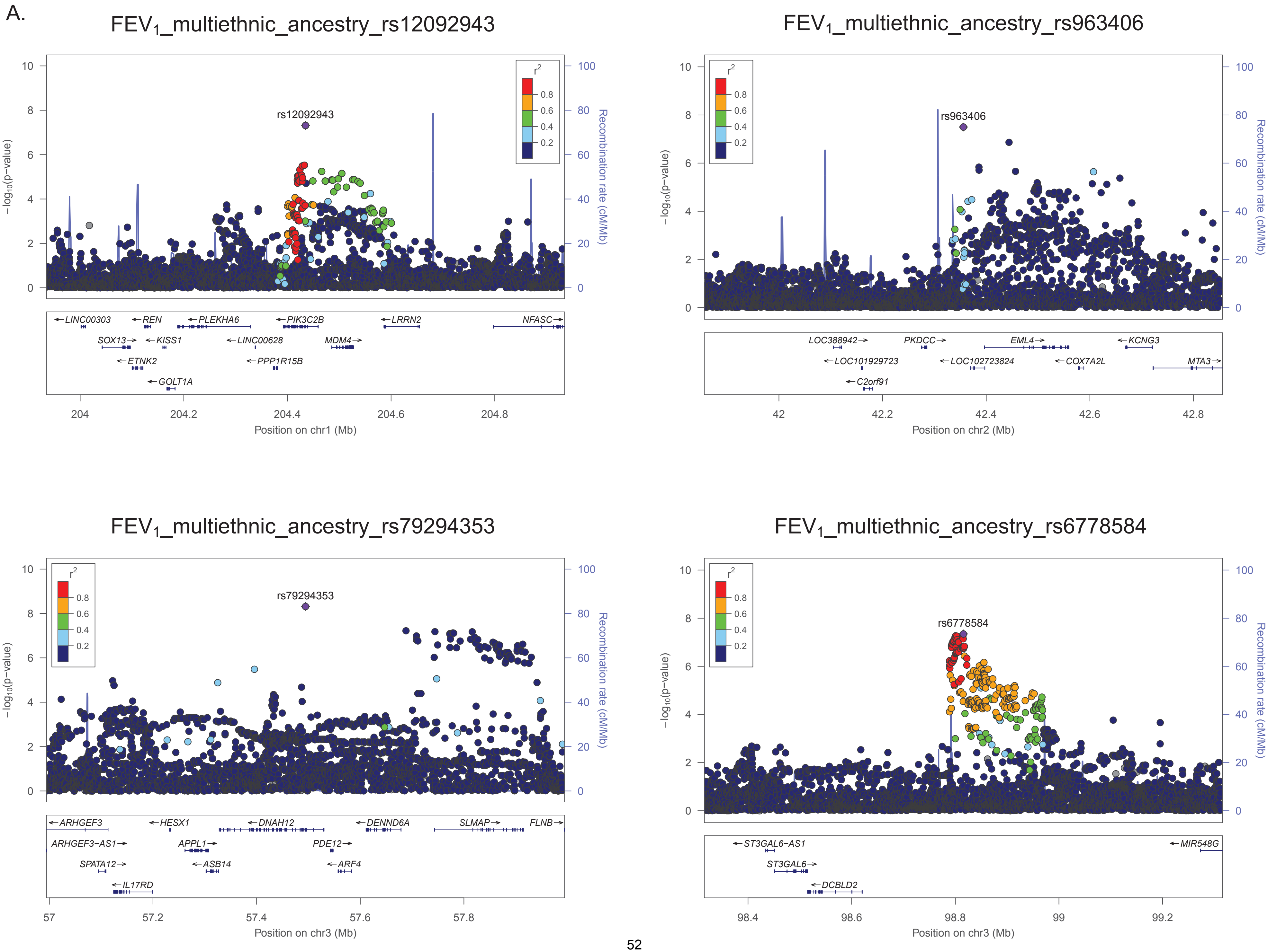
A.
FEV1_multiethnic_ancestry_rs12092943
rs12092943
−log10(p−value)
Recombination rate (cM/Mb)
r2
0.8
0.6
0.4
0.2
LINC00303
REN
PLEKHA6
PIK3C2B
LRRN2
NFASC
SOX13
KISS1
LINC00628
MDM4
ETNK2
PPP1R15B
GOLT1A
Position on chr1 (Mb)
FEV1_multiethnic_ancestry_rs963406
rs963406
LOC388942
PKDCC
EML4
KCNG3
LOC101929723
LOC102723824
COX7A2L
MTA3
C2orf91
Position on chr2 (Mb)
FEV1_multiethnic_ancestry_rs79294353
rs79294353
ARHGEF3
HESX1
DNAH12
DENND6A
SLMAP
FLNB
ARHGEF3−AS1
APPL1
PDE12
SPATA12
ASB14
ARF4
IL17RD
Position on chr3 (Mb)
FEV1_multiethnic_ancestry_rs6778584
rs6778584
ST3GAL6−AS1
MIR548G
ST3GAL6
DCBLD2
Position on chr3 (Mb)

FEV_1_multiethnic_ancestry_rs111898810

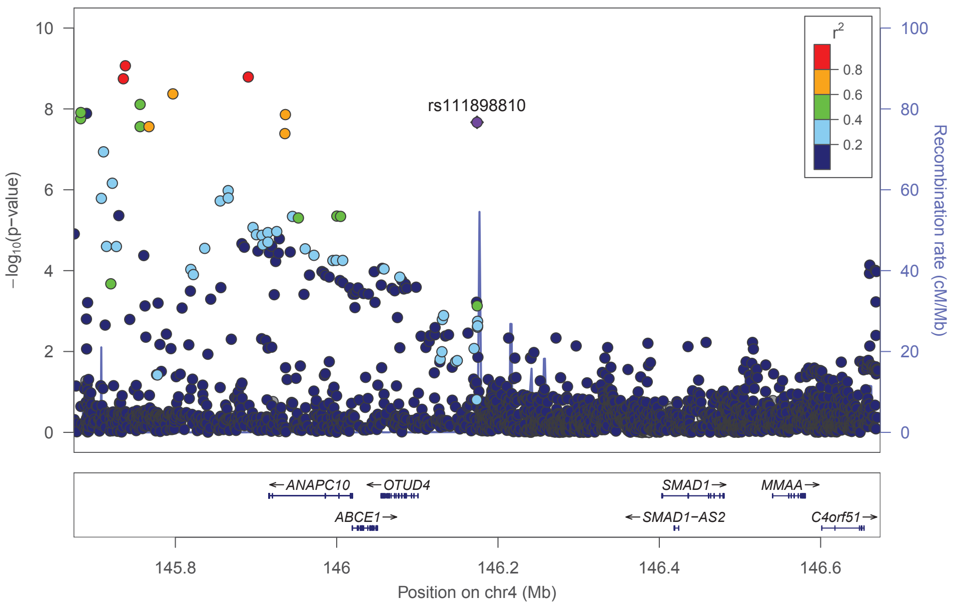

FEV_1_multiethnic_ancestry_rs9407640

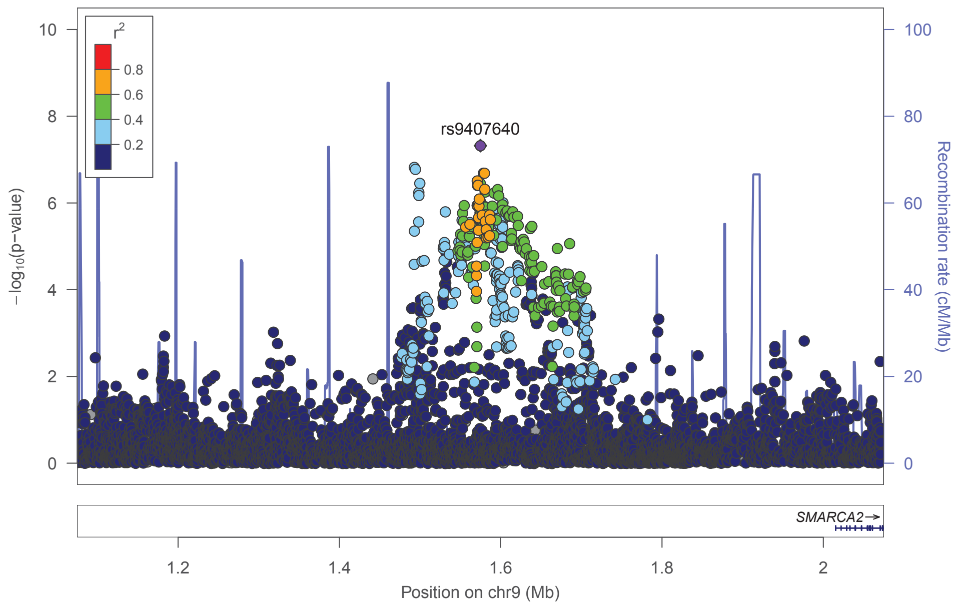

FEV_1_multiethnic_ancestry_rs7899503

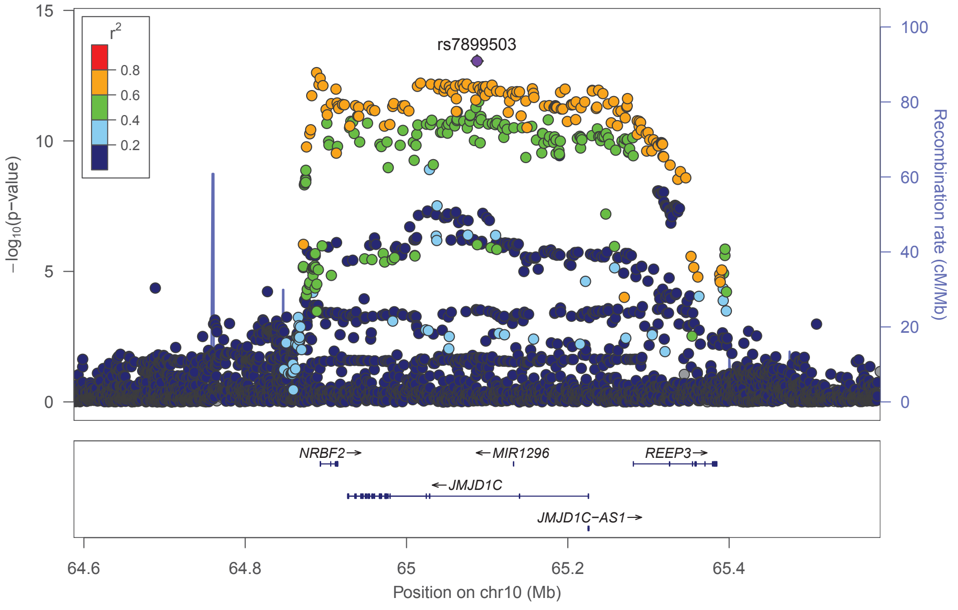

FEV_1_multiethnic_ancestry_rs772920

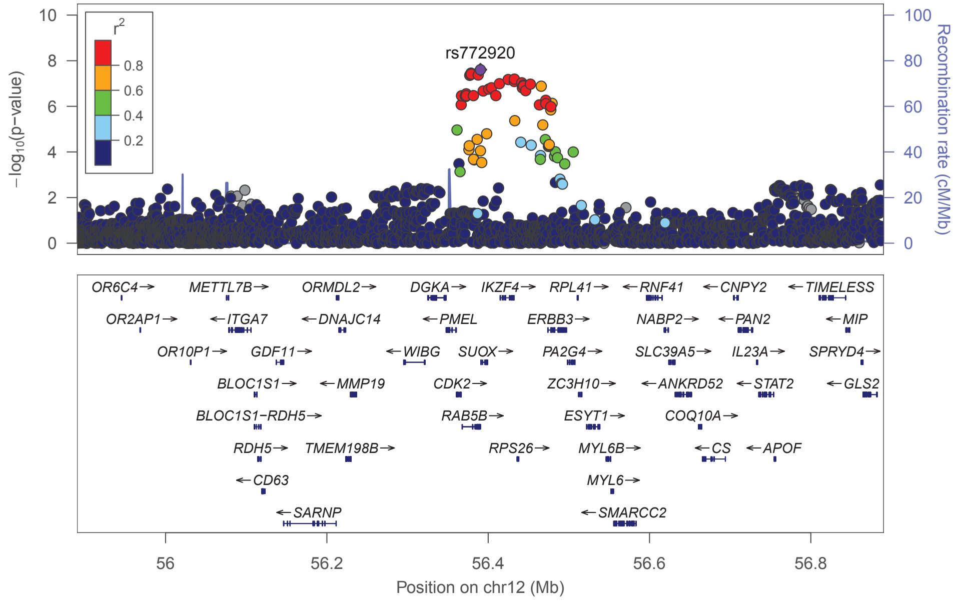

FEV$_1$_multiethnic_ancestry_rs11057793

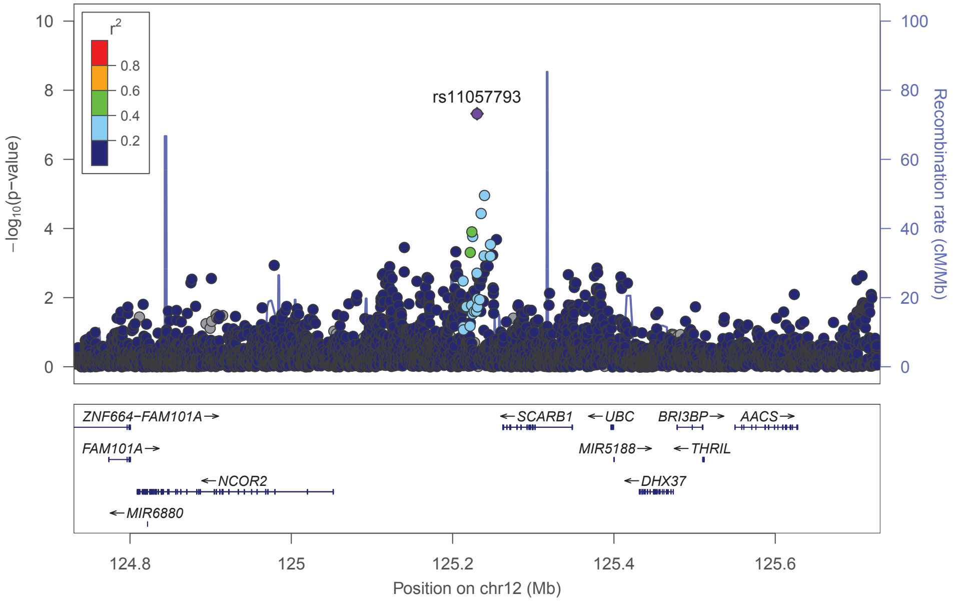

FEV$_1$_multiethnic_ancestry_rs62070631

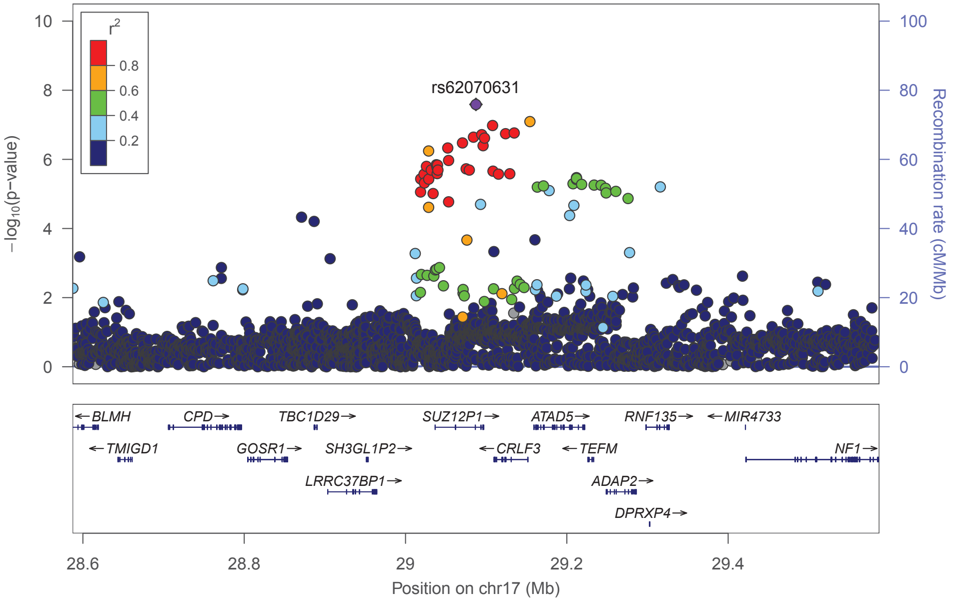

FEV$_1$_multiethnic_ancestry_rs186806998

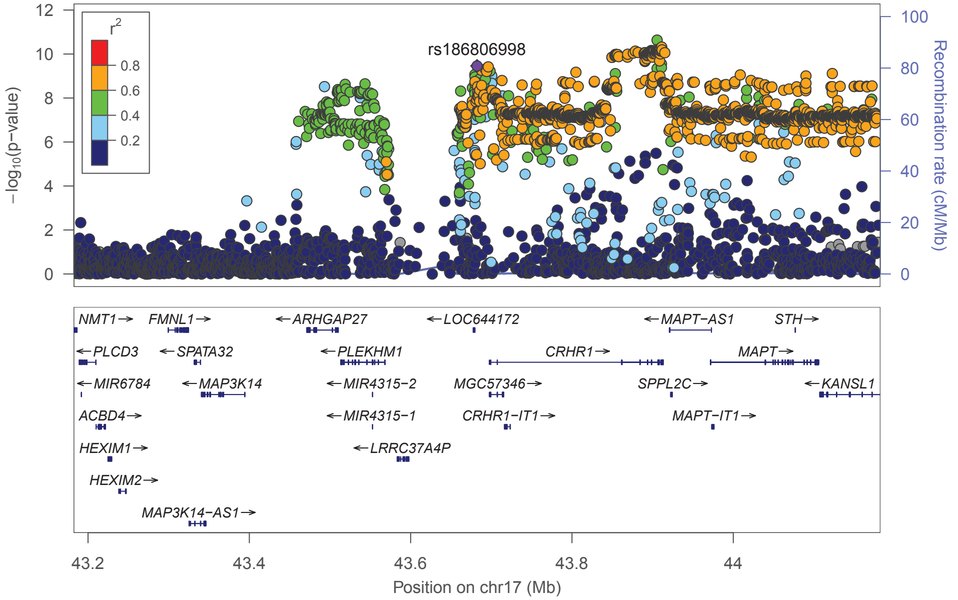

FEV$_1$_multiethnic_ancestry_rs199525

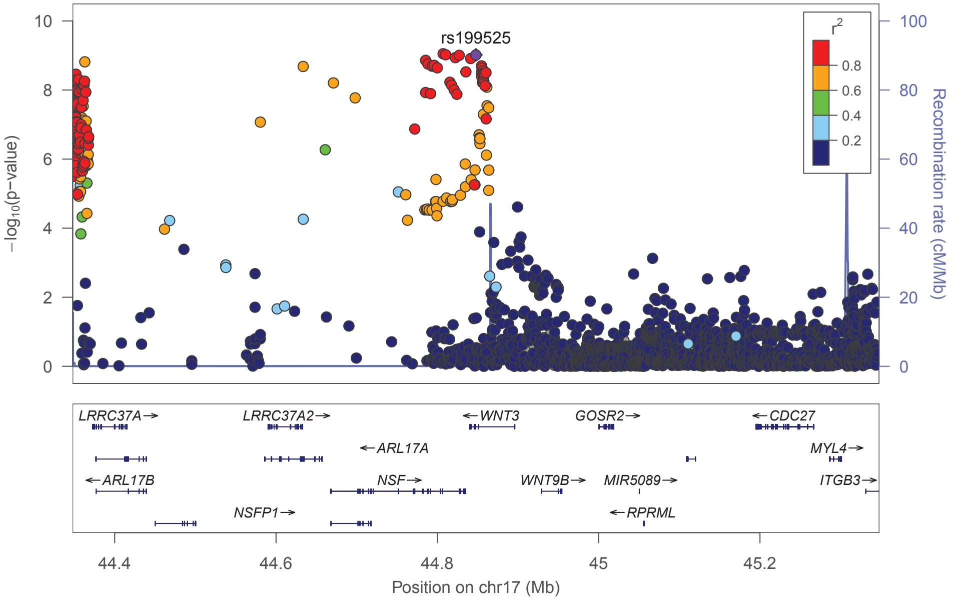

FEV$_1$_multiethnic_ancestry_rs513953

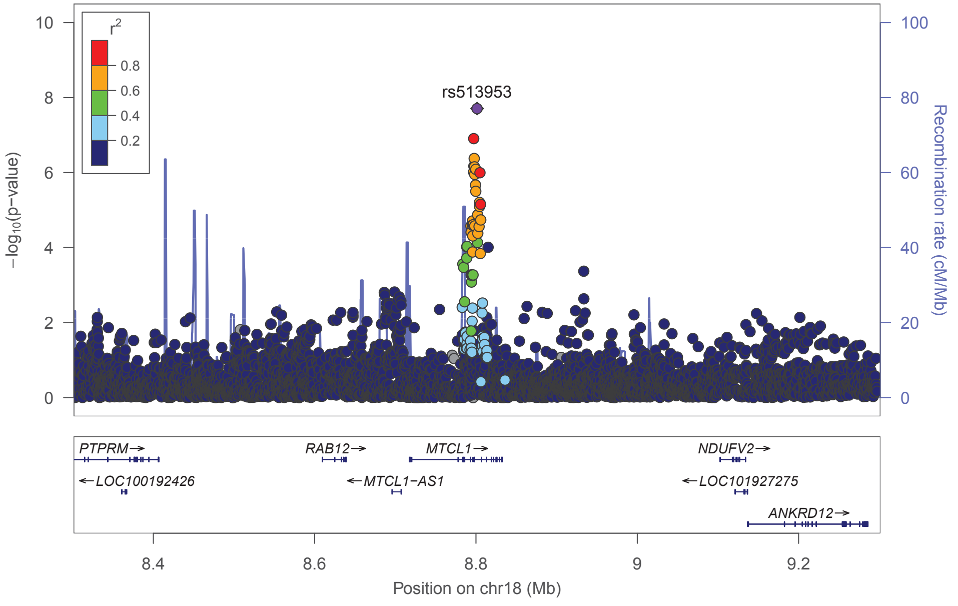

FEV$_1$_multiethnic_ancestry_rs7243351

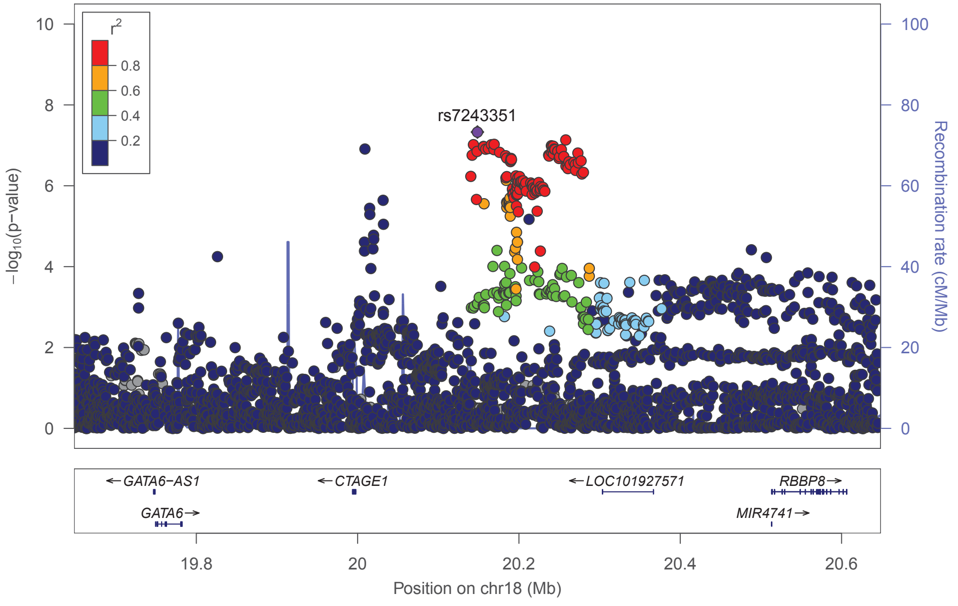

FEV$_1$_multiethnic_ancestry_rs6138639

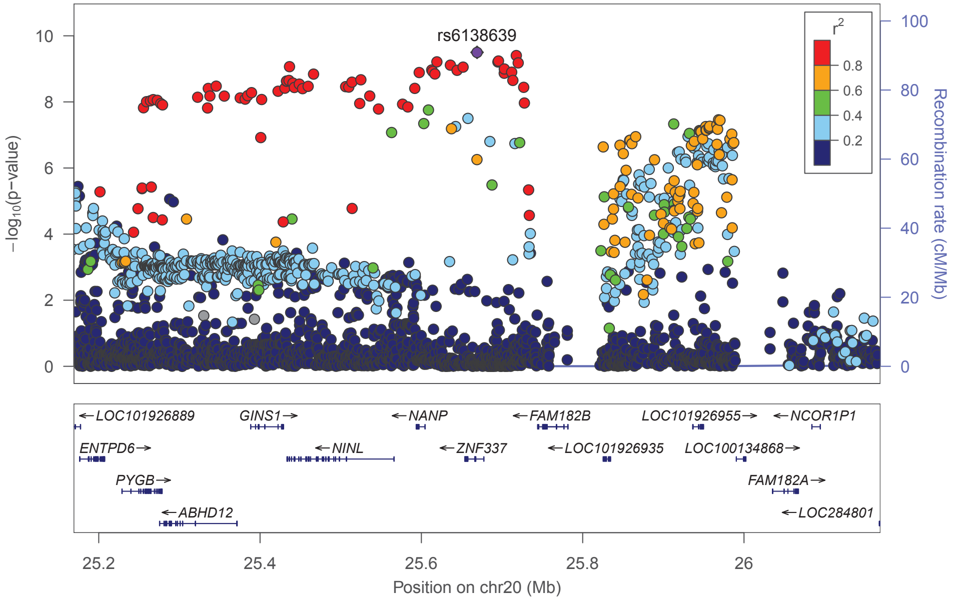

FEV$_1$_multiethnic_ancestry_rs1737889

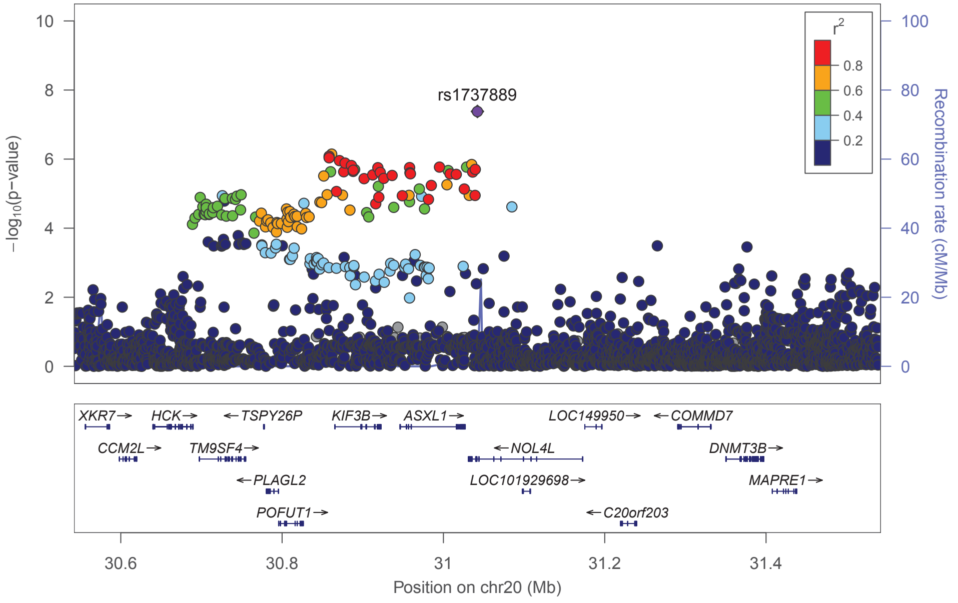

B.

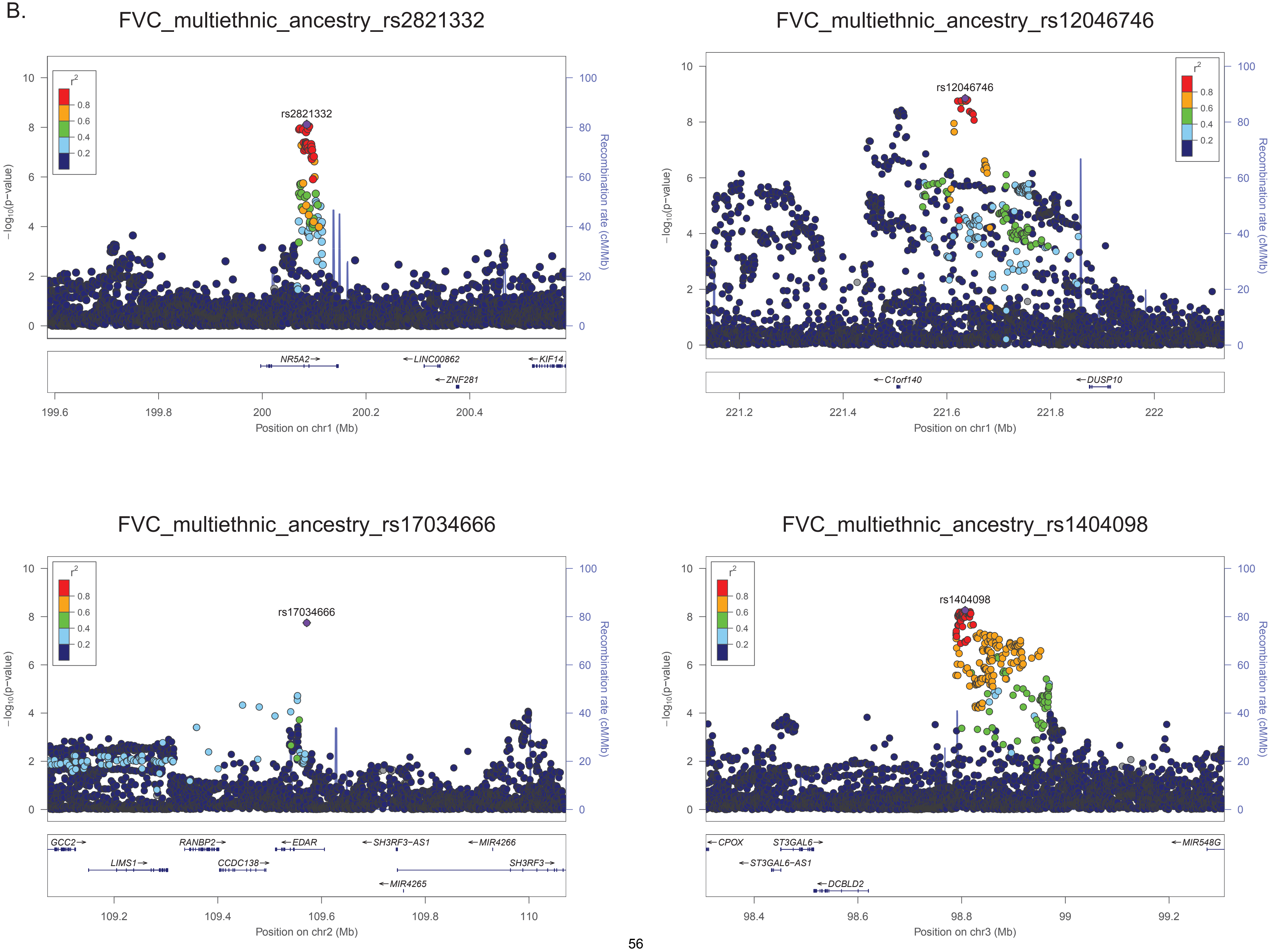

FVC_multiethnic_ancestry_rs2821332
rs2821332
NR5A2
LINC00862
KIF14
ZNF281
Position on chr1 (Mb)
FVC_multiethnic_ancestry_rs12046746
rs12046746
C1orf140
DUSP10
Position on chr1 (Mb)
FVC_multiethnic_ancestry_rs17034666
rs17034666
GCC2
RANBP2
EDAR
SH3RF3-AS1
MIR4266
LIMS1
CCDC138
SH3RF3
MIR4265
Position on chr2 (Mb)
FVC_multiethnic_ancestry_rs1404098
rs1404098
CPOX
ST3GAL6
MIR548G
ST3GAL6-AS1
DCBLD2
Position on chr3 (Mb)
−log10(p−value)
Recombination rate (cM/Mb)
r2

FVC_multiethnic_ancestry_rs72776440

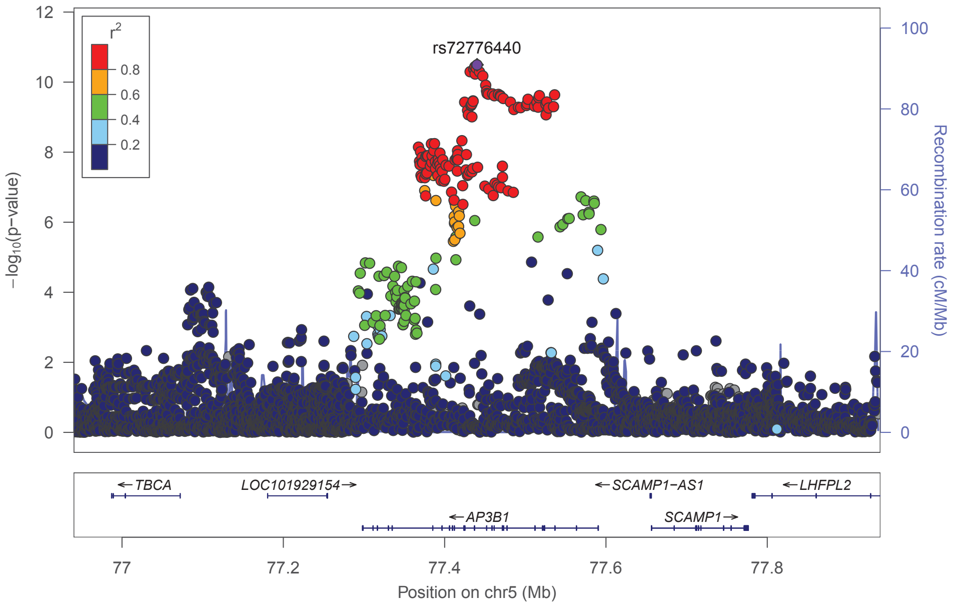

FVC_multiethnic_ancestry_rs11759026

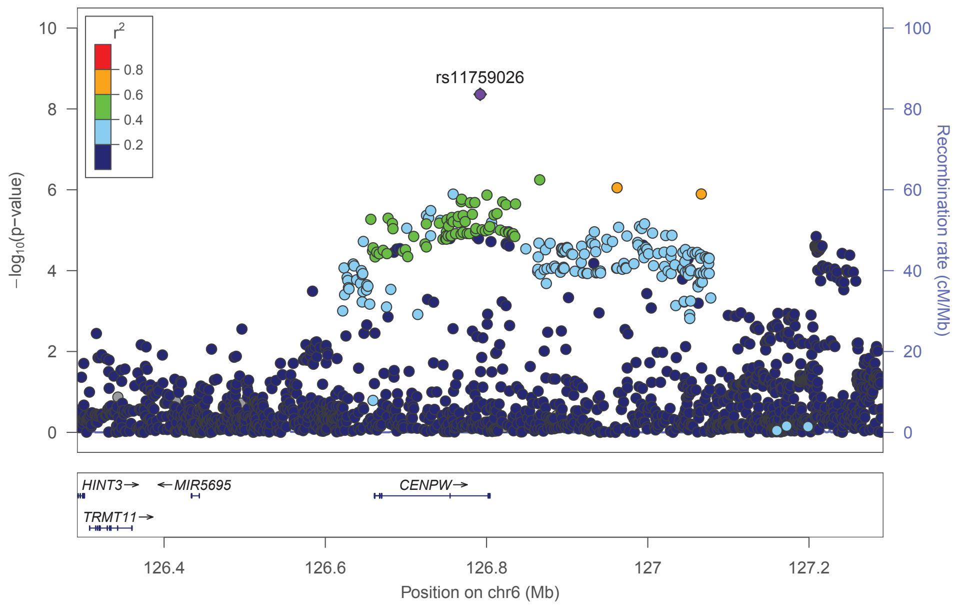

FVC_multiethnic_ancestry_rs55905169

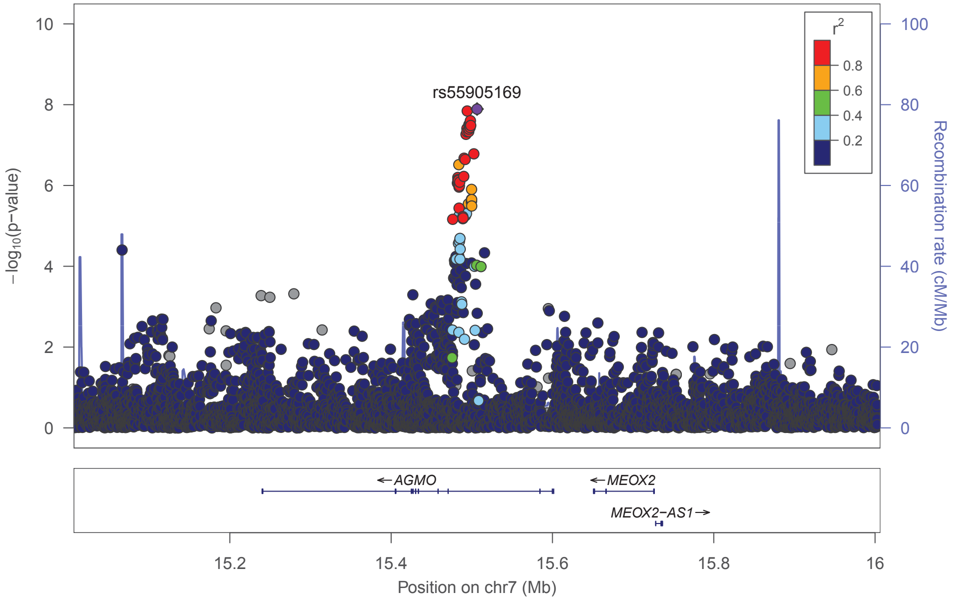

FVC_multiethnic_ancestry_rs9407640

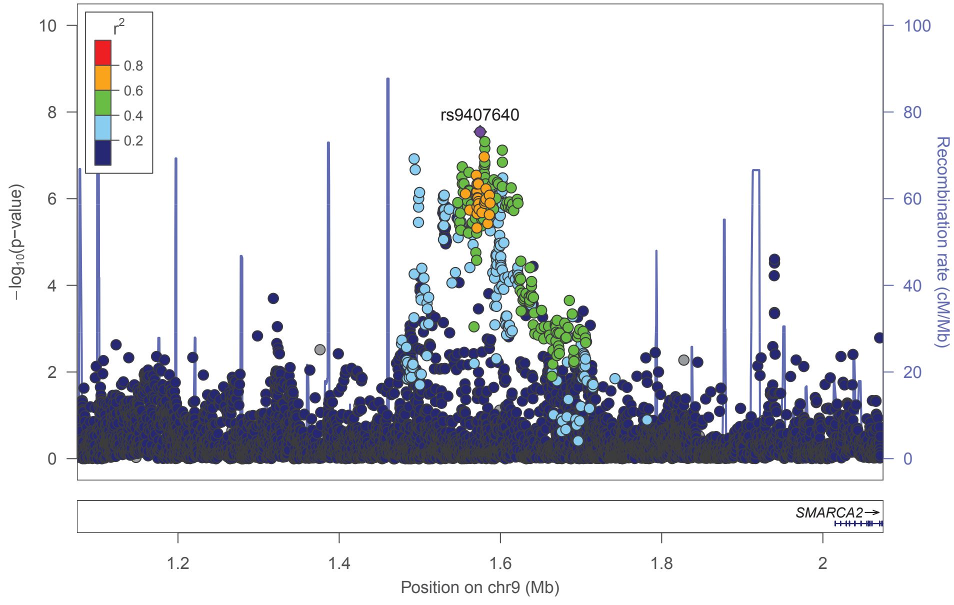

FVC_multiethnic_ancestry_rs73025192

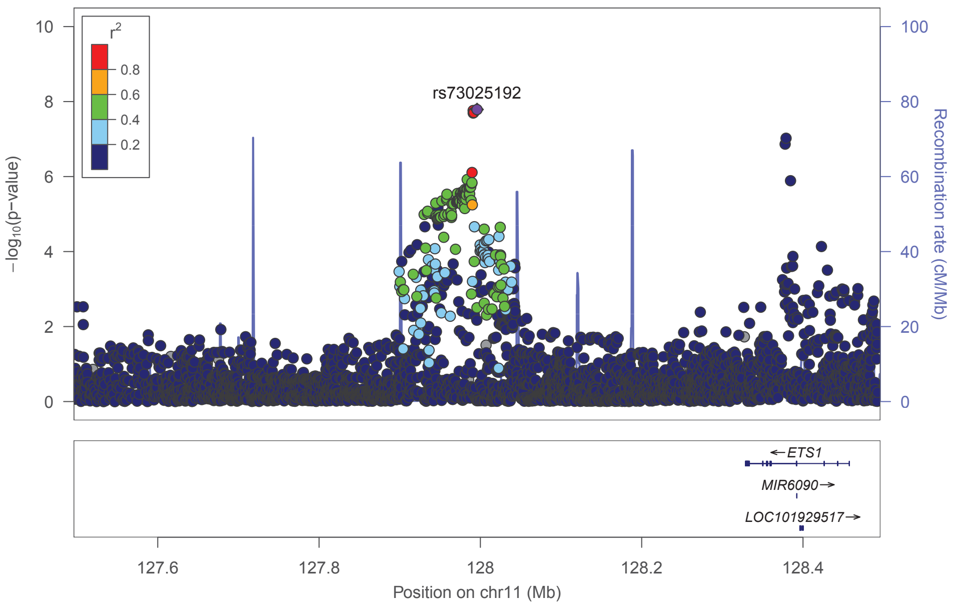

FVC_multiethnic_ancestry_rs7971039

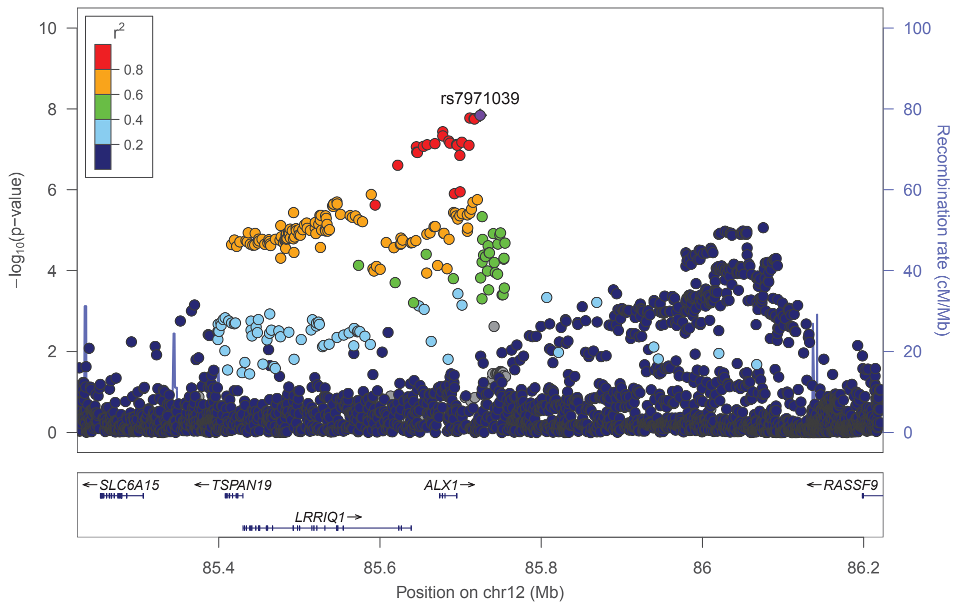

FVC_multiethnic_ancestry_rs11107184

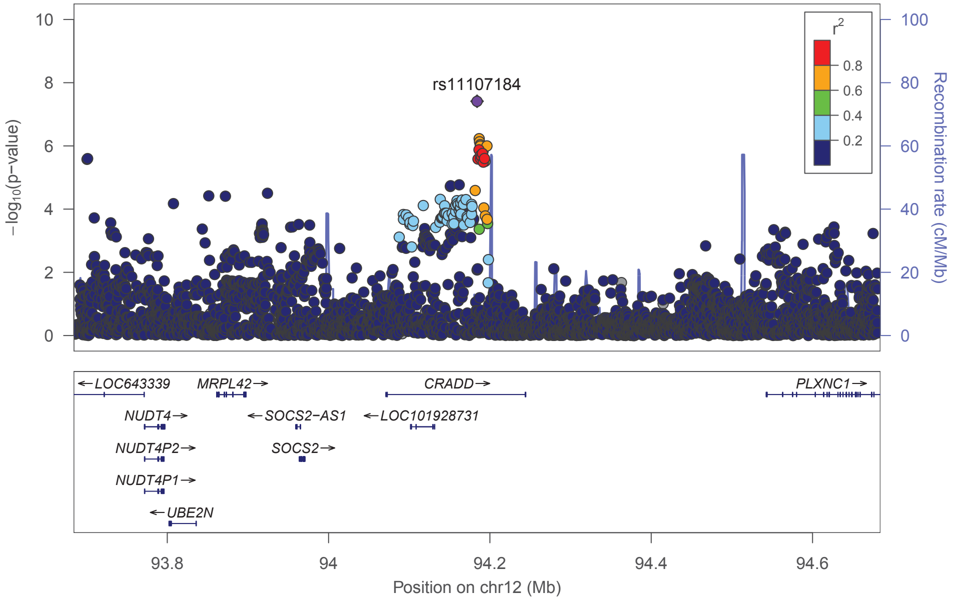

FVC_multiethnic_ancestry_rs10859698

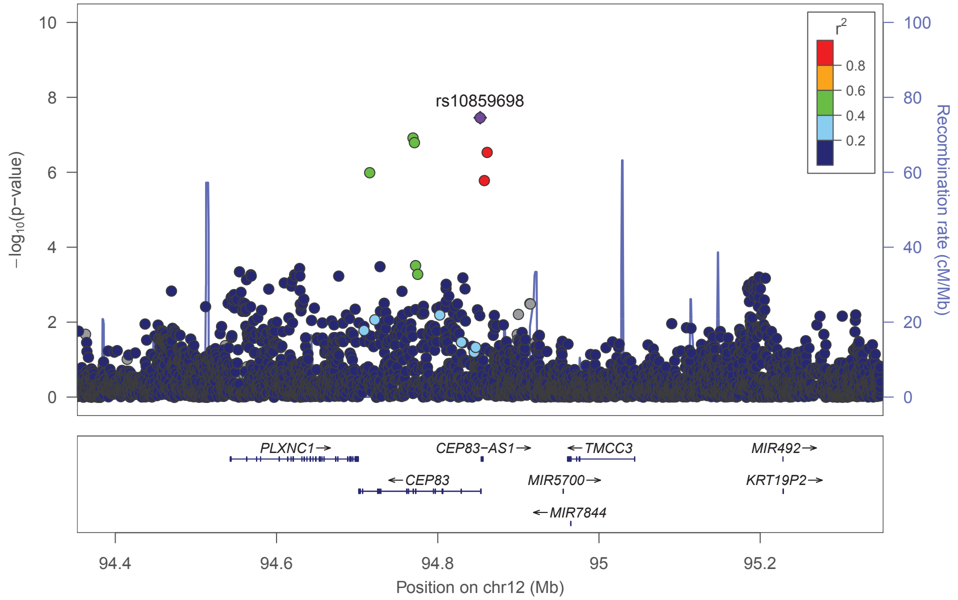

FVC_multiethnic_ancestry_rs4775429

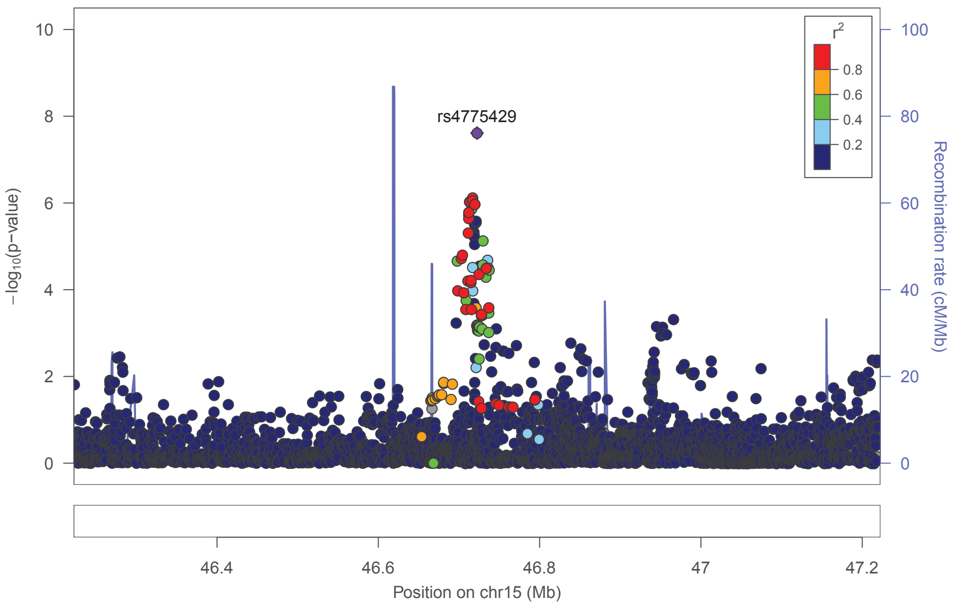

FVC_multiethnic_ancestry_rs8025774

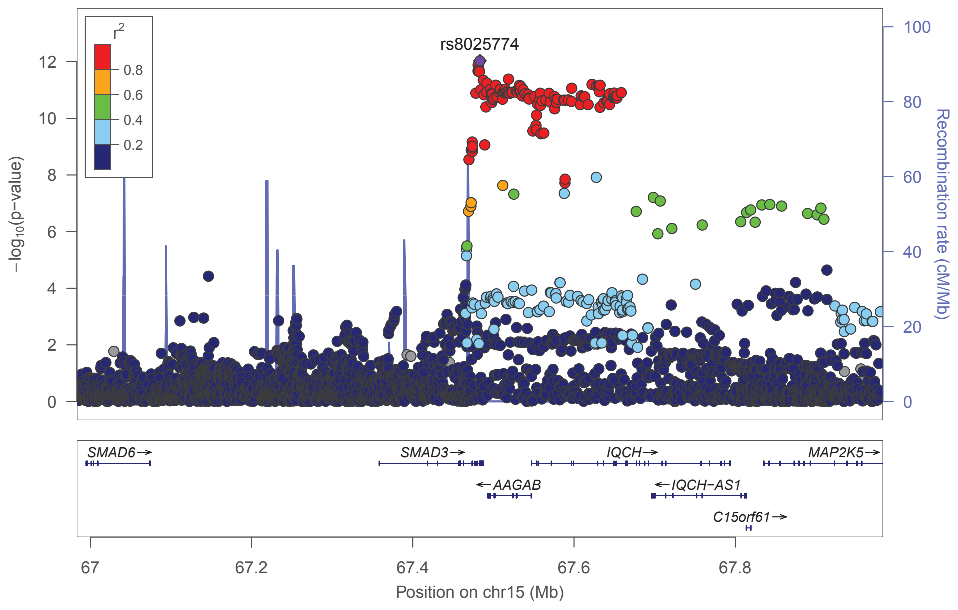

FVC_multiethnic_ancestry_rs3973397

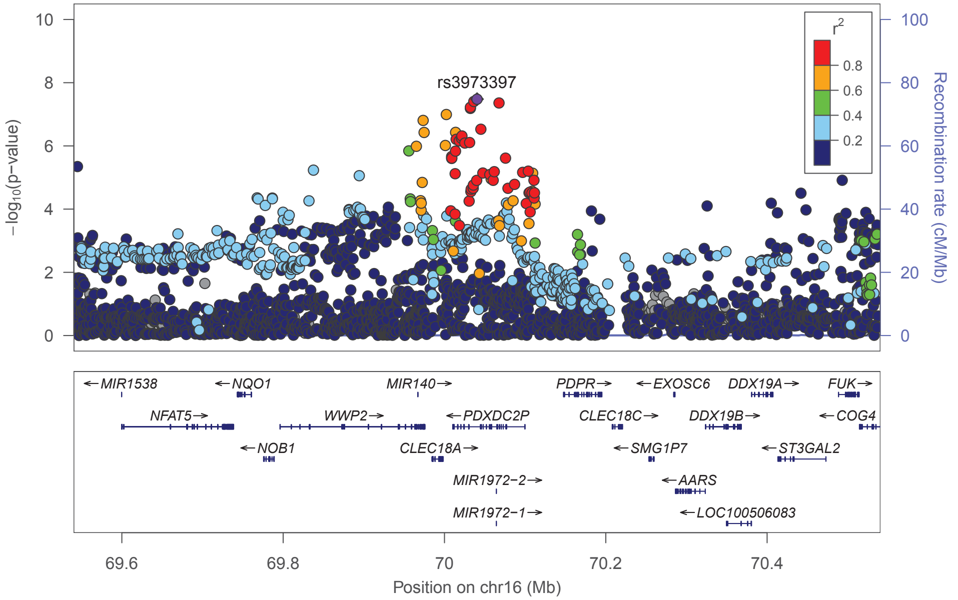

FVC_multiethnic_ancestry_rs55771535

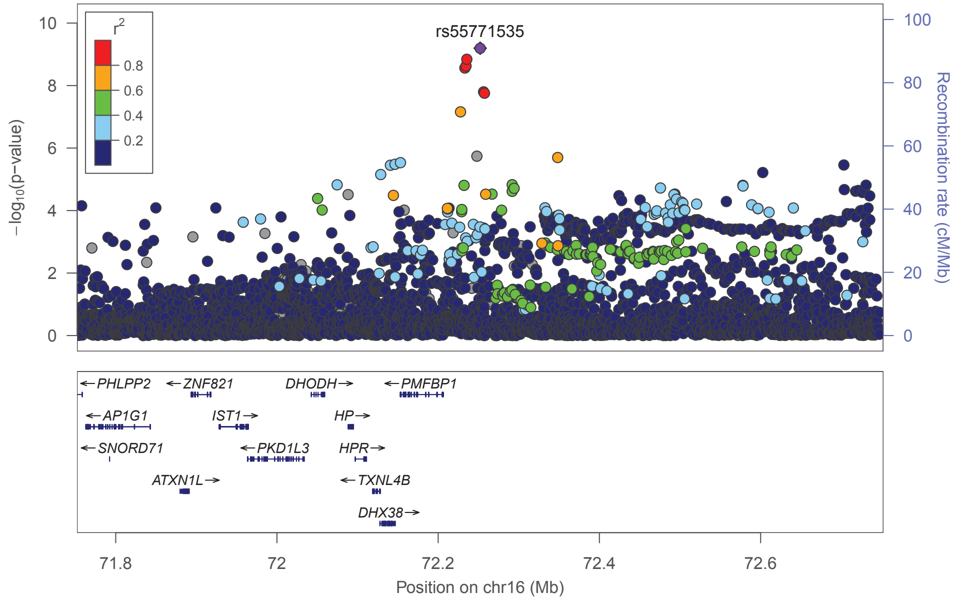

FVC_multiethnic_ancestry_rs8067511

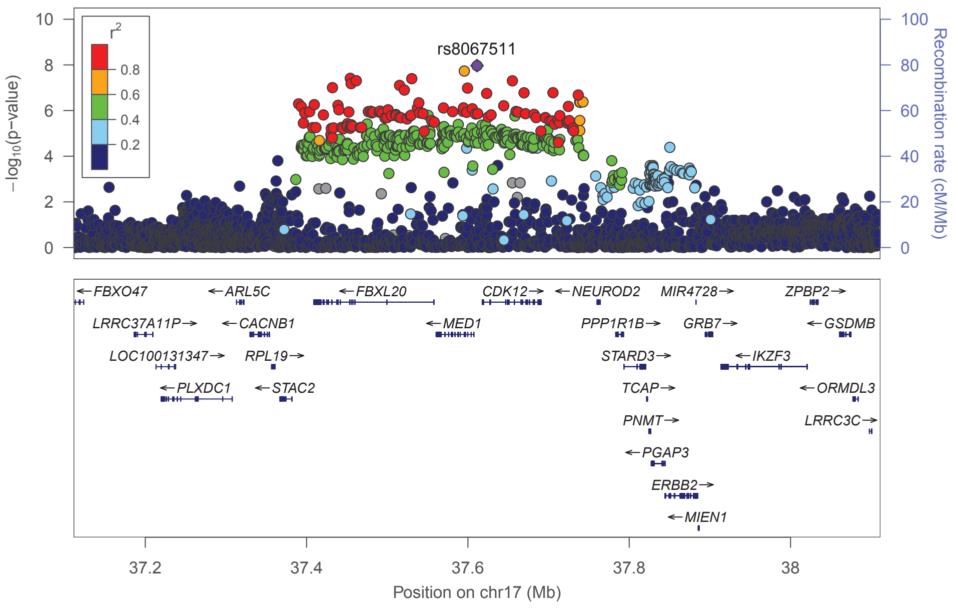

FVC_multiethnic_ancestry_rs150741403

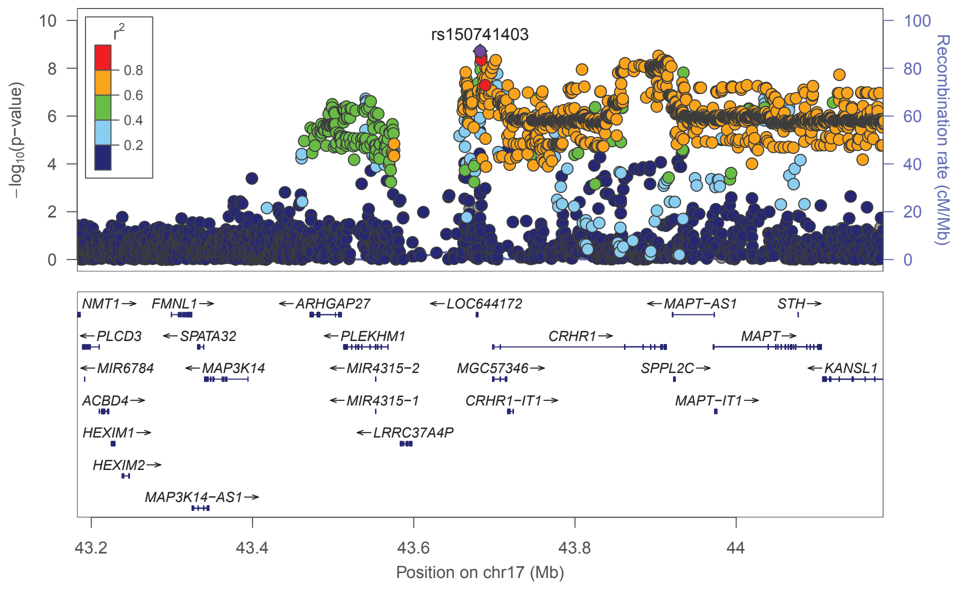

FVC_multiethnic_ancestry_rs199525

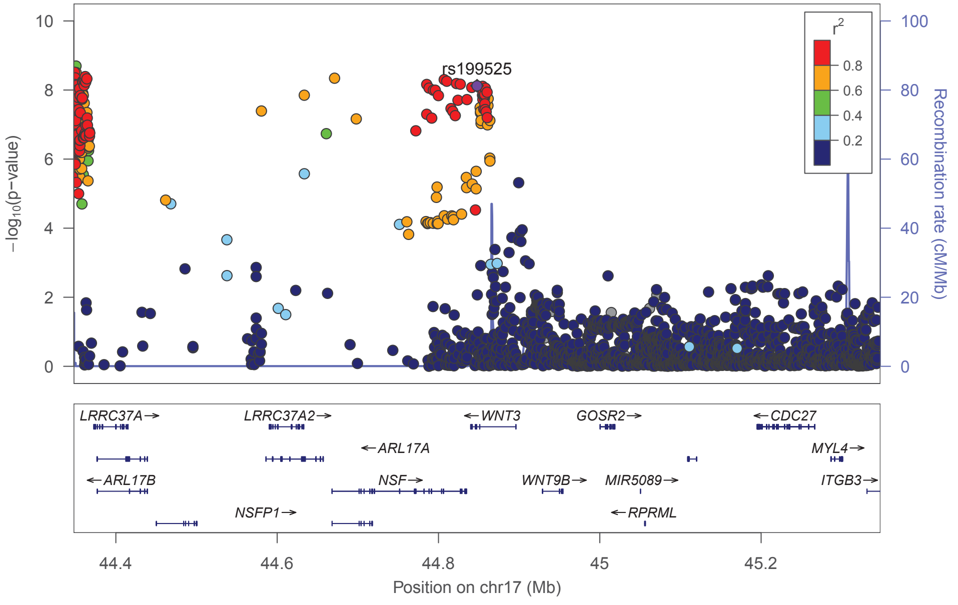

FVC_multiethnic_ancestry_rs7238093

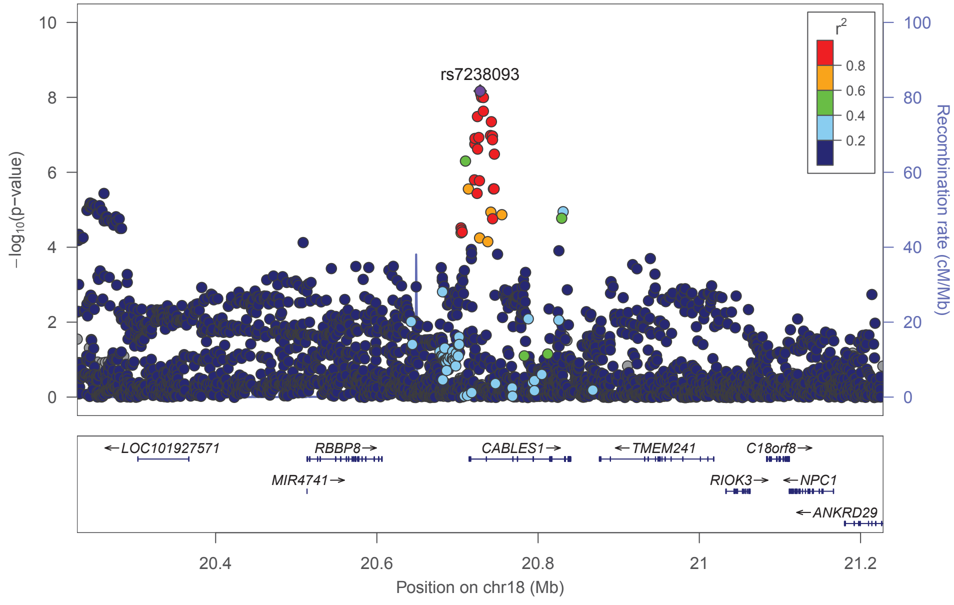

FVC_multiethnic_ancestry_rs8089865

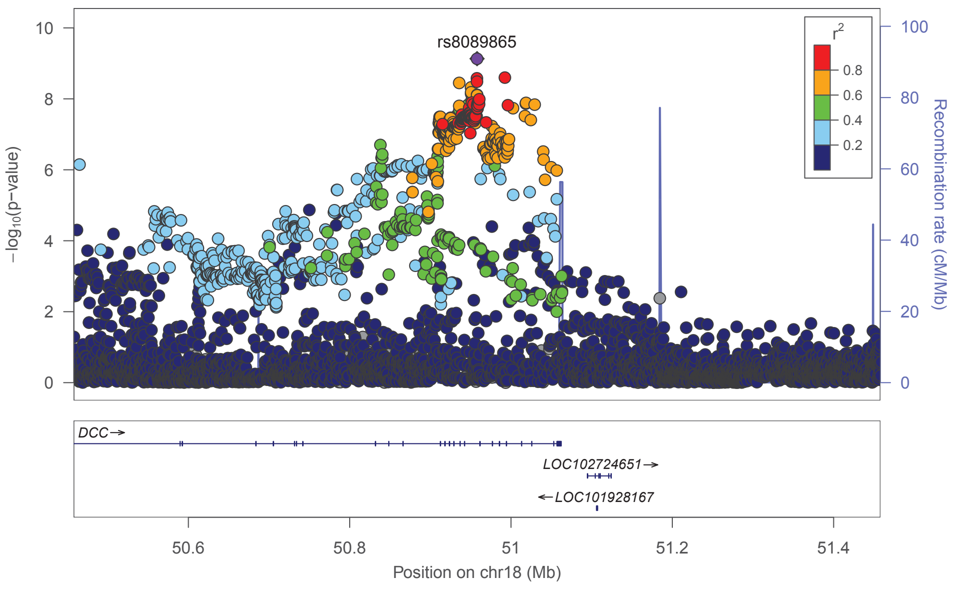

C.

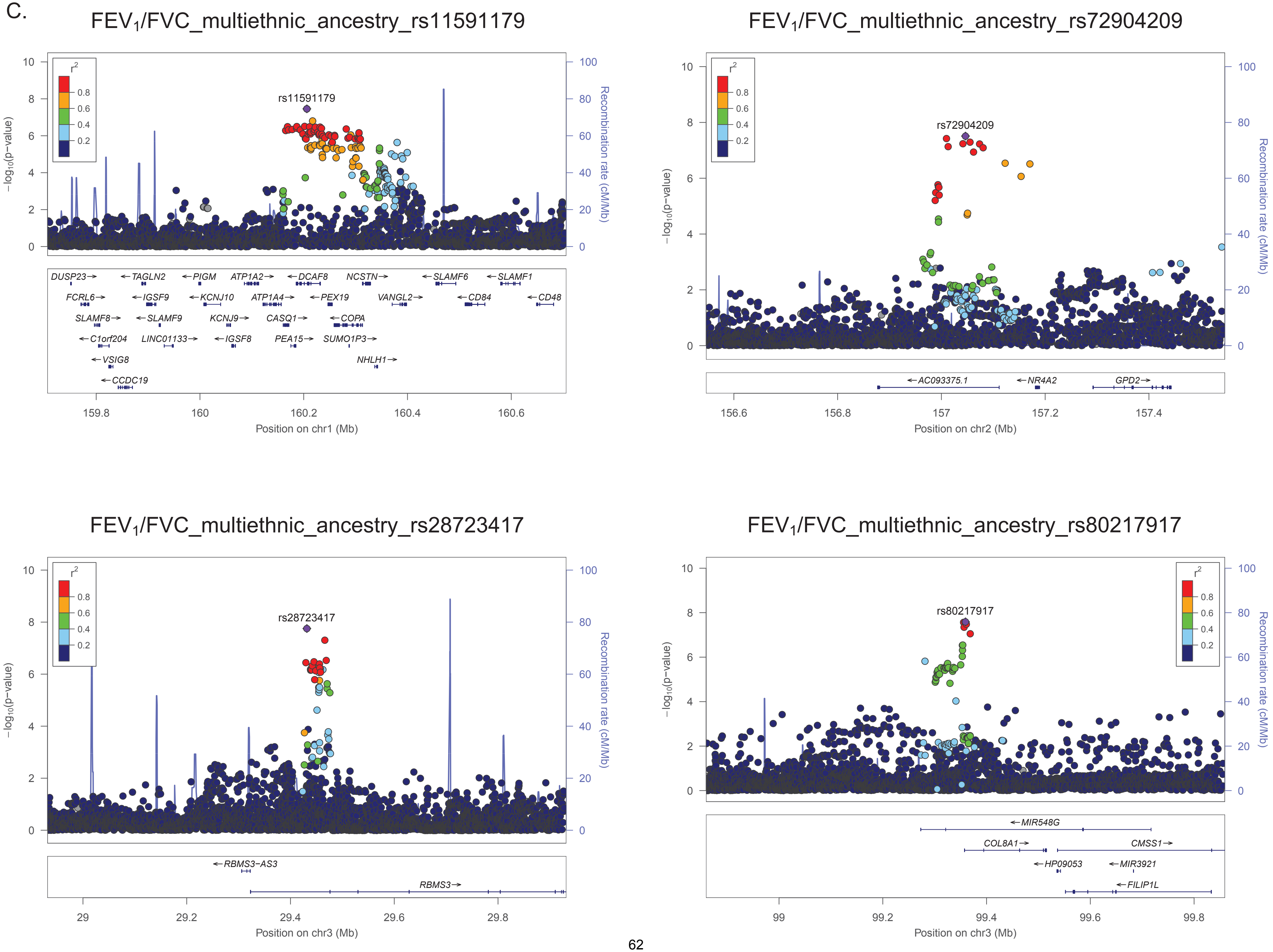
FEV$_1$/FVC_multiethnic_ancestry_rs11591179
rs11591179
−log10(p−value)
Recombination rate (cM/Mb)
r2
DUSP23 TAGLN2 PIGM ATP1A2 DCAF8 NCSTN SLAMF6 SLAMF1
FCRL6 IGSF9 KCNJ10 ATP1A4 PEX19 VANGL2 CD84 CD48
SLAMF8 SLAMF9 KCNJ9 CASQ1 COPA
C1orf204 LINC01133 IGSF8 PEA15 SUMO1P3
VSIG8 NHLH1
CCDC19
Position on chr1 (Mb)
FEV$_1$/FVC_multiethnic_ancestry_rs72904209
rs72904209
AC093375.1 NR4A2 GPD2
Position on chr2 (Mb)
FEV$_1$/FVC_multiethnic_ancestry_rs28723417
rs28723417
RBMS3-AS3
RBMS3
Position on chr3 (Mb)
FEV$_1$/FVC_multiethnic_ancestry_rs80217917
rs80217917
MIR548G
COL8A1 CMSS1
HP09053 MIR3921
FILIP1L
Position on chr3 (Mb)

FEV$_1$/FVC_multiethnic_ancestry_rs28520091

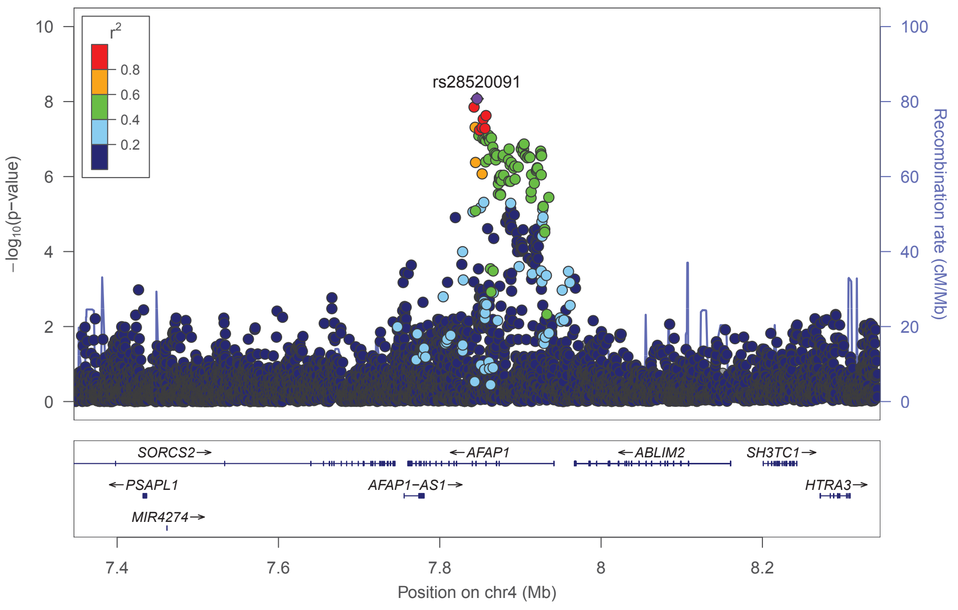

FEV$_1$/FVC multiethnic_ancestry_rs9350408

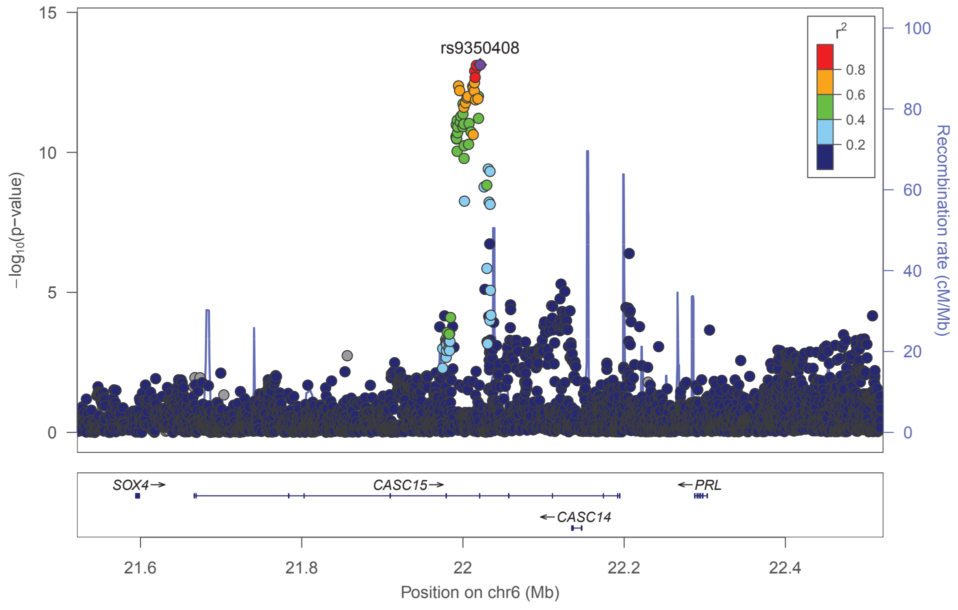

FEV$_1$/VC_multiethnic_ancestry_rs10965947

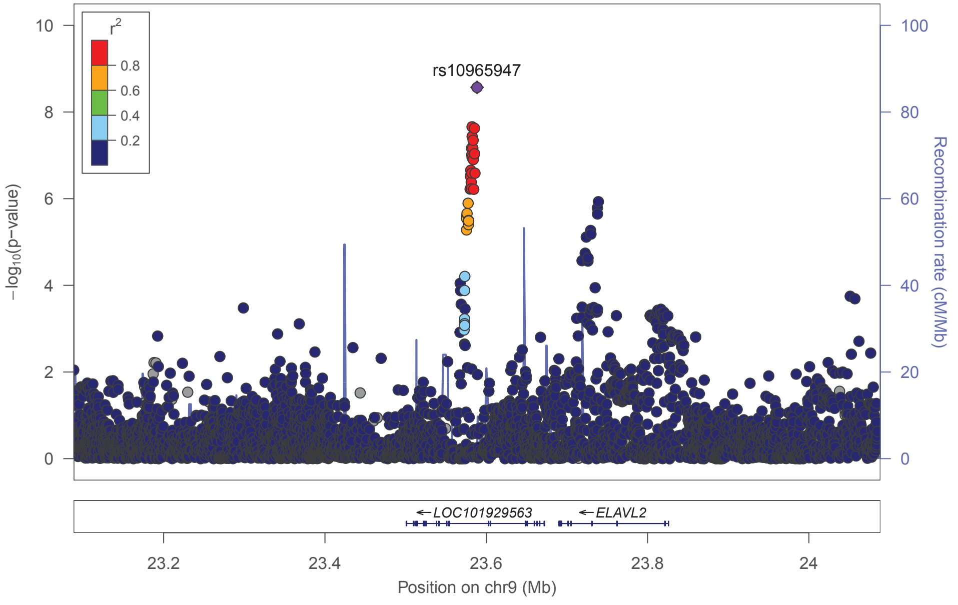

FEV$_1$/FVC multiethnic_ancestry_rs2451951

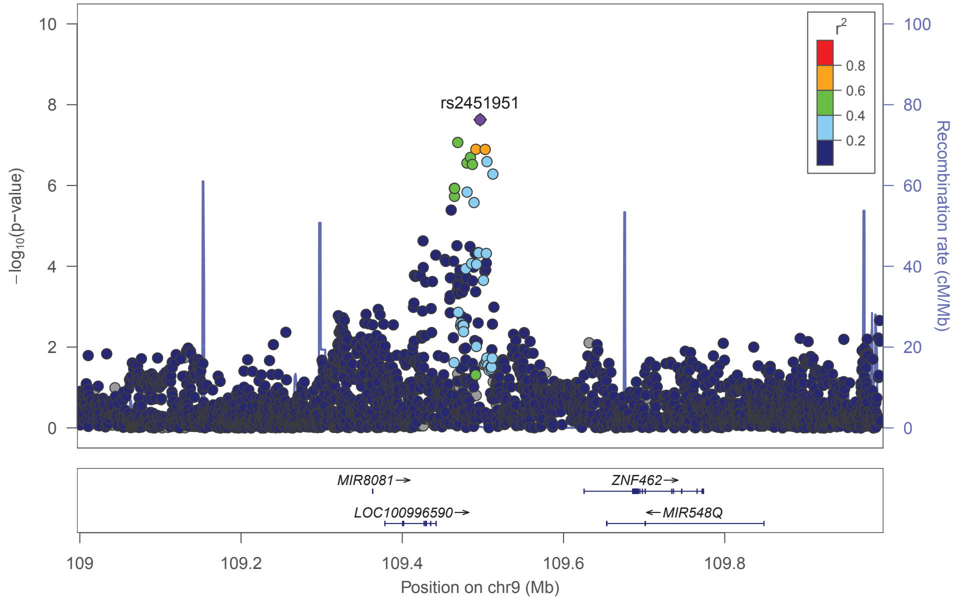

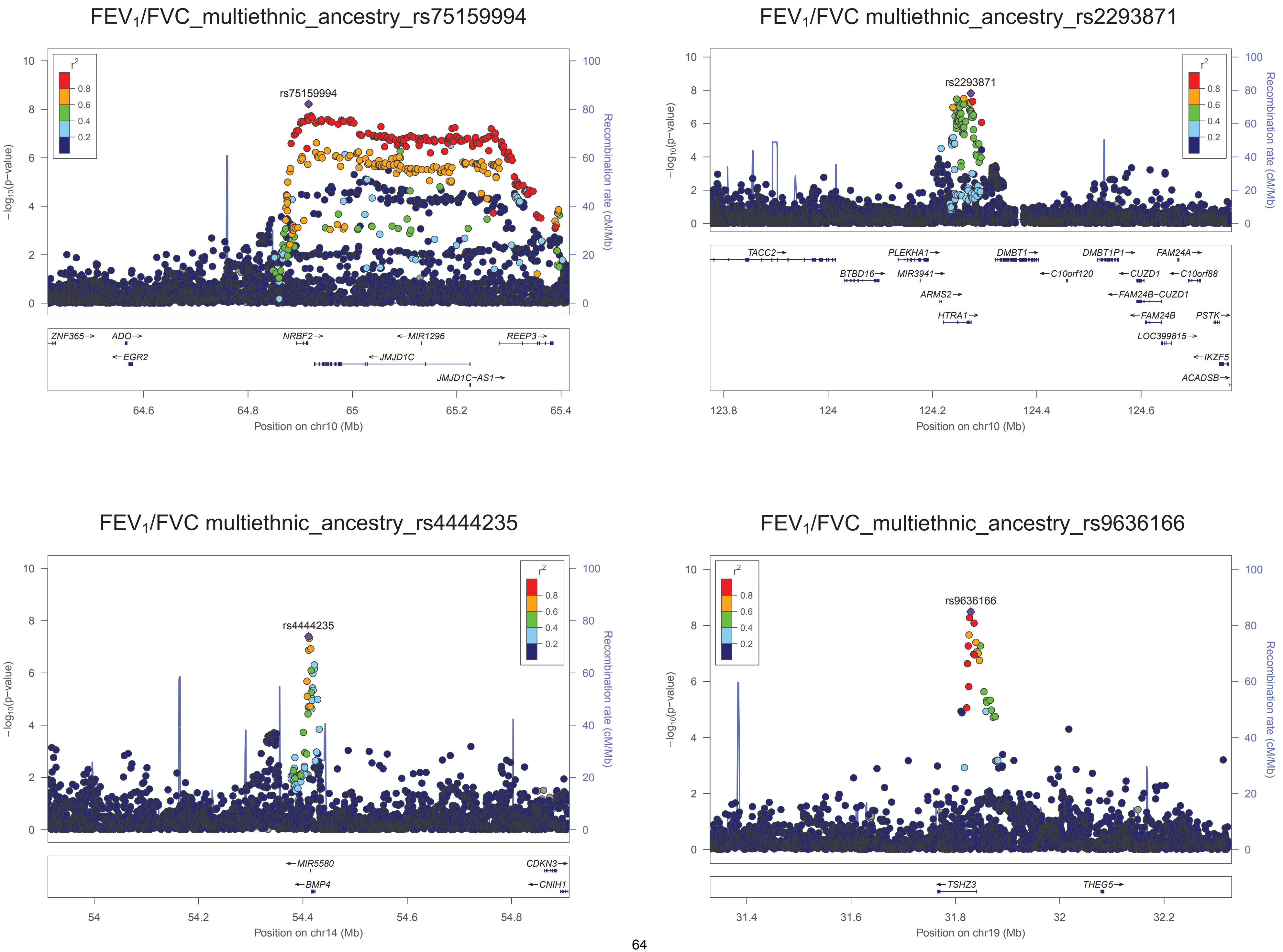
FEV1/FVC_multiethnic_ancestry_rs75159994
rs75159994
−log10(p-value)
Recombination rate (cM/Mb)
r2
0.8
0.6
0.4
0.2
ZNF365→
ADO→
←EGR2
NRBF2→
←MIR1296
←JMJD1C
JMJD1C−AS1→
REEP3→
64.6
64.8
65
65.2
65.4
Position on chr10 (Mb)
FEV1/FVC multiethnic_ancestry_rs2293871
rs2293871
TACC2→
PLEKHA1→
DMBT1→
DMBT1P1→
FAM24A→
BTBD16→
MIR3941→
←C10orf120
←CUZD1
←C10orf88
ARMS2→
←FAM24B−CUZD1
HTRA1→
←FAM24B
PSTK→
LOC399815→
←IKZF5
ACADSB→
123.8
124
124.2
124.4
124.6
Position on chr10 (Mb)
FEV1/FVC multiethnic_ancestry_rs4444235
rs4444235
←MIR5580
CDKN3→
←BMP4
←CNIH1
54
54.2
54.4
54.6
54.8
Position on chr14 (Mb)
FEV1/FVC_multiethnic_ancestry_rs9636166
rs9636166
←TSHZ3
THEG5→
31.4
31.6
31.8
32
32.2
Position on chr19 (Mb)

FEV$_1$/FVC_multiethnic_ancestry_rs4820216

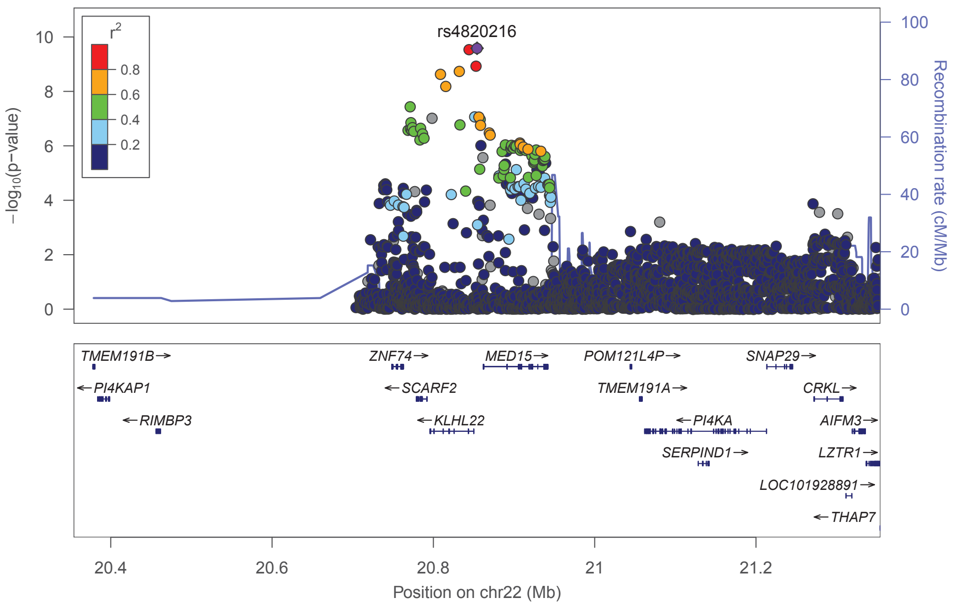

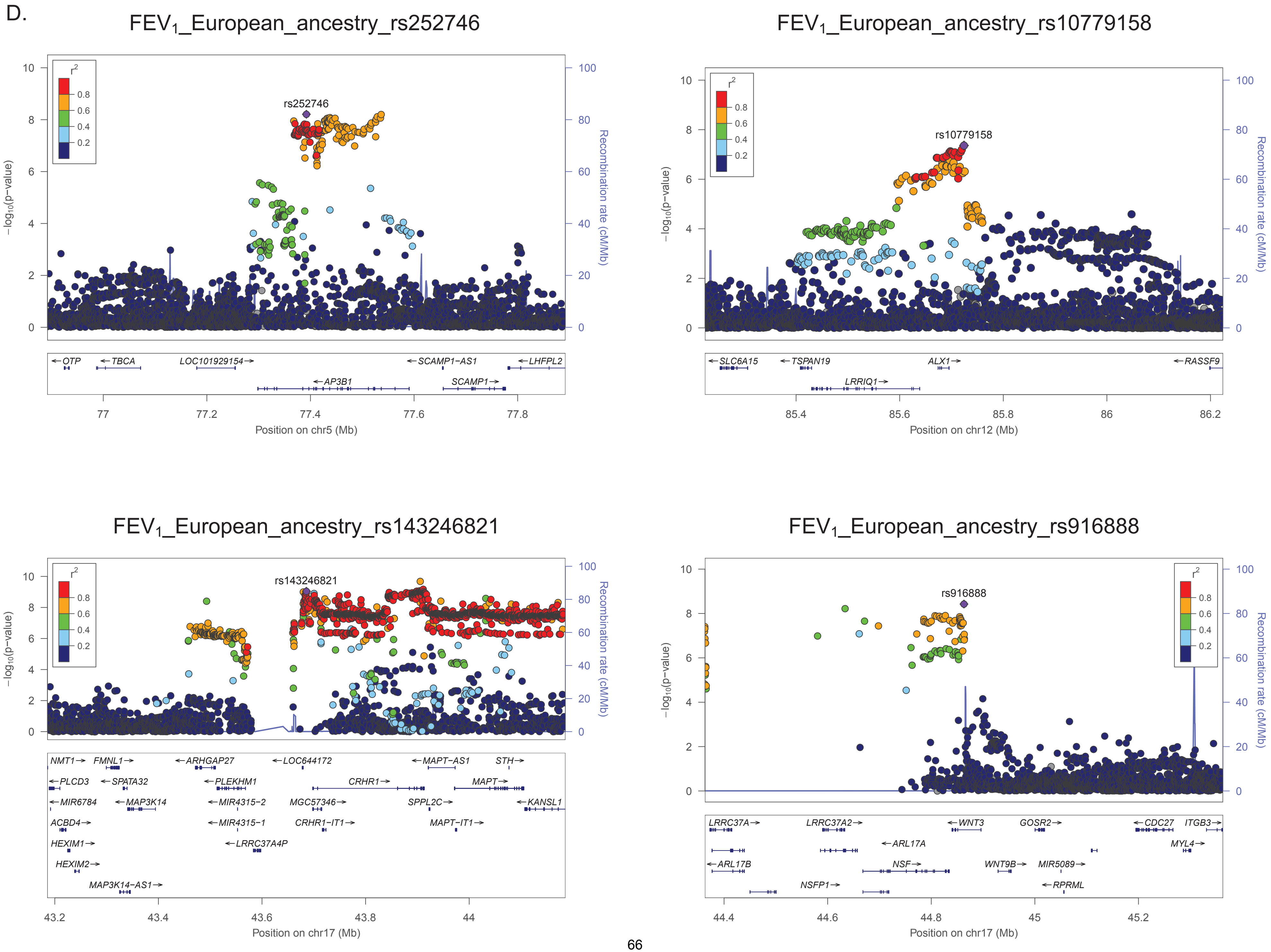
D.
FEV1_European_ancestry_rs252746
rs252746
r2
0.8
0.6
0.4
0.2
−log10(p−value)
Recombination rate (cM/Mb)
OTP
TBCA
LOC101929154
SCAMP1−AS1
LHFPL2
AP3B1
SCAMP1
77
77.2
77.4
77.6
77.8
Position on chr5 (Mb)
FEV1_European_ancestry_rs10779158
rs10779158
SLC6A15
TSPAN19
ALX1
RASSF9
LRRIQ1
85.4
85.6
85.8
86
86.2
Position on chr12 (Mb)
FEV1_European_ancestry_rs143246821
rs143246821
NMT1
FMNL1
ARHGAP27
LOC644172
MAPT−AS1
STH
PLCD3
SPATA32
PLEKHM1
CRHR1
MAPT
MIR6784
MAP3K14
MIR4315−2
MGC57346
SPPL2C
KANSL1
ACBD4
MIR4315−1
CRHR1−IT1
MAPT−IT1
HEXIM1
LRRC37A4P
HEXIM2
MAP3K14−AS1
43.2
43.4
43.6
43.8
44
Position on chr17 (Mb)
FEV1_European_ancestry_rs916888
rs916888
LRRC37A
LRRC37A2
WNT3
GOSR2
CDC27
ITGB3
ARL17A
MYL4
ARL17B
NSF
WNT9B
MIR5089
NSFP1
RPRML
44.4
44.6
44.8
45
45.2
Position on chr17 (Mb)

E.

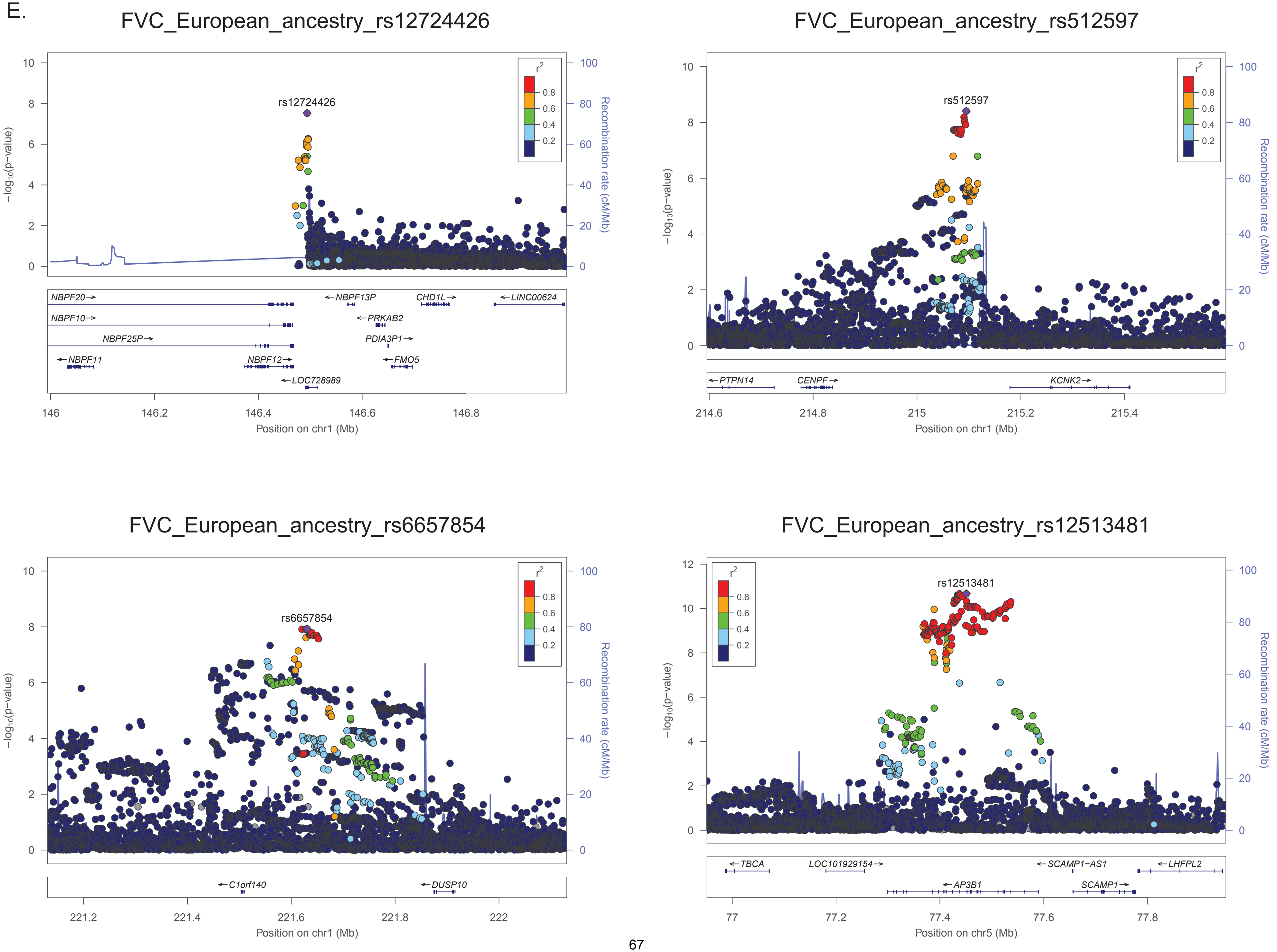
FVC_European_ancestry_rs12724426
rs12724426
−log10(p−value)
Recombination rate (cM/Mb)
r2
0.8
0.6
0.4
0.2
NBPF20→
NBPF10→
NBPF25P→
←NBPF11
NBPF12→
←LOC728989
←NBPF13P
←PRKAB2
PDIA3P1→
←FMO5
CHD1L→
←LINC00624
Position on chr1 (Mb)
FVC_European_ancestry_rs512597
rs512597
←PTPN14
CENPF→
KCNK2→
Position on chr1 (Mb)
FVC_European_ancestry_rs6657854
rs6657854
←C1orf140
←DUSP10
Position on chr1 (Mb)
FVC_European_ancestry_rs12513481
rs12513481
←TBCA
LOC101929154→
←AP3B1
←SCAMP1−AS1
SCAMP1→
←LHFPL2
Position on chr5 (Mb)

FVC_European_ancestry_rs771924

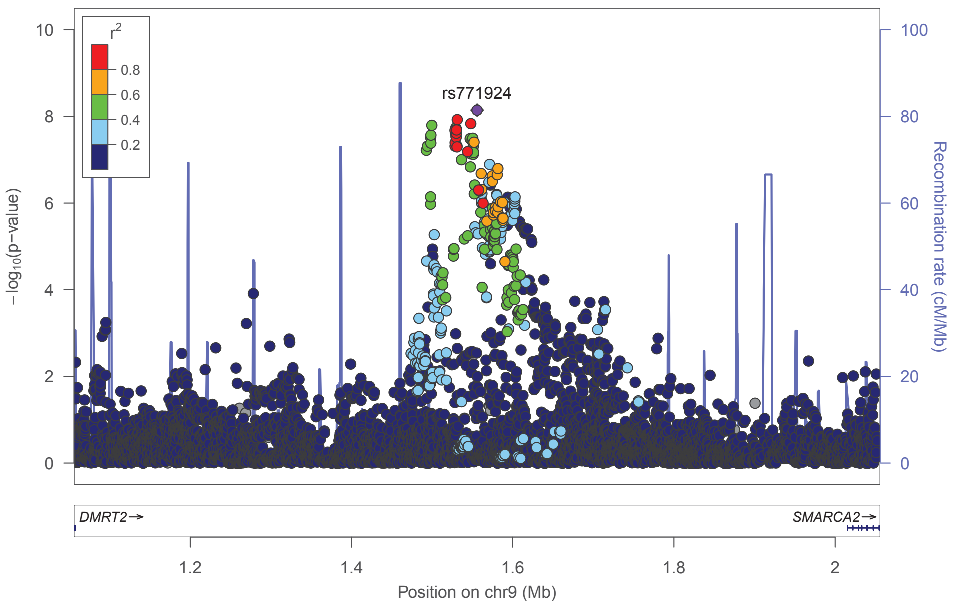

FVC_European_ancestry_rs10779158

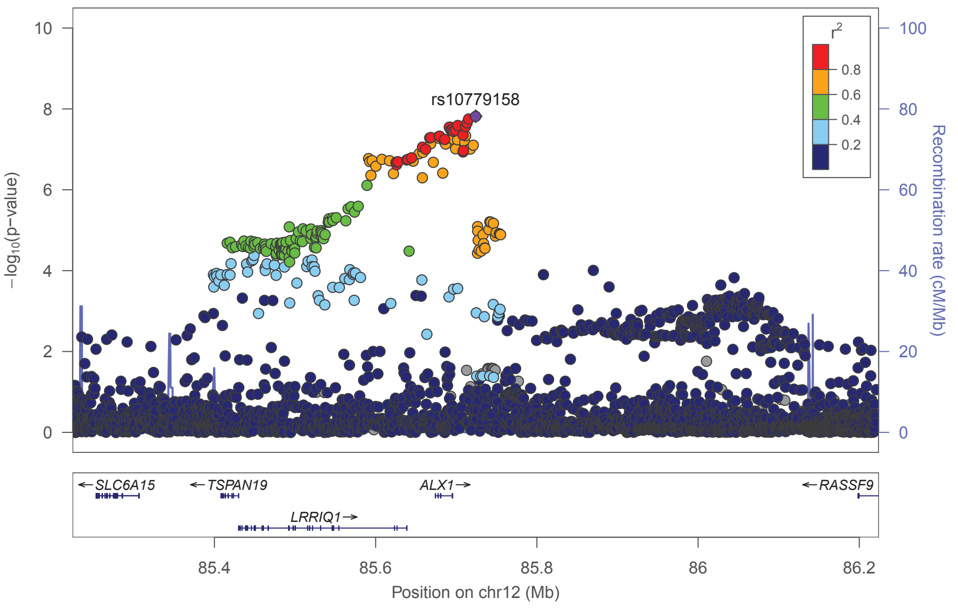

FVC_European_ancestry_rs8089865

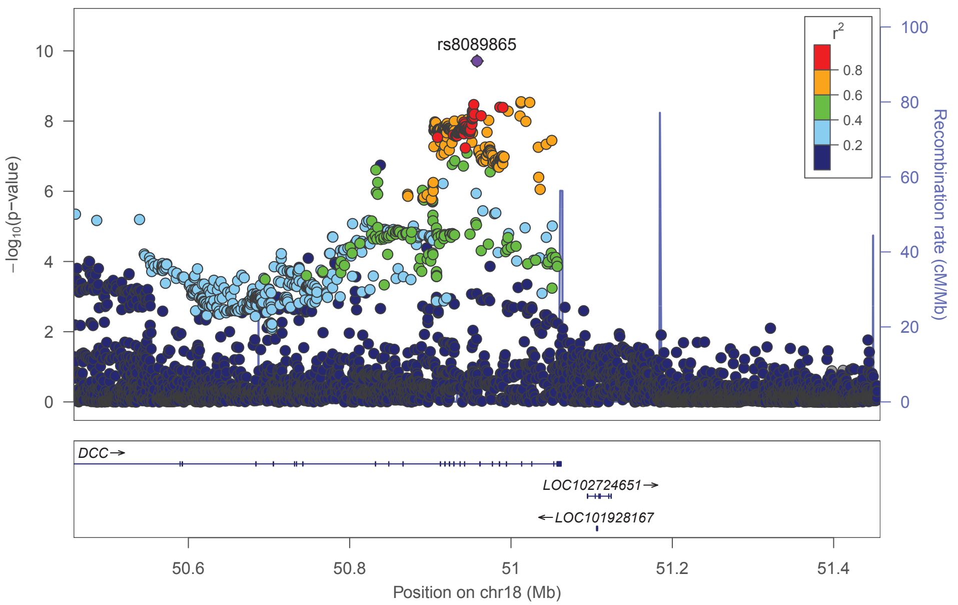

FVC_European_ancestry_rs2236519

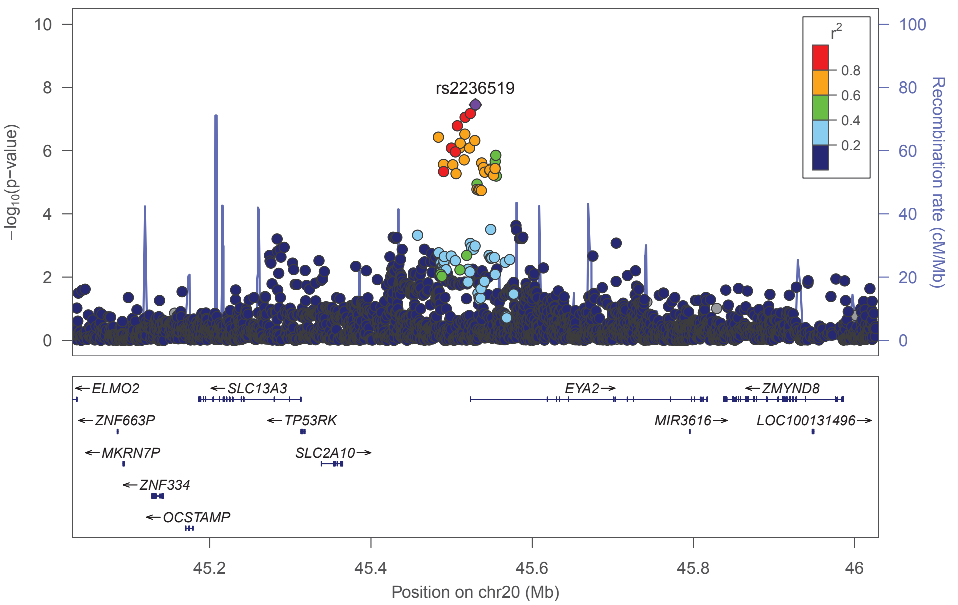

F.

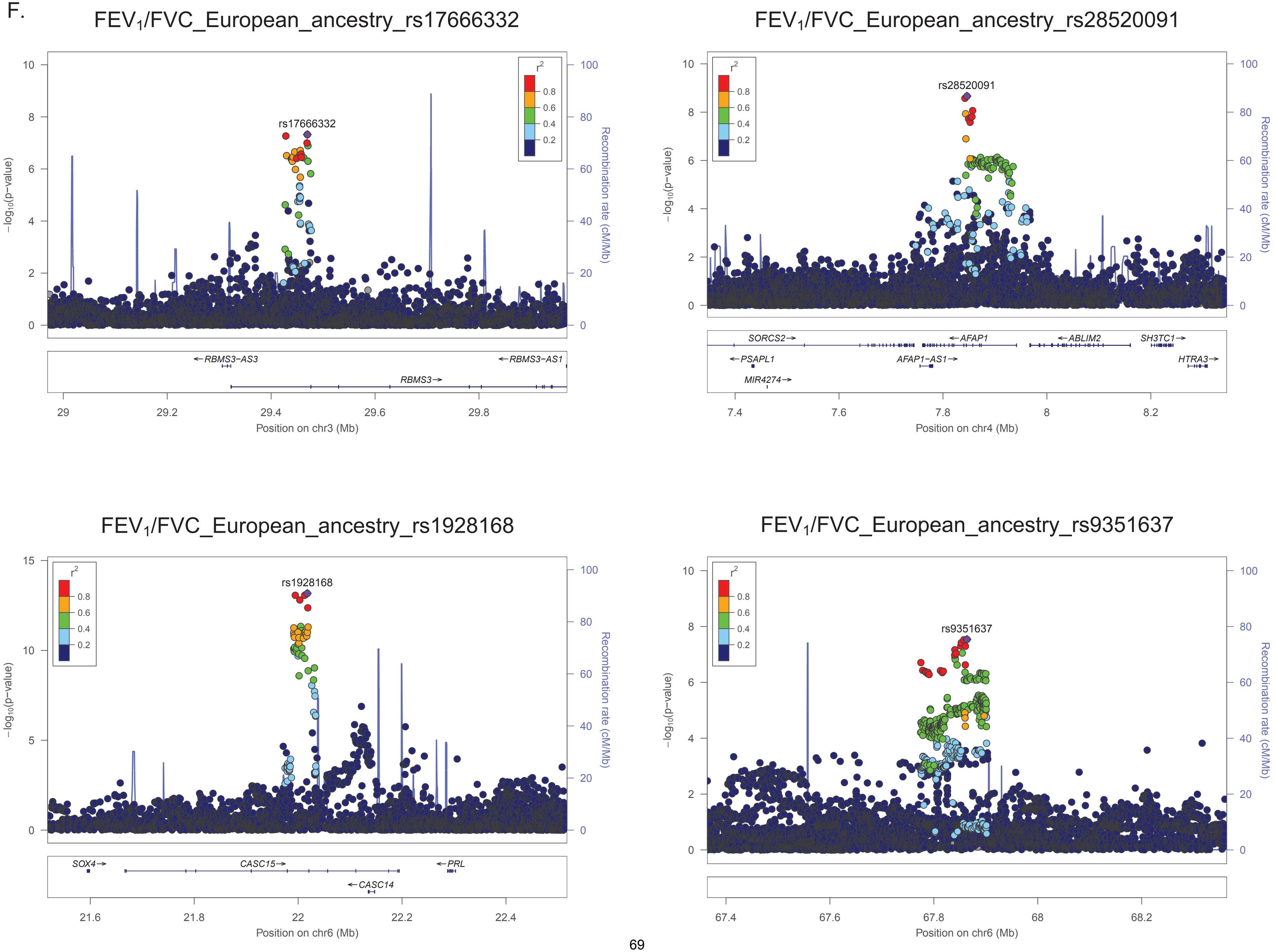
FEV1/FVC_European_ancestry_rs17666332
rs17666332
r2
0.8
0.6
0.4
0.2
−log10(p−value)
Recombination rate (cM/Mb)
RBMS3−AS3
RBMS3−AS1
RBMS3
Position on chr3 (Mb)
FEV1/FVC_European_ancestry_rs28520091
rs28520091
SORCS2
AFAP1
ABLIM2
SH3TC1
PSAPL1
AFAP1−AS1
HTRA3
MIR4274
Position on chr4 (Mb)
FEV1/FVC_European_ancestry_rs1928168
rs1928168
SOX4
CASC15
PRL
CASC14
Position on chr6 (Mb)
FEV1/FVC_European_ancestry_rs9351637
rs9351637
Position on chr6 (Mb)

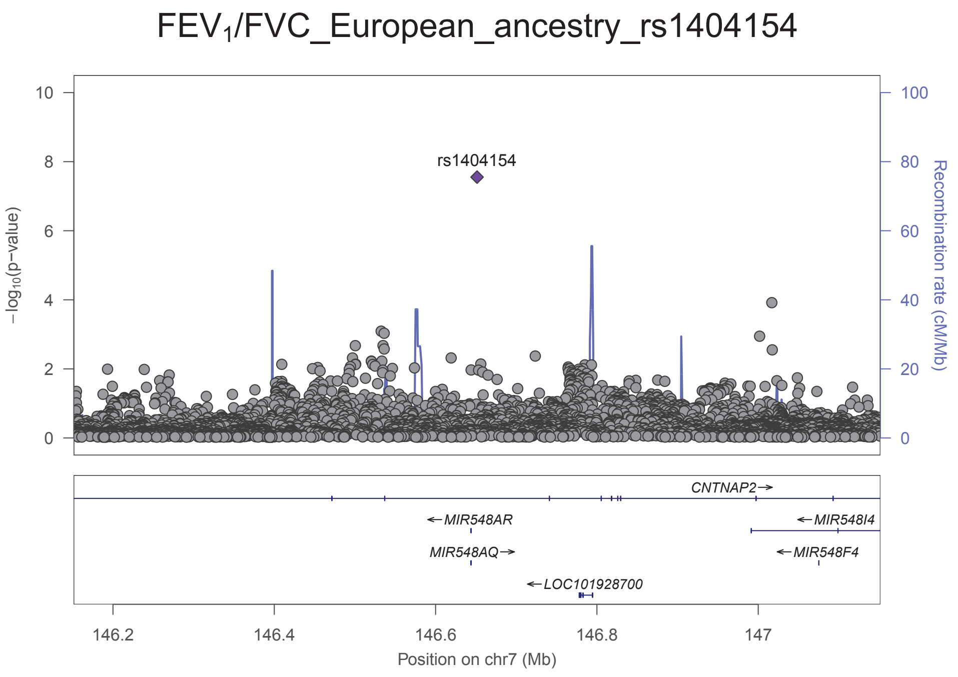
FEV1/FVC_European_ancestry_rs1404154
rs1404154
−log10(p−value)
Recombination rate (cM/Mb)
CNTNAP2
MIR548AR
MIR548I4
MIR548AQ
MIR548F4
LOC101928700
Position on chr7 (Mb)

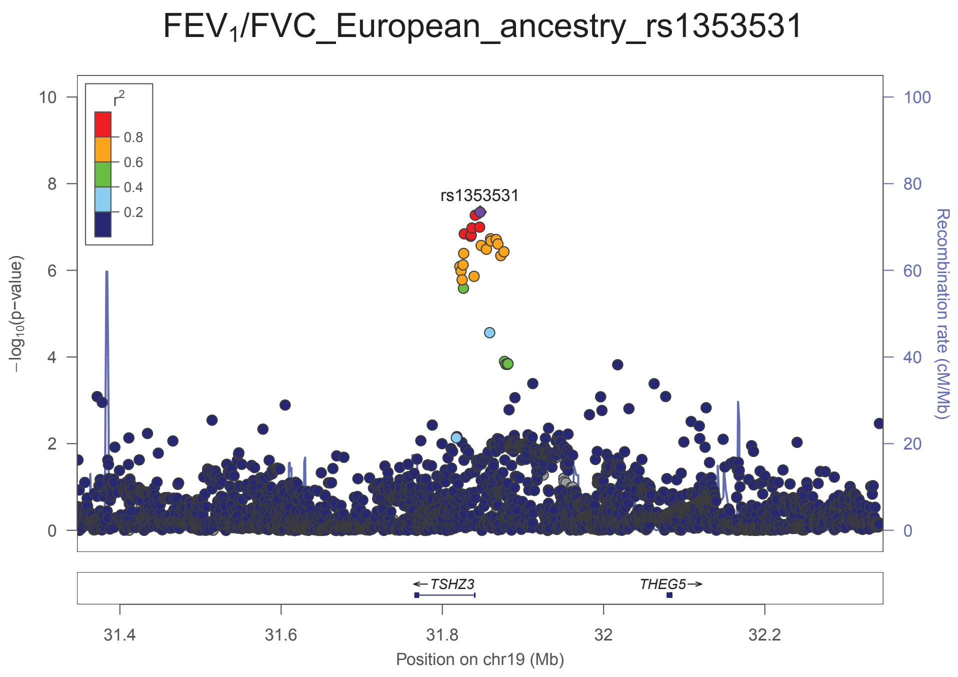
FEV1/FVC_European_ancestry_rs1353531
rs1353531
r2
−log10(p−value)
Recombination rate (cM/Mb)
TSHZ3
THEG5
Position on chr19 (Mb)

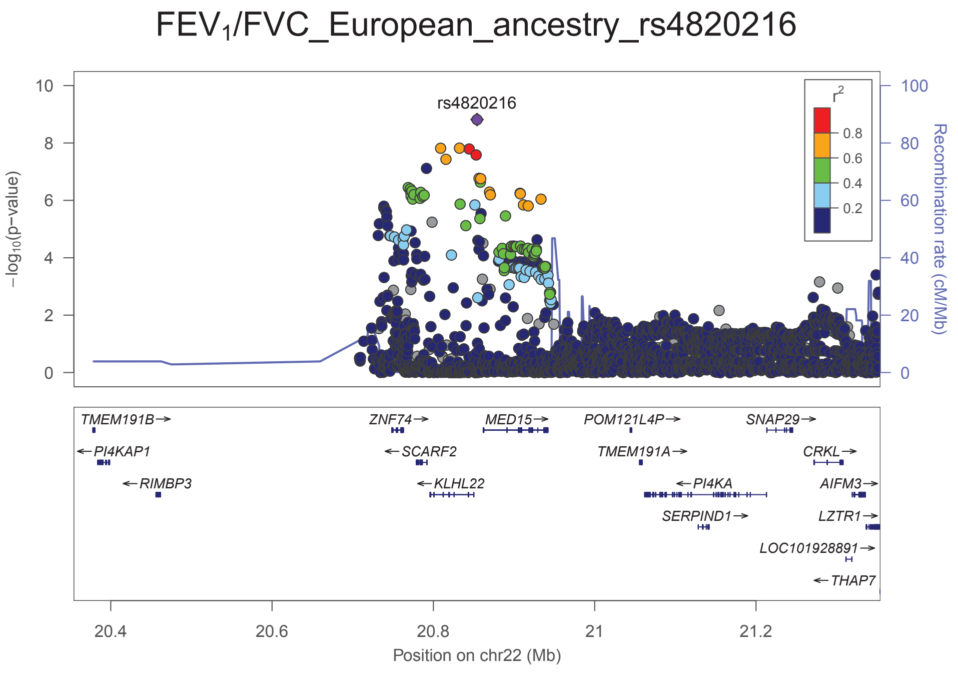
FEV1/FVC_European_ancestry_rs4820216
rs4820216
r2
−log10(p−value)
Recombination rate (cM/Mb)
TMEM191B
PI4KAP1
RIMBP3
ZNF74
SCARF2
KLHL22
MED15
POM121L4P
TMEM191A
PI4KA
SERPIND1
SNAP29
CRKL
AIFM3
LZTR1
LOC101928891
THAP7
Position on chr22 (Mb)

G.

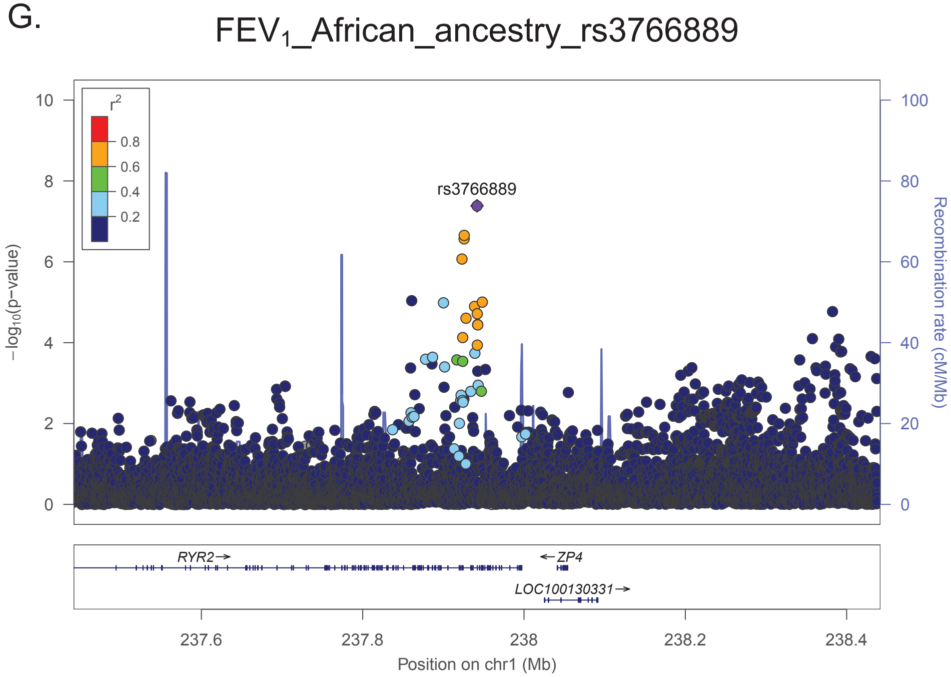
FEV1_African_ancestry_rs3766889
rs3766889
r2
0.8
0.6
0.4
0.2
−log10(p−value)
Recombination rate (cM/Mb)
RYR2→
←ZP4
LOC100130331→
237.6
237.8
238
238.2
238.4
Position on chr1 (Mb)

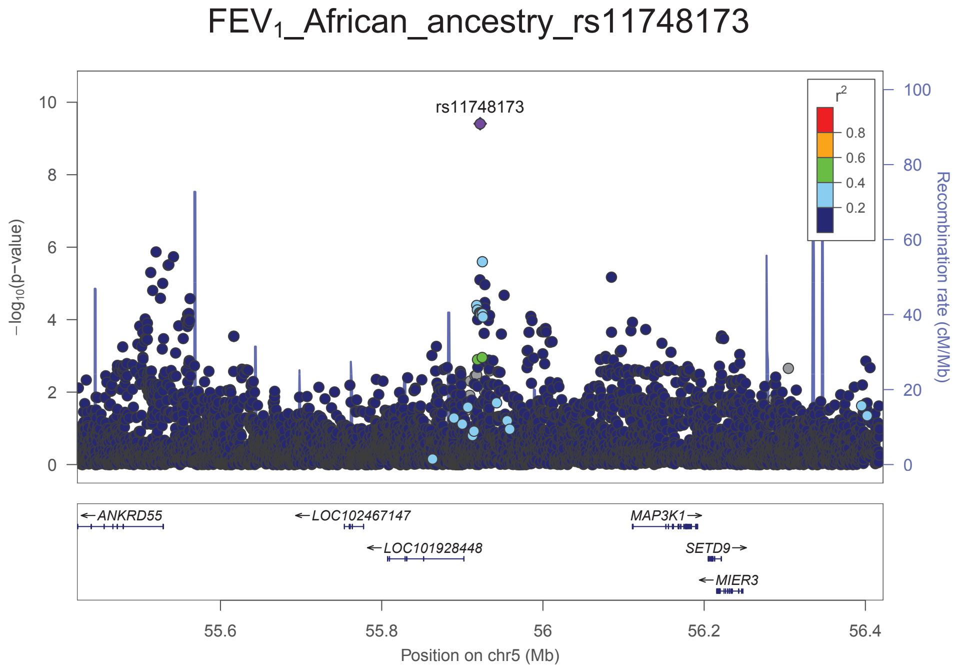
FEV1_African_ancestry_rs11748173
rs11748173
r2
0.8
0.6
0.4
0.2
−log10(p−value)
Recombination rate (cM/Mb)
←ANKRD55
←LOC102467147
←LOC101928448
MAP3K1→
SETD9→
←MIER3
55.6
55.8
56
56.2
56.4
Position on chr5 (Mb)

H.

FVC_African_ancestry_rs11492105

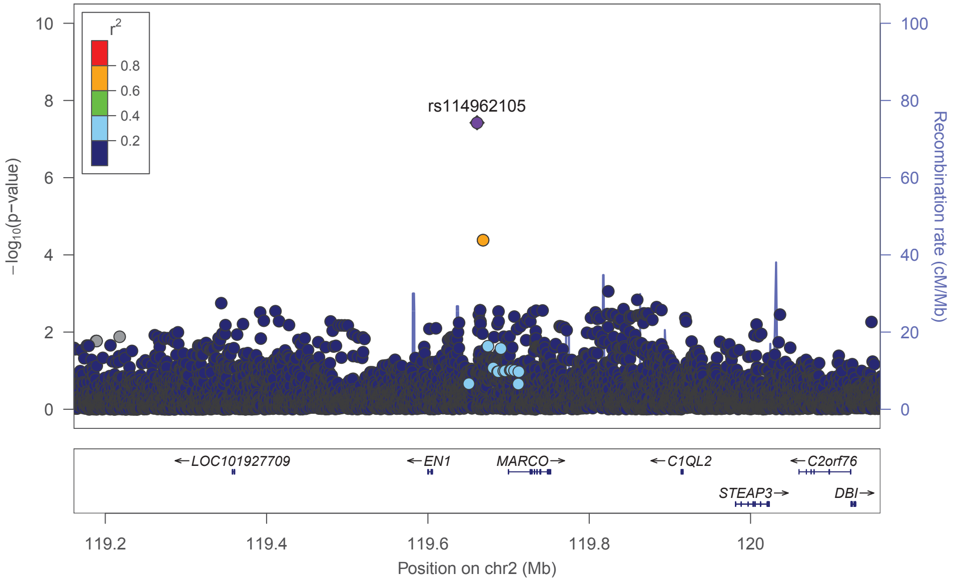

I.

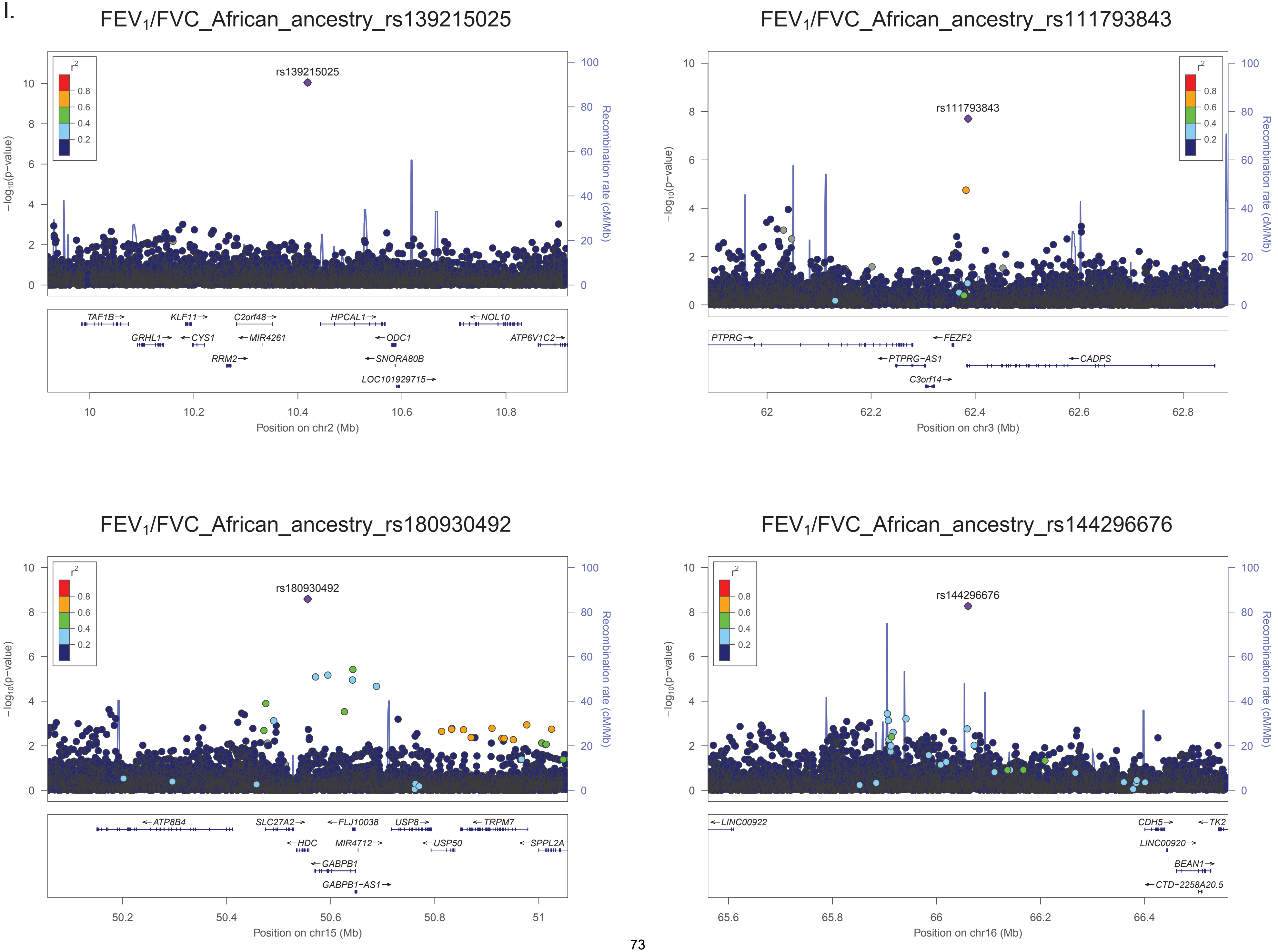
FEV1/FVC_African_ancestry_rs139215025
rs139215025
TAF1B→
KLF11→
C2orf48→
HPCAL1→
←NOL10
GRHL1→
←CYS1
←MIR4261
←ODC1
ATP6V1C2→
RRM2→
←SNORA80B
LOC101929715→
Position on chr2 (Mb)
FEV1/FVC_African_ancestry_rs111793843
rs111793843
PTPRG→
←FEZF2
←PTPRG-AS1
←CADPS
C3orf14→
Position on chr3 (Mb)
FEV1/FVC_African_ancestry_rs180930492
rs180930492
←ATP8B4
SLC27A2→
←FLJ10038
USP8→
←TRPM7
←HDC
MIR4712→
←USP50
←SPPL2A
←GABPB1
GABPB1-AS1→
Position on chr15 (Mb)
FEV1/FVC_African_ancestry_rs144296676
rs144296676
←LINC00922
CDH5→
←TK2
LINC00920→
BEAN1→
←CTD-2258A20.5
Position on chr16 (Mb)
−log10(p−value)
Recombination rate (cM/Mb)
r2

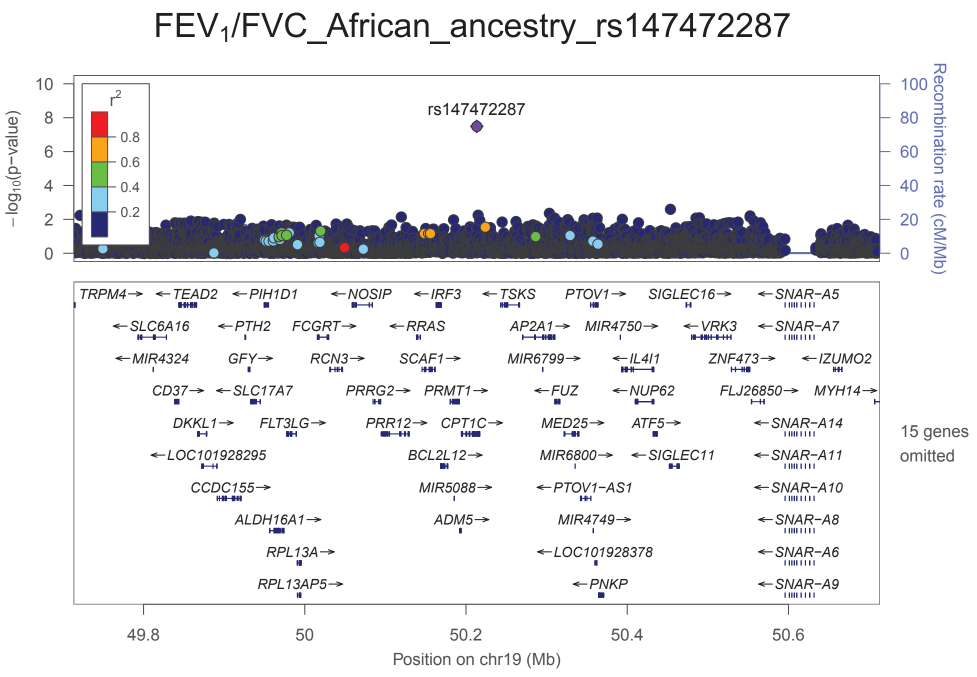
FEV1/FVC_African_ancestry_rs147472287
rs147472287
−log10(p−value)
Recombination rate (cM/Mb)
r2
0.8
0.6
0.4
0.2
TRPM4 TEAD2 PIH1D1 NOSIP IRF3 TSKS PTOV1 SIGLEC16 SNAR−A5
SLC6A16 PTH2 FCGRT RRAS AP2A1 MIR4750 VRK3 SNAR−A7
MIR4324 GFY RCN3 SCAF1 MIR6799 IL4I1 ZNF473 IZUMO2
CD37 SLC17A7 PRRG2 PRMT1 FUZ NUP62 FLJ26850 MYH14
DKKL1 FLT3LG PRR12 CPT1C MED25 ATF5 SNAR−A14
LOC101928295 BCL2L12 MIR6800 SIGLEC11 SNAR−A11
CCDC155 MIR5088 PTOV1−AS1 SNAR−A10
ALDH16A1 ADM5 MIR4749 SNAR−A8
RPL13A LOC101928378 SNAR−A6
RPL13AP5 PNKP SNAR−A9
15 genes omitted
49.8
50
50.2
50.4
50.6
Position on chr19 (Mb)

J.

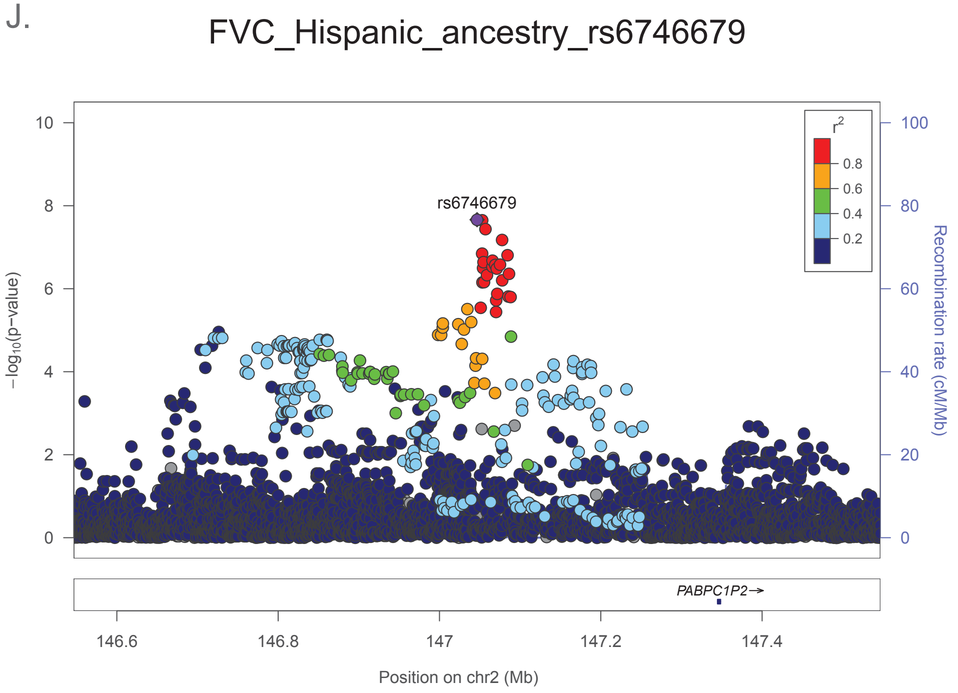

Supplementary Figure 2. Locus Zoom Plots for lead variants in/ near novel loci identified for pulmonary function in the following CHARGE meta-analyses: Panel A Multiethnic FEV_1; Panel B Multiethnic FVC; Panel C Multiethnic FEV_1/FVC; Panel D European ancestry FEV_1; Panel E European ancestry FVC; Panel F European ancestry FEV_1/FVC; Panel G African ancestry FEV_1; Panel H African ancestry FVC; Panel I African ancestry FEV_1/FVC; Panel J Hispanic/Latino ethnicity FVC. Insertion/deletion variants (INDELs) omitted. Linkage disequilibrium for European ancestry and Multiethnic plots were based on hg19/1000 Genomes Mar 2012 EUR, for African ancestry plots based on hg19/1000 Genomes Mar 2012 AFR, and for Hispanic/Latino ethnicity plots based on hg19/1000 Genomes Mar 2012 AMR (http:// locuszoom.sph.umich.edu/).

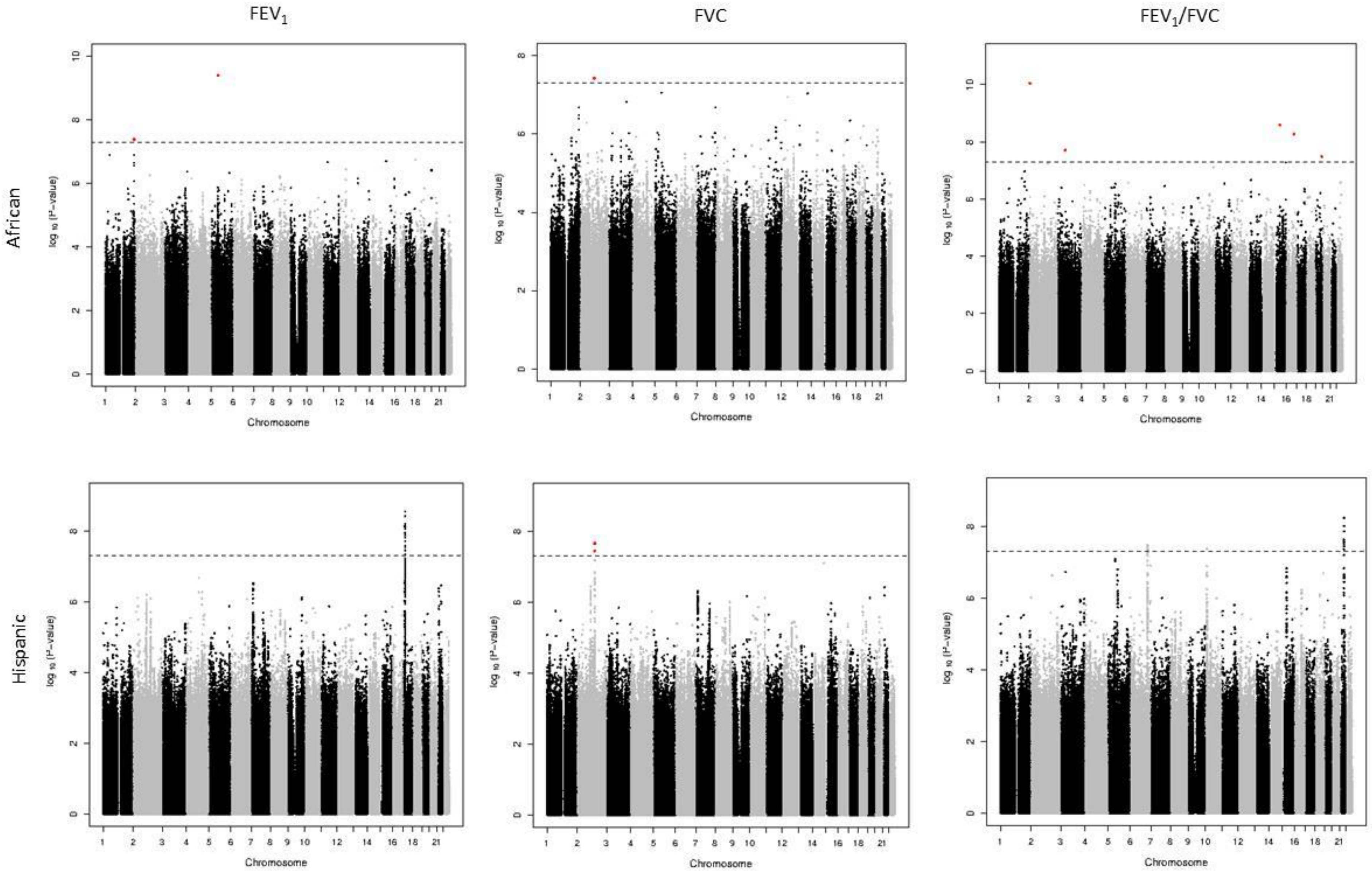
FEV1
FVC
FEV1/FVC
African
Hispanic
log 10 (P-value)
Chromosome

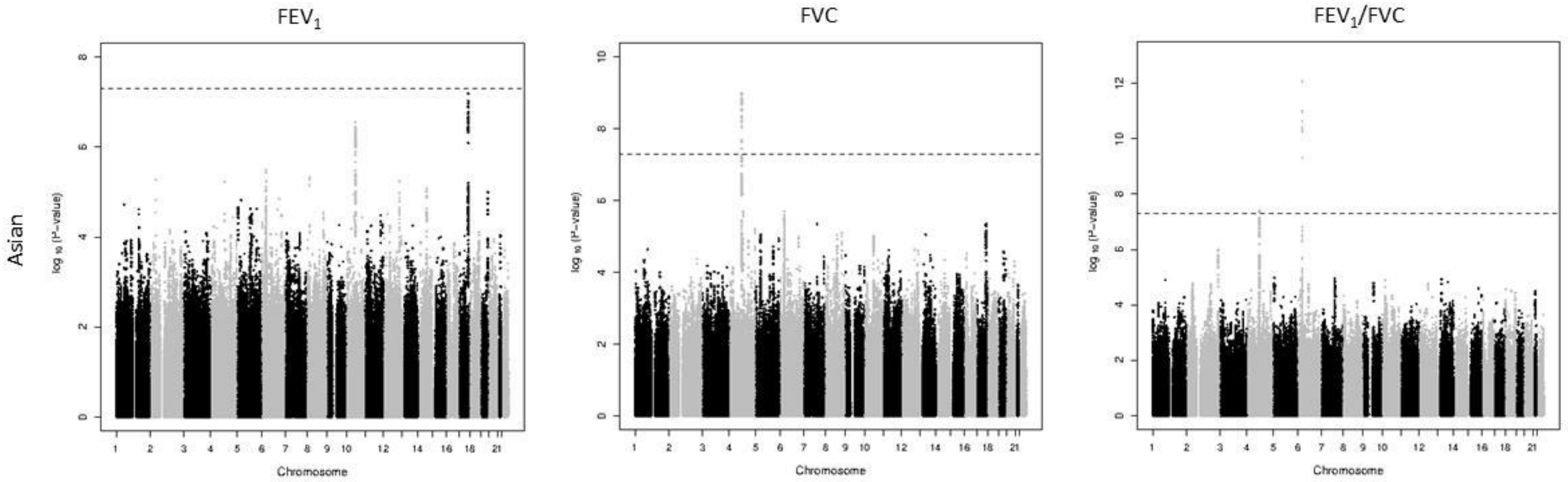

Supplementary Figure 3. Manhattan plots for genome-wide association results for pulmonary function (FEV_1, FVC, and FEV_1/FVC) from African ancestry, Hispanic/Latino ethnicity and Asian ancestry meta-analyses in CHARGE. Novel loci indicated by red. Significance level ($5x10^{-8}$) indicated by dashed line.

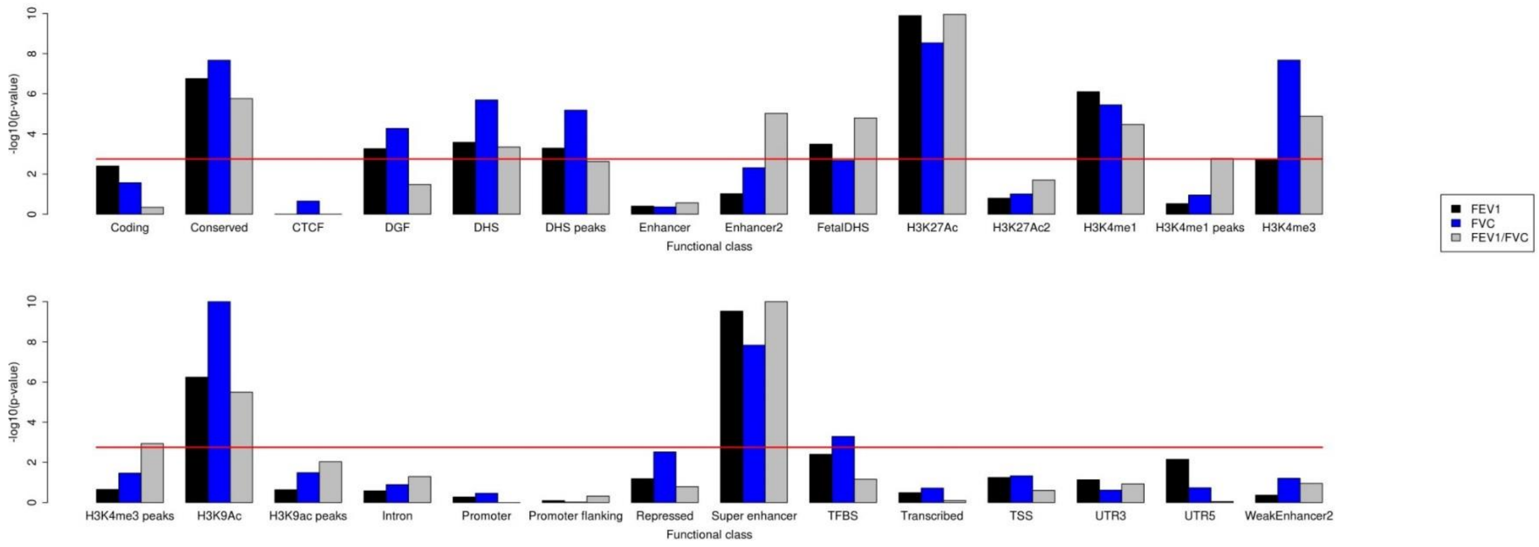

Supplementary Figure 4. Enrichment analysis of common variants associated with FEV_1, FVC and FEV_1/FVC by functional class using LD Score Regression

Legend: TFBS: transcription factor binding site; TSS: transcription start site; DGF: DNase genomic foot printing; DHS: DNase I hypersensitive sites; CTCF: CCCTC-binding factor.

(A) FEV_1

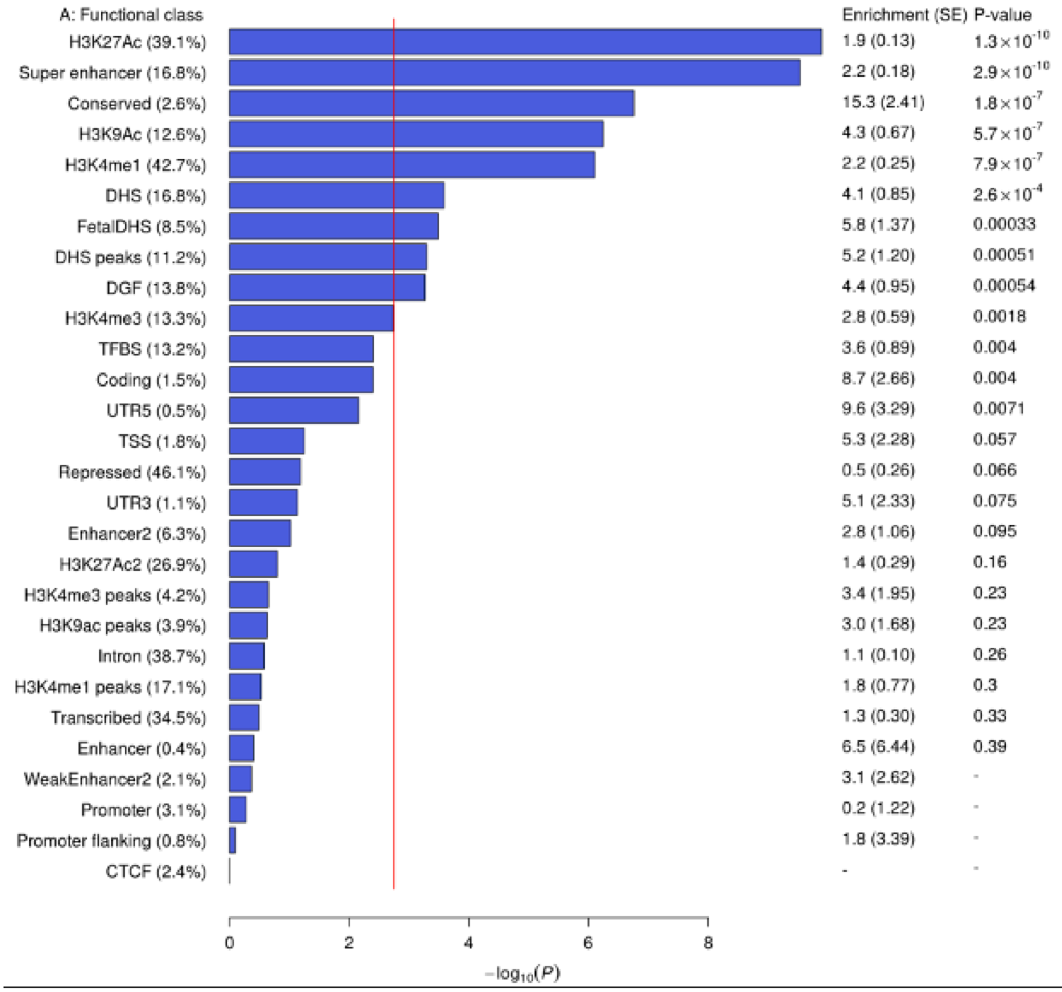

A: Functional class
H3K27Ac (39.1%)
Super enhancer (16.8%)
Conserved (2.6%)
H3K9Ac (12.6%)
H3K4me1 (42.7%)
DHS (16.8%)
FetalDHS (8.5%)
DHS peaks (11.2%)
DGF (13.8%)
H3K4me3 (13.3%)
TFBS (13.2%)
Coding (1.5%)
UTR5 (0.5%)
TSS (1.8%)
Repressed (46.1%)
UTR3 (1.1%)
Enhancer2 (6.3%)
H3K27Ac2 (26.9%)
H3K4me3 peaks (4.2%)
H3K9ac peaks (3.9%)
Intron (38.7%)
H3K4me1 peaks (17.1%)
Transcribed (34.5%)
Enhancer (0.4%)
WeakEnhancer2 (2.1%)
Promoter (3.1%)
Promoter flanking (0.8%)
CTCF (2.4%)
0
2
4
6
8
−log10(P)
Enrichment (SE)
P-value
1.9 (0.13) 1.3×10-10
2.2 (0.18) 2.9×10-10
15.3 (2.41) 1.8×10-7
4.3 (0.67) 5.7×10-7
2.2 (0.25) 7.9×10-7
4.1 (0.85) 2.6×10-4
5.8 (1.37) 0.00033
5.2 (1.20) 0.00051
4.4 (0.95) 0.00054
2.8 (0.59) 0.0018
3.6 (0.89) 0.004
8.7 (2.66) 0.004
9.6 (3.29) 0.0071
5.3 (2.28) 0.057
0.5 (0.26) 0.066
5.1 (2.33) 0.075
2.8 (1.06) 0.095
1.4 (0.29) 0.16
3.4 (1.95) 0.23
3.0 (1.68) 0.23
1.1 (0.10) 0.26
1.8 (0.77) 0.3
1.3 (0.30) 0.33
6.5 (6.44) 0.39
3.1 (2.62) -
0.2 (1.22) -
1.8 (3.39) -
- -

(B) FVC

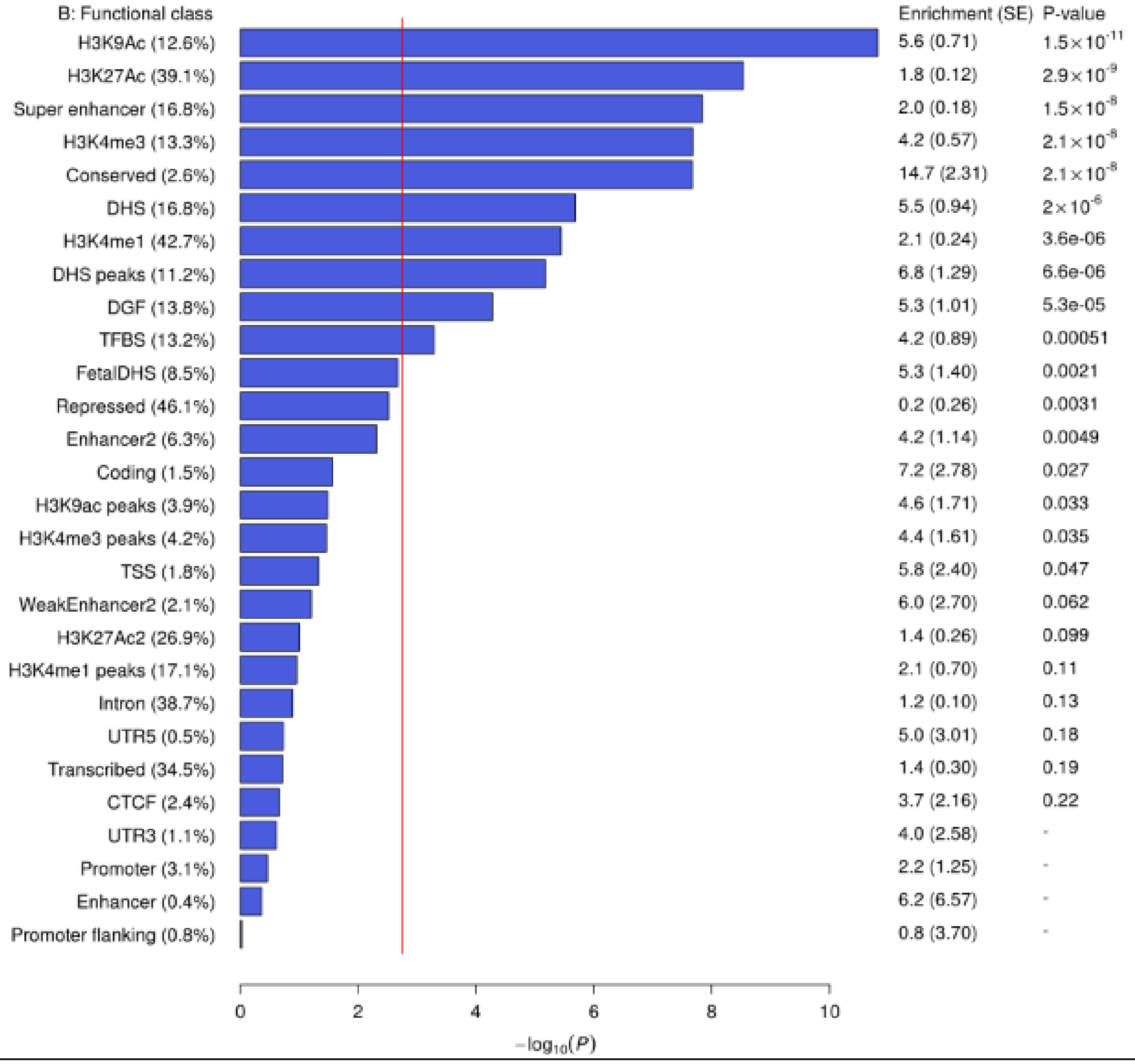
B: Functional class
Enrichment (SE)
P-value
H3K9Ac (12.6%) 5.6 (0.71) 1.5×10-11
H3K27Ac (39.1%) 1.8 (0.12) 2.9×10-9
Super enhancer (16.8%) 2.0 (0.18) 1.5×10-8
H3K4me3 (13.3%) 4.2 (0.57) 2.1×10-8
Conserved (2.6%) 14.7 (2.31) 2.1×10-8
DHS (16.8%) 5.5 (0.94) 2×10-6
H3K4me1 (42.7%) 2.1 (0.24) 3.6e-06
DHS peaks (11.2%) 6.8 (1.29) 6.6e-06
DGF (13.8%) 5.3 (1.01) 5.3e-05
TFBS (13.2%) 4.2 (0.89) 0.00051
FetalDHS (8.5%) 5.3 (1.40) 0.0021
Repressed (46.1%) 0.2 (0.26) 0.0031
Enhancer2 (6.3%) 4.2 (1.14) 0.0049
Coding (1.5%) 7.2 (2.78) 0.027
H3K9ac peaks (3.9%) 4.6 (1.71) 0.033
H3K4me3 peaks (4.2%) 4.4 (1.61) 0.035
TSS (1.8%) 5.8 (2.40) 0.047
WeakEnhancer2 (2.1%) 6.0 (2.70) 0.062
H3K27Ac2 (26.9%) 1.4 (0.26) 0.099
H3K4me1 peaks (17.1%) 2.1 (0.70) 0.11
Intron (38.7%) 1.2 (0.10) 0.13
UTR5 (0.5%) 5.0 (3.01) 0.18
Transcribed (34.5%) 1.4 (0.30) 0.19
CTCF (2.4%) 3.7 (2.16) 0.22
UTR3 (1.1%) 4.0 (2.58) -
Promoter (3.1%) 2.2 (1.25) -
Enhancer (0.4%) 6.2 (6.57) -
Promoter flanking (0.8%) 0.8 (3.70) -
0 2 4 6 8 10
−log10(P)

(C) FEV_1/FVC

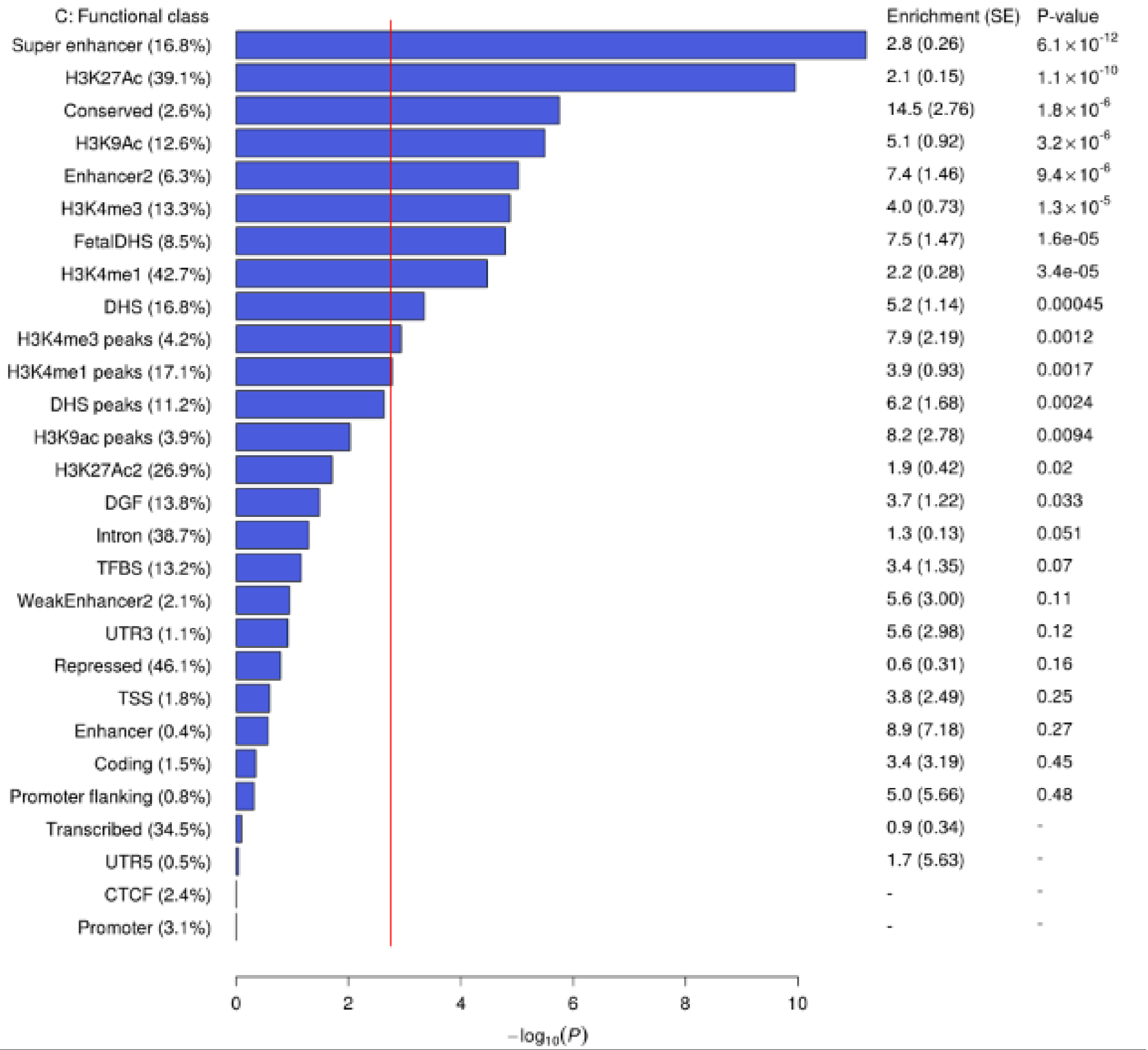

Supplementary Figure 5. Enrichment analysis of common variants associated with pulmonary function by functional class using LD Score Regression

(A) FEV_1

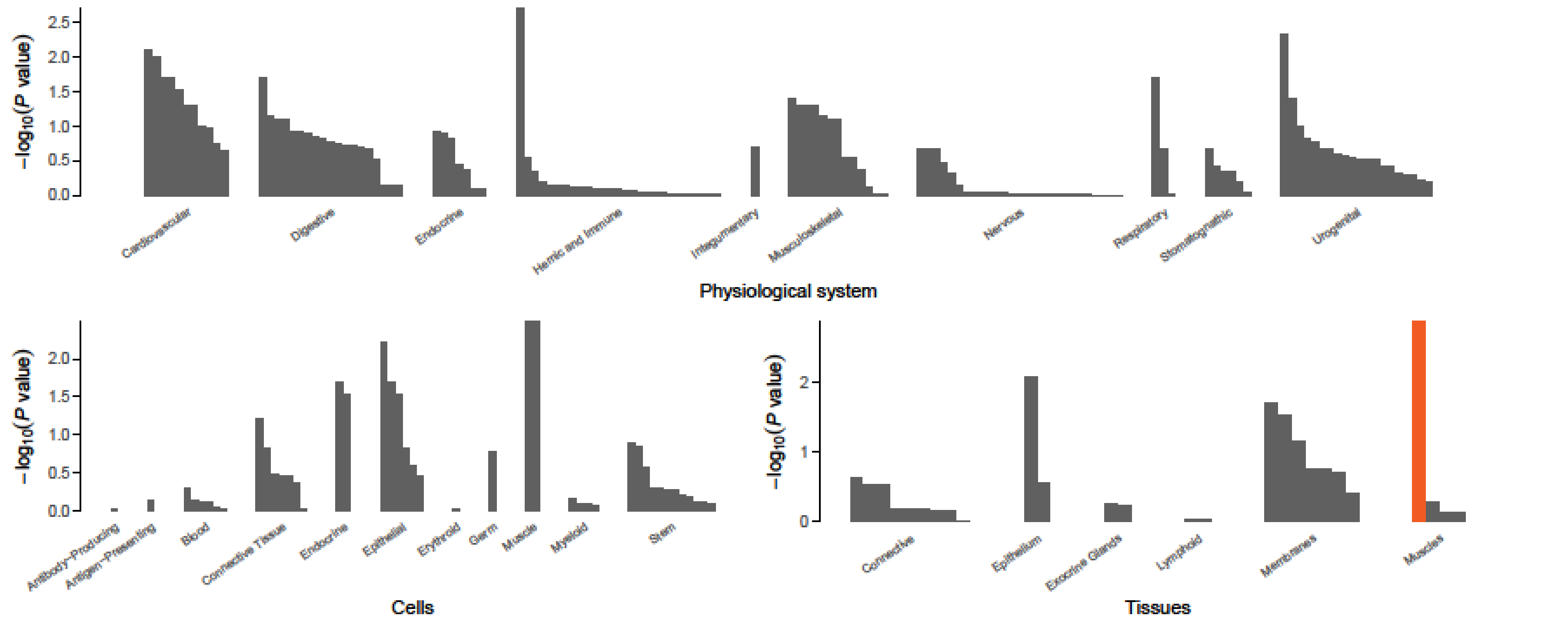

−log10(P value)
Cardiovascular
Digestive
Endocrine
Hemic and Immune
Integumentary
Musculoskeletal
Nervous
Respiratory
Stomatognathic
Urogenital
Physiological system
Antibody−Producing
Antigen−Presenting
Blood
Connective Tissue
Endocrine
Epithelial
Erythroid
Germ
Muscle
Myeloid
Stem
Cells
Connective
Epithelium
Exocrine Glands
Lymphoid
Membranes
Muscles
Tissues

(B) FVC

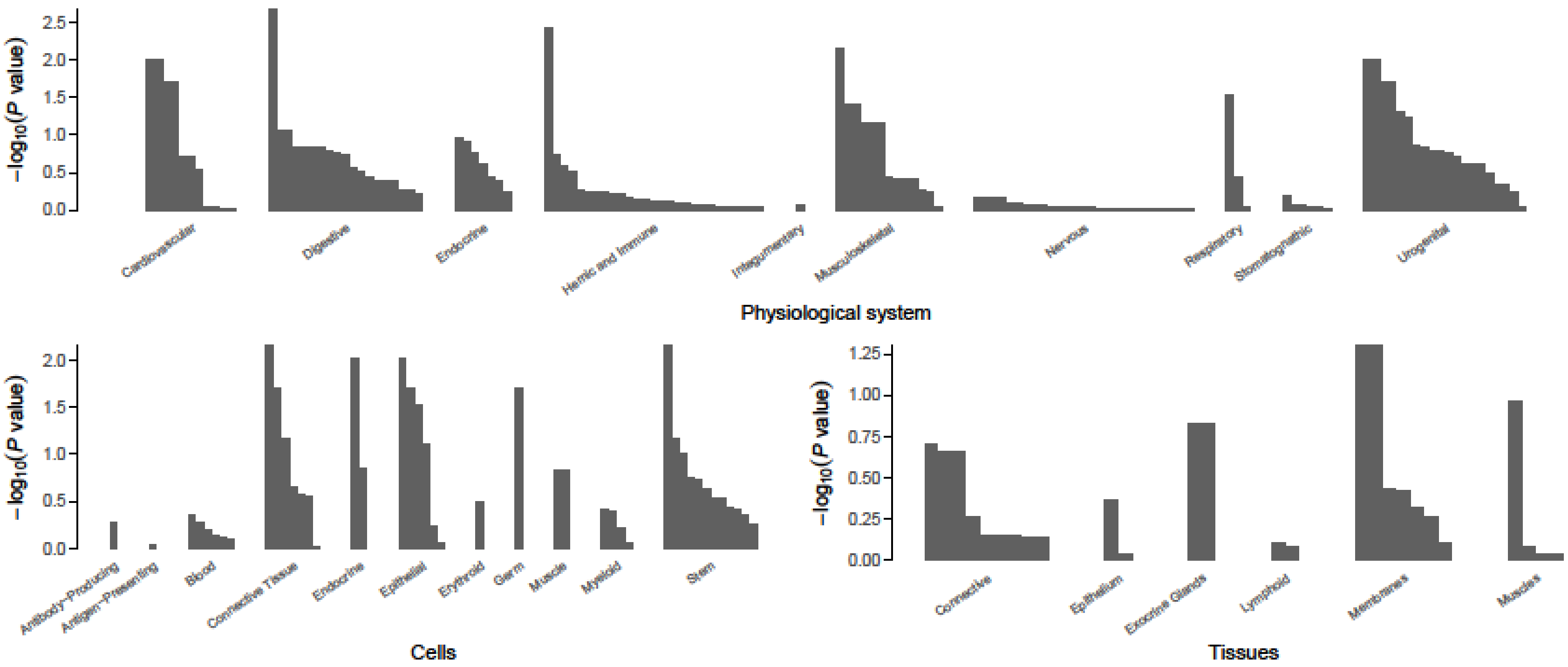
−log10(P value)
2.5
2.0
1.5
1.0
0.5
0.0
Cardiovascular
Digestive
Endocrine
Hemic and Immune
Integumentary
Musculoskeletal
Nervous
Respiratory
Stomatognathic
Urogenital
Physiological system
−log10(P value)
2.0
1.5
1.0
0.5
0.0
Antibody−Producing
Antigen−Presenting
Blood
Connective Tissue
Endocrine
Epithelial
Erythroid
Germ
Muscle
Myeloid
Stem
Cells
−log10(P value)
1.25
1.00
0.75
0.50
0.25
0.00
Connective
Epithelium
Exocrine Glands
Lymphoid
Membranes
Muscles
Tissues

(C) FEV_1/FVC

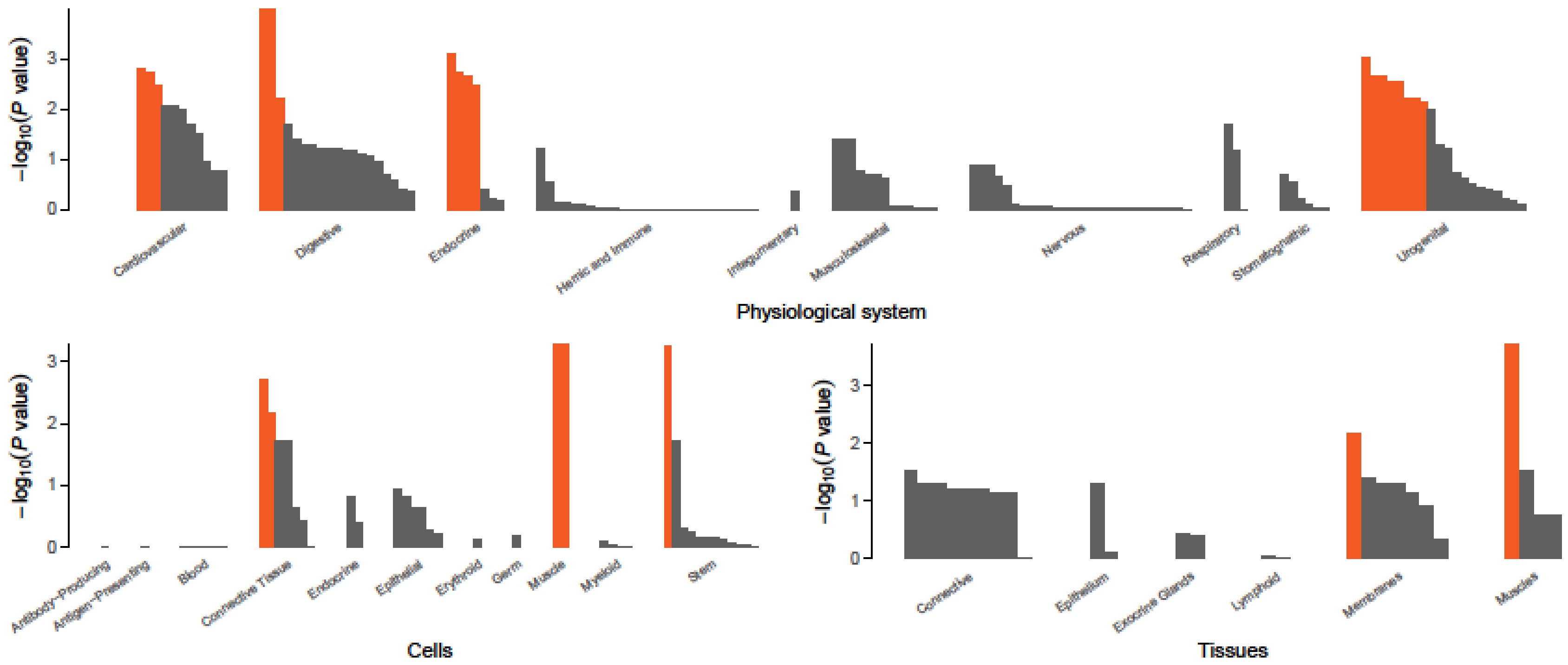

Supplementary Figure 6. Tissue Plots Showing Significantly Enriched (FDR<0.05) Types Based on European Ancestry Results using DEPICT

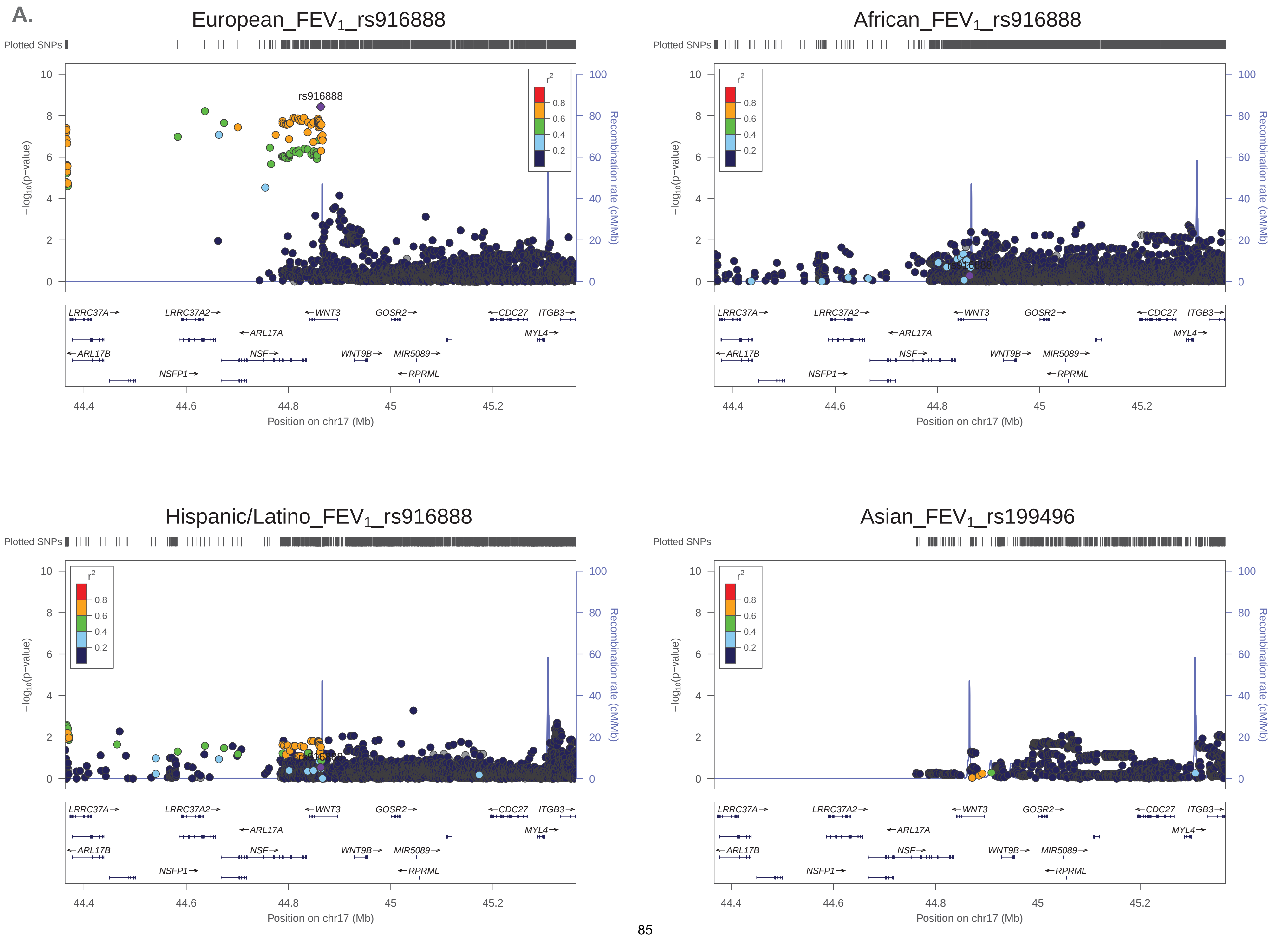
A.
European_FEV1_rs916888
African_FEV1_rs916888
Hispanic/Latino_FEV1_rs916888
Asian_FEV1_rs199496
Plotted SNPs
−log10(p−value)
Recombination rate (cM/Mb)
r2
0.8
0.6
0.4
0.2
rs916888
0
2
4
6
8
10
0
20
40
60
80
100
LRRC37A→
LRRC37A2→
←WNT3
GOSR2→
←CDC27
ITGB3→
←ARL17A
MYL4→
←ARL17B
NSF→
WNT9B→
MIR5089→
NSFP1→
←RPRML
44.4
44.6
44.8
45
45.2
Position on chr17 (Mb)

B.

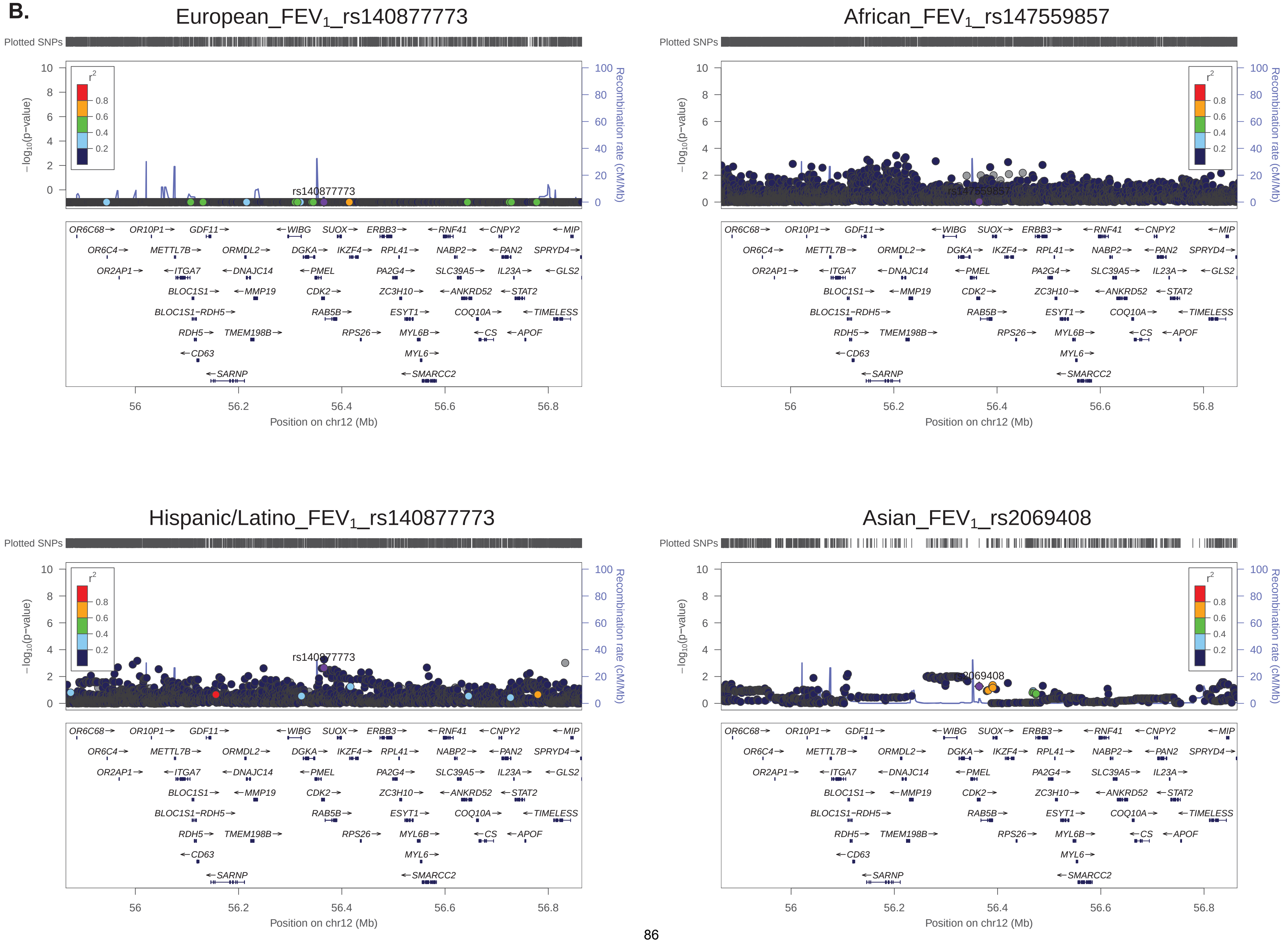
European_FEV1_rs140877773
African_FEV1_rs147559857
Hispanic/Latino_FEV1_rs140877773
Asian_FEV1_rs2069408
Plotted SNPs
−log10(p−value)
Recombination rate (cM/Mb)
r2
rs140877773
rs147559857
rs2069408
Position on chr12 (Mb)
OR6C68 OR10P1 GDF11 WIBG SUOX ERBB3 RNF41 CNPY2 MIP
OR6C4 METTL7B ORMDL2 DGKA IKZF4 RPL41 NABP2 PAN2 SPRYD4
OR2AP1 ITGA7 DNAJC14 PMEL PA2G4 SLC39A5 IL23A GLS2
BLOC1S1 MMP19 CDK2 ZC3H10 ANKRD52 STAT2
BLOC1S1−RDH5 RAB5B ESYT1 COQ10A TIMELESS
RDH5 TMEM198B RPS26 MYL6B CS APOF
CD63 MYL6
SARNP SMARCC2

C.

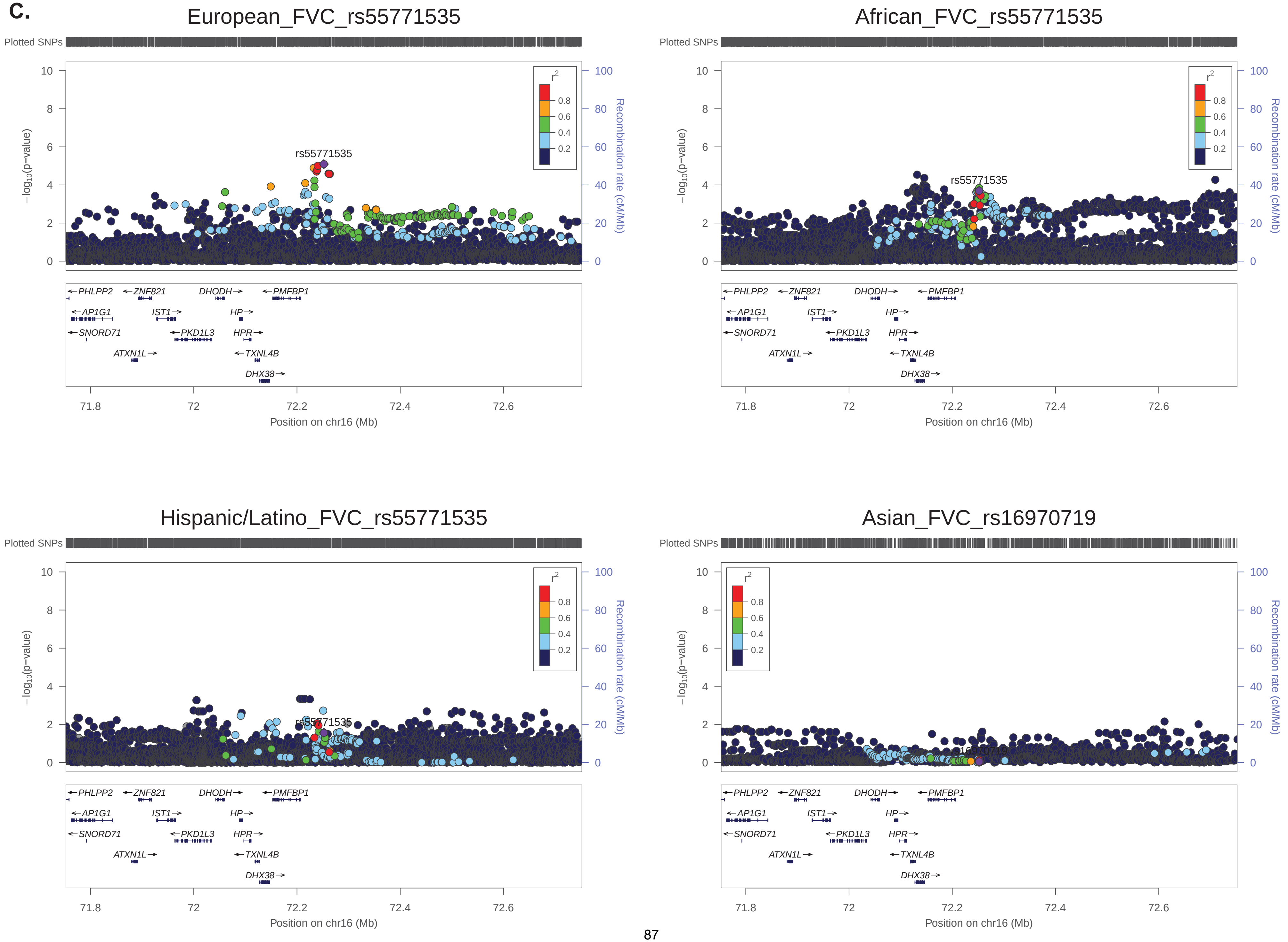
European_FVC_rs55771535
African_FVC_rs55771535
Hispanic/Latino_FVC_rs55771535
Asian_FVC_rs16970719
Plotted SNPs
−log10(p−value)
Recombination rate (cM/Mb)
r2
0.8
0.6
0.4
0.2
rs55771535
PHLPP2
ZNF821
DHODH
PMFBP1
AP1G1
IST1
HP
SNORD71
PKD1L3
HPR
ATXN1L
TXNL4B
DHX38
71.8
72
72.2
72.4
72.6
Position on chr16 (Mb)

D.

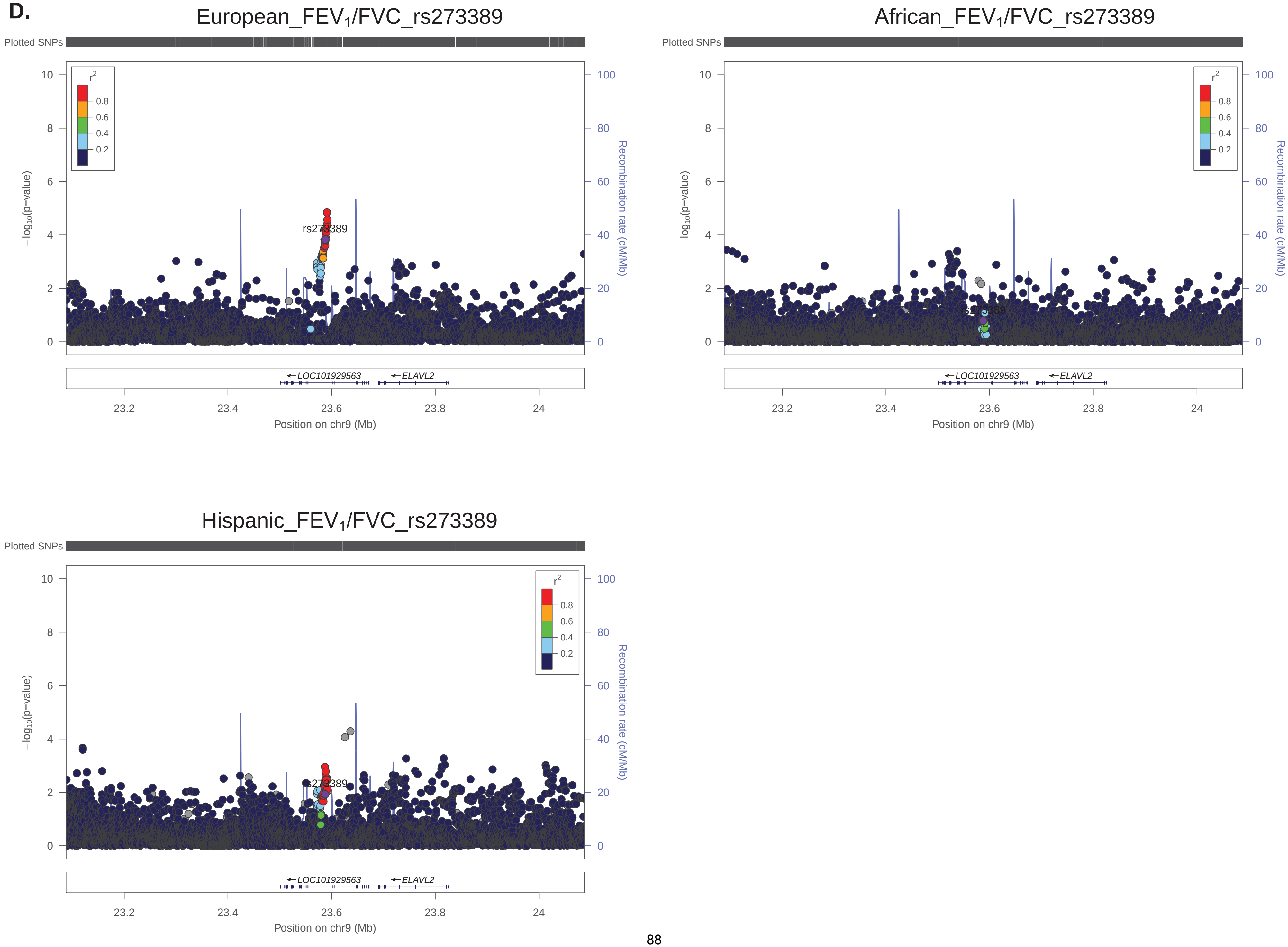
European_FEV1/FVC_rs273389
African_FEV1/FVC_rs273389
Hispanic_FEV1/FVC_rs273389
Plotted SNPs
−log10(p−value)
Recombination rate (cM/Mb)
r2
0.8
0.6
0.4
0.2
rs273389
LOC101929563
ELAVL2
23.2
23.4
23.6
23.8
24
Position on chr9 (Mb)

E.

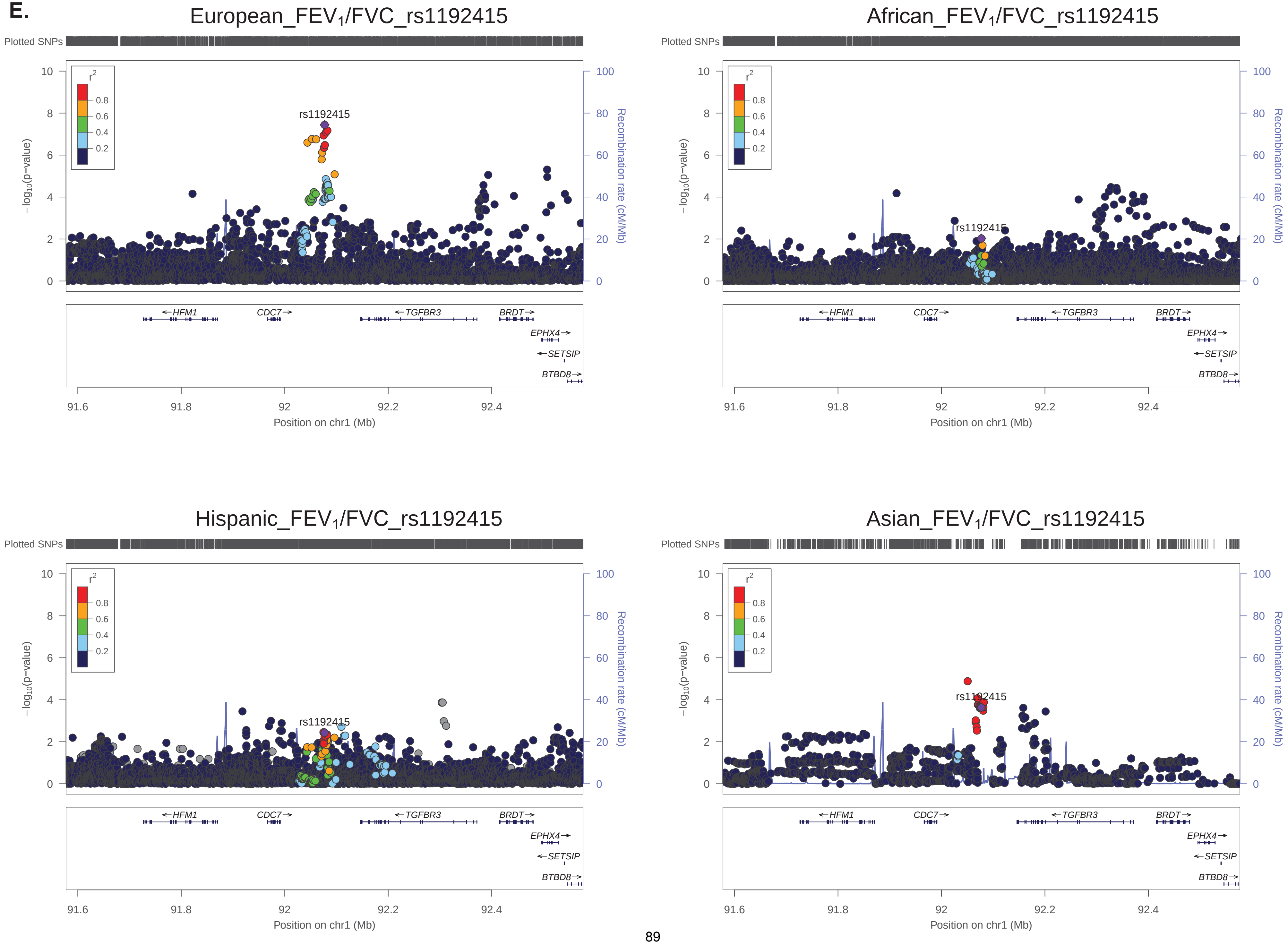
European_FEV$_1$/FVC_rs1192415
African_FEV$_1$/FVC_rs1192415
Hispanic_FEV$_1$/FVC_rs1192415
Asian_FEV$_1$/FVC_rs1192415
Plotted SNPs
r^2
0.8
0.6
0.4
0.2
rs1192415
$-\log_{10}$(p−value)
Recombination rate (cM/Mb)
HFM1
CDC7
TGFBR3
BRDT
EPHX4
SETSIP
BTBD8
91.6
91.8
92
92.2
92.4
Position on chr1 (Mb)

F.

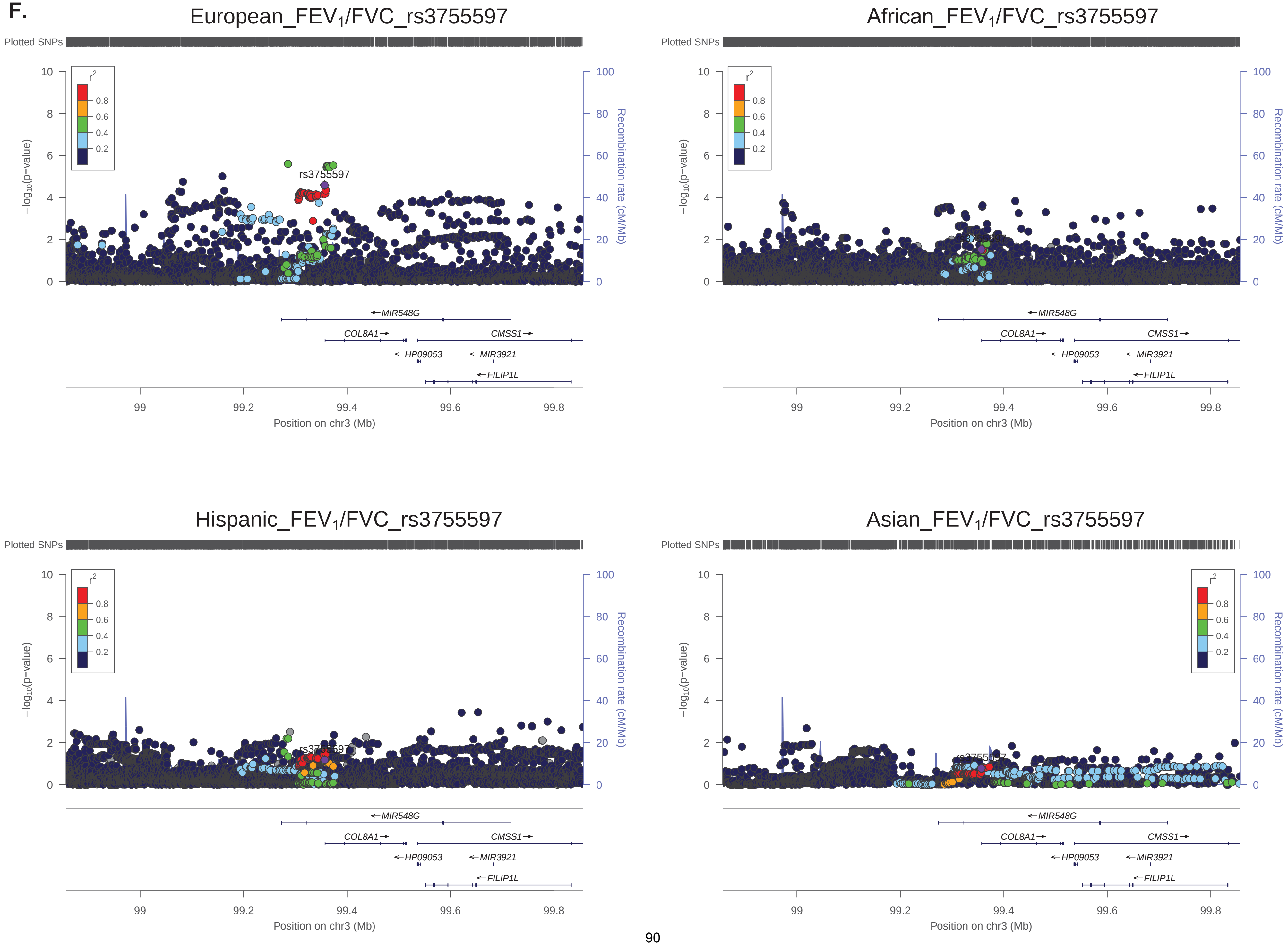
European_FEV1/FVC_rs3755597
African_FEV1/FVC_rs3755597
Hispanic_FEV1/FVC_rs3755597
Asian_FEV1/FVC_rs3755597
Plotted SNPs
−log10(p−value)
Recombination rate (cM/Mb)
r2
0.8
0.6
0.4
0.2
rs3755597
MIR548G
COL8A1
CMSS1
HP09053
MIR3921
FILIP1L
Position on chr3 (Mb)
99
99.2
99.4
99.6
99.8

Supplementary Figure 7. Locus zoom plots by ancestry for non-monomorphic variants (or nearest variant by distance) identified by PAINTOR as having high posterior probabilities of causality (>0.8). Panel A. FEV_1 rs916888; Panel B. FEV_1 rs140877773; Panel C. FVC rs55771535; Panel D. FEV_1/FVC rs273389; Panel E. FEV_1/FVC rs1192415; Panel F. FEV_1/FVC rs3755597. Linkage disequilibrium for European ancestry plots were based on hg19/1000 Genomes Mar 2012 EUR, for African ancestry plots based on hg19/1000 Genomes Mar 2012 AFR, for Hispanic/Latino ethnicity plots based on hg19/1000 Genomes Mar 2012 AMR, and for Asian ethnicity plots based on hg19/1000 Genomes Mar 2012 ASN (http://locuszoom.sph.umich.edu/). rs8043852 not shown because LD information not available for 1000 Genomes EUR reference population. Insertion/ deletion variants (INDELs) omitted.

Supplementary Tables

Supplementary Table 1. Lead Variants for Novel Loci Discovered in the Ancestry-Specific Meta-Analysis and Look-Up in Other Ancestry-Specific Meta-Analyses in the CHARGE Consortium

				European Ancestry						African Ancestry						Asian Ancestry						Hispanic Ancestry						Multiethnic					
Nearest Gene[a]	rsID	Chr:Pos	Phenotype[b]	N	Coded Allele	Coded Allele Freq	Beta[c]	SE	P-value	N	Coded Allele	Coded Allele Freq	Beta[c]	SE	P-value	N	Coded Allele	Coded Allele Freq	Beta[c]	SE	P-value	N	Coded Allele	Coded Allele Freq	Beta[c]	SE	P-value	N	Coded Allele	Coded Allele Freq	Beta[c]	SE	P-value
ALX1,RASSF9	rs10779158	12:85724096	FEV_1	60550	a	0.34	15.89	2.9	4.36E-08	8428	a	0.75	-4.09	9.01	0.65	9934	a	0.51	11.67	5.40	0.03	11775	a	0.45	-3.44	5.73	0.55	90687	a	0.41	10.89	2.26	1.5E-06
LOC644172,CRHR1	rs143246821	17:43685698	FEV_1	39416	a	0.79	30.58	4.99	9.06E-10	4588	a	0.86	3.08	17.16	0.86	NA	NA	NA[e]	NA	NA	NA	NA	NA	NA	NA	NA	NA	44004	a	0.80	28.43	4.79	3.0E-09
WNT3	rs916888	17:44863133	FEV_1	60551	t	0.75	20.53	3.48	3.76E-09	8429	t	0.83	7.77	11.95	0.52	NA	NA	NA[e]	NA	NA	NA	11775	t	0.80	7.93	7.43	0.29	80755	t	0.77	17.58	3.05	8.2E-09
AP3B1	rs252746	5:77392117	FEV_1	60551	a	0.78	20.05	3.45	6.19E-09	8428	a	0.62	-5.62	7.33	0.44	9955	a	0.93	2.41	10.91	0.83	11775	a	0.70	3.13	6.17	0.61	90709	a	0.75	12.25	2.70	5.6E-06
TSHZ3	rs1353531	19:31846907	Ratio	60530	t	0.14	-0.003	0.0006	4.53E-08	8424	t	0.18	0.0009	0.002	0.53	NA	NA	NA	NA	NA	NA	11764	t	0.20	-0.002	0.001	0.02	80718	t	0.16	-0.003	0.0005	9.9E-08
KLHL22,MED15	rs4820216	22:20854161	Ratio	60528	t	0.15	-0.004	0.0006	1.53E-09	8423	t	0.07	-0.0006	0.003	0.80	NA[f]	t	0.12	0.002	0.003	0.47	11764	t	0.10	-0.004	0.001	0.006	82714	t	0.13	-0.003	0.0005	2.6E-10
RBMS3	rs17666332	3:29469675	Ratio	60531	t	0.72	0.003	0.0005	4.76E-08	8423	t	0.88	-0.0025	0.002	0.21	9947	t	0.89	0.002	0.001	0.19	11764	t	0.84	0.0019	0.001	0.09	90665	t	0.75	0.002	0.0004	4.9E-08
AFAP1	rs28520091	4:7846240	Ratio	60527	t	0.48	0.003	0.0004	2.17E-09	8424	t	0.25	0.0023	0.002	0.13	NA	NA	NA	NA	NA	NA	11764	t	0.37	0.0005	0.0008	0.57	80715	t	0.44	0.002	0.0004	8.4E-09
LINC00340	rs1928168	6:22017738	Ratio	60522	t	0.51	0.003	0.0004	6.74E-14	8423	t	0.79	0.0018	0.002	0.23	9959	t	0.80	-0.0001	0.001	0.92	11764	t	0.70	0.0017	0.0009	0.05	90668	t	0.58	0.003	0.0003	3.4E-13
SLC25A51P1,BAI3	rs9351637	6:67863782	Ratio	60528	t	0.61	0.002	0.0004	2.89E-08	8424	t	0.59	-0.0008	0.001	0.53	NA	NA	NA	NA	NA	NA	11764	t	0.55	0.0005	0.0008	0.56	80716	t	0.59	0.002	0.0004	2.5E-06
CNTNAP2	rs1404154	7:146651409	Ratio	23748	t	0.99	-0.03	0.006	2.80E-08	NA	NA	NA[e]	NA	NA	NA	NA	NA	NA[e]	NA	NA	NA	11764	t	0.93	-0.001	0.002	0.61	36834	t	0.94	-0.005	0.002	1.6E-02
LOC728989	rs12724426	1:146494027	FVC	31315	a	0.21	-36.75	6.63	2.95E-08	7477	a	0.20	-10.09	13.89	0.47	NA	NA	NA	NA	NA	NA	11759	a	0.15	1.45	11.06	0.90	58172	a	0.19	-23.40	5.19	6.5E-06
CENPF,KCNK2	rs512597	1:215095003	FVC	60507	t	0.81	-24.26	4.12	3.92E-09	8420	t	0.47	12.11	8.47	0.15	9949	t	0.71	-9.99	6.75	0.14	11759	t	0.66	3.83	6.95	0.58	90635	t	0.72	-12.12	2.94	3.8E-05
C1orf140,DUSP10	rs6657854	1:221630555	FVC	60508	a	0.72	-19.89	3.49	1.18E-08	8421	a	0.89	-13.19	14.47	0.36	9954	a	0.65	-16.52	6.47	0.01	11759	a	0.74	-1.37	7.61	0.86	90642	a	0.71	-16.52	2.79	3.4E-09
ALX1,RASSF9	rs10779158	12:85724096	FVC	60506	a	0.34	18.72	3.31	1.52E-08	8420	a	0.75	-10.28	10.53	0.33	9934	a	0.51	7.08	6.18	0.25	11759	a	0.46	1.91	6.67	0.77	90619	a	0.41	12.39	2.59	1.7E-06
DCC	rs8089865	18:50957922	FVC	60509	a	0.59	20.57	3.23	1.95E-10	8420	a	0.64	12.14	8.57	0.16	9890	a	0.29	2.97	6.85	0.66	11759	a	0.45	9.50	6.89	0.17	90578	a	0.53	15.81	2.57	7.4E-10
EYA2	rs2236519	20:45529571	FVC	60508	a	0.38	-18.06	3.28	3.51E-08	8420	a	0.17	4.14	12.53	0.74	NA	NA	NA	NA	NA	NA	11759	a	0.34	-8.55	6.90	0.22	80687	a	0.36	-15.23	2.88	1.2E-07
AP3B1	rs12513481	5:77450828	FVC	60507	c	0.23	-25.01	3.74	2.15E-11	8420	c	0.14	-2.33	12.33	0.85	9950	c	0.07	-7.96	12.48	0.52	11759	c	0.25	-13.08	7.66	0.09	90636	c	0.22	-20.47	3.14	6.7E-11
DMRT2,SMARCA2	rs771924	9:1555835	FVC	60507	a	0.42	-18.4	3.18	7.16E-09	8421	a	0.62	-3.86	8.77	0.66	NA	NA	NA	NA	NA	NA	11759	a	0.38	4.65	7.00	0.51	88283	a	0.42	-12.46	2.60	1.7E-06
				African Ancestry						European Ancestry						Asian Ancestry						Hispanic Ancestry						Multiethnic					
				N	Coded Allele	Coded Allele Freq	Beta	SE	P-value	N	Coded Allele	Coded Allele Freq	Beta[c]	SE	P-value	N	Coded Allele	Coded Allele Freq	Beta[c]	SE	P-value	N	Coded Allele	Coded Allele Freq	Beta[c]	SE	P-value	N	Coded Allele	Coded Allele Freq	Beta[c]	SE	P-value
RYR2	rs3766889	1:237941781	FEV_1	8428	t	0.82	52.21	9.52	4.12E-08	55092	t	0.97	7.41	7.93	0.35	NA	t	0.93	7.39	21.92	0.74	11775	t	0.94	-16.66	14.26	0.24	77350	t	0.91	18.49	5.43	6.56E-04
ANKRD55,MAP3K1	rs11748173	5:55922145	FEV_1	8429	t	0.21	67.07	10.72	3.91E-10	60552	t	0.44	0.39	2.81	0.89	NA	t	0.10	-19.00	17.89	0.29	11774	t	0.45	-4.83	5.73	0.40	82758	t	0.42	2.52	2.43	0.30
EN1, MARCO	rs114962105	2:119660943	FVC	7099	a	0.98	178.48	32.44	3.77E-08	NA	NA	NA[e]	NA	NA	NA	NA	NA	NA[e]	NA	NA	NA	NA	NA	NA[e]	NA	NA	NA	NA[d]	a	0.98	178.48	32.44	3.77E-08
C2orf48, HPCAL1	rs139215025	2:10418806	Ratio	5653	a	0.01	-0.07	0.01	9.03E-11	NA	NA	NA[e]	NA	NA	NA	NA	NA	NA[e]	NA	NA	NA	NA[d]	a	0.01	-0.0009	0.01	0.94	NA[d]	a	0.01	-0.04	0.008	2.33E-06
CADPS	rs111793843	3:62386350	Ratio	7857	t	0.01	-0.05	0.008	1.97E-08	NA	NA	NA[e]	NA	NA	NA	NA	NA	NA[e]	NA	NA	NA	NA[d]	t	0.002	-0.001	0.01	0.94	NA[d]	t	0.009	-0.03	0.007	1.19E-06
HDC	rs180930492	15:50555681	Ratio	3852	t	0.01	-0.07	0.01	2.59E-09	NA	NA	NA[e]	NA	NA	NA	NA	NA	NA[e]	NA	NA	NA	NA[d]	t	0.007	0.006	0.014	0.67	NA[d]	t	0.008	-0.04	0.009	1.99E-05
LOC283867,CDH5	rs144296676	16:66060569	Ratio	6536	t	0.99	-0.03	0.006	5.35E-09	NA	NA	NA[e]	NA	NA	NA	NA	NA	NA[e]	NA	NA	NA	NA	NA	NA[e]	NA	NA	NA	NA[d]	t	0.99	-0.03	0.006	5.35E-09
CPT1C	rs147472287	19:50213396	Ratio	5653	t	0.01	-0.05	0.009	3.25E-08	NA	NA	NA[e]	NA	NA	NA	NA	NA	NA[e]	NA	NA	NA	NA[d]	t	0.01	0.009	0.01	0.52	NA[d]	t	0.01	-0.03	0.008	2.40E-05
				Hispanic Ancestry						European Ancestry						Asian Ancestry						African Ancestry						Multiethnic					
				N	Coded Allele	Coded Allele Freq	Beta[c]	SE	P-value	N	Coded Allele	Coded Allele Freq	Beta[c]	SE	P-value	N	Coded Allele	Coded Allele Freq	Beta[c]	SE	P-value	N	Coded Allele	Coded Allele Freq	Beta	SE	P-value	N	Coded Allele	Coded Allele Freq	Beta[c]	SE	P-value
DKFZp686O1327, PABPC1P2	rs6746679	2:147046592	FVC	11759	a	0.56	-37.36	6.67	2.17E-08	60507	a	0.57	2.89	3.19	3.65E-01	NA[f]	a	0.38	1.98	12.81	0.88	8421	a	0.75	1.09	9.78	0.91	82774	a	0.58	-3.88	2.70	0.15

[a]Nearest gene indicates gene either harboring the variant or nearest to it

[b]FEV_1 forced expiratory volume in 1 second (in ml), FVC forced vital capacity (in ml), Ratio FEV_1/FVC (as a proportion)

[c]Additive effect of variant on pulmonary function, adjusting for age, age^2, sex, height, $height^2$, smoking status, pack-years of smoking, center (if multi-center cohort), ancestral principal components, and weight (for FVC only)

[d]Met minweight requirement in African ancestry meta-analysis but not in Hispanic ancestry or multiethnic meta-analysis

[e]Monomorphic or minor allele frequency <0.004 in 1000 Genomes representative ancestral population; http://useast.ensembl.org/index.html

[f]Only available in 1 of 2 Asian studies included in meta-analysis; met minweight requirement in multiethnic meta-analysis but not in Asian ancestry meta-analysis

Supplementary Table 2. Associations between Lead Variants for Novel Loci and Discovery Pulmonary Function Phenotype and Look-Up of Associations with the Other Two Phenotypes by Ancestry in the CHARGE Consortium

			Discovery Phenotype				Look-Up Phenotype 1				Look-Up Phenotype 2			
Population	rsID	Chr:Pos	Phenotype[a]	Beta[b]	SE	P-value	Phenotype[a]	Beta[b]	SE	P-value	Phenotype[a]	Beta[b]	SE	P-value
Multiethnic	rs12092943	1:204434927	FEV1	-14.57	2.67	4.83E-08	FVC	-13.72	3.06	7.24E-06	Ratio	-0.001	4.00E-04	0.008
Multiethnic	1:221765779:C_CA	1:221765779:C_CA	FEV1	-36.25	6.57	3.38E-08	FVC	-42.56	7.52	1.52E-08	Ratio	-0.002	0.001	0.08
Multiethnic	rs7899503	10:65087468	FEV1	21.16	2.84	8.70E-14	FVC	17.20	3.25	1.24E-07	Ratio	0.002	4.00E-04	9.75E-07
Multiethnic	rs11057793	12:125230287	FEV1	17.66	3.24	4.78E-08	FVC	17.66	3.70	1.82E-06	Ratio	0.001	5.00E-04	0.01
Multiethnic	rs772920	12:56390364	FEV1	13.86	2.49	2.48E-08	FVC	10.92	2.84	0.0001	Ratio	0.002	4.00E-04	1.19E-05
Multiethnic	rs62070631	17:29087285	FEV1	20.26	3.64	2.57E-08	FVC	20.16	4.16	1.25E-06	Ratio	9.00E-04	5.00E-04	0.10
Multiethnic	rs186806998	17:43682323	FEV1	29.50	4.70	3.47E-10	FVC	30.52	5.36	1.20E-08	Ratio	0.002	7.00E-04	0.02
Multiethnic	rs199525	17:44847834	FEV1	18.85	3.08	9.59E-10	FVC	20.32	3.52	7.52E-09	Ratio	0.001	5.00E-04	0.03
Multiethnic	rs7243351	18:20148531	FEV1	12.31	2.25	4.69E-08	FVC	12.12	2.58	2.69E-06	Ratio	7.00E-04	3.00E-04	0.03
Multiethnic	rs513953	18:8801351	FEV1	-14.50	2.58	1.96E-08	FVC	-11.49	2.97	0.0001	Ratio	-0.002	4.00E-04	2.89E-06
Multiethnic	rs963406	2:42355947	FEV1	-23.13	4.18	3.17E-08	FVC	-25.60	4.78	8.65E-08	Ratio	-0.001	6.00E-04	0.08
Multiethnic	rs6138639	20:25669052	FEV1	17.91	2.85	3.17E-10	FVC	16.70	3.26	2.94E-07	Ratio	0.001	4.00E-04	0.01
Multiethnic	rs1737889	20:31042176	FEV1	-16.82	3.07	4.17E-08	FVC	-10.92	3.52	0.002	Ratio	-0.002	5.00E-04	2.54E-06
Multiethnic	rs79294353	3:57494433	FEV1	-29.56	5.05	4.82E-09	FVC	-22.03	5.78	0.0001	Ratio	-0.003	7.00E-04	2.19E-05
Multiethnic	rs6778584	3:98815640	FEV1	12.98	2.37	4.51E-08	FVC	15.77	2.72	6.56E-09	Ratio	2.00E-04	4.00E-04	0.64
Multiethnic	rs111898810	4:146174040	FEV1	-20.24	3.61	2.14E-08	FVC	-18.67	4.13	6.10E-06	Ratio	-0.002	5.00E-04	0.001
Multiethnic	rs9407640	9:1574877	FEV1	-14.48	2.65	4.77E-08	FVC	-16.82	3.03	2.87E-08	Ratio	0	4.00E-04	0.94
Multiethnic	rs2821332	1:200085714	FVC	14.50	2.51	7.65E-09	FEV1	6.51	2.19	0.003	Ratio	-8.00E-04	3.00E-04	0.01
Multiethnic	rs12046746	1:221635207	FVC	-16.99	2.81	1.41E-09	FEV1	-7.59	2.45	0.002	Ratio	0.001	4.00E-04	0.0002
Multiethnic	1:237929787:T_TCA	1:237929787:T_TCA	FVC	-37.17	6.79	4.46E-08	FEV1	-30.69	5.92	2.16E-07	Ratio	-9.00E-04	9.00E-04	0.35
Multiethnic	10:77002679:TC_T	10:77002679:TC_T	FVC	22.36	4.10	4.89E-08	FEV1	15.17	3.61	2.71E-05	Ratio	-3.00E-04	5.00E-04	0.58
Multiethnic	rs73025192	11:127995904	FVC	-24.18	4.28	1.63E-08	FEV1	-17.70	3.73	2.04E-06	Ratio	-3.00E-04	6.00E-04	0.61
Multiethnic	rs7971039	12:85724305	FVC	16.36	2.88	1.44E-08	FEV1	12.80	2.52	3.79E-07	Ratio	-1.00E-04	4.00E-04	0.82
Multiethnic	rs11107184	12:94184082	FVC	14.89	2.71	3.87E-08	FEV1	10.47	2.37	9.65E-06	Ratio	-3.00E-04	4.00E-04	0.42
Multiethnic	rs10859698	12:94852628	FVC	21.19	3.84	3.49E-08	FEV1	14.63	3.36	1.33E-05	Ratio	-5.00E-04	5.00E-04	0.30
Multiethnic	rs4775429	15:46722435	FVC	40.23	7.21	2.45E-08	FEV1	25.69	6.26	4.12E-05	Ratio	-0.002	0.001	0.11
Multiethnic	rs8025774	15:67483276	FVC	-20.87	2.92	9.34E-13	FEV1	-10.49	2.55	3.84E-05	Ratio	0.001	4.00E-04	0.001
Multiethnic	rs3973397	16:70040398	FVC	-22.38	4.05	3.31E-08	FEV1	-13.88	3.52	8.07E-05	Ratio	4.00E-04	5.00E-04	0.43
Multiethnic	rs55771535	16:72252097	FVC	-29.88	4.83	6.38E-10	FEV1	-18.82	4.21	7.89E-06	Ratio	0.001	6.00E-04	0.10
Multiethnic	rs8067511	17:37611352	FVC	18.30	3.20	1.08E-08	FEV1	14.77	2.79	1.21E-07	Ratio	3.00E-04	4.00E-04	0.46
Multiethnic	rs150741403	17:43682405	FVC	35.83	5.97	1.94E-09	FEV1	31.95	5.24	1.08E-09	Ratio	0.001	8.00E-04	0.19
Multiethnic	rs199525	17:44847834	FVC	20.32	3.52	7.52E-09	FEV1	18.85	3.08	9.59E-10	Ratio	0.001	5.00E-04	0.03
Multiethnic	rs7238093	18:20728158	FVC	18.15	3.13	6.78E-09	FEV1	14.11	2.73	2.36E-07	Ratio	1.00E-04	4.00E-04	0.77
Multiethnic	rs8089865	18:50957922	FVC	15.81	2.57	7.38E-10	FEV1	10.65	2.24	2.05E-06	Ratio	-2.00E-04	3.00E-04	0.58
Multiethnic	rs17034666	2:109571508	FVC	-27.93	4.96	1.81E-08	FEV1	-19.30	4.30	7.30E-06	Ratio	6.00E-04	6.00E-04	0.36
Multiethnic	rs1404098	3:98806782	FVC	15.93	2.73	5.45E-09	FEV1	12.95	2.38	5.58E-08	Ratio	1.00E-04	4.00E-04	0.70
Multiethnic	rs72776440	5:77440196	FVC	-21.30	3.21	3.20E-11	FEV1	-14.41	2.80	2.77E-07	Ratio	4.00E-04	4.00E-04	0.32
Multiethnic	rs11759026	6:126792095	FVC	-20.20	3.44	4.35E-09	FEV1	-12.73	3.00	2.22E-05	Ratio	7.00E-04	4.00E-04	0.11
Multiethnic	rs55905169	7:15506529	FVC	-17.57	3.09	1.28E-08	FEV1	-13.11	2.69	1.13E-06	Ratio	2.00E-04	4.00E-04	0.70
Multiethnic	rs9407640	9:1574877	FVC	-16.82	3.03	2.87E-08	FEV1	-14.48	2.65	4.77E-08	Ratio	0	4.00E-04	0.94
Multiethnic	rs11591179	1:160206067	Ratio	-0.0019	0.0003	3.48E-08	FEV1	-6.82	2.26	0.002	FVC	-0.76	2.58	0.77
Multiethnic	rs2293871	10:124273671	Ratio	0.0023	0.0004	1.51E-08	FEV1	-3.76	2.70	0.16	FVC	-13.91	3.08	6.38E-06
Multiethnic	rs75159994	10:64916064	Ratio	-0.0025	0.0004	6.09E-09	FEV1	-20.50	2.88	1.03E-12	FVC	-14.69	3.30	8.48E-06

Multiethnic	11:73280955:GA_G	11:73280955:GA_G	Ratio	0.0035	0.0006	2.74E-08	FEV1	16.08	4.11	9.30E-05	FVC	3.79	4.70	0.42
Multiethnic	rs4444235	14:54410919	Ratio	0.0019	0.0004	4.03E-08	FEV1	2.96	2.37	0.21	FVC	-5.10	2.72	0.06
Multiethnic	rs9636166	19:31829613	Ratio	0.003	0.0005	3.25E-09	FEV1	10.25	3.50	0.003	FVC	-0.59	4.01	0.88
Multiethnic	rs72904209	2:157046432	Ratio	0.0029	0.0005	3.09E-08	FEV1	15.36	3.62	2.15E-05	FVC	3.55	4.15	0.39
Multiethnic	rs4820216	22:20854161	Ratio	-0.0034	0.0005	2.61E-10	FEV1	-12.69	3.55	0.0003	FVC	-0.98	4.06	0.81
Multiethnic	rs28723417	3:29431565	Ratio	0.0022	0.0004	1.77E-08	FEV1	8.54	2.57	0.0009	FVC	0.53	2.93	0.86
Multiethnic	rs80217917	3:99359368	Ratio	-0.0028	0.0005	2.58E-08	FEV1	-3.39	3.33	0.31	FVC	6.34	3.82	0.10
Multiethnic	rs28520091	4:7846240	Ratio	0.0021	0.0004	8.40E-09	FEV1	3.95	2.53	0.12	FVC	-3.57	2.90	0.22
Multiethnic	rs9350408	6:22021373	Ratio	-0.0026	0.0003	7.45E-14	FEV1	-6.32	2.31	0.006	FVC	3.58	2.65	0.18
Multiethnic	rs2451951	9:109496630	Ratio	0.0019	0.0003	2.36E-08	FEV1	2.52	2.27	0.27	FVC	-6.67	2.60	0.01
Multiethnic	rs10965947	9:23588583	Ratio	0.0022	0.0004	2.70E-09	FEV1	12.20	2.41	4.30E-07	FVC	4.81	2.76	0.08
European	rs10779158	12:85724096	FEV1	15.89	2.90	4.36E-08	FVC	18.72	3.31	1.52E-08	Ratio	3.00E-04	4.00E-04	0.53
European	rs143246821	17:43685698	FEV1	30.58	4.99	9.06E-10	FVC	30.54	5.66	6.83E-08	Ratio	2.00E-03	7.00E-04	0.01
European	rs916888	17:44863133	FEV1	20.53	3.48	3.76E-09	FVC	21.56	3.96	5.05E-08	Ratio	1.30E-03	5.00E-04	0.01
European	rs252746	5:77392117	FEV1	20.05	3.45	6.19E-09	FVC	25.37	3.93	1.08E-10	Ratio	2.00E-04	5.00E-04	0.74
European	rs12724426	1:146494027	FVC	-36.75	6.63	2.95E-08	FEV1	-23.80	5.84	4.66E-05	Ratio	6.00E-04	9.00E-04	0.46
European	rs512597	1:215095003	FVC	-24.26	4.12	3.92E-09	FEV1	-13.34	3.62	2.29E-04	Ratio	1.20E-03	5.00E-04	0.03
European	rs6657854	1:221630555	FVC	-19.89	3.49	1.18E-08	FEV1	-9.65	3.06	1.61E-03	Ratio	1.20E-03	4.00E-04	0.01
European	rs10779158	12:85724096	FVC	18.72	3.31	1.52E-08	FEV1	15.89	2.90	4.36E-08	Ratio	3.00E-04	4.00E-04	0.53
European	rs8089865	18:50957922	FVC	20.57	3.23	1.95E-10	FEV1	14.53	2.84	3.15E-07	Ratio	-3.00E-04	4.00E-04	0.46
European	rs2236519	20:45529571	FVC	-18.06	3.28	3.51E-08	FEV1	-11.01	2.88	1.32E-04	Ratio	2.00E-04	4.00E-04	0.57
European	rs12513481	5:77450828	FVC	-25.01	3.74	2.15E-11	FEV1	-18.87	3.28	8.99E-09	Ratio	0	5.00E-04	0.96
European	rs771924	9:1555835	FVC	-18.40	3.18	7.16E-09	FEV1	-14.40	2.79	2.53E-07	Ratio	2.00E-04	4.00E-04	0.69
European	rs1353531	19:31846907	Ratio	-0.0033	0.0006	4.53E-08	FEV1	-13.08	4.02	0.001	FVC	-1.57	4.58	0.73
European	rs4820216	22:20854161	Ratio	-0.0037	0.0006	1.53E-09	FEV1	-18.24	4.05	6.77E-06	FVC	-5.37	4.61	0.24
European	rs17666332	3:29469675	Ratio	0.0025	0.0005	4.76E-08	FEV1	11.27	3.07	0.0002	FVC	1.37	3.50	0.69
European	rs28520091	4:7846240	Ratio	0.0026	0.0004	2.17E-09	FEV1	4.11	2.94	0.16	FVC	-4.90	3.35	0.14
European	rs1928168	6:22017738	Ratio	0.0031	0.0004	6.74E-14	FEV1	12.52	2.77	6.12E-06	FVC	0.27	3.16	0.93
European	rs9351637	6:67863782	Ratio	0.0024	0.0004	2.89E-08	FEV1	2.07	2.96	0.48	FVC	-7.90	3.37	0.02
European	rs1404154	7:146651409	Ratio	-0.0327	0.0059	2.80E-08	FEV1	-112.00	51.95	0.03	FVC	9.80	57.85	0.87
African	rs3766889	1:237941781	FEV1	52.21	9.52	4.12E-08	FVC	54.87	11.10	7.77E-07	Ratio	0.003	0.002	0.09
African	rs11748173	5:55922145	FEV1	67.07	10.72	3.91E-10	FVC	66.84	12.49	8.82E-08	Ratio	0.003	0.002	0.07
African	rs114962105	2:119660943	FVC	178.48	32.44	3.77E-08	FEV1	133.05	28.67	3.48E-06	Ratio	0.001	0.005	0.78
African	rs180930492	15:50555681	Ratio	-0.07	0.01	2.59E-09	FEV1	-161.82	67.31	0.02	FVC	84.52	78.20	0.28
African	rs144296676	16:66060569	Ratio	-0.03	0.006	5.35E-09	FEV1	-65.34	34.93	0.06	FVC	49.38	41.22	0.23
African	rs147472287	19:50213396	Ratio	-0.05	0.009	3.25E-08	FEV1	-33.91	54.95	0.54	FVC	51.70	60.30	0.39
African	rs139215025	2:10418806	Ratio	-0.07	0.01	9.03E-11	FEV1	-103.64	61.33	0.09	FVC	51.67	71.39	0.47
African	rs111793843	3:62386350	Ratio	-0.05	0.008	1.97E-08	FEV1	-82.34	54.52	0.13	FVC	-51.99	57.32	0.36
Hispanic	rs6746679	2:147046592	FVC	-37.36	6.67	2.17E-08	FEV1	-26.31	5.73	4.44E-06	Ratio	7.00E-04	8.00E-04	0.3666

[a] FEV_1 forced expiratory volume in 1 second (in ml), FVC forced vital capacity (in ml), Ratio FEV_1/FVC (as a proportion)
[b] Additive effect of variant on pulmonary function, adjusting for age, age^2, sex, height, $height^2$, smoking status, pack-years of smoking, center (if multi-center cohort), ancestral principal components, and weight (for FVC only)

Supplementary Table 3. Lead Variants for Novel Loci Discovered in the Multiethnic Meta-Analysis and Look-Up in Ancestry-Specific Meta-Analyses in the CHARGE Consortium

				Multiethnic						European Ancestry						African Ancestry						Asian Ancestry						Hispanic Ancestry					
Nearest Gene[a]	rsID	Chr:Pos	Phenotype[b]	N	Coded Allele	Coded Allele Freq	Beta[c]	SE	P-value	N	Coded Allele	Coded Allele Freq	Beta[c]	SE	P-value	N	Coded Allele	Coded Allele Freq	Beta[c]	SE	P-value	N	Coded Allele	Coded Allele Freq	Beta[c]	SE	P-value	N	Coded Allele	Coded Allele Freq	Beta[c]	SE	P-value
PIK3C2B	rs12092943	1:204434927	FEV1	90703	t	0.74	-14.57	2.67	4.83E-08	60549	t	0.82	-13.41	3.61	2.05E-04	8429	t	0.74	-22.07	8.02	0.006	9950	t	0.50	-14.92	5.39	0.006	11775	t	0.87	-11.69	8.55	0.17
C1orf140,DUSP10	1:221765779:C_CA	1:221765779:C_CA	FEV1	55548	i	0.12	-36.25	6.57	3.38E-08	47120	i	0.03	-42.65	9.49	7.02E-06	8428	i	0.20	-30.37	9.09	8.36E-04	NA	NA	NA	NA	NA	NA	NA	NA	NA	NA	NA	NA
JMJD1C	rs7899503	10:65087468	FEV1	90712	c	0.25	21.16	2.84	8.70E-14	60551	c	0.17	17.35	3.99	1.35E-05	8429	c	0.11	29.14	13.36	0.03	9957	c	0.38	28.29	5.61	4.56E-07	11775	c	0.33	19.86	6.47	0.002
NCOR2,SCARB1	rs11057793	12:125230287	FEV1	78930	t	0.75	17.66	3.24	4.78E-08	58727	t	0.76	15.28	3.85	7.26E-05	8428	t	0.86	28.92	13.95	0.04	NA	NA	NA	NA	NA	NA	11775	t	0.70	22.12	6.59	7.93E-04
RAB5B	rs772920	12:56390364	FEV1	90572	c	0.72	13.86	2.49	2.48E-08	60551	c	0.68	12.75	3.03	2.57E-05	8429	c	0.86	14.81	10.74	0.17	9817	c	0.78	13.39	6.64	0.04	11775	c	0.78	19.66	6.84	0.004
SUZ12P1	rs62070631	17:29087285	FEV1	82835	a	0.15	20.26	3.64	2.57E-08	60549	a	0.17	19.63	4.12	1.93E-06	8428	a	0.08	28.47	15.48	0.07	2083[d]	a	0.08	43.78	19.56	0.03	11775	a	0.09	14.33	10.03	0.15
CRHR1	rs186806998	17:43682323	FEV1	43927	t	0.82	29.5	4.7	3.47E-10	27565	t	0.79	36.39	5.96	9.97E-10	4587	t	0.87	3.15	17.59	0.86	NA	NA	NA[e]	NA	NA	NA	11775	t	0.86	21.62	8.5	0.01
WNT3	rs199525	17:44847834	FEV1	80753	t	0.8	18.85	3.08	9.59E-10	60550	t	0.79	19.16	3.42	2.07E-08	8428	t	0.89	10.49	14.25	0.46	NA	NA	NA[e]	NA	NA	NA	11775	t	0.85	19.82	8.23	0.02
CTAGE1,RBBP8	rs7243351	18:20148531	FEV1	90708	t	0.45	12.31	2.25	4.69E-08	60551	t	0.50	14.09	2.78	3.96E-07	8429	t	0.18	16.13	9.97	0.11	9953	t	0.43	13.22	5.41	0.01	11775	t	0.28	-0.66	6.58	0.92
SOGA2	rs513953	18:8801351	FEV1	82781	a	0.29	-14.5	2.58	1.96E-08	60551	a	0.25	-16.86	3.2	1.42E-07	8428	a	0.49	-11.72	7.15	0.1	2028[d]	a	0.41	-2.57	11.18	0.82	11774	a	0.28	-11.29	6.32	0.07
PKDCC, EML4	rs963406	2:42355947	FEV1	80755	a	0.12	-23.13	4.18	3.17E-08	60552	a	0.13	-24.23	4.51	7.69E-08	8428	a	0.05	-26.98	21.88	0.22	NA	NA	NA[e]	NA	NA	NA	11775	a	0.06	-12.62	12.99	0.33
ZNF337	rs6138639	20:25669052	FEV1	90593	c	0.79	17.91	2.85	3.17E-10	60552	c	0.82	19.38	3.75	2.37E-07	8429	c	0.90	8.3	12.01	0.49	9837	c	0.68	20.63	5.8	3.74E-04	11775	c	0.86	10.29	8.02	0.2
C20orf112	rs1737889	20:31042176	FEV1	80755	t	0.22	-16.82	3.07	4.17E-08	60551	t	0.21	-18.61	3.73	6.14E-07	8429	t	0.30	-9.92	8.2	0.23	NA	NA	NA	NA	NA	NA	11775	t	0.20	-15.48	7.13	0.03
DNAH12	rs79294353	3:57494433	FEV1	79170	a	0.92	-29.56	5.05	4.82E-09	58967	a	0.91	-28.45	5.77	8.27E-07	8428	a	0.96	-41.43	25.13	0.1	NA	NA	NA[e]	NA	NA	NA	11775	a	0.93	-31.44	11.45	0.006
DCBLD2,MIR548G	rs6778584	3:98815640	FEV1	90393	t	0.7	12.98	2.37	4.51E-08	60549	t	0.74	12.34	3.12	7.71E-05	8428	t	0.55	9.16	7.12	0.2	9641	t	0.72	13.77	6.14	0.02	11775	t	0.65	17.19	5.91	0.004
OTUD4,SMAD1	rs111898810	4:146174040	FEV1	80752	a	0.2	-20.24	3.61	2.14E-08	60549	a	0.22	-20.92	4.05	2.41E-07	8428	a	0.09	-7.37	16.92	0.66	NA	NA	NA[e]	NA	NA	NA	11775	a	0.12	-20.52	9.08	0.02
DMRT2,SMARCA2	rs9407640	9:1574877	FEV1	80754	c	0.41	-14.48	2.65	4.77E-08	60550	c	0.45	-14.18	3.11	5.17E-06	8429	c	0.36	-23.92	7.94	0.003	NA	NA	NA	NA	NA	NA	11775	c	0.31	-9.3	6.59	0.16
NR5A2	rs2821332	1:200085714	FVC	90642	a	0.47	14.5	2.51	7.65E-09	60508	a	0.43	16.94	3.15	7.51E-08	8420	a	0.34	18.47	8.59	0.03	9955	a	0.72	11.47	6.85	0.09	11759	a	0.47	4.29	6.58	0.51
C1orf140,DUSP10	rs12046746	1:221635207	FVC	90427	c	0.71	-16.99	2.81	1.41E-09	60506	c	0.72	-19.81	3.49	1.33E-08	8420	c	0.89	-23.45	15.39	0.13	9742	c	0.64	-17.27	6.53	0.008	11759	c	0.74	-1.44	7.65	0.85
RYR2	1:237929787:T_TCA	1:237929787:T_TCA	FVC	48215	i	0.11	-37.17	6.79	4.46E-08	39794	i	0.05	-25.44	8.65	0.003	8421	i	0.19	-55.99	10.96	3.27E-07	NA	NA	NA	NA	NA	NA	NA	NA	NA	NA	NA	NA
COMTD1,ZNF503-AS1	10:77002679:TC_T	10:77002679:TC_T	FVC	55498	d	0.22	22.36	4.1	4.89E-08	47077	d	0.23	21.71	4.25	3.17E-07	8421	d	0.11	31.1	15.64	0.05	NA	NA	NA	NA	NA	NA	NA	NA	NA	NA	NA	NA
KIRREL3-AS3,ETS1	rs73025192	11:127995904	FVC	90529	t	0.12	-24.18	4.28	1.63E-08	60507	t	0.08	-26.71	5.82	4.45E-06	8420	t	0.22	-23.88	10.53	0.02	9844	t	0.03	-20.3	17.34	0.24	11758	t	0.16	-19.52	8.88	0.03
ALX1,RASSF9	rs7971039	12:85724305	FVC	90639	a	0.26	16.36	2.88	1.44E-08	60507	a	0.28	18.6	3.46	7.87E-08	8420	a	0.17	24.76	11.06	0.03	9953	a	0.12	7.7	9.33	0.41	11759	a	0.24	7.23	7.63	0.34
CRADD	rs11107184	12:94184082	FVC	88548	t	0.34	14.89	2.71	3.87E-08	60507	t	0.30	14.16	3.46	4.25E-05	8421	t	0.33	5.13	8.63	0.55	7861[d]	t	0.55	15.05	7.13	0.03	11759	t	0.31	24.48	7.13	5.98E-04
CCDC41	rs10859698	12:94852628	FVC	88159	a	0.21	21.19	3.84	3.49E-08	60499	a	0.10	23.28	5.89	7.71E-05	8420	a	0.13	8.67	12.28	0.48	7481[d]	a	0.43	26.51	7.32	0.0003	11759	a	0.18	15.55	8.57	0.07
SQRDL,SEMA6D	rs4775429	15:46722435	FVC	79231	t	0.17	40.23	7.21	2.45E-08	59051	t	0.02	35.25	14.95	0.02	8421	t	0.27	28.71	9.85	0.004	NA	NA	NA[e]	NA	NA	NA	11759	t	0.08	72.08	15.02	1.61E-06
SMAD3	rs8025774	15:67483276	FVC	88524	t	0.29	-20.87	2.92	9.34E-13	60490	t	0.24	-17.82	3.71	1.57E-06	8420	t	0.13	-18.85	13.58	0.17	7855[d]	NA	NA	NA	NA	NA	11759	t	0.32	-21.78	7.23	0.003
PDXDC2P	rs3973397	16:70040398	FVC	44921	a	0.48	-22.38	4.05	3.31E-08	24742	a	0.39	-27.35	5.53	7.61E-07	8420	a	0.72	-23.42	11.78	0.05	NA	NA	NA	NA	NA	NA	11759	a	0.53	-14.29	6.89	0.04
PMFBP1,ZFHX3	rs55771535	16:72252097	FVC	80688	a	0.13	-29.88	4.83	6.38E-10	60508	a	0.07	-28.02	6.28	8.04E-06	8421	a	0.26	-35.04	9.43	0.0002	NA	NA	NA[e]	NA	NA	NA	11759	a	0.10	-28.14	12.76	0.03
MED1, CDK12	rs8067511	17:37611352	FVC	90632	t	0.8	18.3	3.2	1.08E-08	60508	t	0.84	16.47	4.41	1.85E-04	8421	t	0.74	25.71	9.95	0.01	9944	t	0.76	11.39	7.22	0.11	11759	t	0.76	27.26	7.69	3.97E-04
CRHR1	rs150741403	17:43682405	FVC	43896	c	0.85	35.83	5.97	1.94E-09	27556	c	0.84	40.46	7.72	1.61E-07	4581	c	0.87	31.83	22.43	0.16	NA	NA	NA[e]	NA	NA	NA	11759	c	0.87	28.34	10.36	0.006
WNT3	rs199525	17:44847834	FVC	80686	t	0.8	20.32	3.52	7.52E-09	60506	t	0.79	20.03	3.89	2.58E-07	8421	t	0.89	22.91	16.41	0.16	NA	NA	NA[e]	NA	NA	NA	11759	t	0.85	21.18	9.52	0.03
CABLES1	rs7238093	18:20728158	FVC	90240	a	0.22	18.15	3.13	6.78E-09	60508	a	0.20	14.82	4.01	2.18E-04	8420	a	0.13	59.74	13.13	5.34E-06	9553	a	0.18	21.28	8.17	0.009	11759	a	0.34	13.87	7.26	0.06
DCC	rs8089865	18:50957922	FVC	90578	a	0.53	15.81	2.57	7.38E-10	60509	a	0.59	20.57	3.23	1.95E-10	8420	a	0.64	12.14	8.57	0.16	9890	a	0.29	2.97	6.85	0.66	11759	a	0.45	9.5	6.89	0.17
EDAR	rs17034666	2:109571508	FVC	82747	a	0.23	-27.93	4.96	1.81E-08	60508	a	0.05	-28.94	7.7	1.70E-04	8421	a	0.15	-6.97	12.11	0.57	2059[d]	a	0.91	-33.22	21.59	0.12	11759	a	0.38	-35.69	8.23	1.44E-05
DCBLD2,MIR548G	rs1404098	3:98806782	FVC	90334	a	0.71	15.93	2.73	5.45E-09	60507	a	0.74	18.85	3.56	1.22E-07	8420	a	0.61	10.86	8.43	0.2	9648	a	0.72	9.96	7.03	0.16	11759	a	0.66	14.14	6.91	0.04
AP3B1	rs72776440	5:77440196	FVC	90631	c	0.21	-21.3	3.21	3.20E-11	60508	c	0.22	-24.97	3.75	2.63E-11	8421	c	0.10	3.8	16.13	0.81	9943	c	0.06	-10.25	13.26	0.44	11759	c	0.24	-15.02	7.83	0.06
CENPW,RSPO3	rs11759026	6:126792095	FVC	80687	a	0.72	-20.2	3.44	4.35E-09	60507	a	0.76	-19.5	4.11	2.07E-06	8421	a	0.86	-12.13	13.84	0.38	NA	NA	NA	NA	NA	NA	11759	a	0.59	-24.4	7.08	5.66E-04
AGMO	rs55905169	7:15506529	FVC	90511	c	0.31	-17.57	3.09	1.28E-08	60508	c	0.17	-11.97	4.24	4.75E-03	8420	c	0.13	-24.39	12.97	0.06	9824	c	0.63	-14.46	6.39	0.02	11759	c	0.40	-36.13	7.31	7.72E-07
DMRT2,SMARCA2	rs9407640	9:1574877	FVC	80686	c	0.42	-16.82	3.03	2.87E-08	60507	c	0.45	-18.7	3.54	1.25E-07	8420	c	0.36	-18.77	9.21	0.04	NA	NA	NA	NA	NA	NA	11759	c	0.31	-6.72	7.64	0.38
DCAF8	rs11591179	1:160206067	Ratio	90624	t	0.45	-0.002	0.0003	3.48E-08	60530	t	0.46	-0.002	4.00E-04	1.88E-06	8424	t	0.26	-0.002	0.001	0.16	9906	t	0.50	-0.002	9.00E-04	0.02	11764	t	0.42	-0.001	8.00E-04	0.23
HTRA1	rs2293871	10:124273671	Ratio	90481	t	0.23	0.002	0.0004	1.51E-08	60529	t	0.18	0.002	5.00E-04	6.22E-05	8423	t	0.16	0.004	0.002	0.03	9765	t	0.41	0.002	9.00E-04	0.008	11764	t	0.19	0.002	0.001	0.03
JMJD1C	rs75159994	10:64916064	Ratio	86988	t	0.77	-0.003	0.0004	6.09E-09	58950	t	0.85	-0.003	6.00E-04	1.09E-05	8424	t	0.87	-0.003	0.002	0.11	7850[d]	t	0.62	-0.003	0.001	0.01	11764	t	0.65	-0.002	9.00E-04	0.01
FAM168A	11:73280955:GA_G	11:73280955:GA_G	Ratio	55521	d	0.2	0.004	0.0006	2.74E-08	47098	d	0.12	0.003	7.00E-04	4.64E-06	8423	d	0.40	0.004	0.001	0.002	NA	NA	NA	NA	NA	NA	NA	NA	NA	NA	NA	NA
DDHD1,MIR5580	rs4444235	14:54410919	Ratio	80712	t	0.54	0.002	0.0004	4.03E-08	60525	t	0.54	0.002	4.00E-04	1.57E-07	8423	t	0.64	0.002	0.001	0.09	NA	NA	NA	NA	NA	NA	11764	t	0.53	0.001	8.00E-04	0.21
TSHZ3	rs9636166	19:31829613	Ratio	80714	a	0.86	0.003	0.0005	3.25E-09	60526	a	0.87	0.003	6.00E-04	4.08E-07	8424	a	0.90	-4.00E-04	0.002	0.84	NA	NA	NA	NA	NA	NA	11764	a	0.81	0.004	0.001	3.72E-04
KCNJ3, NR4A2	rs72904209	2:157046432	Ratio	90453	t	0.88	0.003	0.0005	3.09E-08	60529	t	0.87	0.003	6.00E-04	1.29E-06	8423	t	0.94	0.004	0.003	0.13	9737	t	0.97	-0.003	0.003	0.3	11764	t	0.89	0.004	0.001	0.002
KLHL22,MED15	rs4820216	22:20854161	Ratio	82714	t	0.13	-0.003	0.0005	2.61E-10	60528	t	0.15	-0.004	6.00E-04	1.53E-09	8423	t	0.07	-6.00E-04	0.003	0.8	1999[d]	t	0.12	0.002	0.003	0.47	11764	t	0.10	-0.004	0.001	0.006

RBMS3	rs28723417	3:29431565	Ratio	90358	a	0.74	0.002	0.0004	1.77E-08	60532	a	0.72	0.003	5.00E-04	5.28E-08	8423	a	0.67	4.00E-04	0.001	0.78	9639	a	0.90	0.002	0.002	0.19	11764	a	0.79	0.002	0.001	0.08
DCBLD2,MIR548G	rs80217917	3:99359368	Ratio	90617	t	0.88	-0.003	0.0005	2.58E-08	60531	t	0.89	-0.003	7.00E-04	3.09E-06	8423	t	0.87	-0.004	0.002	0.02	9899	t	0.88	-0.002	0.001	0.14	11764	t	0.85	-0.002	0.001	0.1
AFAP1	rs28520091	4:7846240	Ratio	80715	t	0.44	0.002	0.0004	8.40E-09	60527	t	0.48	0.003	4.00E-04	2.17E-09	8424	t	0.25	0.002	0.002	0.13	NA	NA	NA	NA	NA	NA	11764	t	0.37	0.001	8.00E-04	0.57
LINC00340	rs9350408	6:22021373	Ratio	82761	t	0.51	-0.003	0.0003	7.45E-14	60526	t	0.54	-0.003	4.00E-04	9.77E-12	8424	t	0.48	-0.003	0.001	0.01	2047[d]	t	0.46	0.0001	0.002	0.9634	11764	t	0.41	-0.002	8.00E-04	0.01
TMEM38B,ZNF462	rs2451951	9:109496630	Ratio	88436	t	0.47	0.002	0.0003	2.36E-08	58706	t	0.49	0.002	4.00E-04	1.22E-06	8424	t	0.24	0.003	0.002	0.09	9542	t	0.32	0.002	0.001	0.03	11764	t	0.53	0.001	8.00E-04	0.22
FLJ35282,ELAVL2	rs10965947	9:23588583	Ratio	90475	t	0.39	0.002	0.0004	2.70E-09	60531	t	0.45	0.002	4.00E-04	1.47E-05	8424	t	0.29	0.003	0.001	0.07	9756	t	0.16	0.003	0.0012	0.02	11764	t	0.34	0.003	9.00E-04	0.003

[a]Nearest gene indicates gene either harboring the variant or nearest to it
[b]FEV_1 forced expiratory volume in 1 second (in ml), FVC forced vital capacity (in ml), Ratio FEV_1/FVC (as a proportion)
[c]Additive effect of variant on pulmonary function, adjusting for age, age^2, sex, height, $height^2$, smoking status, pack-years of smoking, center (if multi-center cohort), ancestral principal components, and weight (for FVC only)
[d]Only available in 1 of 2 Asian studies included in meta-analysis; met minimum sample size requirement in multiethnic meta-analysis but not in Asian ancestry meta-analysis
[e]Monomorphic or minor allele frequency < 0.004 in 1000 Genomes East Asian populations; http://useast.ensembl.org/index.html

Supplementary Table 4. Results from Han-Eskin[a] Random-effects Model for Lead Variants for 47 Novel Loci Discovered in Multiethnic Fixed-effects Model and 6 Additional Loci Identified Uniquely in the Han-Eskin Model

				Multiethnic Fixed Effects - Cohort Files[b]			Multiethnic Fixed Effects - Ancestry Files[b]			Multiethnic Han Eskin Random Effects - Ancestry Files[b]
Nearest Gene[c]	rsID	Chr:Pos	Phenotype[d]	Beta[e]	SE	P-value	Beta[e]	SE	P-value	P-value
PIK3C2B	rs12092943	1:204434927	FEV1	-14.57	2.67	4.83E-08	-14.61	2.78	1.44E-07	7.13E-08
C1orf140,DUSP10	1:221765779:C_CA	1:221765779:C_CA	FEV1	-36.25	6.57	3.38E-08	-36.02	6.84	1.38E-07	4.24E-08
JMJD1C	rs7899503	10:65087468	FEV1	21.16	2.84	8.70E-14	21.33	2.94	4.36E-13	1.58E-13
NCOR2,SCARB1	rs11057793	12:125230287	FEV1	17.66	3.24	4.78E-08	17.81	3.39	1.48E-07	6.60E-08
RAB5B	rs772920	12:56390364	FEV1	13.86	2.49	2.48E-08	13.93	2.60	8.49E-08	3.55E-08
SUZ12P1	rs62070631	17:29087285	FEV1	20.26	3.64	2.57E-08	19.39	3.90	6.55E-07	2.16E-07
CRHR1	rs186806998	17:43682323	FEV1	29.50	4.70	3.47E-10	29.13	4.91	2.86E-09	5.27E-10
WNT3	rs199525	17:44847834	FEV1	18.85	3.08	9.59E-10	18.83	3.24	6.47E-09	1.43E-09
CTAGE1,RBBP8	rs7243351	18:20148531	FEV1	12.31	2.25	4.69E-08	12.20	2.36	2.26E-07	6.87E-08
SOGA2	rs513953	18:8801351	FEV1	-14.50	2.58	1.96E-08	-15.07	2.78	5.91E-08	1.53E-08
PKDCC, EML4	rs963406	2:42355947	FEV1	-23.13	4.18	3.17E-08	-23.03	4.41	1.78E-07	4.19E-08
ZNF337	rs6138639	20:25669052	FEV1	17.91	2.85	3.17E-10	17.84	2.97	1.79E-09	4.97E-10
C20orf112	rs1737889	20:31042176	FEV1	-16.82	3.07	4.17E-08	-16.72	3.21	1.91E-07	5.90E-08
DNAH12	rs79294353	3:57494433	FEV1	-29.56	5.05	4.82E-09	-29.63	5.30	2.32E-08	6.90E-09
DCBLD2,MIR548G	rs6778584	3:98815640	FEV1	12.98	2.37	4.51E-08	13.02	2.47	1.39E-07	6.50E-08
OTUD4,SMAD1	rs111898810	4:146174040	FEV1	-20.24	3.61	2.14E-08	-20.20	3.80	1.08E-07	3.03E-08
DMRT2,SMARCA2	rs9407640	9:1574877	FEV1	-14.48	2.65	4.77E-08	-14.48	2.78	1.96E-07	6.59E-08
NR5A2	rs2821332	1:200085714	FVC	14.50	2.51	7.65E-09	14.33	2.62	4.74E-08	1.12E-08
C1orf140,DUSP10	rs12046746	1:221635207	FVC	-16.99	2.81	1.41E-09	-16.80	2.93	1.04E-08	2.14E-09
RYR2	1:237929787:T_TCA	1:237929787:T_TCA	FVC	-37.17	6.79	4.46E-08	-37.66	7.13	1.26E-07	3.48E-08
COMTD1,ZNF503-AS1	10:77002679:TC_T	10:77002679:TC_T	FVC	22.36	4.10	4.89E-08	22.40	4.35	2.57E-07	5.82E-08
KIRREL3-AS3,ETS1	rs73025192	11:127995904	FVC	-24.18	4.28	1.63E-08	-24.05	4.46	7.01E-08	2.27E-08
ALX1,RASSF9	rs7971039	12:85724305	FVC	16.36	2.88	1.44E-08	16.19	3.02	8.72E-08	2.06E-08
CRADD	rs11107184	12:94184082	FVC	14.89	2.71	3.87E-08	14.95	3.07	1.16E-06	5.35E-07
CCDC41	rs10859698	12:94852628	FVC	21.19	3.84	3.49E-08	18.97	4.71	5.65E-05	2.94E-05
SQRD,SEMA6D	rs4775429	15:46722435	FVC	40.23	7.21	2.45E-08	40.54	7.44	5.14E-08	2.41E-08
SMAD3	rs8025774	15:67483276	FVC	-20.87	2.92	9.34E-13	-18.72	3.37	2.77E-08	8.57E-09
PDXDC2P	rs3973397	16:70040398	FVC	-22.38	4.05	3.31E-08	-22.11	4.21	1.57E-07	4.48E-08
PMFBP1,ZFHX3	rs55771535	16:72252097	FVC	-29.88	4.83	6.38E-10	-29.95	5.06	3.17E-09	9.56E-10
MED1, CDK12	rs8067511	17:37611352	FVC	18.30	3.20	1.08E-08	18.38	3.33	3.40E-08	1.56E-08
CRHR1	rs150741403	17:43682405	FVC	35.83	5.97	1.94E-09	35.56	6.22	1.11E-08	2.83E-08
WNT3	rs199525	17:44847834	FVC	20.32	3.52	7.52E-09	20.34	3.71	4.08E-08	1.07E-08
CABLES1	rs7238093	18:20728158	FVC	18.15	3.13	6.78E-09	18.28	3.27	2.28E-08	6.87E-09
DCC	rs8089865	18:50957922	FVC	15.81	2.57	7.38E-10	15.53	2.68	7.21E-09	6.41E-10
EDAR	rs17034666	2:109571508	FVC	-27.93	4.96	1.81E-08	-27.70	5.28	1.59E-07	8.00E-08
DCBLD2,MIR548G	rs1404098	3:98806782	FVC	15.93	2.73	5.45E-09	15.78	2.85	3.07E-08	8.02E-09
AP3B1	rs72776440	5:77440196	FVC	-21.30	3.21	3.20E-11	-21.06	3.37	4.06E-10	5.26E-11
CENPW,RSPO3	rs11759026	6:126792095	FVC	-20.20	3.44	4.35E-09	-20.27	3.61	1.92E-08	6.24E-09
AGMO	rs55905169	7:15506529	FVC	-17.57	3.09	1.28E-08	-17.86	3.21	2.72E-08	7.60E-09
DMRT2,SMARCA2	rs9407640	9:1574877	FVC	-16.82	3.03	2.87E-08	-16.67	3.18	1.65E-07	3.81E-08
DCAF8	rs11591179	1:160206067	Ratio	-0.002	0.0003	3.48E-08	-0.002	0.0003	8.38E-08	1.37E-08
HTRA1	rs2293871	10:124273671	Ratio	0.002	0.0004	1.51E-08	0.002	0.0004	1.56E-08	3.92E-09

JMJD1C	rs75159994	10:64916064	Ratio	-0.003	0.0004	6.09E-09	-0.003	0.0005	1.06E-06	2.58E-07
FAM168A	11:73280955:GA_G	11:73280955:GA_G	Ratio	0.004	0.0006	2.74E-08	0.004	0.0006	4.57E-08	6.84E-09
DDHD1,MIR5580	rs4444235	14:54410919	Ratio	0.002	0.0004	4.03E-08	0.002	0.0004	1.57E-07	1.56E-08
TSHZ3	rs9636166	19:31829613	Ratio	0.003	0.0005	3.25E-09	0.003	0.0005	1.21E-08	1.73E-09
KCNJ3, NR4A2	rs72904209	2:157046432	Ratio	0.003	0.0005	3.09E-08	0.003	0.0006	1.54E-07	2.21E-08
KLHL22,MED15	rs4820216	22:20854161	Ratio	-0.003	0.0005	2.61E-10	-0.004	0.0006	1.07E-09	6.38E-11
RBMS3	rs28723417	3:29431565	Ratio	0.002	0.0004	1.77E-08	0.002	0.0004	1.18E-06	2.08E-07
DCBLD2,MIR548G	rs80217917	3:99359368	Ratio	-0.003	0.0005	2.58E-08	-0.003	0.0005	8.23E-07	2.17E-07
AFAP1	rs28520091	4:7846240	Ratio	0.002	0.0004	8.40E-09	0.002	0.0004	1.15E-08	3.38E-10
LINC00340	rs9350408	6:22021373	Ratio	-0.003	0.0003	7.45E-14	-0.003	0.0004	6.32E-13	1.53E-14
TMEM38B,ZNF462	rs2451951	9:109496630	Ratio	0.002	0.0003	2.36E-08	0.002	0.0004	1.04E-07	1.65E-08
FLJ35282,ELAVL2	rs10965947	9:23588583	Ratio	0.002	0.0004	2.70E-09	0.002	0.0004	3.52E-09	7.11E-10
Genome-wide significant loci in the Han-Eskin model that had not quite achieved genome-wide significance in the fixed-effects model										
A4GNT	rs9864090	3:137870554	Ratio	-0.002	0.0003	7.73E-08	-0.002	0.0003	3.51E-08	1.41E-08
FRMD4A	rs1418884	10:13918414	Ratio	-0.002	0.0004	1.43E-07	-0.002	0.0004	3.46E-07	2.87E-08
GSTO1	rs10883990	10:106018558	Ratio	-0.002	0.0004	6.78E-08	-0.002	0.0004	9.11E-08	1.77E-08
GSTO2	rs17826034	10:106033504	Ratio	-0.002	0.0004	1.10E-07	-0.002	0.0004	9.11E-08	1.77E-08
UBASH3B	rs4935813	11:122650172	Ratio	0.002	0.0005	1.30E-07	0.002	0.0005	1.68E-07	4.44E-08
ETFA	rs12440815	15:76627328	Ratio	0.002	0.0003	8.63E-08	0.002	0.0003	1.82E-07	4.00E-08
APP	rs2830155	21:27642567	Ratio	-0.002	0.0004	1.03E-06	-0.002	0.0004	1.97E-07	2.74E-08

[a]Methods reference: Han B, Eskin E (2011) Random-effects model aimed at discovering associations in meta-analysis of genome-wide association studies. Am J Hum Genet 88: 586-598.

[b]"Multiethnic Fixed Effects - Cohort Files" represent estimates from fixed effects meta-analysis of 35 cohort files and match estimates in Table 1; ""Multiethnic Fixed Effects - Ancestry Files" represent estimates from fixed effects meta-analysis of 4 ancestry files resulting from the ancestry-specific fixed effects meta-analyses; "Multiethnic Han Eskin Random Effects - Ancestry Files" represent estimates from Han-Eskin random effects meta-analysis of 4 cohort files resulting from the ancestry-specific fixed effects meta-analyses.

[c]Nearest gene indicates gene either harboring the variant or nearest to it

[d]FEV_1 forced expiratory volume in 1 second (in ml), FVC forced vital capacity (in ml), Ratio FEV_1/FVC (as a proportion)

[e]Additive effect of variant on pulmonary function, adjusting for age, age^2, sex, height, $height^2$, smoking status, pack-years of smoking, center (if multi-center cohort), ancestral principal components, and weight (for FVC only)

Supplementary Table 5. Top Variants from Novel Loci Discovered in the European Ancestry and Multiethnic Meta-analyses and Tested for Replication in the UK BiLEVE Study and UK Biobank Study

							CHARGE Discovery (N=90,715)			UK BiLEVE Look-Up Replication (N=48,943)		UK Biobank Look-Up Replication (N=255,492)	
Nearest Gene(s)[a]	Phenotype[b]	Population[c]	Top Variant	Chr:Pos	Coded Allele[d]	Coded Allele Freq	Beta[e]	SE	P-value	Direction of Effect Concordant with Discovery[f]	P-value[g]	Direction of Effect Concordant with Discovery[h]	P-value[i]
LOC728989	FVC	Eur	rs12724426	1:146494027	a	0.21	-36.75	6.63	2.95E-08	Yes	8.10E-01	NA	NA
DCAF8	FEV_1/FVC	ME	rs11591179	1:160206067	t	0.45	-0.002	0.0003	3.48E-08	Yes	3.69E-01	NA	NA
NR5A2	FVC	ME	rs2821332	1:200085714	a	0.47	14.5	2.51	7.65E-09	Yes	1.20E-02	Yes	4.93E-04
PIK3C2B	FEV_1	ME	rs12092943	1:204434927	t	0.74	-14.57	2.67	4.83E-08	Yes	1.94E-01	Yes	3.55E-04
CENPF,KCNK2	FVC	Eur	rs512597	1:215095003	t	0.81	-24.26	4.12	3.92E-09	Yes	9.06E-02	Yes	6.21E-02
C1orf140,DUSP10	FVC	Eur	rs6657854	1:221630555	a	0.72	-19.89	3.49	1.18E-08	Yes	1.07E-02	Yes	1.82E-03
		ME	rs12046746	1:221635207	c	0.71	-16.99	2.81	1.41E-09	Yes	1.11E-02	Yes	2.04E-03
	FEV_1	ME	1:221765779:C_CA	1:221765779	i	0.12	-36.25	6.57	3.38E-08	Yes	2.07E-01	NA	NA
RYR2	FVC	ME	1:237929787:T_TCA	1:237929787	i	0.11	-37.17	6.79	4.46E-08	Yes	2.29E-01	NA	NA
PKDCC, EML4	FEV_1	ME	rs963406	2:42355947	a	0.12	-23.13	4.18	3.17E-08	Yes	3.38E-03	Yes	1.01E-02
EDAR	FVC	ME	rs17034666	2:109571508	a	0.23	-27.93	4.96	1.81E-08	Yes	4.83E-01	Yes	5.28E-01
KCNJ3, NR4A2	FEV_1/FVC	ME	rs72904209	2:157046432	t	0.88	0.003	0.0005	3.09E-08	Yes	2.36E-02	NA	NA[i]
RBMS3	FEV_1/FVC	Eur	rs17666332	3:29469675	t	0.72	0.003	0.0005	4.76E-08	Yes	6.89E-05	NA	NA
		ME	rs28723417	3:29431565	a	0.74	0.002	0.0004	1.77E-08	Yes	3.44E-04	NA	NA
DNAH12	FEV_1	ME	rs79294353	3:57494433	a	0.92	-29.56	5.05	4.82E-09	Yes	2.94E-01	Yes	1.26E-03
DCBLD2,MIR548G	FEV_1	ME	rs6778584	3:98815640	t	0.7	12.98	2.37	4.51E-08	Yes	2.93E-04	Yes	5.40E-09
	FVC	ME	rs1404098	3:98806782	a	0.71	15.93	2.73	5.45E-09	Yes	4.66E-05	Yes	2.88E-09
	FEV_1/FVC	ME	rs80217917	3:99359368	t	0.88	-0.003	0.0005	2.58E-08	Yes	1.15E-01	NA	NA
AFAP1	FEV_1/FVC	Eur	rs28520091	4:7846240	t	0.48	0.003	0.0004	2.17E-09	Yes	2.95E-02	NA	NA
		ME	rs28520091	4:7846240	t	0.44	0.002	0.0004	8.40E-09	Yes	2.95E-02	NA	NA
OTUD4,SMAD1	FEV_1	ME	rs111898810	4:146174040	a	0.2	-20.24	3.61	2.14E-08	Yes	1.16E-02	Yes	1.33E-08
AP3B1	FEV_1	Eur	rs252746	5:77392117	a	0.78	20.05	3.45	6.19E-09	Yes	2.04E-01	NA	NA[j]
	FVC	Eur	rs12513481	5:77450828	c	0.23	-25.01	3.74	2.15E-11	Yes	3.84E-02	Yes	4.97E-20
		ME	rs72776440	5:77440196	c	0.21	-21.3	3.21	3.20E-11	Yes	3.24E-02	Yes	2.31E-20
LINC00340	FEV_1/FVC	Eur	rs1928168	6:22017738	t	0.51	0.003	0.0004	6.74E-14	Yes	7.95E-05	NA	NA
		ME	rs9350408	6:22021373	t	0.51	-0.003	0.0003	7.45E-14	Yes	1.97E-05	NA	NA
SLC25A51P1,BAI3	FEV_1/FVC	Eur	rs9351637	6:67863782	t	0.61	0.002	0.0004	2.89E-08	No	3.63E-01	NA	NA
CENPW,RSPO3	FVC	ME	rs11759026	6:126792095	a	0.72	-20.2	3.44	4.35E-09	Yes	1.51E-01	Yes	1.14E-15
AGMO	FVC	ME	rs55905169	7:15506529	c	0.31	-17.57	3.09	1.28E-08	Yes	1.70E-03	Yes	8.84E-05
CNTNAP2	FEV_1/FVC	Eur	rs1404154	7:146651409	t	0.99	-0.03	0.006	2.80E-08	NA	NA	NA	NA
DMRT2,SMARCA2	FVC	Eur	rs771924	9:1555835	a	0.42	-18.4	3.18	7.16E-09	Yes	1.75E-01	Yes	8.16E-03
		ME	rs9407640	9:1574877	c	0.42	-16.82	3.03	2.87E-08	Yes	6.87E-02	NA	NA
	FEV_1	ME	rs9407640	9:1574877	c	0.41	-14.48	2.65	4.77E-08	Yes	8.91E-02	NA	NA
FLJ35282,ELAVL2	FEV_1/FVC	ME	rs10965947	9:23588583	t	0.39	0.002	0.0004	2.70E-09	Yes	1.07E-04	NA	NA

TMEM38B,ZNF462	FEV_1/FVC	ME	rs2451951	9:109496630	t	0.47	0.002	0.0003	2.36E-08	Yes	3.72E-02	NA	NA
JMJD1C	FEV_1	ME	rs7899503	10:65087468	c	0.25	21.16	2.84	8.70E-14	Yes	1.64E-03	Yes	8.44E-02
	FEV_1/FVC	ME	rs75159994	10:64916064	t	0.77	-0.003	0.0004	6.09E-09	Yes	2.63E-02	NA	NA
COMTD1,ZNF503-AS1	FVC	ME	10:77002679:TC_T	10:77002679	d	0.22	22.36	4.1	4.89E-08	Yes	5.88E-01	NA	NA
HTRA1	FEV_1/FVC	ME	rs2293871	10:124273671	t	0.23	0.002	0.0004	1.51E-08	Yes	4.00E-02	NA	NA
FAM168A	FEV_1/FVC	ME	11:73280955:GA_G	11:73280955	d	0.2	0.004	0.0006	2.74E-08	Yes	2.32E-04	NA	NA
KIRREL3-AS3,ETS1	FVC	ME	rs73025192	11:127995904	t	0.12	-24.18	4.28	1.63E-08	Yes	2.53E-01	Yes	3.06E-01
RAB5B	FEV_1	ME	rs772920	12:56390364	c	0.72	13.86	2.49	2.48E-08	Yes	1.26E-03	Yes	2.70E-10
ALX1,RASSF9	FEV_1	Eur	rs10779158	12:85724096	a	0.34	15.89	2.9	4.36E-08	Yes	1.60E-03	Yes	6.53E-08
	FVC	Eur	rs10779158	12:85724096	a	0.34	18.72	3.31	1.52E-08	Yes	1.28E-03	Yes	6.53E-08
		ME	rs7971039	12:85724305	a	0.26	16.36	2.88	1.44E-08	Yes	4.37E-04	Yes	1.18E-07
CRADD	FVC	ME	rs11107184	12:94184082	t	0.34	14.89	2.71	3.87E-08	Yes	1.84E-01	No	3.09E-01
CCDC41	FVC	ME	rs10859698	12:94852628	a	0.21	21.19	3.84	3.49E-08	Yes	6.70E-01	No	4.60E-01
NCOR2,SCARB1	FEV_1	ME	rs11057793	12:125230287	t	0.75	17.66	3.24	4.78E-08	Yes	9.93E-02	Yes	6.82E-02
DDHD1,MIR5580	FEV_1/FVC	ME	rs4444235	14:54410919	t	0.54	0.002	0.0004	4.03E-08	Yes	4.32E-05	NA	NA
SQRDL,SEMA6D	FVC	ME	rs4775429	15:46722435	t	0.17	40.23	7.21	2.45E-08	No	3.04E-01	No	7.40E-01
SMAD3	FVC	ME	rs8025774	15:67483276	t	0.29	-20.87	2.92	9.34E-13	Yes	9.10E-03	Yes	1.93E-07
PDXDC2P	FVC	ME	rs3973397	16:70040398	a	0.48	-22.38	4.05	3.31E-08	Yes	5.90E-03	Yes	9.92E-05
PMFBP1,ZFHX3	FVC	ME	rs55771535	16:72252097	a	0.13	-29.88	4.83	6.38E-10	Yes	7.95E-01	Yes	2.92E-02
SUZ12P1	FEV_1	ME	rs62070631	17:29087285	a	0.15	20.26	3.64	2.57E-08	Yes	3.19E-04	Yes	8.67E-01
MED1, CDK12	FVC	ME	rs8067511	17:37611352	t	0.8	18.3	3.2	1.08E-08	Yes	2.12E-05	Yes	1.00E-03
LOC644172,CRHR1	FEV_1	Eur	rs143246821	17:43685698	a	0.79	30.58	4.99	9.06E-10	Yes	1.31E-12	NA	NA[j]
		ME	rs186806998	17:43682323	t	0.82	29.5	4.7	3.47E-10	Yes	3.72E-12	NA	NA[j]
	FVC	ME	rs150741403	17:43682405	c	0.85	35.83	5.97	1.94E-09	Yes	4.15E-10	NA	NA[j]
WNT3	FEV_1	Eur	rs916888	17:44863133	t	0.75	20.53	3.48	3.76E-09	Yes	5.19E-07	Yes	7.60E-22
		ME	rs199525	17:44847834	t	0.8	18.85	3.08	9.59E-10	Yes	4.69E-11	Yes	1.19E-23
	FVC	ME	rs199525	17:44847834	t	0.8	20.32	3.52	7.52E-09	Yes	3.35E-11	Yes	1.19E-23
SOGA2	FEV_1	ME	rs513953	18:8801351	a	0.29	-14.5	2.58	1.96E-08	Yes	2.23E-03	Yes	3.53E-11
CTAGE1,RBBP8	FEV_1	ME	rs7243351	18:20148531	t	0.45	12.31	2.25	4.69E-08	Yes	9.62E-02	Yes	8.53E-03
CABLES1	FVC	ME	rs7238093	18:20728158	a	0.22	18.15	3.13	6.78E-09	Yes	6.76E-02	No	2.83E-05
DCC	FVC	Eur	rs8089865	18:50957922	a	0.59	20.57	3.23	1.95E-10	Yes	3.10E-03	Yes	7.08E-08
		ME	rs8089865	18:50957922	a	0.53	15.81	2.57	7.38E-10	Yes	3.10E-03	Yes	7.08E-08
TSHZ3	FEV_1/FVC	Eur	rs1353531	19:31846907	t	0.14	-0.003	0.0006	4.53E-08	Yes	2.69E-05	NA	NA
		ME	rs9636166	19:31829613	a	0.86	0.003	0.0005	3.25E-09	Yes	1.38E-06	NA	NA
ZNF337	FEV_1	ME	rs6138639	20:25669052	c	0.79	17.91	2.85	3.17E-10	Yes	1.54E-04	Yes	1.62E-05
C20orf112	FEV_1	ME	rs1737889	20:31042176	t	0.22	-16.82	3.07	4.17E-08	Yes	1.69E-03	Yes	1.18E-06
EYA2	FVC	Eur	rs2236519	20:45529571	a	0.38	-18.06	3.28	3.51E-08	Yes	1.15E-04	Yes	7.15E-08
KLHL22,MED15	FEV_1/FVC	Eur	rs4820216	22:20854161	t	0.15	-0.004	0.0006	1.53E-09	Yes	2.22E-04	NA	NA
		ME	rs4820216	22:20854161	t	0.13	-0.003	0.0005	2.61E-10	Yes	2.22E-04	NA	NA

[a]Nearest gene indicates gene either harboring the variant or nearest to it.
[b]Phenotypes: FEV_1 forced expiratory volume in 1 second (in ml), FVC forced vital capacity (in ml), Ratio FEV_1/FVC (as a proportion)
[c]Ancestral/ethnic populations: Eur European, ME Multiethnic
[d]Alleles for INDELS: I Insertion, D Deletion
[e]Additive effect of variant on pulmonary function, adjusting for age, age^2, sex, height, $height^2$, smoking status, pack-years of smoking, weight (for FVC only), and center, ancestral principal components, and a random familial effect to account for family relatedness when appropriate.
[f]UK BiLEVE estimates are on a different scale (inverse normal transformation) therefore we report only whether the direction of effect is concordant between discovery and replication studies. Adjustment included age, age2, sex, height, pack-years of smoking, and ancestral principal components.
[g]Based on a two-sided $p<9.6x10^{-4}$, 15 loci replicated for the same trait based on the lead variant from our analysis: *DCBLD2/MIR548G, SUZ12P1, CRHR1, WNT3, ZNF337, ALX1/RASSF9, MED1/CDK12, EYA2, RBMS3, LINC00340, FLJ35282/ELAVL2, DDHD1/MIR5580, TSHZ3, KLHL22/MED15, FAM168A*. Based on a one-sided p-value, an additional 4 loci replicated for the same trait based on the lead variant: *RAB5B, JMJD1C, AGMO,* and *C20orf112*. One other loci replicated for the same trait based on a different significant variant in the locus (*DCC,* rs8085778, replication $p=2.80x10^{-4}$, R^2 with lead variant=0.9) and another loci replicated for a different pulmonary function trait based on the lead variant (*PDXDC2P* discovered for FVC replicated for FEV_1, replication $p=3.99x10^{-4}$).
[h]UK Biobank estimates are on a different scale (inverse normal transformation) therefore we report only whether the direction of effect is concordant between discovery and replication studies. Adjustment included sex and ancestral principal components
[i]Among lead variants that did not replicate for the same trait in UK BiLEVE, 9 loci replicated for the same trait with a two-sided p<9.6x10-4 based on the lead variant in UK Biobank: *NR5A2, PIK3C2B, OTUD4/SMAD1, AP3B1, CENPW/RSPO3, SMAD3, PDXDC2P, SOGA2, DCC*. Based on a one-sided p<9.6x10-4, 1 additional locus replicated for the same trait based on the lead variant from our analysis: *DNAH12*. One other locus replicated for a different pulmonary function trait based on the lead variant (*KCNJ3/NR4A2* discovered for FEV_1/FVC replicated for FEV_1 and FVC, replication $p=2.06x10^{-12}$ and $p=3.55x10^{-7}$).
[j]Proxy values: *AP3B1* FEV_1 rs72776502 $p=7.67x10^{-12}$; *LOC644172/CRHR1* FEV_1 rs117368197 $p=2.36x10^{-27}$; *LOC644172/CRHR1* FVC rs1724390 $p=8.56x10^{-30}$

Supplementary Table 6. Top Variants from Novel Loci Discovered in the African Ancestry and Hispanic Ethnicity Meta-analyses and Tested for Replication in Various Datasets

African Ancestry

					CHARGE Discovery (N=8,429)					COPDGene Replication (N=3,219)					SAPPHIRE (N=1,707)					SAGE (N=1,405)			
Nearest Gene(s)[a]	Phenotype[b]	Top Variant	Chr:Pos	Coded Allele	Coded Allele Freq	Beta[c]	SE	P-value	Imputation Score (ARIC)	Coded Allele Freq	Beta[d]	SE	P-value	Imputation Score	Coded Allele Freq	Beta[e]	SE	P-value	Imputation Score	Coded Allele Freq	Direction of Effect Concordant with Discovery[f]	P-value	Imputation Score
RYR2	FEV_1	rs3766889	1:237941781	t	0.82	52.21	9.52	4.12E-08	0.96	0.81	46.85	19.96	0.03	0.97	0.81	22.00	22.00	0.32	0.94	0.82	no	0.50	0.97
C2orf48, HPCAL1	FEV_1/FVC	rs139215025	2:10418806	a	0.01	-0.07	0.01	9.03E-11	0.60	0.01	-0.007	0.01	0.66	0.72	0.01	0.01	0.02	0.40	0.59	0.01	yes	0.60	0.75
EN1, MARCO	FVC	rs114962105	2:119660943	a	0.98	178.48	32.44	3.77E-08	0.85	0.98	-42.54	67.31	0.53	0.88	0.99	-65.00	77.00	0.40	1.00	0.99	yes	0.88	0.89
CADPS	FEV_1/FVC	rs111793843	3:62386350	t	0.01	-0.05	0.008	1.97E-08	0.50	0.004	0.05	0.03	0.12	0.56	0.01	-0.003	0.02	0.85	0.62	0.004	yes	0.13	0.75
ANKRD55,MAP3K1	FEV_1	rs11748173	5:55922145	t	0.21	67.07	10.72	3.91E-10	0.90	NA	NA	NA	NA	NA	0.12	-16.00	26.00	0.54	0.92	0.14	no	0.83	0.92
HDC	FEV_1/FVC	rs180930492	15:50555681	t	0.01	-0.07	0.01	2.59E-09	0.76	0.01	-0.003	0.02	0.90	0.83	0.01	0.01	0.02	0.63	0.88	0.004	yes	0.41	0.88
LOC283867,CDH5	FEV_1/FVC	rs144296676	16:66060569	t	0.99	-0.03	0.006	5.35E-09	0.93	0.98	0.01	0.01	0.31	0.95	0.99	0.01	0.01	0.51	0.91	0.99	no	0.001	0.90
CPT1C	FEV_1/FVC	rs147472287	19:50213396	t	0.01	-0.05	0.009	3.25E-08	0.49	0.01	-0.005	0.01	0.72	0.85	0.01	0.02	0.01	0.11	0.74	0.02	yes	0.65	0.83

Hispanic Ethnicity

					CHARGE Discovery (N=11,775)					MESA (N=806)					GALA II (N=2,203)				
					Coded Allele Freq	Beta[c]	SE	P-value	Imputation Score (HCHS/SOL)	Coded Allele Freq	Beta[c]	SE	P-value	Imputation Score	Coded Allele Freq	Beta[g]	SE	P-value	Imputation Score
DKFZp686O1327, PABPC1P2	FVC	rs6746679	2:147046592	a	0.56	-37.36	6.67	2.17E-08	0.99	0.58	14.95	24.44	0.54	0.98	0.56	-13.82	14.83	0.35	0.98

[a]Nearest gene indicates gene either harboring the variant or nearest to it.

[b]Phenotypes: FEV_1 forced expiratory volume in 1 second (in ml), FVC forced vital capacity (in ml), Ratio FEV_1/FVC (as a proportion)

[c]Adjusted for age, age^2, sex, height, $height^2$, smoking status, pack-years of smoking, ancestral principal components, and weight (for FVC only)

[d]Adjusted for age, age^2, sex, height, $height^2$, smoking status, pack-years of smoking, and principal components of ancestry

[e]Adjusted for age, age^2, sex, height, $height^2$, smoking status, packyears of smoking, ancestral principal components, and weight (for FVC). Also adjusted for asthma status since population included 1200 asthma patients and 2507 healthy controls.

[f]SAGE analyzed percent predicted FEV_1, FVC and FEV_1/FVC, therefore we report only whether the direction of effect is concordant between discovery and replication studies. Adjusted for age, sex, height, asthma status, and BMI category. Population included individuals aged 7-41.

[g]Adjusted for sex, age, ethnicity (Mexican or Puerto Rican), height, global ancestry for AFR, and global ancestry for NAM.

Supplementary Table 7. Lead Variants from 52 Novel Loci Discovered for Pulmonary Function in the CHARGE Consortium European and Multiethnic Meta-analyses and Look-Up of Association with COPD in the ICGC Consortium[a]

				CHARGE Pulmonary Function Discovery						ICGC COPD Lookup		
Nearest Gene[b]	rsID	Chr	Position	Allele1	Freq1	Population_Phenotype[c]	Effect[d]	StdErr	P-value	Effect	StdErr	P-value[e]
LOC728989	rs12724426	1	146494027	a	0.21	European_FVC	-36.75	6.63	2.95E-08	NA	NA	NA
DCAF8	rs11591179	1	160206067	t	0.45	Multiethnic_Ratio	-0.002	0.0003	3.48E-08	0.063	0.022	0.003
NR5A2	rs2821332	1	200085714	a	0.47	Multiethnic_FVC	14.50	2.51	7.65E-09	0.001	0.02	0.96
PIK3C2B	rs12092943	1	204434927	t	0.74	Multiethnic_FEV1	-14.57	2.67	4.83E-08	0.039	0.025	0.11
CENPF,KCNK2	rs512597	1	215095003	t	0.81	European_FVC	-24.26	4.12	3.92E-09	-0.023	0.025	0.35
C1orf140,DUSP10	rs6657854	1	221630555	a	0.72	European_FVC	-19.89	3.49	1.18E-08	0.019	0.023	0.42
C1orf140,DUSP10	rs12046746	1	221635207	c	0.71	Multiethnic_FVC	-16.99	2.81	1.41E-09	0.017	0.023	0.45
C1orf140,DUSP10	1:221765779:C_CA	1	221765779	i	0.12	Multiethnic_FEV1	-36.25	6.57	3.38E-08	0.116	0.05	0.02
RYR2	1:237929787:T_TCA	1	237929787	i	0.11	Multiethnic_FVC	-37.17	6.79	4.46E-08	0.011	0.042	0.79
PKDCC,EML4	rs963406	2	42355947	a	0.12	Multiethnic_FEV1	-23.13	4.18	3.17E-08	0.018	0.036	0.61
EDAR	rs17034666	2	109571508	a	0.23	Multiethnic_FVC	-27.93	4.96	1.81E-08	-0.013	0.046	0.78
KCNJ3,NR4A2	rs72904209	2	157046432	t	0.88	Multiethnic_Ratio	0.003	0.0005	3.09E-08	-0.079	0.033	0.02
RBMS3	rs17666332	3	29469675	t	0.72	European_Ratio	0.003	0.0005	4.76E-08	-0.089	0.023	1.42E-04
RBMS3	rs28723417	3	29431565	a	0.74	Multiethnic_Ratio	0.002	0.0004	1.77E-08	-0.082	0.023	2.85E-04
DNAH12	rs79294353	3	57494433	a	0.92	Multiethnic_FEV1	-29.56	5.05	4.82E-09	0.082	0.047	0.08
DCBLD2,MIR548G	rs6778584	3	98815640	t	0.7	Multiethnic_FEV1	12.98	2.37	4.51E-08	-0.012	0.022	0.58
DCBLD2,MIR548G	rs1404098	3	98806782	a	0.71	Multiethnic_FVC	15.93	2.73	5.45E-09	-0.006	0.022	0.8
MIR548G	rs80217917	3	99359368	t	0.88	Multiethnic_Ratio	-0.003	0.0005	2.58E-08	0.031	0.032	0.33
AFAP1	rs28520091	4	7846240	t	0.48	European_Ratio	0.003	0.0004	2.17E-09	-0.032	0.023	0.16
OTUD4,SMAD1	rs111898810	4	146174040	a	0.2	Multiethnic_FEV1	-20.24	3.61	2.14E-08	0.115	0.031	1.61E-04
AP3B1	rs252746	5	77392117	a	0.78	European_FEV1	20.05	3.45	6.19E-09	-0.017	0.025	0.49
AP3B1	rs12513481	5	77450828	c	0.23	European_FVC	-25.01	3.74	2.15E-11	0.011	0.024	0.66
AP3B1	rs72776440	5	77440196	c	0.21	Multiethnic_FVC	-21.30	3.21	3.20E-11	0.02	0.024	0.41
LINC00340	rs1928168	6	22017738	t	0.51	European_Ratio	0.003	0.0004	6.74E-14	-0.066	0.02	0.001
LINC00340	rs9350408	6	22021373	t	0.54	European_Ratio	-0.003	0.0004	9.77E-12	0.045	0.02	0.02
SLC25A51P1,BAI3	rs9351637	6	67863782	t	0.61	European_Ratio	0.002	0.0004	2.89E-08	-0.0002	0.021	0.99
CENPW,RSPO3	rs11759026	6	126792095	a	0.72	Multiethnic_FVC	-20.20	3.44	4.35E-09	0.032	0.027	0.23
AGMO	rs55905169	7	15506529	c	0.31	Multiethnic_FVC	-17.57	3.09	1.28E-08	-0.013	0.027	0.62
CNTNAP2	rs1404154	7	146651409	t	0.99	European_Ratio	-0.03	0.006	2.80E-08	NA	NA	NA
DMRT2,SMARCA2	rs771924	9	1555835	a	0.42	European_FVC	-18.40	3.18	7.16E-09	0.002	0.02	0.91
DMRT2,SMARCA2	rs9407640	9	1574877	c	0.41	Multiethnic_FEV1	-14.48	2.65	4.77E-08	0.033	0.023	0.15
FLJ35282,ELAVL2	rs10965947	9	23588583	t	0.39	Multiethnic_Ratio	0.002	0.0004	2.70E-09	-0.067	0.023	0.004
TMEM38B,ZNF462	rs2451951	9	109496630	t	0.47	Multiethnic_Ratio	0.002	0.0003	2.36E-08	-0.071	0.021	5.99E-04
JMJD1C	rs7899503	10	65087468	c	0.25	Multiethnic_FEV1	21.16	2.84	8.70E-14	-0.041	0.03	0.16
NRBF2,JMJD1C	rs75159994	10	64916064	t	0.77	Multiethnic_Ratio	-0.003	0.0004	6.09E-09	0.044	0.028	0.11

COMTD1,ZNF503-AS1	10:77002679:TC_T	10	77002679	d	0.22	Multiethnic_FVC	22.36	4.1	4.89E-08	-0.01	0.026	0.69
HTRA1	rs2293871	10	124273671	t	0.23	Multiethnic_Ratio	0.002	0.0004	1.51E-08	-0.037	0.026	0.16
FAM168A	11:73280955:GA_G	11	73280955	d	0.2	Multiethnic_Ratio	0.004	0.001	2.74E-08	-0.032	0.031	0.3
KIRREL3-AS3,ETS1	rs73025192	11	127995904	t	0.12	Multiethnic_FVC	-24.18	4.28	1.63E-08	0.01	0.035	0.77
RAB5B	rs772920	12	56390364	c	0.72	Multiethnic_FEV1	13.86	2.49	2.48E-08	-0.039	0.022	0.09
ALX1,RASSF9	rs10779158	12	85724096	a	0.34	European_FEV1	15.89	2.9	4.36E-08	-0.045	0.021	0.04
ALX1,RASSF9	rs7971039	12	85724305	a	0.26	Multiethnic_FVC	16.36	2.88	1.44E-08	-0.035	0.023	0.12
CRADD	rs11107184	12	94184082	t	0.34	Multiethnic_FVC	14.89	2.71	3.87E-08	-0.048	0.022	0.03
CCDC41	rs10859698	12	94852628	a	0.21	Multiethnic_FVC	21.19	3.84	3.49E-08	-0.002	0.033	0.95
NCOR2,SCARB1	rs11057793	12	125230287	t	0.75	Multiethnic_FEV1	17.66	3.24	4.78E-08	-0.104	0.03	4.41E-04
DDHD1,MIR5580	rs4444235	14	54410919	t	0.54	Multiethnic_Ratio	0.002	0.0004	4.03E-08	-0.049	0.019	0.01
SQRDL,SEMA6D	rs4775429	15	46722435	t	0.17	Multiethnic_FVC	40.23	7.21	2.45E-08	NA	NA	NA
SMAD3	rs8025774	15	67483276	t	0.29	Multiethnic_FVC	-20.87	2.92	9.34E-13	-0.017	0.024	0.46
PDXDC2P	rs3973397	16	70040398	a	0.48	Multiethnic_FVC	-22.38	4.05	3.31E-08	NA	NA	NA
PMFBP1,ZFHX3	rs55771535	16	72252097	a	0.13	Multiethnic_FVC	-29.88	4.83	6.38E-10	-0.023	0.036	0.52
SUZ12P1	rs62070631	17	29087285	a	0.15	Multiethnic_FEV1	20.26	3.64	2.57E-08	-0.126	0.032	7.88E-05
MED1,CDK12	rs8067511	17	37611352	t	0.8	Multiethnic_FVC	18.30	3.20	1.08E-08	-0.011	0.026	0.69
LOC644172,CRHR1[f]	rs143246821	17	43685698	a	0.79	European_FEV1	30.58	4.99	9.06E-10	NA	NA	NA
LOC644172,CRHR1	rs186806998	17	43682323	t	0.82	Multiethnic_FEV1	29.50	4.7	3.47E-10	NA	NA	NA
LOC644172,CRHR1	rs150741403	17	43682405	c	0.85	Multiethnic_FVC	35.83	5.97	1.94E-09	NA	NA	NA
WNT3	rs916888	17	44863133	t	0.75	European_FEV1	20.53	3.48	3.76E-09	-0.082	0.024	6.03E-04
WNT3	rs199525	17	44847834	t	0.80	Multiethnic_FEV1	18.85	3.08	9.59E-10	-0.081	0.024	8.77E-04
SOGA2	rs513953	18	8801351	a	0.29	Multiethnic_FEV1	-14.50	2.58	1.96E-08	0.107	0.023	2.81E-06
CTAGE1,RBBP8	rs7243351	18	20148531	t	0.45	Multiethnic_FEV1	12.31	2.25	4.69E-08	-0.029	0.021	0.16
CABLES1	rs7238093	18	20728158	a	0.22	Multiethnic_FVC	18.15	3.13	6.78E-09	-0.052	0.026	0.04
DCC	rs8089865	18	50957922	a	0.59	European_FVC	20.57	3.23	1.95E-10	-0.037	0.021	0.07
TSHZ3,THEG5	rs1353531	19	31846907	t	0.14	European_Ratio	-0.003	0.0006	4.53E-08	0.035	0.029	0.22
TSHZ3	rs9636166	19	31829613	a	0.86	Multiethnic_Ratio	0.003	0.0005	3.25E-09	-0.048	0.031	0.12
ZNF337	rs6138639	20	25669052	c	0.79	Multiethnic_FEV1	17.91	2.85	3.17E-10	-0.064	0.027	0.02
C20orf112	rs1737889	20	31042176	t	0.22	Multiethnic_FEV1	-16.82	3.07	4.17E-08	0.126	0.028	6.52E-06
EYA2	rs2236519	20	45529571	a	0.38	European_FVC	-18.06	3.28	3.51E-08	0.002	0.022	0.93
KLHL22,MED15	rs4820216	22	20854161	t	0.15	European_Ratio	-0.004	0.0006	1.53E-09	0.069	0.029	0.02

[a]ICGC Consortium reference: Hobbs BD, de Jong K, Lamontagne M, Bosse Y, Shrine N, et al. (2017) Genetic loci associated with chronic obstructive pulmonary disease overlap with loci for lung function and pulmonary fibrosis. Nat Genet 49: 426-432.

[b]Nearest gene indicates gene either harboring the variant or nearest to it

[c]FEV_1 forced expiratory volume in 1 second (in ml), FVC forced vital capacity (in ml), Ratio FEV_1/FVC (as a proportion)

[d]Additive effect of variant on pulmonary function, adjusting for age, age^2, sex, height, $height^2$, smoking status, pack-years of smoking, center (if multi-center cohort), ancestral principal components, and weight (for FVC only)

[e]10 lead variants representing 8 novel loci were associated with COPD at p<9.6x10-4: *RBMS3, OTUD4/SMAD1, TMEM38B/ZNF462, NCOR2/SCARB1, SUZ12P1, WNT3, SOGA2, C20orf112*

[f]Proxy variant in CRHR1 (rs114860868) had p-value=0.002 for COPD in ICGC Consortium

Supplementary Table 8. Lead Variants from 60 Novel Loci Discovered for Pulmonary Function in the CHARGE Consortium and Looked-Up for eQTLs in 5 Datasets (Lung eQTL Consortium, GTEx, BIOS, Westra, FHS). "Yes" indicates eQTL associations meeting FDR significance within specified dataset[a]

			Lung eQTL Consortium[c]		GTEx	BIOS[d]			Westra[e]		FHS	
Nearest Gene(s)[b]	Chr:Pos	rsID	cis eQTL	trans eQTL	cis eQTL	cis eQTL	cis mQTL	trans mQTL	cis eQTL	trans eQTL	cis eQTL	cis mQTL
LOC728989	1:146494027	rs12724426	no	no	no	yes	no	no	no	no	yes	yes
DCAF8	1:160206067	rs11591179	yes	no	no	yes	no	no	no	no	yes	yes
NR5A2	1:200085714	rs2821332	no	no	no	no	no	no	no	no	no	yes
PIK3C2B	1:204434927	rs12092943	yes	no	no	no	no	no	yes	no	no	yes
CENPF,KCNK2	1:215095003	rs512597	no	no	no	no	no	no	no	no	no	yes
C1orf140,DUSP10	1:221630555	rs6657854	no	no	no	no	no	no	no	no	no	yes
C1orf140,DUSP10	1:221635207	rs12046746	no	no	no	no	no	no	no	no	no	yes
C1orf140,DUSP10	1:221765779:C_CA	1:221765779:C_CA	no	no	no	no	no	no	no	no	no	no
RYR2	1:237929787:T_TCA	1:237929787:T_TCA	no	no	no	no	no	no	no	no	no	no
PKDCC, EML4	2:42355947	rs963406	no	no	no	no	no	no	no	no	no	yes
EDAR	2:109571508	rs17034666	no	no	no	no	no	no	no	no	yes	yes
KCNJ3, NR4A2	2:157046432	rs72904209	no	no	no	yes	no	no	no	no	yes	yes
RBMS3	3:29469675	rs17666332	no	no	no	no	no	no	no	no	no	yes
RBMS3	3:29431565	rs28723417	no	no	no	no	no	no	no	no	no	yes
DNAH12	3:57494433	rs79294353	no	no	no	no	no	no	no	no	no	yes
DCBLD2,MIR548G	3:98815640	rs6778584	no	no	no	no	no	no	no	no	no	yes
DCBLD2,MIR548G	3:98806782	rs1404098	no	no	no	no	no	no	no	no	no	yes
MIR548G	3:99359368	rs80217917	no	no	no	no	no	no	no	no	no	yes
AFAP1	4:7846240	rs28520091	no	no	no	yes	no	no	no	no	yes	yes
OTUD4,SMAD1	4:146174040	rs111898810	no	no	no	no	no	no	no	no	no	yes
AP3B1	5:77392117	rs252746	no	no	no	yes	no	no	no	no	no	yes
AP3B1	5:77450828	rs12513481	no	no	no	yes	no	no	no	no	no	yes
AP3B1	5:77440196	rs72776440	no	no	no	yes	no	no	no	no	no	yes
LINC00340	6:22017738	rs1928168	no	no	no	no	no	no	no	no	no	yes
LINC00340	6:22021373	rs9350408	no	no	no	no	no	no	no	no	no	yes
SLC25A51P1,BAI3	6:67863782	rs9351637	no	no	no	no	no	no	no	no	no	yes
CENPW,RSPO3	6:126792095	rs11759026	no	no	no	no	no	no	no	no	no	yes
AGMO	7:15506529	rs55905169	no	no	no	no	no	no	no	no	no	no
CNTNAP2	7:146651409	rs1404154	no	no	no	no	no	no	no	no	no	no
DMRT2,SMARCA2	9:1555835	rs771924	no	no	no	no	no	no	no	no	no	no
DMRT2,SMARCA2	9:1574877	rs9407640	no	no	no	no	no	no	no	no	no	no
FLJ35282,ELAVL2	9:23588583	rs10965947	no	no	no	no	no	no	no	no	no	no
TMEM38B,ZNF462	9:109496630	rs2451951	no	yes	no	no	no	no	no	no	no	yes
JMJD1C	10:65087468	rs7899503	no	no	no	yes	no	no	no	no	no	yes
NRBF2,JMJD1C	10:64916064	rs75159994	no	no	no	yes	no	no	no	no	no	no

COMTD1,ZNF503-AS1	10:77002679:TC_T	10:77002679:TC_T	yes	no	no	no	no	no	no	no	no	no
HTRA1	10:124273671	rs2293871	no	no	no	no	no	no	no	no	no	yes
FAM168A	11:73280955:GA_G	11:73280955:GA_G	yes	no	no	no	no	no	no	no	no	no
KIRREL3-AS3,ETS1	11:127995904	rs73025192	no	no	no	no	no	no	no	no	no	no
RAB5B	12:56390364	rs772920	yes	yes	yes	yes	no	no	no	no	yes	yes
ALX1,RASSF9	12:85724096	rs10779158	no	no	no	no	no	no	no	no	no	yes
ALX1,RASSF9	12:85724305	rs7971039	no	no	no	no	no	no	no	no	no	yes
CRADD	12:94184082	rs11107184	no	no	no	yes	no	no	yes	no	yes	yes
CCDC41	12:94852628	rs10859698	no	no	no	no	no	no	yes	no	no	yes
NCOR2,SCARB1	12:125230287	rs11057793	no	no	no	no	no	no	no	no	no	yes
DDHD1,MIR5580	14:54410919	rs4444235	no	no	no	yes	no	yes	no	no	no	yes
SQRD,SEMA6D	15:46722435	rs4775429	no	no	no	no	no	no	no	no	no	no
SMAD3	15:67483276	rs8025774	yes	no	yes	yes	no	no	yes	no	yes	yes
PDXDC2P	16:70040398	rs3973397	yes	no	yes	yes	no	no	no	no	no	no
PMFBP1,ZFHX3	16:72252097	rs55771535	no	no	no	yes	no	no	no	no	yes	yes
SUZ12P1	17:29087285	rs62070631	yes	no	no	yes	no	no	no	no	yes	yes
MED1, CDK12	17:37611352	rs8067511	yes	no	no	yes	no	no	no	no	yes	yes
LOC644172,CRHR1	17:43685698	rs143246821	yes	yes	yes	no	no	no	no	no	no	no
LOC644172,CRHR1	17:43682323	rs186806998	yes	yes	yes	no	no	no	no	no	no	no
LOC644172,CRHR1	17:43682405	rs150741403	yes	yes	yes	yes	no	no	no	no	yes	yes
WNT3	17:44863133	rs916888	yes	yes	yes	yes	no	no	yes	no	no	no
WNT3	17:44847834	rs199525	yes	yes	yes	yes	yes	no	no	no	yes	yes
SOGA2	18:8801351	rs513953	yes	no	yes	no	no	no	no	no	no	yes
CTAGE1,RBBP8	18:20148531	rs7243351	no	no	no	no	no	no	no	no	no	yes
CABLES1	18:20728158	rs7238093	no	no	no	no	yes	no	no	no	no	yes
DCC	18:50957922	rs8089865	no	no	no	no	no	no	no	no	no	no
TSHZ3, THEG5	19:31846907	rs1353531	no	no	no	yes	no	no	yes	no	yes	yes
TSHZ3	19:31829613	rs9636166	no	no	no	yes	no	no	no	no	yes	yes
ZNF337	20:25669052	rs6138639	yes	no	no	yes	no	no	yes	no	yes	yes
C20orf112	20:31042176	rs1737889	yes	no	no	yes	no	no	no	no	yes	yes
EYA2	20:45529571	rs2236519	no	no	no	no	no	no	no	no	no	yes
KLHL22,MED15	22:20854161	rs4820216	no	no	no	yes	no	no	yes	no	no	yes
RYR2	1:237941781	rs3766889	no	no	no	no	no	no	no	no	no	no
C2orf48, HPCAL1	2:10418806	rs139215025	no	no	no	no	no	no	no	no	no	no
EN1, MARCO	2:119660943	rs114962105	no	no	no	no	no	no	no	no	no	no
CADPS	3:62386350	rs111793843	no	no	no	no	no	no	no	no	no	no
ANKRD55,MAP3K1	5:55922145	rs11748173	no	no	no	yes	no	no	no	no	no	yes
HDC	15:50555681	rs180930492	no	no	no	no	no	no	no	no	no	no
LOC283867,CDH5	16:66060569	rs144296676	no	no	no	no	no	no	no	no	no	no
CPT1C	19:50213396	rs147472287	no	no	no	no	no	no	no	no	no	no
DKFZp686O1327, PABPC1P2	2:147046592	rs6746679	no	no	no	no	no	no	no	no	no	yes

[a]The Lung eQTL Consortium study used a 10% FDR cut-off, while all other studies used a 5% FDR cut-off
[b] Nearest gene indicates gene either harboring the variant or nearest to it
[c]Lung eQTL Consortium includes studies from Laval University, the University of Groningen and the University of British Columbia
[d]BIOS includes studies from CODAM, LLD, LLS, RS
[e]Westra includes studies from EGCUT, InCHIANTI, Rotterdam Study, Fehrmann, HVH, SHIP-TREND and DILGOM

Supplementary Table 9. Lead Variants from 8 Novel Loci Discovered for Pulmonary Function in the CHARGE Consortium and Looked-Up for eQTLs in 2 Datasets (SAPPHIRE and MESA) among Individuals of African Ancestry. "Yes" indicates eQTL associations meeting FDR significance (<0.05) within specified dataset

			SAPPHIRE						MESA
			Asthma Cases		Non-Asthma Controls		Combined Cases & Controls		
**Nearest Gene(s)**	**Chr:Pos**	**rsID**	**cis eQTL**	**trans eQTL**	**cis eQTL**	**trans eQTL**	**cis eQTL**	**trans eQTL**	**cis eQTL**
RYR2	1:237941781	rs3766889	no	no	no	no	no	no	no
C2orf48, HPCAL1	2:10418806	rs139215025	no	yes	no	no	no	no	NA
EN1, MARCO	2:119660943	rs114962105	no	no	no	no	no	no	NA
CADPS	3:62386350	rs111793843	no	yes	no	no	no	no	NA
ANKRD55,MAP3K1	5:55922145	rs11748173	no	no	no	no	no	no	no
HDC	15:50555681	rs180930492	no	no	no	yes	no	no	NA
LOC283867,CDH5	16:66060569	rs144296676	no	no	no	no	no	no	no
CPT1C	19:50213396	rs147472287	no	no	no	no	no	no	NA

Supplementary Table 10. Genetic correlation between our pulmonary function (FEV_1, FVC and FEV_1/FVC) results and publicly available GWAS of ever smoking, height and asthma based on LD score regression.

	FEV_1			FVC			FEV_1/FVC		
	$\rho_{genetic}$	SE	P-value	$\rho_{genetic}$	SE	P-value	$\rho_{genetic}$	SE	P-value
**FEV_1**	-	-	-	0.81	0.01	<0.0001	0.53	0.03	<0.0001
**FVC**	0.81	0.01	<0.0001	-	-	-	-0.06	0.05	0.23
**FEV_1/FVC**	0.53	0.03	<0.0001	-0.06	0.05	0.23	-	-	-
**Smoking[a]**	-0.10	0.06	0.07	-0.06	0.06	0.28	-0.07	0.06	0.23
**Height[b]**	-0.03	0.03	0.35	-0.04	0.03	0.20	0.02	0.03	0.41
**Asthma[c]**	0.51	0.10	<0.0001	0.41	0.10	<0.0001	0.27	0.10	0.003

SE standard error

[a]Tobacco, Genetics C (2010) Genome-wide meta-analyses identify multiple loci associated with smoking behavior. Nat Genet 42: 441-447.

[b]Wood AR, Esko T, Yang J, Vedantam S, Pers TH, et al. (2014) Defining the role of common variation in the genomic and biological architecture of adult human height. Nat Genet 46: 1173-1186

[c]Moffatt MF, Gut IG, Demenais F, Strachan DP, Bouzigon E, Heath S, von Mutius E, Farrall M, Lathrop M, Cookson WOCM, GABRIEL Consortium. (2010). A large-scale, consortium-based genomewide association study of asthma. N Engl J Med 363(13):1211-1221.

Supplementary Table 11. Missense and synonymous variants within genome-wide significant signals using Ensemble Variant Effect Predictor

Chromosomal location	Allele change	Gene	Transcript	Consequence on protein sequence	Amino acid change	Codon change	CADD scaled C-score	Lead SNV
chr5:77412011	A>G	AP3B1	NM_001271769.1	synonymous	A	gcT/gcC	10.5	chr5:77450828
chr5:77473165	A>G	AP3B1	NM_001271769.1	splice region, synonymous	N	aaT/aaC	13.1	chr5:77450828
chr6:28891176	T>C	TRIM27	NM_006510.4	synonymous	V	gtA/gtG	4.3	chr6:26199903
chr6:26199903	T>C	HIST1H2BF	NM_003522.3	synonymous	S	tcT/tcC	11.6	chr6:26377939
chr6:26368279	A>G	-	ENSESTT00000078123	synonymous	R	agA/agG	0.0	chr6:26377939
chr6:26370572	T>C	BTN3A2	NM_001197246.2	synonymous	V	gtT/gtC	10.2	chr6:26377939
chr6:26370657[a,b]	A>G	BTN3A2	NM_001197246.2	missense	N/D	Aac/Gac	0.001	chr6:26377939
chr6:26370659	T>C	BTN3A2	NM_001197246.2	synonymous	N	aaT/aaC	7.1	chr6:26377939
chr6:26373150	T>C	BTN3A2	NM_001197246.2	synonymous	P	ccT/ccC	9.4	chr6:26377939
chr6:26373507	A>G	BTN3A2	NM_001197246.2	missense	N/S	aAc/aGc	11.5	chr6:26377939
chr6:26377028	A>G	LOC101928771	XM_005249511.1	missense	M/V	Atg/Gtg	0.7	chr6:26377939
chr6:26377385	C>G	LOC101928771	XM_005249511.1	missense	L/V	Ctg/Gtg	2.7	chr6:26385093
chr6:26392515	C>G	BTN2A2	NM_001197240.1	missense	P/A	Cct/Gct	0.7	chr6:26407482
chr6:26409890	C>G	BTN3A1	NM_001145008.1	missense	T/R	aCa/aGa	0.003	chr6:26463271
chr6:26463574	T>G	BTN2A1	NM_001197233.2	missense	L/W	tTg/tGg	24.4	chr6:26463271
chr6:26463575	T>G	BTN2A1	NM_001197233.2	missense	C/W	tgT/tgG	24.6	chr6:26463271
chr6:26463660	A>G	BTN2A1	NM_001197233.2	missense	M/V	Atg/Gtg	23.6	chr6:26463271
chr6:26468326	A>G	BTN2A1	NM_001197233.2	missense	Q/R	cAg/cGg	7.5	chr6:26463271
chr6:26468545	C>G	BTN2A1	NM_001197233.2	missense	A/G	gCc/gGc	6.7	chr6:26511076
chr6:26509382	T>C	BTN1A1	NM_001732.2	missense	S/P	Tcc/Ccc	0.03	chr6:27278020
chr6:27278020	A>C	POM121L2	NM_033482.3	missense	C/G	Tgt/Ggt	24.3	chr6:27774824
chr6:27775674[a,b]	A>G	HIST1H2BL	NM_003519.3	missense	L/P	cTg/cCg	2.7	chr6:28885865
chr6:29342775	A>G	OR12D3	NM_030959.2	missense	I/T	aTc/aCc	1.6	chr6:29340743
chr6:29523957	A>G	UBD	NM_006398.3	synonymous	Y	taT/taC	3.1	chr6:29523957
chr6:30032522	A>C	-	ENSESTT00000015858	missense	E/A	gAa/gCa	7.4	chr6:30032522

chr6:30166266	T>C	TRIM26	NM_001242783.1	synonymous	R	agA/agG	16.4	chr6:30173538
chr6:30515043	T>C	GNL1	NM_005275.3	synonymous	A	gcA/gcG	3.1	chr6:30508956
chr6:30652781	T>C	PPP1R18	NM_001134870.1	missense	R/G	Agg/Ggg	32.0	chr6:30650026
chr6:30782205	C>G	-	ENSESTT00000017163	missense	R/P	cGc/cCc	2.5	chr6:30763562
chr6:30893941	A>G	VARS2	NM_001167733.1	missense	Q/R	cAg/cGg	7.0	chr6:30894965
chr6:30899524[a,c]	T>C	SFTA2	NM_205854.2	missense	N/S	aAt/aGt	12.9	chr6:30899195
chr6:30919391	A>G	DPCR1	NM_080870.3	synonymous	S	tcA/tcG	8.3	chr6:30913458
chr6:30920124	A>G	DPCR1	NM_080870.3	missense	K/E	Aag/Gag	1.3	chr6:30913458
chr6:30951561	T>C	-	ENSESTT00000080349	synonymous	P	ccT/ccC	0.5	chr6:30951561
chr6:30993313	T>C	MUC22	NM_001198815.1	synonymous	S	tcT/tcC	10.0	chr6:30977680
chr6:30996593[d]	A>T	MUC22	XM_005248779.1	missense	T/S	Act/Tct	0.001	chr6:30977680
chr10:64916064	T>G	-	ENSESTT00000055432	missense	F/L	ttT/ttG	0.1	chr10:64892052, chr10:65087468
chr10:64945364	A>G	JMJD1C	NM_004241.2	synonymous	D	gaT/gaC	8.8	chr10:65087468
chr10:75558867	A>G	ZSWIM8	NM_001242487.1	synonymous	L	ctA/ctG	6.4	chr10:75545251
chr15:67528374[a,c]	T>G	AAGAB	NM_024666.4	missense	I/L	Atc/Ctc	23.9	chr15:67521810
chr17:43545893	T>C	PLEKHM1	NM_014798.2	synonymous	T	acA/acG	0.1	chr17:43545893
chr17:43552717	C>G	PLEKHM1	NM_014798.2	synonymous	S	tcG/tcC	1.0	chr17:43545893
chr18:50936994	T>C	DCC	NM_005215.3	synonymous	P	ccT/ccC	0.3	chr18:50957922
chr18:50942653	T>C	DCC	ENST00000412726	missense	M/T	aTg/aCg	16.7	chr18:50957922
chr20:25259006	T>G	PYGB	NM_002862.3	missense	S/A	Tcc/Gcc	26.5	chr20:25271033
chr20:25262769	A>G	PYGB	NM_002862.3	missense	N/D	Aat/Gat	21.7	chr20:25271033
chr20:25715507	T>G	-	ENSESTT00000071551	missense	C/G	Tgt/Ggt	0.6	chr20:25669052, chr20:25721248

[a]SIFT category of 'tolerated'. The amino acid substitution is predicted damaging is the score is ≤0.05, and tolerated if the score is >0.05.
[b]PolyPhen-2 category of 'benign'. Mutations with estimated false positive rates above the second (higher) FPR value are classified as 'benign'.
[c]PolyPhen-2 category of 'possibly damaging'. Mutations with the posterior probabilities associated with false positive rates at or below the second (higher) FPR value are predicted to be 'possibly damaging'.
[d]PolyPhen-2 category of 'unknown'. If the lack of data does not allow to make a prediction, then the outcome is reported as 'unknown'.

Supplementary Table 12. Tissue/cell type enrichment results for FEV_1 and FEV_1/FVC in European ancestry using DEPICT[a] (FDR<0.05)

MeSH term	Name	MeSH first level term	MeSH second level term
Trait: FEV_1			
A10.690.467	Muscle Smooth	Tissues	Muscles
Trait: FEV_1/FVC			
A03.556.875.875	Stomach	Digestive System	Gastrointestinal Tract
A03.556.875	Upper Gastrointestinal Tract	Digestive System	Gastrointestinal Tract
A10.690.467	Muscle Smooth	Tissues	Muscles
A11.620	Muscle Cells	Cells	Muscle Cells
A11.620.520	Myocytes Smooth Muscle	Cells	Muscle Cells
A11.872.190.260	Embryoid Bodies	Cells	Stem Cells
A06.407	Endocrine Glands	Endocrine System	Endocrine Glands
A05.360.319	Genitalia Female	Urogenital System	Genitalia
A07.231.114	Arteries	Cardiovascular System	Blood Vessels
A07.541	Heart	Cardiovascular System	Heart
A06.407.071	Adrenal Glands	Endocrine System	Endocrine Glands
A11.329.171	Chondrocytes	Cells	Connective Tissue Cells
A06.407.071.140	Adrenal Cortex	Endocrine System	Endocrine Glands
A05.360.319.114.630	Ovary	Urogenital System	Genitalia
A05.360.319.114	Adnexa Uteri	Urogenital System	Genitalia
A05.360.319.114.373	Fallopian Tubes	Urogenital System	Genitalia
A05.360.319.679	Uterus	Urogenital System	Genitalia
A06.407.312	Gonads	Endocrine System	Endocrine Glands
A07.541.560	Heart Ventricles	Cardiovascular System	Heart
A05.360.319.679.690	Myometrium	Urogenital System	Genitalia
A05.360	Genitalia	Urogenital System	Genitalia
A03.556.875.500	Esophagus	Digestive System	Gastrointestinal Tract
A11.329.114	Adipocytes	Cells	Connective Tissue Cells
A10.615.789	Serous Membrane	Tissues	Membranes
A05.810.890	Urinary Bladder	Urogenital System	Urinary Tract

[a]Methods reference: Pers, T. H., J. M. Karjalainen, et al. (2015). "Biological interpretation of genome-wide association studies using predicted
gene functions." Nat Commun 6: 5890

Supplementary Table 13. Enriched networks in genes related to FEV_1, FVC, or FEV_1/FVC in European ancestry meta-analysis results using standard Ingenuity Pathway Analysis (IPA) and including the major histocompatibility complex (MHC)

Top Diseases and Functions	Score[a]	#Focus Molecules[b]	Molecules in Network
Endocrine System Disorders, Gastrointestinal Disease, Immunological Disease	57	35	ABT1,APP,ATAT1,C1orf87,CALML4,CAVIN4,CFDP1,CHRAC1,CLUAP1,CXXC4,CYB5B,DPCR1,DZIP1L,FBXL2,FCF1,GPANK1,HIST1H2BM,IQCH,KCNIP4,KLHL32,LMNTD1,MND1,NTM,PRR16,STARD10,TBCK,TCF19,TCP11,THSD4,TMCC2,TRIML1,ZKSCAN8,ZNF165,ZNF322,ZSCAN16
Cellular Assembly and Organization, DNA Replication, Recombination, and Repair, Endocrine System Disorders	51	33	ABHD16A,ADAM19,ATAD5,BAG6,BAIAP2L1,BRD2,C6orf136,CCDC88A,COPS2,CSNK2B,DCAF8,DMRT2,EGLN,F11R,FKBPL,GANAB,GLIS3,HIST1H2AB,HIST1H3A,HIST1H4A,HMGN4,KLHL22,MFAP2,MLF1,NDFIP2,PCDH1,PCGF6,PRRC2A,RNF5,SH3PXD2A,SLC38A2,SRC (family),VKORC1,VSX1,WWP2
Cancer, Dermatological Diseases and Conditions, Organismal Injury and Abnormalities	48	32	ADGRL2,AGAP1,AP-3,AP3B1,AP3D1,BLVRA,CAMK2G,CCSER2,CD7,CNTN2,CNTNAP2,CRADD,DCXR,DLGAP1,DMWD,DYNLL1,EML3,EML4,EPB41L3,ERBIN,FBXO38,Lymphotoxin,NFkB (complex),NFKBIL1,PCDH9,PNPT1,RELT,SECTM1,SLC17A1,SLMAP,SOX30,SYNE1,TMEM132B,TRIM15,TRIM40
RNA Post-Transcriptional Modification, DNA Replication, Recombination, and Repair, Cell Death and Survival	39	28	Alpha tubulin,ATPase,Beta Tubulin,CDC5L,CLINT1,DDX39B,EHMT2,FNBP1,FSD2,Gsk3,HP1,HSPA1A/HSPA1B,JAZF1,KANSL1,KAT8,LRRC45,MACF1,MDC1,Mi2,MTA2,MTA3,NSF,PFDN1,R3HDM1,RUVBL1,SEC24C,Syntaxin,THRAP3,TRAF3IP1,TUBB,USB1,WRN,WWOX,ZGPAT,ZSCAN26
Inflammatory Disease, Neurological Disease, Skeletal and Muscular Disorders	37	27	ARHGAP23,BCKDK,BTN3A1,BTN3A2,BTN3A3,CHSY3,CNPY3,Cr3,DCBLD2,DPH5,EMP2,EPHX4,FRMD4A,GNL1,HLA-DOB,HLA-DQ,HLA-DQA2,HLA-DQB1,HLA-DQB2,HLA-DR group,HLA-DRA,Hla-Drb,HLA-DRB1,HLA-E,HLA-G,Ifnar,METTL21C,MHC II-β,Mhc2 Alpha,MICAL3,MIR124,PEBP4,RPAP1,SPSB1,TNF
Cardiovascular System Development and Function, Organismal Development, Tissue Development	37	27	caspase,CCHCR1,DGKB,DLC1,DNMT1,DSP,Focal adhesion kinase,GOT,Hdac,HDAC4,HDAC9,HIST1H3C,HIST1H4J,Histone h4,HSP,IL6R,MEF2C,mir-302,MSH5,NEBL,NINL,NOTCH4,Pias,PIAS1,REV3L,RPL10A,TNP1,TRIM27,TRIM31,TSHZ3,TTC28,UBE2,UBE2H,VGLL4,ZNF76
Post-Translational Modification, Protein Synthesis, Protein	37	27	26s Proteasome,ABCF1,ACTR2,AFF3,ATXN2L,Calbindin,CCNT2,CENPW,CHEK2,CSNK1D,

Trafficking			FLOT1,FSH,GATA6,HIST1H3B,HIST1H3I,HIST1H4B,Hsp70,Ige,MAP7D1,MED15,NANP,PHOX2B,POU5F1,PRKN,RNA polymerase II,SH3GL3,SMAD3,Smad2/3,SP1,TEFM,TET2,Ubiquitin,USP20,VAPA,ZNF74
Cardiovascular Disease, Developmental Disorder, Hematological Disease	33	25	AIF1,AK4,ATF6B,Cg,CISD3,COX7A2L,CRLF3,CYFIP2,Cytochrome bc1,cytochrome-c oxidase,DOCK7,EDN2,EFEMP1,endothelin receptor,ETFA,FANCC,GLS,glutathione peroxidase,GPX5,GPX6,HOXB1,Lh,Mitochondrial complex 1,NDUFA12,NUAK1,Pka,PPP1R18,RAC3,RASSF10,Rnr,RPS10,RPS26,RSRC1,Sod,UQCR10
Cellular Movement, Developmental Disorder, Hereditary Disorder	29	23	AGPAT1,APOM,ARHGEF17,BTN1A1,CFB,Cpla2,Ferritin,FOXP1,HDL,HDL-cholesterol,hemoglobin,HIST1H2BL,Ikb,IL23,IREB2,JMJD7-PLA2G4B,KLF6,LDL,LIPG,LY75,MKI67,MUC21,MUC22,Mucin,NID2,Nr1h,NR2F2,NR5A2,PKDCC,SAA,SCARF2,SCUBE3,SPINK7,TRIM10,Vegf
Endocrine System Disorders, Gastrointestinal Disease, Immunological Disease	27	22	20s proteasome,ASPSCR1,C/ebp,CD6,ERK1/2,HERC3,HFE,HLA Class I,HLA-A,Hla-abc,HLA-B,HLA-C,IFN alpha/beta,IL27,IL17RD,Immunoproteasome Pa28/20s,KIR,LTA,MAP1LC3,MAT1A,MAT2B,MHC Class I (complex),MHC CLASS I (family),MHC I-α,NEU1,NFE2L1,NTN4,PSMA4,PSMB8,PSMB9,RARB,SEMA6A,Tap,TAP2,ZBTB12
Cardiovascular Disease, Neurological Disease, Organismal Injury and Abnormalities	27	22	AChR,Akt,ARHGEF3,ATF6,BTN2A1,BTN2A2,C1q,CAPZA3,CD83,CDON,CHRNA3,CHRNA5,CHRNB4,CORO6,CYP19,DENND6A,FAM168A,GPSM3,GYPA,Iga,IgG1,Igg3,IgG2a,IgG2b,Igm,INTS5,INTS12,KLHL42,LY86,MHC II,MTCL1,nicotinic acetylcholine receptor,NRG (family),RAB3GAP1,SPPL2A
Cellular Development, Connective Tissue Development and Function, Skeletal and Muscular System Development and Function	27	22	Alp,ALPP,BMP,BMP2,BMP4,BMP6,BMP2/4,BMP8A,CLIC1,Collagen type II,ERK,FUT11,GDF5,Gli,GTPase,Hedgehog,HHIP,LHX3,LMX1B,NOG,NPNT,Patched,PTCH1,RIMS2,SHH,Smad,SMAD1/5,Smad1/5/8,SP7,ST6GALNAC3,STMN3,TBX1,TMSB4,UBP1,ZNF184
Molecular Transport, RNA Trafficking, Cell Cycle	27	22	Actin,ANXA1,CYP1A1,EEF1G,ENTPD6,estrogen receptor,F Actin,HISTONE,Histone h3,HLA-L,HLX,IL12 (complex),Immunoglobulin,Interferon alpha,LCT,LSM2,LST1,MAPT,MHC Class II (complex),NAB1,NKAPL,nucleoside-triphosphatase,PYGB,SCGN,SETMAR,snRNP,SNRNP48,SNRPF,SRRM1,STH,TFAP2D,Tnf (family),trypsin,ZNF239,ZRANB3
Cellular Development, Cellular Growth and Proliferation, Hereditary Disorder	25	21	AIMP1,ANAPC10,APC (complex),Cdc2,CDC7,Cdk,CDK6,CENPF,chemokine receptor,COMTD1,Cyclin A,Cyclin E,DARS,farnesyl transferase,HIST1H1A,HIST1H1B,HIST1H1C,HIST1H1E,HIST1H1T,Histone

			H1,JUN/JUNB/JUND,KIF3B,LOX,Mapk,MCM6,P-TEFb,PPT2,Rab5,Rb,RIN3,RPA,SCAMP4,SHB,SPTLC2,SYMPK
Endocrine System Development and Function, Lipid Metabolism, Small Molecule Biochemistry	22	19	AAR2,ADAP2,Adaptor protein 1,Caspase 3/7,CK1,Coup-Tf,Ctbp,Cyclin D,CYP21A2,DGAT1,E2f,Hat,HDAC7,HIST1H2BD,HIST1H4C,histone deacetylase,histone-lysine N-methyltransferase,IKZF4,Jnk,KAT6B,MAP3K,MECOM,MICA,MICB,PCCA,RBBP8,TCF,thymidine kinase,TNXB,TRIM38,VANGL2,VLDL,Wnt,WNT3,WNT4
Organismal Injury and Abnormalities, Cell-To-Cell Signaling and Interaction, Nervous System Development and Function	22	19	ADCY,AFAP1,AHNAK,AMBP,ATP13A2,Camk,CDK4/6,CEP72,CHRM3,chymotrypsin,CLASP2,CNR1,CRHR1,G protein,G protein beta gamma,GABBR1,Gi-coupled receptor,GRM4,Gs-coupled receptor,HTR4,Kcnj,KCNJ2,KCNJ3,KCNK3,L-type Calcium Channel,Ldh (complex),LOR,P2RY6,Pkc(s),Plc beta,Ryr,TSH,TUFM,voltage-gated calcium channel,YLPM1

[a]Derived from a p value in the Ingenuity Pathway Analysis software. The score of 20 means a 1 in 1000 chance of having the Focus Molecules together in a network at random.

[b]Molecules are related to genes to which our association results ($P<1x10^{-5}$) mapped among genes from each network from the Ingenuity Pathway Analysis (Ingenuity Systems, Redwood City, CA, USA, http://www.ingenuity.com/)

Supplementary Table 14. Enriched networks in genes related to FEV_1, FVC, or FEV_1/FVC in multi-ethnic meta-analysis results using standard Ingenuity Pathway Analysis (IPA) and including the major histocompatibility complex (MHC)

Top Diseases and Functions	Score[a]	#Focus Molecules[b]	Molecules in Network
Cellular Assembly and Organization, Tissue Development, Connective Tissue Disorders	47	33	ABT1,ADAP2,APP,ATAT1,C9orf72,CAVIN4,CFAP58,CFDP1,CYB5B,DPCR1,DZIP1L,FBXL2,FBXL20,FBXO38,GPANK1,HARS,HIST1H2BD,HIST1H2BM,HM13,IQCH,KCNIP4,LMNTD1,NWASP,PRR16,SOX30,SPIC,TBCK,TCF19,TCP11,THSD4,TMEM266,TTLL6,Wave,ZNF322,ZSCAN16
Embryonic Development, Nervous System Development and Function, Organ Development	45	32	ABCF1,ANK1,AURK,BAIAP2L1,DLC1,EGFL8,Gq-coupled receptor,GSTO2,ITGA1,KIAA1107,KLHL22,LIME1,Mapk,MEF2A,METTL15,MUM1,OBSCN,PCGF6,PGBD1,PLPBP,ROBO1,SCAF8,SCARF2,SPATA18,SRGAP3,TEFM,WWC1,WWC2,ZBED9,ZKSCAN1,ZKSCAN3,ZKSCAN4,ZKSCAN8,ZNF165,ZSCAN21
Cell-To-Cell Signaling and Interaction, Cellular Function and Maintenance, Inflammatory Response	36	28	ARHGAP23,ARL5B,BCKDHB,BTN3A1,BTN3A2,BTN3A3,BTNL2,CEACAM,CEACAM3,CEACAM6,DCBLD2,DPH5,DUPD1,EPHX4,FERMT1,FRMD4A,GATS,GNL1,Gpd,GPD2,H2-a,HLA-DQ,HLA-DR group,HLA-DRA,lipoprotein lipase,LRRC8D,NLRC3,PEBP4,peptidase,SCUBE2,SPNS1,SPSB1,TNF,TNN,ZDHHC8
Cardiovascular Disease, Developmental Disorder, Hematological Disease	36	28	ATP13A2,CFB,COPA,FAM13A,Fanc,FANCC,FANCE,GTF2H4,HIST1H2AC,Holo RNA polymerase II,I kappa b kinase,IK,Ikb,Insulin,LINC00475,LOR,MAP3K14,NELFE,NFIX,PEA15,RFX6,RNA polymerase II,Rnr,RPL10A,RPS10,RPS26,RTEL1-TNFRSF6B,SCN3B,SLX4,TFAP2B,VTRNA1-1,VTRNA1-2,VTRNA1-3,ZNF74,ZSCAN12
Cell-To-Cell Signaling and Interaction, Nervous System Development and Function, Amino Acid Metabolism	34	27	Akt,BTN2A1,BTN2A2,CDON,CNTN5,CNTNAP4,CYP19,DENND6A,DNER,FAM168A,GPSM3,INTS5,INTS12,KCNT2,KLHL42,LRP,LRP1B,LRPAP1,MTCL1,NCSTN,NLGN1,Notch,POFUT1,Presenilin,RAB3GAP1,RNF135,Secretase gamma,SEMA6D,SLC17A,SLC17A1,SLC17A2,SLC17A6,SLC1A3,UTP23,VLDL
Cardiac Arrythmia, Cardiovascular Disease, Hereditary Disorder	34	27	ADGRL2,ARHGEF17,BCAS3,BTN1A1,C2orf48,CABP2,Camk,CAMK1D,CAMK2G,CNGB1,CNKSR3,DDAH2,DDX11,DIP2B,FAT1,Kcnj,KCNJ2,KCNJ3,L-type Calcium Channel,LARP4,LY75,Pde,Pde4,PDE4D,PDE5A,Pka catalytic subunit,PKDCC,Ryr,RYR2,SCGN,SCUBE3,SEMA3A,SLMAP,TTC19,Vegf
Metabolic Disease, Developmental Disorder, Hereditary Disorder	34	27	AHNAK,AIF1,BLVRA,CDK4/6,CISD3,CLUAP1,COX4I2,COX7A2L,Cytochrome bc1,cytochrome-c oxidase,endothelin receptor,EPOP,ERBB3,ETFA,FKBPL,GALK2,glutathione peroxidase,GPX5,GPX6,Gsk3,HDAC7,HERC1,JUN/JUNB/JUND,MACF1,MICA,Mitochondrial complex

			1,MLF1,MTIF2,NDUFA2,NDUFA12,RCN2,TRAF3IP1,TUFM,UQCR10,ZSWIM7
Cancer, Cell-To-Cell Signaling and Interaction, Organismal Injury and Abnormalities	34	27	ATF6B,C2,CYP,DDX1,GLS,HIST1H2AB,HIST1H4J,HLA-L,HLX,HTRA1,IGFBP3,IGFBP5,Interferon alpha,Laminin1,LSM2,MSH5,NCR2,p70 S6k,PAPPA,PNPT1,PREP,PRSS16,PRSS36,PTEN,RAD51B,Serine Protease,snRNP,SNRNP48,SNRPF,Tgf beta,TMPRSS9,TMPRSS15,TSH,WWP2,ZMAT5
Cancer, Gastrointestinal Disease, Organismal Injury and Abnormalities	34	27	ATXN2L,C8orf34,CCHCR1,COPS2,Ctbp,DCAF8,DOT1L,EHMT2,EPC1,FLOT1,GINS1,HIST1H2BB,HIST1H4A,histone-lysine N-methyltransferase,HP1,Ige,MCM7,MECOM,MED15,mediator,MICAL3,mir-365,MTA2,NINL,p85 (pik3r),PRDM16,RASSF8,Smad2/3,TH1 Cytokine,TRIM27,TRIM31,TRIM32,TRPS1,ZGPAT,ZRANB3
Connective Tissue Disorders, Dermatological Diseases and Conditions, Organ Morphology	32	26	ADCY9,CARMIL1,Cg,CNPY4,CRIPT,DUSP16,EFEMP1,ELAVL2,Erm,EYA2,FSH,HIST1H1C,HIST1H1T,HIST1H2AG,ID4,KLHDC4,Lh,LOX,MMP15,MTMR3,NUPR1,PABPC4,PI3K p85,PPT2,PTPase,PTPRG,Sod,SRC (family),STAU2,TRA2A,TRIB1,TULP1,TYRO3,tyrosine kinase,UBASH3B
Endocrine System Disorders, Gastrointestinal Disease, Immunological Disease	32	26	ABHD16A,ADCY,ADRB,APOM,ASCC2,ASPSCR1,BAG6,BOK,CACNA1S,Calmodulin,CENPW,DNAJB4,DNAJC1,DNAJC11,DND1,FAF1,Hdac,HSP,Hsp70,Hsp22/Hsp40/Hsp90,HSPA1L,METTL21C,NCR3,Nos,PRKG1,Proinsulin,REEP3,RNF5,RPAP1,TBX3,TRIM39,TRIM39-RPP21,UBR3,UBXN1,ZNF311
Auditory Disease, Developmental Disorder, Endocrine System Disorders	30	25	20s proteasome,ABCB5,ADAM19,ANKRD13B,ANKS1A,ATPase,BNIP3,C/ebp,CALCOCO2,DGKG,EGLN,FSD2,HARS2,HERC3,JAK1/2,KANSL1,MAP1LC3,MHC CLASS I (family),NDFIP2,NFE2L1,NRBF2,P glycoprotein,PPP1R18,PSMA4,PSMB9,Ras,RSPO2,SDHB,TAT,UBD,UBE4B,Ubiquitin,ZMAT2,ZNF337,ZSCAN26
Developmental Disorder, Hereditary Disorder, Metabolic Disease	30	25	AP3B1,AP3D1,Arf,AZGP1,BRD2,C6orf136,calpain,CDK5RAP3,CLASP2,CNTN2,Collagen Alpha1,CSNK2B,CYB5R4,cyclooxygenase,EPB41L3,EVI5,Focal adhesion kinase,LYPLAL1,MUC21,MUC22,Mucin,Muscarinic cholinergic receptor,NUF2,OTUD4,PIP4K2A,PLA2,PLA2R1,Pld,RASSF10,Spectrin,SQOR,TRIM10,TRIM44,WDR26,ZIC1
Cell Death and Survival, Cellular Compromise, Cellular Growth and Proliferation	28	24	CD8,CLEC18A/CLEC18C,CRADD,DCXR,EDAR,HFE,HIVEP3,HLA Class I,HLA-A,HLA-B,HLA-C,HLA-DQA2,HLA-DQB2,HLA-E,HLA-G,Ifnar,ITPRIP,KIR,Mac,MHC,MHC Class I (complex),MHC I-α,MHC II-β,MLKL,NFkB (complex),NFKBIL1,PYDC2,RELT,ST3GAL1,STK40,Tap,TAP2,TRIM40,ZBTB12,ZFAND6
Connective Tissue Disorders,	26	23	Alp,BIN3,BMP,BMP2,BMP4,BMP6,BMP2/4,BMP8A,CLIC1,Collagen type

Organismal Injury and Abnormalities, Skeletal and Muscular Disorders			II,DCAF16,ERK,GDF5,Gli,growth factor receptor,JMJD1C,KLF10,LMX1B,mir-302,NOG,PLCL1,PYGB,SGF29,Smad,SMAD1/5,Smad1/5/8,SMOOTH MUSCLE ACTIN,SP7,SPRED2,SUFU,TMSB4,TSHZ3,UBP1,ZNF3,ZNF184
Cancer, Gastrointestinal Disease, Organismal Injury and Abnormalities	24	22	AFAP1,AFF1,AFF3,ANAPC10,APC (complex),CCNT2,Cdc2,CDC7,Cdk,CDK12,CENPF,CXXC4,Cyclin A,Cyclin B,E2f,ELAVL4,farnesyl transferase,Fascin,FCF1,GYPC,HIST1H1B,HIST1H1D,Histone H1,Importin beta,LHFPL6,P-TEFb,Pkc(s),PPP1R13L,Rab5,RAB5B,RIN3,SAP130,SUDS3,TET2,TPX2
Cellular Development, Connective Tissue Development and Function, Tissue Development	24	22	7S NGF,ADAMTS2,CK1,Collagen type I,Collagen type III,Collagen type IV,Cyclin D,CYP21A2,FBXO31,FGF18,FRAS1,FUT11,GLIS3,Hedgehog,HHIP,ISL2,JAG1,KDM4C,KLF5,LHX3,MEF2,MFAP2,NOTCH4,NPNT,P38 MAPK,Patched,PRRX2,PTCH1,SFRP2,SHH,TCF,tubulin (family),Wnt,WNT3,WNT4
Antigen Presentation, Cell-mediated Immune Response, Cellular Assembly and Organization	24	22	ARAP1,ARHGAP27,atypical protein kinase C,BEGAIN,CDC42,CYFIP2,DEF6,DLGAP1,DOCK2,DOK7,Ephb,F11R,Fibrin,Fibrinogen,GIT1,Glycogen synthase,Homer,HOMER2,HOXB1,Integrin alpha 5 beta 1,LTK,MARK3,NCK2,Pak,PDGFB,Phosphatidylinositol4,5 kinase,Pka,Ptk,RAC3,Rap,RSRC1,sPla2,TMEM132B,VARS2,YLPM1
Cellular Assembly and Organization, Organismal Survival, Cell Death and Survival	24	22	ABCA4,AGER,ASXL1,BCL2L1,C11orf74,caspase,CASQ1,CCAR2,collagen,CSNK1D,CYP1A1,cytochrome C,DDX39B,estrogen receptor,ETS1,GOT,Hsp27,Hsp90,HSPA1A/HSPA1B,Ifn gamma,MAPT,MDC1,MDM4,mGluR,Mmp,NRP1,PARP,PCDH18,SMAD3,STH,TGFBR2,Tnf (family),trypsin,WDR35,WWOX
Post-Translational Modification, Protein Synthesis, Protein Trafficking	23	21	26s Proteasome,AGO2,ARID3B,Brd7,C6orf48,CERS6,Chtop,Ck2,Fendrr,Fendrr,HIST1H2BK,HIST1H3I,HIST1H4D,HIST1H4H,HIST1H4K,HIST1H4L,HISTONE,Histone h3,Histone h4,Hst1,Huwe1,IL12 (complex),IPO8,JAZF1,LOC100910107,MEF2C,MICB,mir-548,NEWGENE_2116,NFATC2IP,NKAPL,POU5F1,RUVBL1,TRIM71,VGLL4
Drug Metabolism, Molecular Transport, Small Molecule Biochemistry	21	20	adenosine triphosphate,ANKRD33B,ARMC2,BCL6,CCDC91,CDH23,COQ5,CSMD1,CTAGE1,DENND2A,DLG4,EEFSEC,ENTPD6,GPC6,GRIA,GTPase,HORMAD2,HSF1,HYKK,Inflammasome (NALP1, ASC, CASP1, CASP5),MPIG6B,MSRB3,NXF1,OGFOD3,PANX3,PES1,Prrt1,PYCARD,PYURF,RIPPLY2,RNF2,SHISA9,STAG3,TRIM26,TRIP6

[a]Derived from a p value in the Ingenuity Pathway Analysis software. The score of 20 means a 1 in 1000 chance of having the Focus Molecules together in a network at random.
[b]Molecules are related to genes to which our association results ($P<1x10^{-5}$) mapped among genes from each network from the Ingenuity Pathway Analysis (Ingenuity Systems, Redwood City, CA, USA, http://www.ingenuity.com/)

Supplementary Table 15. Trans-ethnic association results having high posterior probabilities for causality (posterior probabilities with annotations >0.8) using PAINTOR[a]

Trait[b]	rsID	Chromosomal position	Gene	Association (Z score)				Posterior probability		Functional annotations
				European	African	East Asian	Hispanic/Latino	Without annotations	With annotations	
FEV1	rs916888*	chr17:44,863,133	WNT3	-5.89	-0.65	NA	-1.07	1.00	1.00	
FEV1	rs140877773	chr12:56,365,267	CDK2 (RAB5B)	-2.58	NA	NA	-3.06	0.23	0.97	Strong transcription (fetal lung), Genic enhancers (NHLF)
FEV1	rs118127236	chr10:75,480,558	BMS1P4	3.57	NA	NA	1.87	0.13	0.96	Strong transcription (fetal lung), Genic enhancers (NHLF)
FVC	rs114962105*	chr2:119,660,943	EN1, MARCO	NA	-5.5	NA	NA	1.00	1.00	
FVC	rs8043852	chr16:72,248,132	PMFBP1,ZFHX3	-0.67	-3.64	NA	-3.11	0.95	0.95	
FVC	rs55771535*	chr16:72,252,097	PMFBP1,ZFHX3	-4.46	-3.72	NA	-2.2	0.92	0.92	
Ratio	rs273389	chr9:23,587,811	FLJ35282,ELAVL2	4	1.42	NA	2.63	1.00	1.00	
Ratio	rs139215025*	chr2:10,418,806	C2orf48,HPCAL1	NA	-6.47	NA	NA	1.00	1.00	H4K8ac (fetal lung, lung carcinoma), H4K8ac (NHLF), DNase hotspot (fetal lung)
Ratio	rs147472287*	chr19:50,213,396	CPT1C	NA	-5.53	NA	NA	1.00	1.00	
Ratio	rs111793843*	chr3:62,386,350	CADPS	NA	-5.59	NA	NA	1.00	1.00	
Ratio	rs144296676*	chr16:66,060,569	LOC283867,CDH5	NA	5.86	NA	NA	1.00	1.00	
Ratio	rs180930492*	chr15:50,555,681	HDC	NA	-5.97	NA	NA	1.00	1.00	
Ratio	rs142842953	chr15:50,646,275	HDC	NA	-4.65	NA	NA	0.47	0.99	H2A.Z (fetal lung, lung carcinoma), H4K8ac (NHLF), DNase hotspot (fetal lung)
Ratio	rs1192415*	chr1:92,077,097	CDC7,TGFBR3	5.80	2.53	3.69	2.89	0.80	0.98	H2A.Z (A549 lung carcinoma), DNase hotspot (fetal lung)
Ratio	rs3755597	chr3:99,356,614	COL8A1 (DCBLD2)	4.38	2.21	1.43	1.91	0.16	0.91	H2A.Z (fetal lung, lung carcinoma), H4K8ac (NHLF), DNase hotspot (fetal lung)

[a]Methods refernce: Kichaev G, Pasaniuc B (2015) Leveraging Functional-Annotation Data in Trans-ethnic Fine-Mapping Studies. Am J Hum Genet 97: 260-271.
[b]FEV1, forced expiratory volume in 1 second; FVC, forced vital capacity; Ratio, FEV1/FVC;
NA=not available; NHLF=Normal Human Lung Fibroblasts
*=the top SNPs from the fixed-effects meta-analyses

Supplementary Table 16. Posterior probabilities of causality (>0.6) for SNPs in the region of known and novel lead SNPs using FINEMAP[a]

Phenotype	GWAS Lead SNP	Chr	Pos	Closest Gene lead SNP	Independent signal	Chr	Pos	Posterior probability[b]	Closest Gene to independent signal
FEV_1	rs10516526	4	106688904	GSTCD	rs34712979	4	106819053	0.8825	NPNT
	rs11172113	12	57527283	LRP1	rs507562	12	57849768	0.8677	INHBE
	**rs1928168**[*]	6	22017738	LINC00340	rs9393231	6	22123695	0.7174	LINC00340
	rs34712979	4	106819053	NPNT	rs34712979	4	106819053	0.8167	NPNT
FVC	rs10858246	9	139102831	QSOX2	rs56969554	9	138960042	0.7809	NACC2
					rs62583535	9	138990435	0.736	NACC2,C9orf69
	rs12149828	16	10706328	EMP2,TEKT5	rs9939578	16	10903108	0.999	TVP23A
					rs4780999	16	10907363	0.999	TVP23A
					rs9745621	16	10896769	0.7363	TVP23A
	rs1344555	3	169300219	MECOM	rs76089058	3	169269161	0.7562	MECOM
	rs138641402	4	145445779	GYPA,HHIP-AS1	rs17767210	4	145330792	0.7885	GYPA,HHIP-AS1
	rs1595029	3	158241767	RSRC1	rs1656369	3	158280085	0.999	RSRC1,MLF1
FEV_1/FVC	rs10498230	2	229502503	SPHKAP,PID1	rs191121583	2	229517352	0.777	SPHKAP,PID1
	rs10515750	5	156810072	CYFIP2	rs11740562	5	156942285	0.9999	ADAM19
					rs6899205	5	156943285	0.6241	ADAM19
	rs10516526	4	106688904	GSTCD	rs34712979	4	106819053	0.9967	NPNT
					rs2193856	4	106742958	0.9904	GSTCD
	rs113745635	12	95554771	FGD6	rs11107915	12	95549025	0.9338	FGD6
					rs11613607	12	95529427	0.6295	FGD6
	rs11658500	17	36886828	CISD3	rs11654269	17	36888147	0.6678	CISD3
	rs12149828	16	10706328	EMP2,TEKT5	rs2719651	16	10750047	0.7334	TEKT5
	rs12447804	16	58075282	MMP15	rs11648508	16	58063513	0.8818	MMP15
	rs1294421	6	6743149	LY86,RREB1	rs11759956	6	7109097	0.7339	RREB1
	rs148274477	6	142838173	GPR126,LOC153910	rs262119	6	142842793	0.999	GPR126,LOC153910
					rs11155242	6	142691549	0.9864	GPR126
					rs12200161	6	142636740	0.9148	GPR126
					rs9399401	6	142668901	0.8301	GPR126
	rs1529672	3	25520582	RARB	rs73051993	3	25685589	0.8679	TOP2B
					rs2037860	3	25681432	0.8673	TOP2B
	rs1990950	5	156920756	ADAM19	rs11740562	5	156942285	0.9998	ADAM19
					rs6899205	5	156943285	0.6241	ADAM19
	rs2811415	3	127991527	EEFSEC	rs2999045	3	127865397	0.708	RUVBL1,EEFSEC
	rs34712979	4	106819053	NPNT	rs34712979	4	106819053	0.9965	NPNT
					rs2193856	4	106742958	0.9818	GSTCD
	rs7715901	5	147856392	HTR4	rs58746316	5	147837672	0.942	HTR4
	**rs9351637**[*]	6	67863782	SLC25A51P1,BAI3	rs2374021	6	67854972	0.9984	SLC25A51P1,BAI3
	rs993925	1	218860068	TGFB2,LOC643723	rs17048367	1	218833890	0.9999	TGFB2,LOC643723
					rs74143099	1	218815428	0.7854	TGFB2,LOC643723
					rs7527236	1	218846832	0.6758	TGFB2,LOC643723

[a]Methods reference: Benner C, Spencer CC, Havulinna AS, Salomaa V, Ripatti S, et al. (2016) FINEMAP: efficient variable selection using summary data from genome-wide association studies. Bioinformatics 32: 1493-1501; [b]posterior probability of the independent signal; [*]novel SNPs (+/-500kb from known)

Supplementary Table 17: Overlap Between Novel Loci and Significant S-PrediXcan Genes

gene	novel_locus_SNP	distance	phenotype	population	expression_model	effect_size	bonf_pvalue
COPA	rs11591179	52996	FEV1/FVC	ME	GTEx Whole Blood	-0.003106445	0.02030953
NCSTN	rs11591179	106995	FEV1/FVC	ME	GTEx Whole Blood	-0.002382718	0.026916837
AFAP1	rs28520091	85799	FEV1/FVC	Eur	GTEx Whole Blood	0.002025097	0.048553765
AFAP1	rs28520091	85799	FEV1/FVC	ME	GTEx Whole Blood	0.001785143	0.036518526
NRBF2	rs7899503	194418	FEV1	ME	GTEx Lung	48.4236672	0.009290803
ARHGEF17	11:73280955:GA_G	261621	FEV1/FVC	ME	GTEx Whole Blood	0.003437503	0.018688926
FAM168A	11:73280955:GA_G	169423	FEV1/FVC	ME	GTEx Whole Blood	0.007032284	0.015245291
MAP2K5	rs8025774	351771	FVC	ME	GTEx Lung	27.51606686	0.034740808
MAP2K5	rs8025774	351771	FVC	ME	GTEx Whole Blood	33.06787296	0.000133528
LRRC37A	rs199525	477735	FEV1	ME	GTEx Lung	-15.31036633	0.013632915
LRRC37A2	rs199525	258957	FEV1	ME	GTEx Lung	-16.04564124	0.002527071
LRRC37A2	rs199525	258957	FVC	ME	GTEx Lung	-16.56508937	0.03109477
WNT3	rs199525	7962	FEV1	ME	GTEx Lung	-54.39915418	0.000125326
WNT3	rs199525	7962	FVC	ME	GTEx Lung	-60.94877411	0.000225597
LRRC37A	rs916888	493034	FEV1	Eur	GTEx Lung	-19.92690597	0.009923734
LRRC37A2	rs916888	274256	FEV1	Eur	GTEx Lung	-19.29760479	0.002544678
LRRC37A2	rs916888	274256	FEV1	Eur	GTEx Whole Blood	-22.06716073	0.002916702
WNT3	rs916888	23261	FEV1	Eur	GTEx Lung	-62.99302499	0.000268613

This table shows the effect sizes and Bonferroni adjusted p-values of genes identified by S-PrediXcan in each tissue expression model in which the start of the gene is within 500kb (distance) of the novel locus SNP in the respective phenotype and population analysis. Methods reference: Barbeira, A. *et al.* Exploring the phenotypic consequences of tissue specific gene expression variation inferred from GWAS summary statistics. *Nat Commun* 9, 1825 (2018).

Supplementary Table 18: Bayesian Colocalization Results

gene	phenotype	population	expression_model	P_H0	P_H1	P_H2	P_H3	P_H4
AFAP1	FF	ME	GTEx Whole Blood	5.49E-115	2.66E-113	0.012697892	0.614780829	0.372521279
AFAP1	FF	EUR	GTEx Whole Blood	4.80E-117	6.23E-113	7.18E-05	0.930930279	0.068997936
ARHGEF17	FF	ME	GTEx Whole Blood	1.98E-19	2.35E-17	0.00242864	0.287352465	0.710218895
COPA	FF	ME	GTEx Whole Blood	1.76E-28	4.32E-25	0.000333231	0.818626522	0.181040247
FAM168A	FF	ME	GTEx Whole Blood	1.40E-06	0.000170701	0.000426979	0.051052103	0.948348815
LRRC37A	FEV	ME	GTEx Lung	4.08E-41	3.06E-35	1.14E-06	0.860022433	0.139976423
LRRC37A	FEV	EUR	GTEx Lung	1.99E-40	2.81E-35	5.50E-06	0.778963125	0.221031378
LRRC37A2	FEV	ME	GTEx Lung	4.28E-56	3.29E-50	1.03E-06	0.791729615	0.208269354
LRRC37A2	FVC	ME	GTEx Lung	2.37E-54	3.47E-50	5.70E-05	0.83589161	0.164051381
LRRC37A2	FEV	EUR	GTEx Lung	2.30E-55	3.28E-50	5.39E-06	0.767408964	0.232585646
LRRC37A2	FEV	EUR	GTEx Whole Blood	1.38E-35	1.96E-30	4.88E-06	0.694734481	0.305260639
MAP2K5	FVC	ME	GTEx Lung	3.91E-13	1.62E-06	2.41E-07	0.99924187	0.000756267
MAP2K5	FVC	ME	GTEx Whole Blood	1.29E-16	5.35E-10	2.41E-07	0.999224405	0.000775354
NCSTN	FF	ME	GTEx Whole Blood	6.33E-38	1.56E-34	0.000330447	0.81178213	0.187887423
NRBF2	FEV	ME	GTEx Lung	4.91E-09	0.169047278	2.33E-08	0.802572908	0.028379786
WNT3	FEV	ME	GTEx Lung	1.17E-11	2.47E-06	4.69E-06	0.986482502	0.013510343
WNT3	FVC	ME	GTEx Lung	1.37E-10	1.06E-06	5.46E-05	0.422288338	0.577656023
WNT3	FEV	EUR	GTEx Lung	4.30E-11	2.45E-06	1.72E-05	0.978831008	0.02114938

This table presents the Bayesian colocalization results for the genes identified in Supplementary table 17. The columns with the prefix P refer to the posterior probability of the following hypotheses: H0: no association with either trait, H1: association with trait 1 but not 2, H2: association with trait 2 but not 1, H3: association with trait 1 and 2 by two independent SNPs, H4: association with trait 1 and 2 by one shared SNP, with trait 1 being the phenotype and trait 2 being gene expression. Methods reference: Giambartolomei C, Vukcevic D, Schadt EE, Franke L, Hingorani AD, et al. (2014) Bayesian test for colocalisation between pairs of genetic association studies using summary statistics. PLoS Genet 10: e1004383.

Supplementary Table 19. Genes which encode proteins with predicted or known drug targets as identified in ChEMBL database

Gene	Sentinel Variant rsID	Sentinel Variant Chr:Pos	Durg target (ChEMBL ID)	Approved drugs and clinical candidates (ChEMBL ID)	Approved drugs and Clinical candidates (Name)	Indications [MeSH/EFO term] (Max phase for indication)
**BAZ2B**	rs13022640	2:160346488	CHEMBL1741220	No	No	
**CDK12**	rs8067511	17:37611352	CHEMBL3559691	CHEMBL445813	AT-7519	
				CHEMBL3544942	Roniciclib	
				CHEMBL488436	AZD-5438	
				CHEMBL1230607	PHA-793887	
**EDAR**	rs17034666	2:109571508	CHEMBL1250376	No	No	
**EYA2**	rs2236519	20:45529571	CHEMBL1293275	No	No	
**GSTO1**	rs10883990	10:106018558	CHEMBL3174	No	No	
**GSTO2**	rs17826034	10:106033504	CHEMBL2161	No	No	
**KCNJ3**	rs72904209	2:157046432	CHEMBL3038488	No	No	
**KCNK2**	rs512597	1:215095003	CHEMBL2321615	CHEMBL931	HALOTHANE	
				CHEMBL1256	ISOFLURANE	BREAST NEOPLASM (4), CORONARY HEART DISEASE (4), INJURY (4), OPIATE DEPENDENCE (2)
				CHEMBL1257	ENFLURANE	
				CHEMBL1200694	SEVOFLURANE	BREAST NEOPLASMS (4), CAROTID STENOSIS (4), COUGH (4), DEPRESSIVE DISORDER (4), HEART DISEASES (4), HEMORRHAGE (4), MYOCARDIAL INFARCTION (4), POSTOPERATIVE COMPLICATIONS (4), POSTOPERATIVE (4), NAUSEA AND VOMITING (4)
				CHEMBL1200733	DESFLURANE	AORTIC VALVE STENOSIS (4), OBESITY (4), PROSTATIC NEOPLASMS (4), PSYCHOMOTOR AGITATION (4), RENAL INSUFFICIENCY, CHRONIC (4), STRABISMUS (4), COLORECTAL NEOPLASMS (2), POSTOPERATIVE COMPLICATIONS (1)
**NANP**	rs6138616	20:25600835	CHEMBL2401602	No	No	
**NCOR1**	rs21180	17:16088008	CHEMBL2096976	No	No	
**NCOR2**	rs11057793	12:125230287	CHEMBL1961787	No	No	
**NR4A2**	rs72904209	2:157046432	CHEMBL5002	No	No	
**NR5A2**	rs2821332	1:200085714	CHEMBL3544	No	No	
**PYGB**	rs2258053	20:25271033	CHEMBL3856	No	No	
**SMAD3**	rs8025774	15:67483276	CHEMBL1293258	No	No	
**WNT3**	rs199525	17:44847834	CHEMBL5314	No	No	
**A4GNT**	rs9864090	3:137870554	CHEMBL6079	No	No	

Max phases for indication: Phase 1: Testing of drug on healthy volunteers for dose-ranging; Phase 2: Testing of drug on patients to assess efficacy and safety; Phase 3: Testing of drug on patients to assess efficacy, effectiveness and safety; Phase 4: Approval of drug and post-marketing surveillance

Supplementary Table 20. Genes which encode proteins with predicted or known drug targets identified by ingenuity pathway analysis (IPA)

ID	Location	Type(s)	Drug(s)
**ADORA2B**	Plasma Membrane	G-protein coupled receptor	adenosine, enprofylline, dyphylline, aspirin/butalbital/caffeine, acetaminophen/caffeine/dihydrocodeine, acetaminophen/aspirin/caffeine, caffeine/ergotamine, aspirin/caffeine/propoxyphene, aspirin/butalbital/caffeine/codeine, aspirin/caffeine/dihydrocodeine, acetaminophen/butalbital/caffeine, aspirin/caffeine/orphenadrine, acetaminophen/butalbital/caffeine/codeine, theophylline, caffeine, acetaminophen/caffeine/chlorpheniramine/hydrocodone/phenylephrine
**APP**	Plasma Membrane	other	bapineuzumab, florbetapir F18, florbetaben F
**CRHR1**	Plasma Membrane	G-protein coupled receptor	CRA0165, CRA1001, SSR125543A
**MAP3K1**	Cytoplasm	kinase	E 6201

Supplementary Table 21. 37 genes regulated by 88 upstream drug/chemicals predicted by ingenuity pathway analysis (IPA)

Upstream Regulator	Molecule Type	p-value of overlap	Target molecules in dataset
butylphen	chemical toxicant	3.00E-02	ADORA2B
prostaglandin E2	chemical - endogenous mammalian	4.93E-04	ADORA2B,APP,EGR2,ETS1,HDC,IER3,NR4A2,NR5A2
doxorubicin	chemical drug	2.97E-02	ADORA2B,APP,IER3,RYR2,SMAD3,WNT3
U0126	chemical - kinase inhibitor	2.88E-02	ADORA2B,CDH5,DUSP10,ETS1,IER3,NR4A2,SMAD3
paclitaxel	chemical drug	3.31E-02	ADORA2B,DUSP10,IER3,MAP3K1,WNT3
5-N-ethylcarboxamido adenosine	chemical reagent	2.63E-02	ADORA2B,EGR2,NR4A2
poly rI:rC-RNA	biologic drug	4.59E-02	AFAP1,ELAVL2,MARCO,NR4A2,SCARB1,SUOX
bapineuzumab	biologic drug	6.08E-03	APP
triacylglycerol	chemical - endogenous mammalian	3.00E-02	APP
ganglioside GM1	chemical - endogenous mammalian	4.76E-02	APP
E64	chemical - endogenous non-mammalian	1.81E-02	APP
protocatechuic acid	chemical - endogenous non-mammalian	2.41E-02	APP
sphingosine kinase inhibitor V	chemical - kinase inhibitor	6.08E-03	APP
IC261	chemical - kinase inhibitor	6.08E-03	APP
MW-III-36C	chemical - protease inhibitor	6.08E-03	APP
Z-IL-CHO	chemical - protease inhibitor	1.21E-02	APP
aleplasinin	chemical drug	6.08E-03	APP
acetylcholinesterase inhibitor	chemical drug	6.08E-03	APP
CHF 5074	chemical drug	6.08E-03	APP
LY2811376	chemical drug	6.08E-03	APP
ABC-294640	chemical drug	6.08E-03	APP
meclofenamic acid	chemical drug	6.08E-03	APP
diflunisal	chemical drug	6.08E-03	APP
pinacidil	chemical drug	6.08E-03	APP
donepezil	chemical drug	6.08E-03	APP
PK 11195	chemical drug	6.08E-03	APP
fenoprofen	chemical drug	1.21E-02	APP
arundic acid	chemical drug	1.81E-02	APP
cromolyn	chemical drug	2.41E-02	APP
sertindole	chemical drug	2.41E-02	APP
aurintricarboxylic acid	chemical drug	3.59E-02	APP
dantrolene	chemical drug	4.76E-02	APP
N6-methyl-(R)-roscovitine	chemical reagent	6.08E-03	APP

BTA-EG4	chemical reagent	6.08E-03	APP
EG443	chemical reagent	6.08E-03	APP
MC622	chemical reagent	6.08E-03	APP
MC55w21	chemical reagent	6.08E-03	APP
MC55w10	chemical reagent	6.08E-03	APP
CRANAD-17	chemical reagent	6.08E-03	APP
GF 120918	chemical reagent	6.08E-03	APP
RS86	chemical reagent	6.08E-03	APP
D-2-amino-5-phosphonovaleric acid	chemical reagent	2.41E-02	APP
(-)-arctigenin	chemical reagent	3.59E-02	APP
3,5-dihydroxyphenylglycine	chemical reagent	3.59E-02	APP
Congo Red	chemical toxicant	3.59E-02	APP
MDL 28170	chemical toxicant	4.76E-02	APP
beta-estradiol	chemical - endogenous mammalian	3.99E-02	APP,CPT1C,CRADD,CRHR1,DDR1,DUSP10,EGR2,ETS1,HTRA1,IER3,NCOR1,NR5A2,PIK3C2B,RBBP8,SCARB1,SMAD3,WNT3
corticosterone	chemical - endogenous mammalian	3.24E-02	APP,CRHR1,SCARB1
benzyloxycarbonyl-Leu-Leu-Leu aldehyde	chemical - protease inhibitor	3.65E-02	APP,DCC,ETS1,IER3,NCOR1
N-Ac-Leu-Leu-norleucinal	chemical - protease inhibitor	1.38E-03	APP,DCC,IER3,SCARB1
kainic acid	chemical toxicant	1.16E-02	APP,EGR2,HDC,NR4A2
bicuculline	chemical - endogenous non-mammalian	1.71E-03	APP,EGR2,NR4A2
dalfampridine	chemical drug	9.57E-04	APP,EGR2,NR4A2
H89	chemical - kinase inhibitor	9.59E-03	APP,EGR2,NR4A2,SMAD3
TO-901317	chemical reagent	4.93E-02	APP,NR5A2,SCARB1,SMAD3
acetovanillone	chemical drug	1.81E-02	APP,RYR2
farnesyl pyrophosphate	chemical - endogenous mammalian	3.18E-03	APP,SMAD3
maslinic acid	chemical - endogenous non-mammalian	1.37E-02	CAMK2G,DCC,ETS1
medroxyprogesterone acetate	chemical drug	4.25E-02	CENPF,ETS1,HTRA1
potassium chloride	chemical drug	1.65E-03	CRADD,EGR2,NR4A2,RYR2
PD98059	chemical - kinase inhibitor	1.89E-02	DCBLD2,EGR2,ETS1,HPCAL1,IER3,PPP1R10,SCARB1,SMAD3
N-acetylmuramyl-L-alanyl-D-isoglutamine	chemical - endogenous non-mammalian	4.71E-02	DMBT1,ETS1
zinostatin	biologic drug	4.18E-02	DUSP10
edratide	biologic drug	3.00E-02	EGR2
leukotriene D4	chemical - endogenous mammalian	4.57E-03	EGR2,HLX,NR4A2

ST3-Hel2A-2	chemical reagent	3.49E-05	EGR2,IER3,NR4A2
KN 93	chemical - kinase inhibitor	1.34E-02	ETS1,NR4A2
Go 6976	chemical - kinase inhibitor	4.27E-02	ETS1,NR4A2
H-7	chemical - kinase inhibitor	2.46E-02	HDC,SMAD3
CC8490	chemical drug	2.41E-02	IER3
gliotoxin	chemical toxicant	4.18E-02	IER3
SU6656	chemical toxicant	1.71E-02	IER3,NR4A2
7-dehydrocholesterol	chemical - endogenous mammalian	1.21E-02	MAP3K1
fish oils	chemical drug	3.45E-02	MED1,NCOR2
7beta-hydroxycholesterol	chemical - endogenous mammalian	1.21E-02	NR4A2
(+)-fluprostenol	chemical drug	1.81E-02	NR4A2
fluphenazine	chemical drug	3.59E-02	NR4A2
raclopride	chemical drug	4.18E-02	NR4A2
JTV519	chemical drug	1.21E-02	RYR2
cholesterol ester	chemical - endogenous mammalian	6.08E-03	SCARB1
acetone	chemical - endogenous non-mammalian	4.76E-02	SCARB1
Rp-8-CPT-cAMPS	chemical - kinase inhibitor	6.08E-03	SCARB1
resatorvid	chemical drug	3.00E-02	SCARB1
olmesartan	chemical drug	3.00E-02	SCARB1
sobetirome	chemical drug	3.00E-02	SCARB1
urotensin II	biologic drug	4.18E-02	SMAD3
pectin	chemical drug	6.08E-03	SMAD3
LGK-974	chemical drug	3.00E-02	WNT3

Supplementary Table 22. Characteristics of cohorts and participants included in the CHARGE consortium 1000 Genomes and pulmonary function meta-analysis

					Demographics							Smoking				Pulmonary function					
					Gender	Age (y)		Height (cm)		Weight (kg)		Status		Pack-years		FEV_1 (ml)		FVC (ml)		FEV_1/FVC (proportion)	
Cohort	Country	Ancestral Population	Total Sample Size	Analysis Sample Size	% Male	Mean	s.d.	Mean	s.d.	Mean	s.d.	% Former Smoking	% Current Smoking	Mean	s.d.	Mean	s.d.	Mean	s.d.	Mean	s.d.
AGES	Iceland	European	1706	1620	40.62	76.20	5.62	166.69	9.40	75.39	14.71	44.29	12.76	24.54	21.92	2125.82	690.56	2863.08	849.53	0.74	0.10
ALHS Cases	United States	European	1198	1180	47.83	62.36	10.71	168.48	9.69	88.75	20.58	30.01	4.65	16.99	20.49	2391.37	827.91	3370.30	1011.02	0.71	0.11
ALHS Controls	United States	European	1693	1664	53.63	63.63	11.11	169.85	9.48	85.12	18.57	29.13	4.14	17.90	21.04	2705.62	787.81	3617.77	996.75	0.75	0.07
ARIC	United States	European	8992	8878	46.94	54.76	5.70	168.73	9.41	77.12	16.28	35.96	24.86	28.66	21.72	2942.94	770.34	3989.97	980.48	0.74	0.08
		African	1900	1837	37.53	54.34	5.87	167.99	9.05	83.84	17.60	23.47	31.89	23.10	21.77	2456.25	650.10	3230.40	822.34	0.76	0.08
CARDIA	United States	European	1580	1580	47.28	25.60	3.33	171.32	9.27	69.85	14.32	17.13	25.20	6.10	6.67	3842.2	783.25	4696.68	1011.11	0.82	0.06
		African	883	883	39.93	24.44	3.85	169.01	9.58	73.10	16.96	8.60	30.09	4.15	4.37	3219.99	679.76	3854.24	861.81	0.84	0.06
CHS	United States	European	3221	3135	39.10	72.33	5.35	164.64	9.35	71.41	14.16	40.90	11.30	33.18	26.95	2114.47	658.64	3004.82	865.22	0.70	0.10
		African	635	566	37.50	73.09	5.42	164.92	9.22	77.21	14.86	42.40	14.80	26.80	24.75	1751.26	581.19	2491.00	799.18	0.71	0.12
FamHS	United States	European	1728	1679	56.40	53.49	12.62	168.64	9.65	80.27	20.71	30.96	11.34	27.12	24.88	2913.51	852.65	3896.57	1049.79	0.75	0.08
FHS	United States	European	7909	7689	46.18	52.22	14.63	168.48	9.69	77.85	17.63	39.97	15.09	12.25	19.43	3029.87	941.87	4016.19	1142.42	0.75	0.08
GOYA Cases	Denmark	European	670	670	100.00	43.00	6.20	178.10	6.20	113.00	19.20	22.60	52.90	23.67	22.00	3815.00	763.60	4758.00	877.10	0.80	0.07
GOYA Controls	Denmark	European	786	786	100.00	47.70	8.50	177.40	6.20	82.20	12.30	27.70	50.00	20.60	17.80	3746.00	760.60	4769.00	878.70	0.78	0.07
HCHS/SOL	United States	Central American	1324	1294	40.41	44.43	13.15	160.10	8.74	76.93	16.43	19.79	13.60	11.57	16.68	2881.69	742.96	3540.02	889.15	0.81	0.06
		Cuban	2161	2121	46.78	48.69	13.13	164.13	8.74	79.07	17.62	20.50	28.83	25.00	24.32	2799.31	802.57	3554.07	931.77	0.79	0.08
		Dominican	1077	1067	34.73	45.38	14.37	162.01	8.99	77.19	16.70	13.83	10.40	14.08	15.83	2696.31	758.74	3291.32	875.09	0.82	0.07
		Mexican	4587	4434	39.79	44.46	13.78	161.41	9.34	77.89	17.60	20.84	16.02	8.70	12.41	2971.65	784.02	3682.89	950.47	0.81	0.06
		Puerto Rican	2040	2001	43.19	47.49	14.36	163.26	9.39	82.19	20.01	20.29	32.60	17.97	20.46	2701.41	832.56	3412.29	1017.73	0.79	0.08
		South American	870	858	39.43	46.66	13.23	160.72	9.17	73.87	15.22	21.15	14.02	12.25	18.22	2940.66	784.26	3680.14	959.21	0.80	0.07
HCS	Australia	European	1876	1822	49.90	66.10	7.43	166.24	9.19	79.63	15.72	38.50	6.45	23.43	26.32	2436.94	685.08	2963.11	829.92	0.83	0.08
Health ABC	United States	European	1499	1472	53.40	73.72	2.83	167.09	9.25	74.65	14.48	49.86	6.59	36.59	32.00	2311.79	656.15	3113.30	812.18	0.74	0.08
		African	952	943	45.92	73.42	2.90	165.88	9.37	78.09	15.24	40.30	17.29	29.52	24.13	1955.55	573.78	2609.17	712.11	0.75	0.09
Healthy Twin	South Korea	Asian	2202	2098	39.37	45.58	13.70	161.73	8.71	62.53	11.32	12.85	21.53	18.67	18.16	2891.02	720.18	3613.55	865.28	0.80	0.07
JHS	United States	African	3029	2015	39.05	49.86	11.88	169.80	9.36	93.14	22.51	16.51	14.68	29.59	29.11	2523.00	709.40	3102.00	850.88	0.82	0.08
KARE3	South Korea	Asian	7921	7861	47.67	54.86	8.85	160.17	8.72	63.08	9.91	18.23	20.58	26.29	18.58	2793.19	684.07	3547.79	865.88	0.79	0.07
LifeLines	Netherlands	European	12461	11851	41.30	48.35	11.22	174.37	9.21	80.17	14.95	36.70	22.40	13.14	11.50	3379.35	834.91	4431.24	1025.69	0.76	0.07
LLFS	United States and Denmark	European	3899	3787	44.00	68.81	15.18	166.12	10.34	75.25	16.52	36.00	7.00	21.61	21.78	2454.62	865.06	3202.39	1052.33	0.76	0.07
MESA	United States	European	1400	1339	50.50	66.09	9.72	168.90	9.60	80.21	17.59	49.14	7.93	29.21	28.39	2573.00	763.48	3524.00	994.97	0.73	0.09
		African	909	863	48.51	65.63	9.60	168.50	9.70	84.81	17.38	40.48	14.74	24.47	23.49	2198.00	657.84	2929.00	855.87	0.75	0.09
NEO	Netherlands	European	5572	5460	48.00	55.90	5.95	173.70	9.58	90.60	16.60	50.00	16.00	18.38	18.34	3263.00	800.00	4260.00	1022.00	0.77	0.07
1982 Pelotas	Brazil	European	1364	1357	50.22	30.18	0.35	168.58	9.18	76.59	17.75	17.74	21.63	7.15	6.76	3528.26	755.16	4297.02	942.43	0.82	0.06
		African	1328	1322	48.27	30.17	0.34	166.82	9.18	75.60	18.01	19.95	25.68	6.99	7.17	3350.67	714.92	4080.14	875.00	0.82	0.06
RS I	Netherlands	European	1233	1232	42.50	78.74	4.62	165.76	9.26	75.71	13.27			15.38	20.68	2193.37	653.96	2917.60	825.86	0.75	0.08
RS II	Netherlands	European	1137	1135	46.40	71.81	4.97	168.64	9.00	78.53	13.54	55.80	10.80	23.04	21.80	2488.65	688.90	3276.58	867.59	0.76	0.08
RS III	Netherlands	European	2217	2216	43.90	61.80	5.58	171.65	9.37	81.40	15.68	51.60	14.70	20.83	20.54	2939.59	783.55	3857.19	1007.60	0.76	0.07

Abbreviations: AGES Age Gene Environment Susceptibility Study; ALHS Agricultural Lung Health Study; ARIC Atherosclerosis Risk in Communities Study; CARDIA Coronary Artery Risk Development in Young Adults; CHS Cardiovascular Health Study; FHS Framingham Heart Study; GOYA Genetics of Overweight Young Adults Study; HCHS/SOL Hispanic Community Health Study/Study of Latinos; HCS Hunter Community Study; JHS Jackson Heart Study; KARE3 Korean Association Resource Phase 3 Study; LLFS Long Life Family Study; MESA Multi-Ethnic Study of Atherosclerosis; NEO Netherlands Epidemiology of Obesity Study; RS Rotterdam Study

Supplementary Table 23. Genomic inflation factors (lambda values) from quantile-quantile plots of observed and expected p-values for ancestry- and phenotype-specific meta-analyses of 1000 Genomes imputed variants and pulmonary function in the CHARGE consortium

	**FEV_1**	**FVC**	**Ratio**
**European**	1.13	1.13	1.12
**African**	1.05	1.06	1.04
**Asian**	1.03	1.03	1.01
**Hispanic**	1.03	1.02	1.02
**Multiethnic**	1.16	1.16	1.14

Supplementary References

1. Hobbs BD, de Jong K, Lamontagne M, Bosse Y, Shrine N, et al. (2017) Genetic loci associated with chronic obstructive pulmonary disease overlap with loci for lung function and pulmonary fibrosis. Nat Genet 49: 426-432.
2. Loth DW, Soler Artigas M, Gharib SA, Wain LV, Franceschini N, et al. (2014) Genome-wide association analysis identifies six new loci associated with forced vital capacity. Nat Genet 46: 669-677.
3. Carnes MU, Hoppin JA, Metwali N, Wyss AB, Hankinson JL, et al. (2017) House Dust Endotoxin Levels Are Associated with Adult Asthma in a U.S. Farming Population. Ann Am Thorac Soc 14: 324-331.
4. House JS, Wyss AB, Hoppin JA, Richards M, Long S, et al. (2017) Early-life farm exposures and adult asthma and atopy in the Agricultural Lung Health Study. J Allergy Clin Immunol 140: 249-256 e214.
5. Wyss AB, House JS, Hoppin JA, Richards M, Hankinson JL, et al. (2017) Raw milk consumption and other early-life farm exposures and adult pulmonary function in the Agricultural Lung Health Study. Thorax.
6. (1989) The Atherosclerosis Risk in Communities (ARIC) Study: design and objectives. The ARIC investigators. Am J Epidemiol 129: 687-702.
7. Friedman GD, Cutter GR, Donahue RP, Hughes GH, Hulley SB, et al. (1988) CARDIA: study design, recruitment, and some characteristics of the examined subjects. J Clin Epidemiol 41: 1105-1116.
8. Hughes GH, Cutter G, Donahue R, Friedman GD, Hulley S, et al. (1987) Recruitment in the Coronary Artery Disease Risk Development in Young Adults (Cardia) Study. Control Clin Trials 8: 68S-73S.
9. (1979) ATS statement--Snowbird workshop on standardization of spirometry. Am Rev Respir Dis 119: 831-838.
10. Fried LP, Borhani NO, Enright P, Furberg CD, Gardin JM, et al. (1991) The Cardiovascular Health Study: design and rationale. Ann Epidemiol 1: 263-276.
11. Howie B, Fuchsberger C, Stephens M, Marchini J, Abecasis GR (2012) Fast and accurate genotype imputation in genome-wide association studies through pre-phasing. Nat Genet 44: 955-959.
12. Higgins M, Province M, Heiss G, Eckfeldt J, Ellison RC, et al. (1996) NHLBI Family Heart Study: objectives and design. Am J Epidemiol 143: 1219-1228.
13. Heath SC (1997) Markov chain Monte Carlo segregation and linkage analysis for oligogenic models. Am J Hum Genet 61: 748-760.
14. Abecasis GR, Auton A, Brooks LD, DePristo MA, Durbin RM, et al. (2012) An integrated map of genetic variation from 1,092 human genomes. Nature 491: 56-65.
15. Li Y, Willer C, Sanna S, Abecasis G (2009) Genotype imputation. Annu Rev Genomics Hum Genet 10: 387-406.
16. Li Y, Willer CJ, Ding J, Scheet P, Abecasis GR (2010) MaCH: using sequence and genotype data to estimate haplotypes and unobserved genotypes. Genet Epidemiol 34: 816-834.
17. Hancock DB, Eijgelsheim M, Wilk JB, Gharib SA, Loehr LR, et al. (2010) Meta-analyses of genome-wide association studies identify multiple loci associated with pulmonary function. Nat Genet 42: 45-52.
18. Wilk JB, Shrine NR, Loehr LR, Zhao JH, Manichaikul A, et al. (2012) Genome-wide association studies identify CHRNA5/3 and HTR4 in the development of airflow obstruction. Am J Respir Crit Care Med 186: 622-632.
19. Genomes Project C, Abecasis GR, Auton A, Brooks LD, DePristo MA, et al. (2012) An integrated map of genetic variation from 1,092 human genomes. Nature 491: 56-65.
20. Hollensted M, Ahluwalia TS, Have CT, Grarup N, Fonvig CE, et al. (2015) Common variants in LEPR, IL6, AMD1, and NAMPT do not associate with risk of juvenile and childhood obesity in Danes: a case-control study. BMC Med Genet 16: 105.

21. Paternoster L, Evans DM, Nohr EA, Holst C, Gaborieau V, et al. (2011) Genome-wide population-based association study of extremely overweight young adults--the GOYA study. PLoS One 6: e24303.
22. Lavange LM, Kalsbeek WD, Sorlie PD, Aviles-Santa LM, Kaplan RC, et al. (2010) Sample design and cohort selection in the Hispanic Community Health Study/Study of Latinos. Ann Epidemiol 20: 642-649.
23. Sorlie PD, Aviles-Santa LM, Wassertheil-Smoller S, Kaplan RC, Daviglus ML, et al. (2010) Design and implementation of the Hispanic Community Health Study/Study of Latinos. Ann Epidemiol 20: 629-641.
24. Barr RG, Aviles-Santa L, Davis SM, Aldrich TK, Gonzalez F, 2nd, et al. (2016) Pulmonary Disease and Age at Immigration among Hispanics. Results from the Hispanic Community Health Study/Study of Latinos. Am J Respir Crit Care Med 193: 386-395.
25. Rosenberg NA, Li LM, Ward R, Pritchard JK (2003) Informativeness of genetic markers for inference of ancestry. Am J Hum Genet 73: 1402-1422.
26. Laurie CC, Doheny KF, Mirel DB, Pugh EW, Bierut LJ, et al. (2010) Quality control and quality assurance in genotypic data for genome-wide association studies. Genet Epidemiol 34: 591-602.
27. Conomos MP, Laurie CA, Stilp AM, Gogarten SM, McHugh CP, et al. (2016) Genetic Diversity and Association Studies in US Hispanic/Latino Populations: Applications in the Hispanic Community Health Study/Study of Latinos. Am J Hum Genet 98: 165-184.
28. Pellegrino R, Viegi G, Brusasco V, Crapo RO, Burgos F, et al. (2005) Interpretative strategies for lung function tests. Eur Respir J 26: 948-968.
29. Waterer GW, Wan JY, Kritchevsky SB, Wunderink RG, Satterfield S, et al. (2001) Airflow limitation is underrecognized in well-functioning older people. J Am Geriatr Soc 49: 1032-1038.
30. Gombojav B, Song YM, Lee K, Yang S, Kho M, et al. (2013) The Healthy Twin Study, Korea updates: resources for omics and genome epidemiology studies. Twin Res Hum Genet 16: 241-245.
31. Sung J, Cho SH, Cho SI, Duffy DL, Kim JH, et al. (2002) The Korean Twin Registry--methods, current stage, and interim results. Twin Res 5: 394-400.
32. Sung J, Cho SI, Lee K, Ha M, Choi EY, et al. (2006) Healthy Twin: a twin-family study of Korea--protocols and current status. Twin Res Hum Genet 9: 844-848.
33. Taylor HA, Jr. (2005) The Jackson Heart Study: an overview. Ethn Dis 15: S6-1-3.
34. Taylor HA, Jr., Wilson JG, Jones DW, Sarpong DF, Srinivasan A, et al. (2005) Toward resolution of cardiovascular health disparities in African Americans: design and methods of the Jackson Heart Study. Ethn Dis 15: S6-4-17.
35. Wilson JG, Rotimi CN, Ekunwe L, Royal CD, Crump ME, et al. (2005) Study design for genetic analysis in the Jackson Heart Study. Ethn Dis 15: S6-30-37.
36. Cho YS, Go MJ, Kim YJ, Heo JY, Oh JH, et al. (2009) A large-scale genome-wide association study of Asian populations uncovers genetic factors influencing eight quantitative traits. Nat Genet 41: 527-534.
37. Kim WJ, Lee MK, Shin C, Cho NH, Lee SD, et al. (2014) Genome-wide association studies identify locus on 6p21 influencing lung function in the Korean population. Respirology 19: 360-368.
38. Purcell S PLINK [version 1.07]. URL: http://pngu.mgh.harvard.edu/purcell/plink/.
39. de Jong K, Vonk JM, Kromhout H, Vermeulen R, Postma DS, et al. (2014) NOS1: a susceptibility gene for reduced level of FEV1 in the setting of pesticide exposure. Am J Respir Crit Care Med 190: 1188-1190.
40. de Jong K, Vonk JM, Timens W, Bosse Y, Sin DD, et al. (2015) Genome-wide interaction study of gene-by-occupational exposure and effects on FEV1 levels. J Allergy Clin Immunol 136: 1664-1672 e1661-1614.

41. Scholtens S, Smidt N, Swertz MA, Bakker SJ, Dotinga A, et al. (2015) Cohort Profile: LifeLines, a three-generation cohort study and biobank. Int J Epidemiol 44: 1172-1180.
42. Purcell S, Neale B, Todd-Brown K, Thomas L, Ferreira MA, et al. (2007) PLINK: a tool set for whole-genome association and population-based linkage analyses. Am J Hum Genet 81: 559-575.
43. Delaneau O, Zagury JF, Marchini J (2013) Improved whole-chromosome phasing for disease and population genetic studies. Nat Methods 10: 5-6.
44. Pedersen CB, Gotzsche H, Moller JO, Mortensen PB (2006) The Danish Civil Registration System. A cohort of eight million persons. Dan Med Bull 53: 441-449.
45. Sebastiani P, Hadley EC, Province M, Christensen K, Rossi W, et al. (2009) A family longevity selection score: ranking sibships by their longevity, size, and availability for study. Am J Epidemiol 170: 1555-1562.
46. Price AL, Patterson NJ, Plenge RM, Weinblatt ME, Shadick NA, et al. (2006) Principal components analysis corrects for stratification in genome-wide association studies. Nat Genet 38: 904-909.
47. Bild DE, Bluemke DA, Burke GL, Detrano R, Diez Roux AV, et al. (2002) Multi-Ethnic Study of Atherosclerosis: objectives and design. Am J Epidemiol 156: 871-881.
48. Kaufman JD, Adar SD, Allen RW, Barr RG, Budoff MJ, et al. (2012) Prospective study of particulate air pollution exposures, subclinical atherosclerosis, and clinical cardiovascular disease: The Multi-Ethnic Study of Atherosclerosis and Air Pollution (MESA Air). Am J Epidemiol 176: 825-837.
49. Hankinson JL, Kawut SM, Shahar E, Smith LJ, Stukovsky KH, et al. (2010) Performance of American Thoracic Society-recommended spirometry reference values in a multiethnic sample of adults: the multi-ethnic study of atherosclerosis (MESA) lung study. Chest 137: 138-145.
50. Manichaikul A, Naj AC, Herrington D, Post W, Rich SS, et al. (2012) Association of SCARB1 variants with subclinical atherosclerosis and incident cardiovascular disease: the multi-ethnic study of atherosclerosis. Arterioscler Thromb Vasc Biol 32: 1991-1999.
51. Marchini J, Howie B (2010) Genotype imputation for genome-wide association studies. Nat Rev Genet 11: 499-511.
52. de Mutsert R, den Heijer M, Rabelink TJ, Smit JW, Romijn JA, et al. (2013) The Netherlands Epidemiology of Obesity (NEO) study: study design and data collection. Eur J Epidemiol 28: 513-523.
53. Horta BL, Gigante DP, Goncalves H, dos Santos Motta J, Loret de Mola C, et al. (2015) Cohort Profile Update: The 1982 Pelotas (Brazil) Birth Cohort Study. Int J Epidemiol 44: 441, 441a-441e.
54. Victora CG, Barros FC (2006) Cohort profile: the 1982 Pelotas (Brazil) birth cohort study. Int J Epidemiol 35: 237-242.
55. Hofman A, Brusselle GG, Darwish Murad S, van Duijn CM, Franco OH, et al. (2015) The Rotterdam Study: 2016 objectives and design update. Eur J Epidemiol 30: 661-708.
56. Kreiner-Moller E, Medina-Gomez C, Uitterlinden AG, Rivadeneira F, Estrada K (2015) Improving accuracy of rare variant imputation with a two-step imputation approach. Eur J Hum Genet 23: 395-400.
57. Aulchenko YS, Struchalin MV, van Duijn CM (2010) ProbABEL package for genome-wide association analysis of imputed data. BMC Bioinformatics 11: 134.
58. Wain LV, Shrine N, Miller S, Jackson VE, Ntalla I, et al. (2015) Novel insights into the genetics of smoking behaviour, lung function, and chronic obstructive pulmonary disease (UK BiLEVE): a genetic association study in UK Biobank. Lancet Respir Med 3: 769-781.
59. Miller MR, Hankinson J, Brusasco V, Burgos F, Casaburi R, et al. (2005) Standardisation of spirometry. Eur Respir J 26: 319-338.
60. Consortium UK, Walter K, Min JL, Huang J, Crooks L, et al. (2015) The UK10K project identifies rare variants in health and disease. Nature 526: 82-90.

61. Regan EA, Hokanson JE, Murphy JR, Make B, Lynch DA, et al. (2010) Genetic epidemiology of COPD (COPDGene) study design. COPD 7: 32-43.
62. Cho MH, McDonald ML, Zhou X, Mattheisen M, Castaldi PJ, et al. (2014) Risk loci for chronic obstructive pulmonary disease: a genome-wide association study and meta-analysis. Lancet Respir Med 2: 214-225.
63. Levin AM, Wang Y, Wells KE, Padhukasahasram B, Yang JJ, et al. (2014) Nocturnal asthma and the importance of race/ethnicity and genetic ancestry. Am J Respir Crit Care Med 190: 266-273.
64. Padhukasahasram B, Yang JJ, Levin AM, Yang M, Burchard EG, et al. (2014) Gene-based association identifies SPATA13-AS1 as a pharmacogenomic predictor of inhaled short-acting beta-agonist response in multiple population groups. Pharmacogenomics J 14: 365-371.
65. Chang CC, Chow CC, Tellier LC, Vattikuti S, Purcell SM, et al. (2015) Second-generation PLINK: rising to the challenge of larger and richer datasets. Gigascience 4: 7.
66. Borrell LN, Nguyen EA, Roth LA, Oh SS, Tcheurekdjian H, et al. (2013) Childhood obesity and asthma control in the GALA II and SAGE II studies. Am J Respir Crit Care Med 187: 697-702.
67. Drake KA, Torgerson DG, Gignoux CR, Galanter JM, Roth LA, et al. (2014) A genome-wide association study of bronchodilator response in Latinos implicates rare variants. J Allergy Clin Immunol 133: 370-378.
68. Obeidat M, Miller S, Probert K, Billington CK, Henry AP, et al. (2013) GSTCD and INTS12 regulation and expression in the human lung. PLoS One 8: e74630.
69. Hao K, Bosse Y, Nickle DC, Pare PD, Postma DS, et al. (2012) Lung eQTLs to help reveal the molecular underpinnings of asthma. PLoS Genet 8: e1003029.
70. Obeidat M, Hao K, Bosse Y, Nickle DC, Nie Y, et al. (2015) Molecular mechanisms underlying variations in lung function: a systems genetics analysis. Lancet Respir Med 3: 782-795.
71. Wain LV, Shrine N, Artigas MS, Erzurumluoglu AM, Noyvert B, et al. (2017) Genome-wide association analyses for lung function and chronic obstructive pulmonary disease identify new loci and potential druggable targets. Nat Genet 49: 416-425.
72. Irizarry RA, Hobbs B, Collin F, Beazer-Barclay YD, Antonellis KJ, et al. (2003) Exploration, normalization, and summaries of high density oligonucleotide array probe level data. Biostatistics 4: 249-264.
73. Westra HJ, Peters MJ, Esko T, Yaghootkar H, Schurmann C, et al. (2013) Systematic identification of trans eQTLs as putative drivers of known disease associations. Nat Genet 45: 1238-1243.
74. Zhernakova DV, Deelen P, Vermaat M, van Iterson M, van Galen M, et al. (2017) Identification of context-dependent expression quantitative trait loci in whole blood. Nat Genet 49: 139-145.
75. Bonder MJ, Luijk R, Zhernakova DV, Moed M, Deelen P, et al. (2017) Disease variants alter transcription factor levels and methylation of their binding sites. Nat Genet 49: 131-138.
76. Joehanes R, Zhang X, Huan T, Yao C, Ying SX, et al. (2017) Integrated genome-wide analysis of expression quantitative trait loci aids interpretation of genomic association studies. Genome Biol 18: 16.
77. Mendelson MM, Marioni RE, Joehanes R, Liu C, Hedman AK, et al. (2017) Association of Body Mass Index with DNA Methylation and Gene Expression in Blood Cells and Relations to Cardiometabolic Disease: A Mendelian Randomization Approach. PLoS Med 14: e1002215.
78. Love MI, Huber W, Anders S (2014) Moderated estimation of fold change and dispersion for RNA-seq data with DESeq2. Genome Biol 15: 550.
79. Stegle O, Parts L, Piipari M, Winn J, Durbin R (2012) Using probabilistic estimation of expression residuals (PEER) to obtain increased power and interpretability of gene expression analyses. Nat Protoc 7: 500-507.
80. Shabalin AA (2012) Matrix eQTL: ultra fast eQTL analysis via large matrix operations. Bioinformatics 28: 1353-1358.

81. Mogil LS, Andaleon A, Badalamenti A, Dickinson SP, Guo X, et al. (2018) Genetic architecture of gene expression traits across diverse populations. bioRxiv.
