# Supplementary material for: Multiethnic meta-analysis identifies ancestry-specific and cross-ancestry loci for pulmonary function
Source: Nat Commun. 2018 Jul 30;9:2976. doi: 10.1038/s41467-018-05369-0 (PMC6065313; doi:10.1038/s41467-018-05369-0)
Supplement: Supplementary file 2 — Description of Additional Supplementary Files [file 41467_2018_5369_MOESM2_ESM.pdf]

Description of Additional Supplementary Files

File Name: Supplementary Data 1
Description: Deleterious (CADD scaled C-score>15) or regulatory (RegulomeDB category.

File Name: Supplementary Data 2
Description: Gene set enrichment results for FEV1/FVC in European ancestry using DEPICT.

File Name: Supplementary Data 3
Description: Gene prioritization results for FEV1, FVC, and FEV1/FVC in European ancestry using DEPICT.
